# Assessment of airborne bacteria from a public health institution in Mexico City

**DOI:** 10.1371/journal.pgph.0003672

**Published:** 2024-11-07

**Authors:** Maria Carmen Calderón-Ezquerro, Alfredo Ponce de León A., Carolina Brunner-Mendoza, César Guerrero-Guerra C., Alejandro Sanchez-Flores, Ilse Salinas-Peralta, Luis Esau López Jacome, Claudia Adriana Colín Castro C., María Guadalupe Martínez Zavaleta

**Affiliations:** 1 Departamento de Ciencias Ambientales, Instituto de Ciencias de la Atmósfera y Cambio Climático, UNAM, Mexico City, México; 2 Departamento de Infectología, Instituto Nacional de Ciencias Médicas y Nutrición Salvador Zubirán, Mexico City, México; 3 Facultad de Medicina, Departamento de Microbiología y Parasitología, UNAM, Mexico City, México; 4 Instituto de Biotecnología, Unidad Universitaria de Secuenciación Masiva y Bioinformática, UNAM, Morelos, México; 5 Laboratorio de Microbiología Clínica, Instituto Nacional de Rehabilitación Luis Guillermo Ibarra Ibarra, Mexico City, México; 6 Facultad de Química, Laboratorio, UNAM, Mexico City, México; University of Oslo Faculty of Medicine: Universitetet i Oslo Det medisinske fakultet, NORWAY

## Abstract

In this work, the composition of the bacterial community in the air of a hospital in Mexico City was evaluated using metabarcoding and proteomics approaches, along with the assessment of environmental factors such as temperature, humidity, and suspended particles. Two types of aerobiological samplers were used: Andersen One-Stage Viable Particle Sampler (AVPS) and Coriolis μ sampler (CμS-Sampler). Sampling was performed in four areas of the hospital: Floor 1 (F1), Floor 2 (F2), and Emergency Unit (EU), as well as outdoors (OH). The use of both samplers showed variations in diversity and composition. Bacterial abundance was 89.55% with the CμS-Sampler and 74.00% with the AVPS. The predominant phyla with the AVPS were Firmicutes, Proteobacteria and Actinobacteria, while with the CμS-Sampler, the main phyla were Proteobacteria, followed by Actinobacteria and Firmicutes. The highest diversity and richness of bacteria was recorded in F1 and F2, with 32 species identified, with a greater number within the hospital. Potentially pathogenic bacteria such as *Bacillus* spp., B. *cereus*, *B*. *pumilus*, *Clostridium* spp., *Enterococcus gallinarum*, *Micrococcus luteus* and *Staphylococcus* spp. were detected. Furthermore, a high concentration of particles between 2.5 μm and 10 μm, and Total Particulate Matter (TPM) was observed, with values of TPM, 303 μg/m^3^ in F1, 195 μg/m^3^ in F2, 235 μg/m^3^ in EU and 188 μg/m^3^ in OH. Temperatures averaged between 26 and 27°C, and relative humidity ranged between 39.8 and 43.5%. These environmental conditions and particulate matter can promote bacterial growth and their dispersion in the air, constituting a continuous risk of exposure to pathogens, mainly in indoor areas of the hospital. This study provides a framework for air monitoring, where the results of different samplers complement the detection of potential pathogens.

## Introduction

Airborne microorganisms within hospitals are implicated in nosocomial infections [1].

These cause diseases both in patients and in the health personnel, causing prolonged and high-risk hospital stays, which often leads to more significant patient mortality, mainly those belonging to vulnerable groups such as the older adults, children, and immunocompromised/suppressed patients. In these environments, respiratory and enteric bacteria and viruses are easily transmitted [2].

The spread of infection in hospitals occurs by contact, droplet, or airborne transmission of bioaerosols [3,4] causing cross-infections or outbreaks in hospitals. The transmission by infectious droplets larger than 5 μm is usually released from patients, while the transmission from droplet nuclei (< 5 μm) occurs in the airborne hospital environment. These can be present for long periods and become part of the indoor air microbiota [3,5]. Both types of infectious droplets can remain in the environment depending on the environmental conditions (e.g., humidity) [6–8]. Therefore, there is a high risk of spreading infectious diseases inside hospitals, so several precautionary measures, such as filtration and air renewal systems, have been recommended, mainly in critical areas (operating rooms, transplant rooms, and intensive care units). The measure of hospital-associated infection has been found not only to indicate safety for patients but also to show the overall quality of hygiene care provided in hospitals [9–12].

The severity and effect on patients exposed to various groups of bacteria present in hospital environments will depend mainly on the diversity of factors such as the type of bacterial community, its concentration in the environment, its toxicity and exposure time, and the inherent immune response of each host [13], as well as parameters or environmental factors characteristic of intramural environments.

Among the main environmental factors involved in the development and transmission of aerosols in the indoor environment of the hospital are temperature, relative humidity, ventilation and airflow, the presence and concentration of aero particles and their size, which directly influences both the airborne microbial load and its survival, as well as airborne microbial load and the health and well-being of patients and health workers [1]. Studies worldwide have agreed and recommended that the optimal environmental conditions in these interiors to prevent the proliferation and dispersion of microorganisms will have temperatures between 16–25°C and humidity of 40%–60% [14]. Likewise, the particles present in the air provide a general measure of the quality of hospital air and are related to sources and activities that occur inside or with the conditions of the exterior and the ventilation systems of the building [1,14,15].

Among the pathogens related to nosocomial infections the most frequent and serious infections are related to *Acinetobacter baumannii*, *Pseudomonas aeruginosa*, *Stutzerimonas stutzeri*, *Stenotrophomonas maltophilia*, and *Ochrobactrum anthropi*, which are associated with higher mortality rates [12,15–18].

Health institutions are committed to providing assistance and support to patients, so their priority should be to ensure a clean environment with high hygienic quality, mainly in critical areas of the hospital, for which it is crucial to monitor and evaluate the quality of the environment. Evaluating the biological quality of the air in these nosocomial environments allows us to determine the presence of pathogenic microbiota, which will lead to the reduction and prevent intrahospital infections and the implementation of epidemiological surveillance strategies and systems for the control of infections caused by airborne microbiota present in the environment of these health institutions. Therefore, the objective of this study was to evaluate and determine the composition of the bacterial community present in the air of a hospital environment using a metagenomic and proteomic approach. Two types of aerobiological samplers were used to assess how changes in bacterial community composition might be influenced by factors such as temperature, humidity, and airborne particulate matter.

## Methods

### Study area

**Fig 1:**
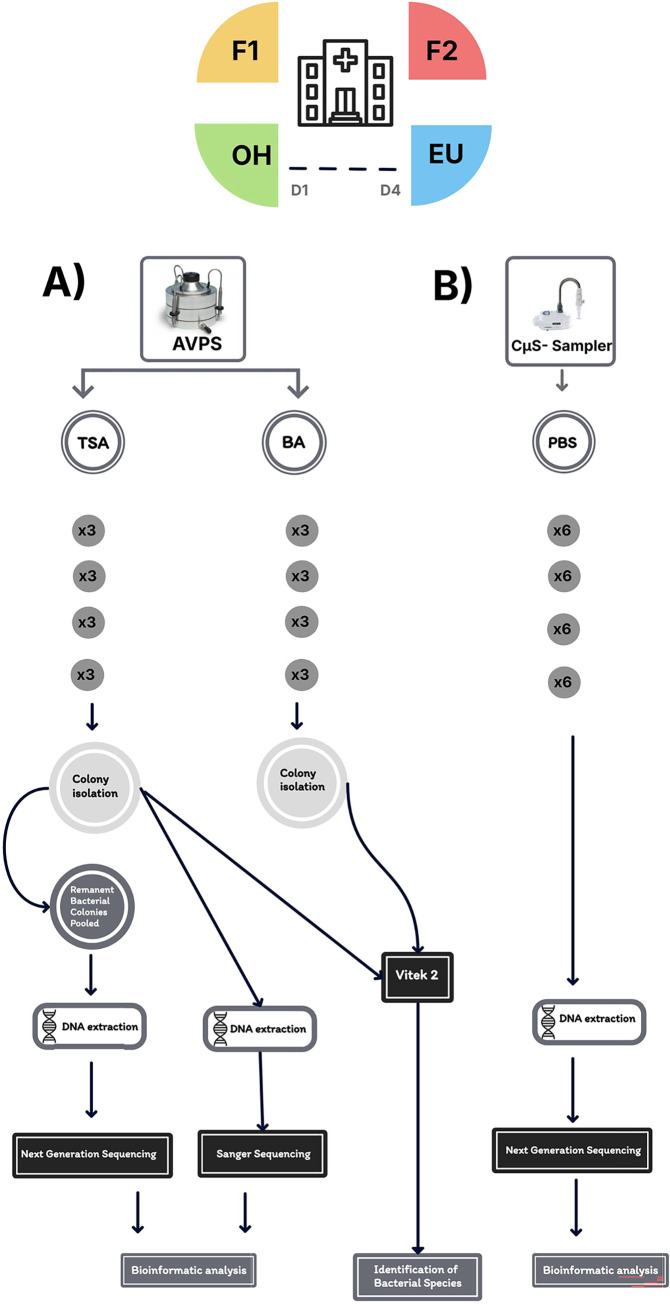
Methodological process for sampling airborne bacteria inside and outside the hospital using the samplers A) AVPS and B) CμS-Sampler. (A) shows the Trypticase Soy Agar (TSA) and 5% Sheep Blood Agar (BA) culture media used for the collection of airborne bacteria, as well as the subsequent steps for their detection, either directly from the colonies developed and identified by the Vitek systems or by Sanger Sequencing or from the pool of remaining bacteria through their identification by Next Generation Sequencing (NGS). (B) illustrates the use of PBS solution for collecting bacteria from the air, DNA extraction, and identification of bacteria by NGS. It also shows the number of replicates, sampling days, and monitored areas.

The study was conducted at a hospital within the Mexico City Health System, located in the southern part of Mexico City (19°17′17″N 99°09′23″W). This hospital includes departments such as internal medicine, highly specialized surgery, endocrinology, gastroenterology, hematology-oncology, infectious disease, nephrology and mineral metabolism, geriatric rheumatology, and cardio-pneumology. Sampling was performed in the mornings (between 8:00 AM and 1:00 PM), when the hospital has the highest influx activity from doctors, nurses, cleaning and kitchen staff, patients, and some family members. Sampling was carried out as follows: in the Emergency Unit (EU), near the nursing station, and in the main corridor of Floor 1 (F1) and Floor 2 (F2). These floors house patients with various pathologies: some receive medical treatment; others are awaiting surgery or are in the postoperative stage. In addition, sampling was carried out outside the hospital (OH), specifically at the main entrance. Air sampling was performed over three consecutive days, except in the EU, where it was limited to two days due to hospital restrictions (June 14 to 16, 2022, and from June 21 to 23, 2022) (Fig 1). The AVPS sampler collected air samples using two types of culture media (TSA and BA). Sampling was carried out in triplicate on F1 and F2, at EU and as well as outside of the hospital. Sixty-six samples were obtained, corresponding to 33 in TSA and 33 in BA. In addition, 11 samples were taken in PBS per zone and day with the CμS-Sampler, resulting in 88 samples inside the hospital and 33 outside. The hospital has 167 beds, distributed as follows: 60 in F1, 60 in F2, and 20 in the EU [19].

### Aerobiological samplers

1. Andersen One-Stage Viable Particle Sampler (AVPS) (Thermo Fisher Scientific, USA). Airborne bacteria were collected using the AVPS, with a flow rate of 28.3 L/min. Sampling was performed for 15 minutes by triplicate in each hospital area as well as outside. The culture media used for sampling included Trypticase Soy Agar (TSA) and 5% Sheep Blood Agar (BA). After sampling, the Petri dishes were transported to the laboratory and incubated at 37°C for 24 hours (Fig 1A).
2. Coriolis μ sampler (CμS-Sampler) (Bertin Technologies, St-Berthely, France). The CμS-Sampler collects airborne bacteria with a flow rate of 250 L/min into a conical tube containing 10 ml of phosphate-buffered saline solution (PBS) (Thermo Fisher Scientific). Each sampling was conducted for 10 minutes with six replicates per area. All samples were transported in a cold chamber to the laboratory and stored at -20°C until molecular processing (Fig 1B).

#### Quantification of airborne particulate matter and environmental parameters

In each monitored area, the concentration of suspended particles with diameters of 0.5 μm, 1.0 μm, 2.5 μm, 5.0 μm, 10.0 μm and total particulate matter (TPM), temperature, and relative humidity were measured daily using a Model 8306 particle counter (Particles Plus®). The airborne particulate matter and environmental parameters measurements were taken 1.5 meters above the ground. Sampling was conducted in cycles of 1 minute on, followed by 3 minutes off, during 2 hours.

#### Microbiological processing of the collected samples

In this study, our first challenge was to recover the greatest diversity of bacteria from the air, so we used two samplers with different technologies and different collection media (TSA, BA). The second challenge was obtaining sufficient DNA from air samples. To address this, we carried out individual colony isolation and also recovered material from the remaining colonies after isolation (Fig 1A).

Different molecular detection technologies were applied to more precisely identify the species level. Next-generation sequencing (NGS) allowed us to identify a broader range of bacteria (Fig 1A and 1B). On the other hand, the Vitek system and Sanger sequencing facilitated the identification at species level of some groups. (Fig 1A).

### AVPS samples

#### Process of samples obtained from bacteria collected with the AVPS (Fig 1A)

Airborne bacteria collected from the air on TSA and BA media were individually isolated for identification to species level. After colony isolation, the Petri dishes with TSA medium were not discarded, but 5 ml of PBS solution was added to each Petri dish by sweeping the surface of the culture medium with an L-shaped inoculation spatula. This material was poured into 15 ml Falcon tubes and kept at 4°C for further processing.

Isolated bacterial colonies were identified using Vitek 2 Compact or Vitek MS (MALDI-ToF). Samples that could not be identified using Vitek systems were identified using Sanger sequencing (Fig 1A).

### Description of methods used for the identification of bacteria by Vitek systems and Sanger Sequencing

1. Vitek 2 Compact and Vitek MS (MALDI-ToF): Identification with the Vitek MS was performed following the manufacturer’s instructions. For Vitek 2 Compact, colonies were suspended to a 0.5 MacFarland scale and tested using a Gram-negative identification card (BioMérieux, France). For Vitek MS, a colony was picked with the Vitek Pickme® and placed on a sample slide. After the sample dried, 1 μL of Vitek MS CHCA resin was added. Results were visualized using MyLa software (BioMérieux, France).
2. 16S rRNA Sanger Sequencing:
  DNA extraction from the isolated bacteria colonies were performed followed the Wizard Genomic DNA Purification Kit protocol (Promega Corporation, USA). Two nuclease-free water elutions were performed, each with a volume of 50 μL, to obtain a higher yield of DNA. The elutions were concentrated to 25 μL with a SpeedVac concentrator (DNA120 Savant, Thermo Scientific, USA). NanoDrop spectrophotometer (Thermo Scientific, USA) was used for quantification and purity determination of DNA. The results were also confirmed by gel electrophoresis using 0.8% agarose gel stained with SYBER Safe.
  PCR Amplification: The reaction mix included 5 μL of 10X Omega Biotek buffer (with 2 mM MgSO4), 1.2 μL of a dNTP mixture (0.2 mM each, Invitrogen®), 5 U of Biotek Taq DNA polymerase, 15 pmol of each T4 oligonucleotide, 29.2 μL of Sigma-Aldrich® ultrapure water, and 5 μL of DNA. Amplification was performed in a Veriti thermal cycler (Applied Biosystems, USA) with the following program: 1 minute at 95°C, 30 cycles of 30 seconds at 95°C, 15 seconds at 55°C, and 45 seconds at 68°C, followed by a final 5-minute extension at 68°C. The oligonucleotides used were 27F (AGAGTTTGATCMTGGCTCAG) and 801R (GGCGTGGACTTCCAGGGTATCT) (MM18, CLSI 2008). The resulting amplicon was 880 bp.
  Gel Electrophoresis and Purification: PCR products were separated by gel electrophoresis on a 1% agarose gel with SYBR Safe at 100 V for 45 minutes. Products were purified using the QIAquick PCR Purification Kit (Qiagen, Germany), and quantified with a NanoDrop (Thermo Fisher Scientific Inc, USA), and adjusted to 20 ng/μL.
  Sequencing: the BigDye Terminator v3.1 Cycle Sequencing Kit (Applied Biosystems, Thermo Fisher Scientific, USA) was used. Each reaction included 4 pmol of each oligonucleotide and 20 ng of the PCR product. The sequencing reaction was purified with the BigDye® Xterminator Purification Kit (Applied Biosystems, Thermo Fisher Scientific, USA), vortexed at 2000 rpm for 30 minutes, and centrifuged at 2000 rpm for 2 minutes. Sequencing was performed using the 3700xl DNA Analyzer (Applied Biosystems, USA) with POP-7 polymer (Thermo Scientific, USA).

On the other hand, DNA extraction from the samples poured into 15 ml Falcon tubes maintained at 4°C (remaining material from colonies collected in the TSA medium) was carried out. A Wizard Genomic DNA Purification Kit protocol (Promega Corporation, USA) was used for this. Two nuclease-free water elutions were performed, each with a volume of 50 μL, to obtain a higher yield of DNA. The elutions were concentrated at approximately 25 μL with a SpeedVac concentrator (DNA120 Savant, Thermo Scientific, USA).

#### CμS-Sampler samples

The PBS used for air sampling with the CμS-Sampler was filtered using Swinnex Filter Holders (25 mm) equipped with 0.22 μm sterile Millipore filters (Merk). The filters were stored at 4°C until they were processed for DNA extraction, purification, and subsequent massive sequencing using (Fig 1B).

DNA extraction from bacteria collected in PBS with CμS-Sampler. Six samples of PBS from the air of each evaluated area were processed to perform the metagenomic analysis (Fig 1B). The DNA extraction was performed using the ’Wizard Genomic DNA Purification Kit’ protocol (Promega Corporation, USA). Two nuclease-free water elutions were performed, each with a volume of 50 μL, to obtain a higher yield of DNA. The elutions were concentrated at a volume of approximately 25 μL with a SpeedVac concentrator (DNA120 Savant, Thermo Scientific, USA).

### Illumina sequencing

Illumina DNA sequencing of bacteria samples collected from the air with the AVPS and CμS-Sampler was carried out as follows: The metagbarcoding DNA was quantified using a Qubit fluorometer (Thermo Fisher Scientific, USA), and its purity was verified and sent to the National Institute of Genomic Medicine (INMEGEN) to construct and sequence 16S rRNA libraries. The variable regions, V3 and V4, of the bacterial 16S rRNA gene were amplified using 12.5 ng of DNA and the following primers: S-D-Bact-0341-b-S-17 (5′-CCTACGGGNGGCWGCAG-3′) and S-D-Bact-0785 -a-A-21 (5′-GACTACHVGGGTATCTAATCC-3′) [20], and sequenced according to the protocol for sequencing on the Illumina NextSeq 1000/2000 using a 600-cycle kit in a P1 flowcell. The bioinformatics analysis was performed at the University Mass Sequencing and Bioinformatics Unit (UUSMB, IBT). The sequencing raw data and sample information are publicly available in the NCBI BioProject database under the PRJNA1085383 ID.

### Bioinformatic analysis

#### Sanger sequencing

The complementary reverse sequence was created to keep the 5’ and 3’ ends correctly oriented, using the SeqKit v.0.9.1 toolkit [21]. Subsequently, the merger application of the EMBOSS v.6.6.0 [22] bioinformatics tool was used to reconstruct each amplicon using the forward and reverse complementary sequences of each sample. This tool uses a global alignment algorithm (Needleman-Wunsch) to align the sequences (Shen et al., 2016; Rice et al., 2000. A BLAST search was performed at the NCBI site (https://blast.ncbi.nlm.nih.gov/Blast.cgi) for each of the reconstructed amplicons using default parameters.

#### NGS analysis

The assembly reconstruction of the sequenced 16S rRNA variable regions fragments was performed using the Flash software [23]. From this process, consensus sequence files were generated for each sample, which were then input into the Parallel-META v2.4.1 program [24] for annotation and taxonomic classification of the generated sequences, utilizing a specific database for 16S rRNA. Finally, Perl and R scripts developed at UUSMB, IBT were used to generate tables by taxonomic level, diversity indices, stacked bar graphs, and Krona graphics. Rarefaction curves were generated using the Ranacapa R package [25].

## Results

### Sequencing data

The DNA concentration and purity obtained from the samples were suitable for sequencing. All the samples presented a base quality equal to or greater than Q20 (99% of accuracy) and no adapters were detected (The quality control report for each sample is included in the supplementary material in HTML format S1 Text).

The characteristics of the metagenomic analysis are shown in Table 1. Samples obtained with the AVPS had total maximum sequenced reads within the hospital of 2893471 (raw sequences) obtained from F2, followed by F1and the EU. On the other hand, with the CųS- Sampler, the maximum sequenced reads within the hospital were 3611425 obtained in F1, followed by those of F2 (zones with greater activity) and EU, which were validated after quality filtering and obtaining post-fusion reads for subsequent analysis. Outside the hospital (OH), the maximum readings were 633057 obtained with the CμS-Sampler (Table 1). Regarding sequence identity, the total taxonomic annotations at the genera level were obtained with the CųS-Sampler, mainly in F1 (1057) and F2 (984) (Table 1). The present study obtained more sequenced readings with the CμS-Sampler.

**Table 1:** Descriptive characteristics of the metagenomic analysis (16S).

Characteristic	16S							
	AVPS				CųS			
	OH	F 1	F 2	EU	OH	F 1	F 2	EU
Raw reads	3306752	2697901	2893471	2236483	633057	3611425	1227003	63854
Post-merging reads	3300838	2692438	2887841	2232283	631920	3605383	1224419	63655
Total taxonomy annotations at genus level	904	630	438	575	691	1057	984	644
Unclassified taxa at genus level	12	12	6	7	9	13	13	10
Classified taxa at genus level	892	618	432	568	682	1044	971	634
Unique Known Genera	178	75	19	59	37	163	220	27

**Fig 2:**
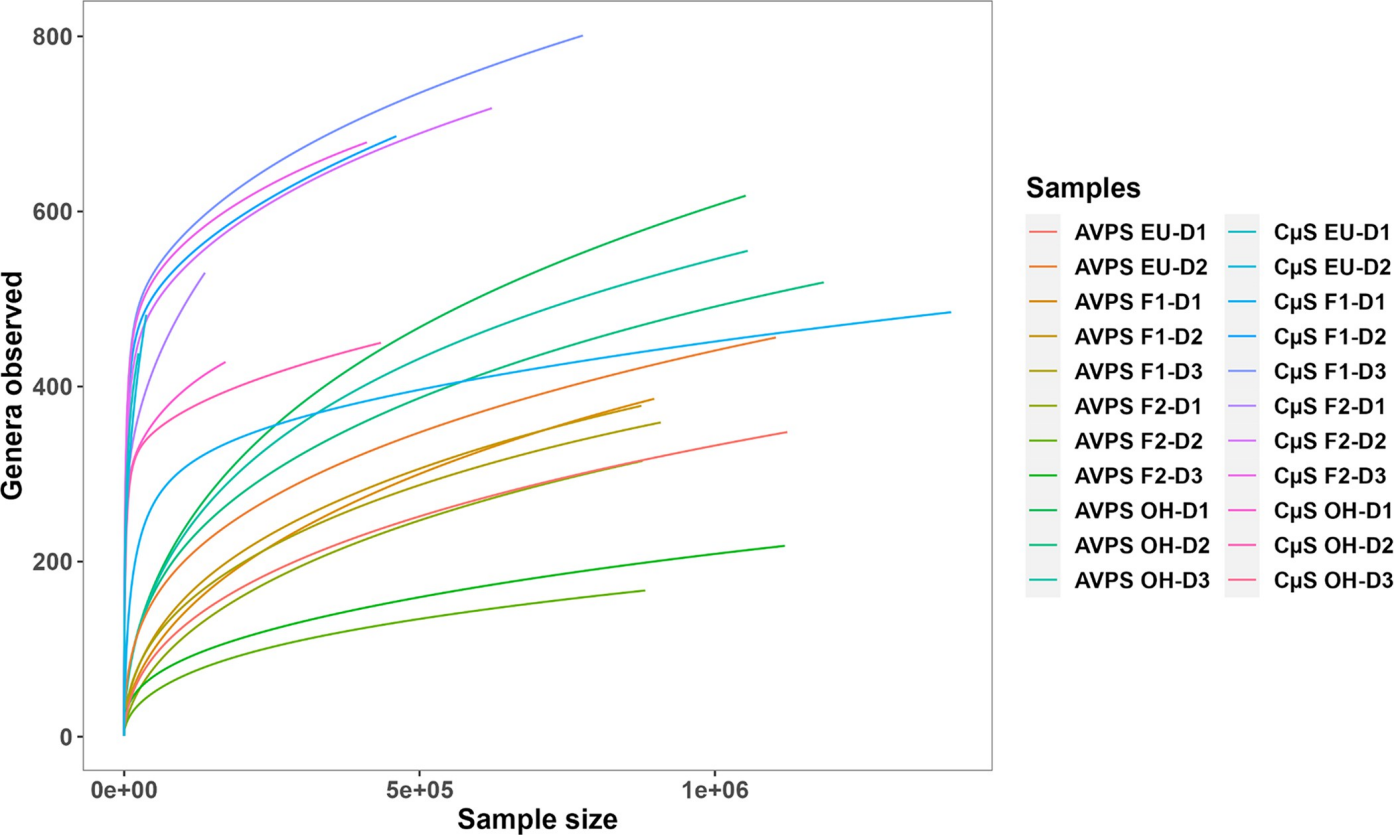
Rarefaction curves of the bacterial genera were observed for each sampling area. The legends correspond to the samples obtained with the AVPS (lower richness and higher density of genera), and those on the right side indicate the bacteria samples collected with the CųS-Sampler (higher richness of genera and lower density) in each monitored hospital area.

The bioinformatics analysis of the 16S rRNA indicated through the rarefaction curves that the sampling of the bioaerosols and the detection of DNA allowed reaching the correct sampling force to carry out the molecular analysis (Fig 2). The Rarefaction curves estimate the expected number of species for a given sample size based on a hypergeometric distribution. This figure shows that most of the samples are close to plateau, demonstrating that the sequencing depth obtained is sufficient for our analysis.

#### Bacterial community composition

**Fig 3:**
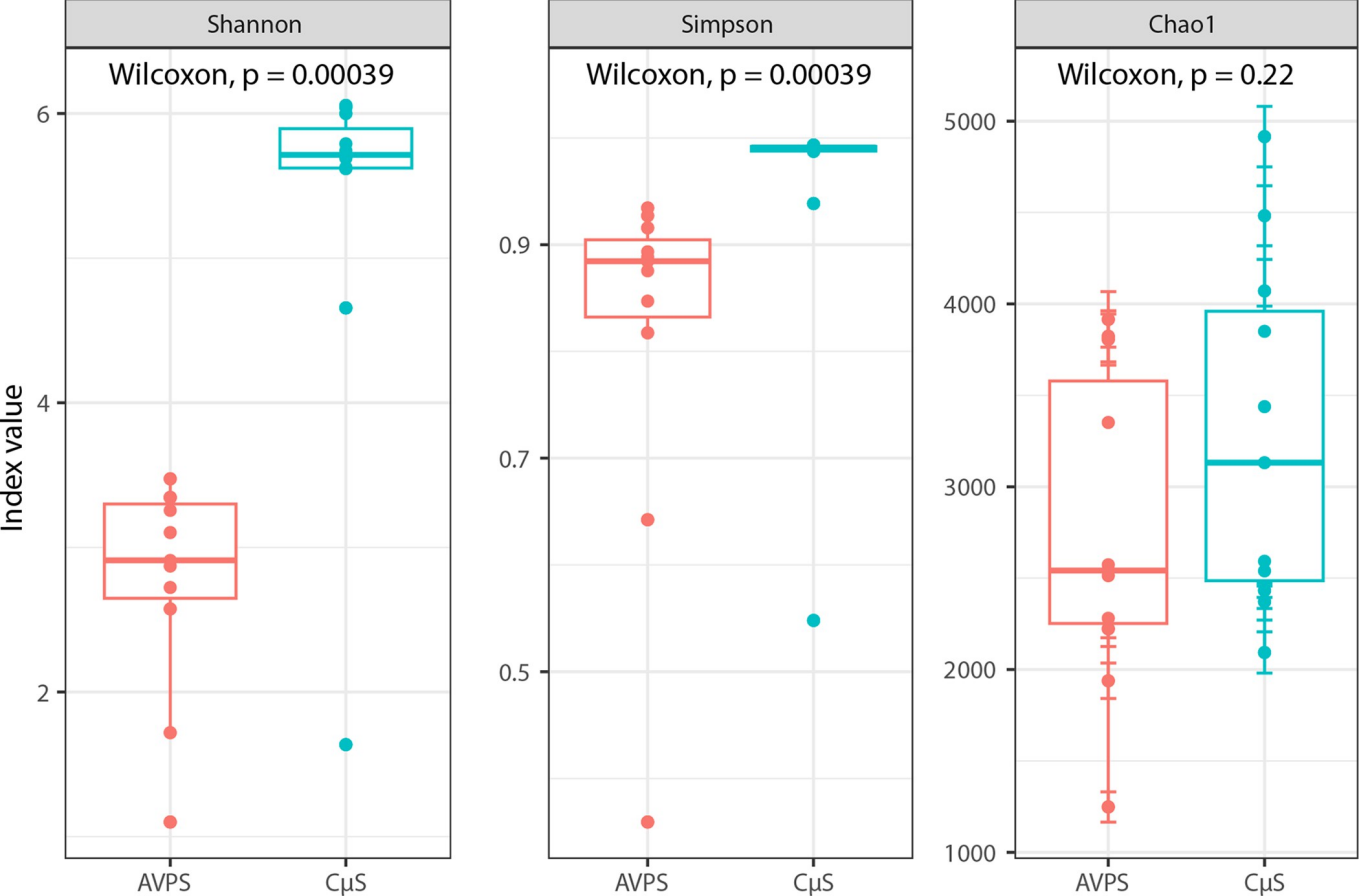
Alpha indices (Shannon and Simpson and Chao) from the total samples collected with the AVPS and the CųS-Sampler samplers, and the statistical values obtained with the Wilcoxon test to determine differences between both air samplers.

To better describe and compare communities, we used metrics adapted to metagenomics. The Chao, Shannon, and Simpson indices are alpha (α) diversity metrics that assess local community diversity. The Chao 1 index is useful for analyzing datasets biased toward low abundances. The Simpson index calculates diversity by considering the number of taxa and their abundance, giving more weight to common or dominant species so that rare species have a lower impact on the diversity measure. On the other hand, the Shannon index summarizes population diversity, assuming that all species are represented in the sample and that samples are taken randomly. The Shannon index increases as both community richness and evenness increase. We used all three indices (Chao, Shannon, and Simpson) to demonstrate that both samplers were able to collect airborne microorganisms. A Wilcoxon signed rank test was performed to assess the difference in sample median; The Chao index did not show a clear difference (p = 0.22) in the abundance of microbiota collected with both samplers. However, the Shannon and Simpson indices showed statistically significant differences (p = 0.00039) between the AVPS and CųS-Sampler samplers, and we can conclude that the CųS-Sampler can capture a greater bacterial diversity and richness (Fig 3).

**Fig 4:**
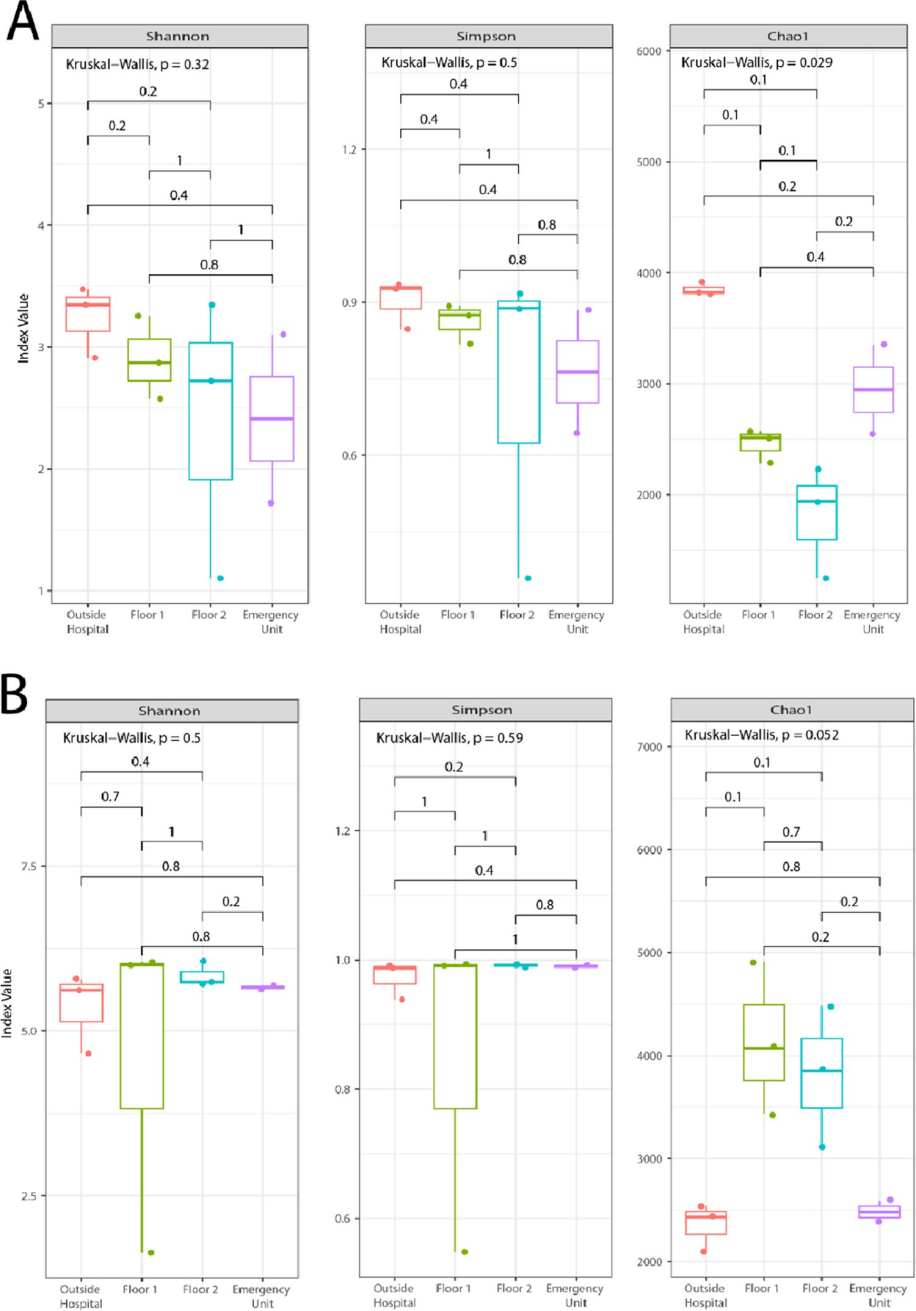
Alpha indices (Shannon and Simpson) and Chao index from the samples collected with the (A) AVPS and the (B) CųS-Sampler samplers in the different sampled areas of the hospital and outside; and the statistical values obtained with the Kruskal Wallis test to determine differences between the sampled areas.

Samples collected with the AVPS (Fig 4A) and CųS-Sampler (Fig 4B) samplers were compared for each index. The nonparametric Kruskal-Wallis test was used to determine possible significant differences between the sampled areas of the hospital (OH, F1, F2 and EU) for each diversity index.

According to the Shannon and Simpson diversity indices and the Chao index, for the bacterial diversity data collected with the AVPS, no significant differences were found (Kruskal-Wallis test) between the sampled areas of the hospital (p = 0.32, p = 0.5, p = 0.29, respectively) not even concerning the outside area. However, it can be observed that the collected samples that presented the most notable richness of bacterial species were recorded outside the hospital, the diversity between F1 and F2 was similar, while the EU recorded the lowest diversity (Fig 4A).

On the other hand, for the CųS-Sampler, Shannon and Simpson indices showed no significant differences (Kruskal-Wallis test, p = 0.5, p = 0.59, respectively) between the groups, i.e., between the sampled areas of the hospital. The Chao index (with a significance level of 0.10) shows differences between the groups, observing that the greatest diversity of bacteria was recorded on F1, followed by F2 (Fig 4B).

The KRONA graph shows all phyla and genera recorded with both samplers in each sampled area (S1 Fig):

#### Phyla present in the air sampled from the different areas of the hospital and outside

In general, the relative abundance of airborne bacteria genera with the CųS-Sampler was 89.5% while the AVPS 74.4% (S1 Fig). The relative abundance is based on the total read count obtained by the sequencing.

The Phylum quantification was obtained from the taxonomy annotation which is provided by Parallel-Meta version 2.4.1 at Phylum, class, order, family and genus levels. The lower rank (genus in this case) was calculated first with a 97% cutoff value and from the full taxonomic ranking each relative abundance was calculated by the program.

**Fig 5:**
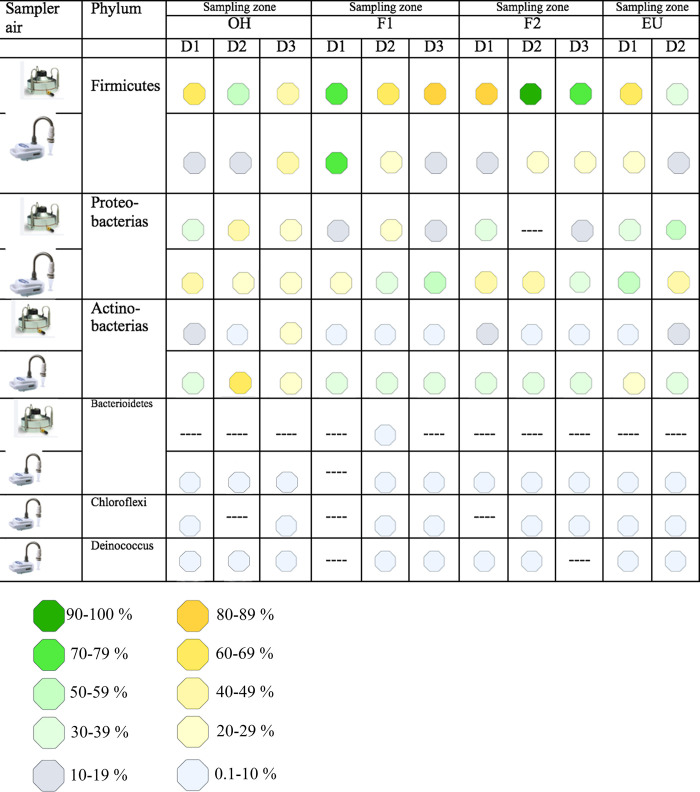
Percentages of the main phyla of bacteria registered in the AVPS and the CųS in each hospital area and obtained from KRONA graph (S1 Fig).

The results showed that the main relative abundance were collected with the AVPS and corresponded to Firmicutes, followed by Proteobacteria and Actinobacteria. Firmicutes were present in 76 to 97% of F2, 62 to 83% in F1, 35 to 68% in EU, and 48 to 60% at out-of-hospital. In contrast, Proteobacteria presented low proportions in all areas, registering values between 31 and 55% in the EU and 3 to 15% in F2. Finally, Actinobacteria registered from 0.8 to 10% in the EU and from 2 to 24% abroad. The percentage values were obtained from the bioinformatics analysis performed on the sequencing with Illumina NextSeq 1000/2000 using the KRONA software (Figs 5 and S1).

A considerable number of bacterial phyla were collected with CųS-Sampler (Fig 5); the main ones are Firmicutes, Proteobacteria, Actinobacteria, Bacteroidetes, Chloroflexi and Deinococcus, among others. Firmicutes were present from 12 to 72% in F1; Proteobacteria recorded from 25 to 51% in F2; Actinobacteria from 28 to 65% at OH, followed by 33 to 39% in EU. The rest of the phyla are present with relative abundance >1%, except for Chloroflexi and Deinococcus (Fig 5).

In summary, with both samplers, the predominant bacterial phyla were Firmicutes, Proteobacteria, and Actinobacteria; and the areas with the highest percentage of bacteria were F2 and F1 for Firmicutes; EU and F2 for Proteobacteria, and at OH and F2 for Actinobacteria.

#### Main genera of identified bacteria in the air in the different areas of the hospital and outside

**Fig 6:**
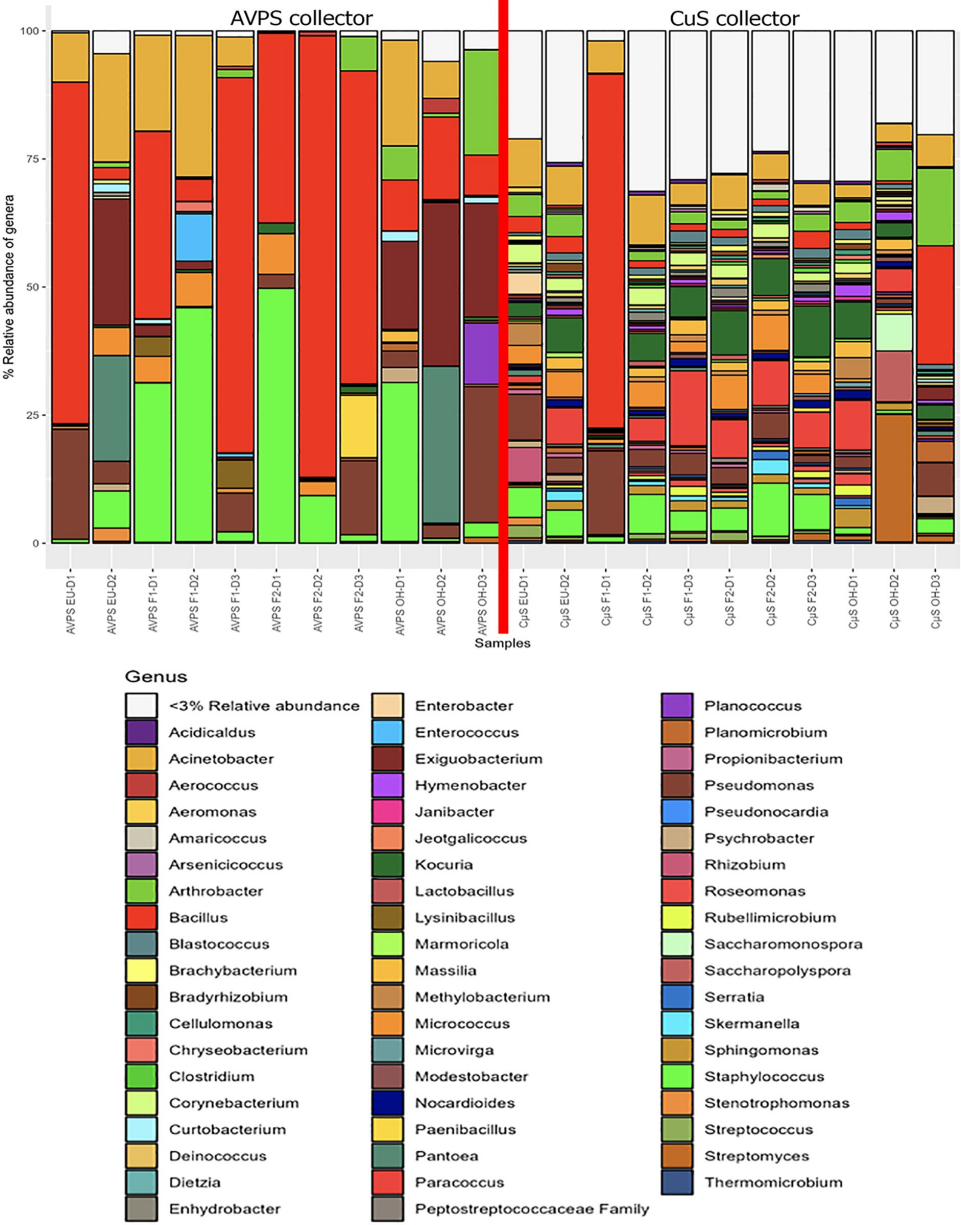
Relative abundance bacterial genera collected with both AVPS and CųS-Sampler for each sampled area. AVPS sample suffix = Samples obtained with the AVPS collector. CųS-Sampler sample suffix = Samples obtained with the CuS-Sampler collector.

Regarding the diversity of bacteria identified, Fig 6 show the relative abundance of the main genera collected with AVPS and CųS-Sampler for each sampled area (EU, F 1, F 2 and at OH).

**Fig 7:**
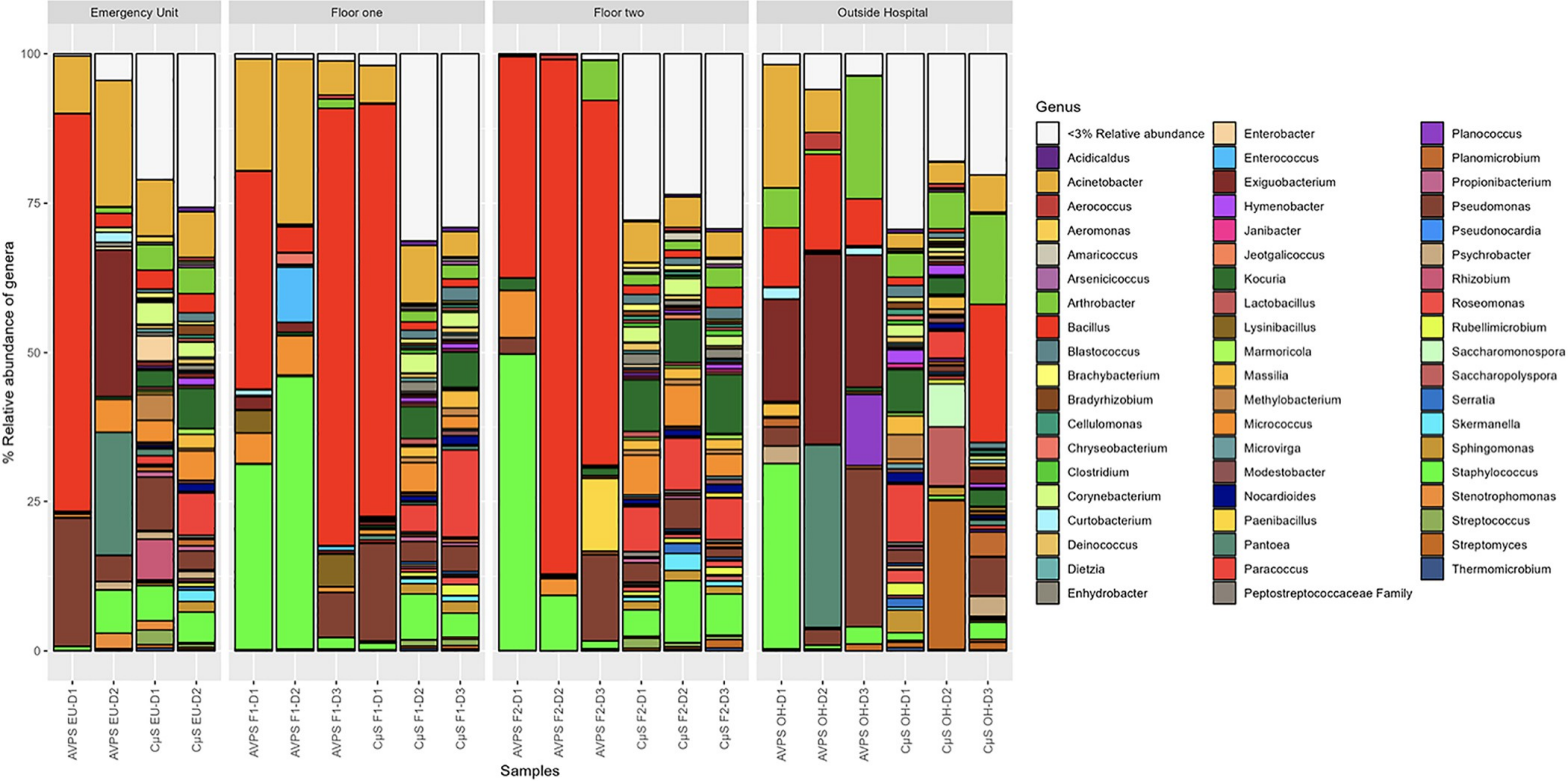
Relative abundance of the main genera of bacteria identified in each area sampled inside and outside the hospital. AVPS sample suffix = Samples obtained with the AVPS collector. CuS-Sampler sample suffix = Samples obtained with the CuS-Sampler collector.

**Fig 8:**
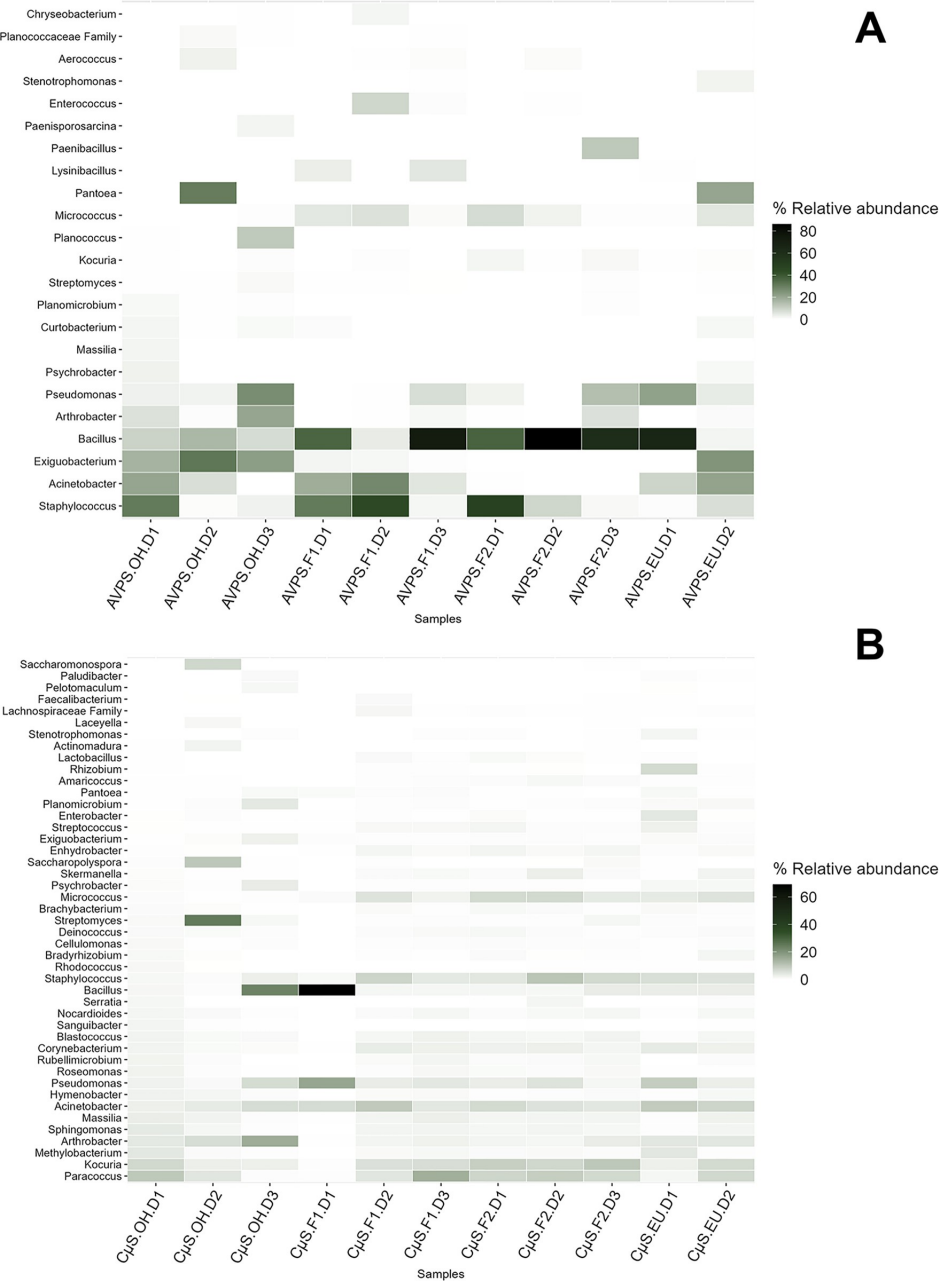
Heat map showing the relative abundance of the main genera of fungi collected with the AVPS (A) and with the CųS-Sampler (B) in the different sampling areas inside and outside the hospital. The AVPS collected *Bacillus* in great abundance on floors one, two and the emergency unit, while with CyS, *Bacillus* was only detected at the outside of the hospital and on floor one.

Among the most abundant genera collected with the AVPS are *Bacillus*, *Staphylococcus*, *Acinetobacter*, *Exiguobacterium*, and *Arthrobacter*, among others, while in the samples collected with the CųS-Sampler, a greater diversity was determined, standing out: *Bacillus*, *Acinetobacter*, *Kocuria*, *Staphylococcus*, *Streptococcus*, *Streptomyces*, *Corynebacterium*, *Serratia*, *Clostridium*, *Massilia*, among others.When analyzing the main genera of air bacteria determined for each sampling area, it was found that at out of the hospital (OH), the predominant bacteria collected with the AVPS were *Bacillus*, *Staphylococus*, *Acinetobacter*, *Exiguobacterium*, *Pseudomonas*, *Arthrobacter*, *Pantoea* (Enterobacteriaceae), *Planococcus* and with the CųS-Sampler, a greater diversity of bacteria was identified; Among the main ones were *Acinetobacter*, *Arthrobacter*, *Bacillus*, *Massilia*, *Sacchromonospora*, *Streptomyces*, *Nocardioides*, *Paracoccus*, *Kocuria*, *Clostridium*, among others. On F1, the main bacteria detected with AVPS were Bacillus, Staphylococcus and *Acinetobacter*, followed by *Micrococcus*, *Enterococcus*, On F2, *Bacillus*, *Staphylococcus*, *Pseudomonas*, *Micrococcus*, *Paenibacillus*, *Pseudomonas Kocuria*, and at EU were *Acinetobacter*, *Bacillus*, *Enterococcus*, *Exiguobacterium*, *Staphylococcus*, *Pseudomonas*, *Pantoea*, *Stenotrophomonas*, *Micrococcus;* and at OH, *Acinetobacter*, *Arthrobacter*, *Bacillus*, *Exiguobacterium*, *Pantoea*, *Planococcus*, *Pseudomonas*, *Staphylococcus*. CųS-Sampler the main bacteria detected on F1, F2 and EU were *Staphylococcus Acinetobacter*, *Bacillus*, *Kocuria*, *Micrococcus*, *Paracoccus* and *Pseudomonas*, among many others; and outside the hospital, there was a predominance of *Acinetobacter*, *Arthrobacter*, *Bacillus*, *Pseudomonas*, *Staphylococcus*, *Streptomyces*, *Pantoea*, *and Planococcus*, among others (Figs 7, 8A, 8B and S1).

It is interesting to mention that microorganisms belonging to the group of Archaea were also present in the air inside and outside the hospital. However, in very low percentages (0.001 to 0.3%), the main archaea registered are part of the Euryachaeota group and among them were Methanomicrobia, Thermoplasmatales, Methanobacteriales, Methanococcales, Methanocellales and Halobacteriales (S1 Fig).

**Fig 9:**
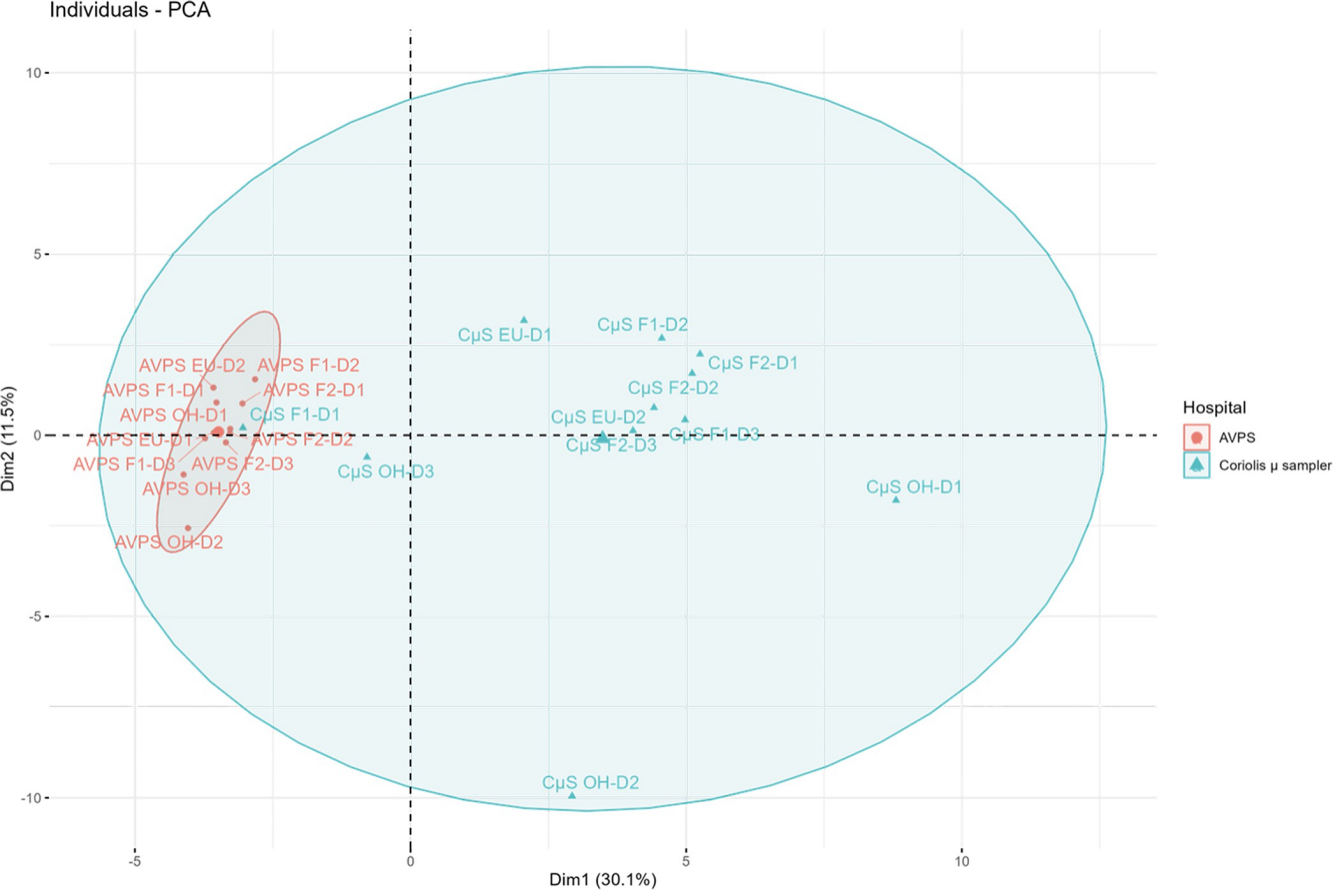
Principal component graph shows the grouping of the main genera of bacteria collected with the AVPS and the Coriolis μ Sampler (CųS-Sampler) in the different sampled areas.

Fig 9 shows the principal components graph (PCG) that groups the genera of bacteria collected with the AVPS and the CųS-Sampler in the different sampled areas. It can be observed that the bacteria collected with the CųS-Sampler present a greater diversity of genera compared to those collected with the AVPS, whose types of genera are concentrated in only two quadrants. In comparison, those collected with the CųS-Sampler cover the four quadrants of PCG.

**Fig 10:**
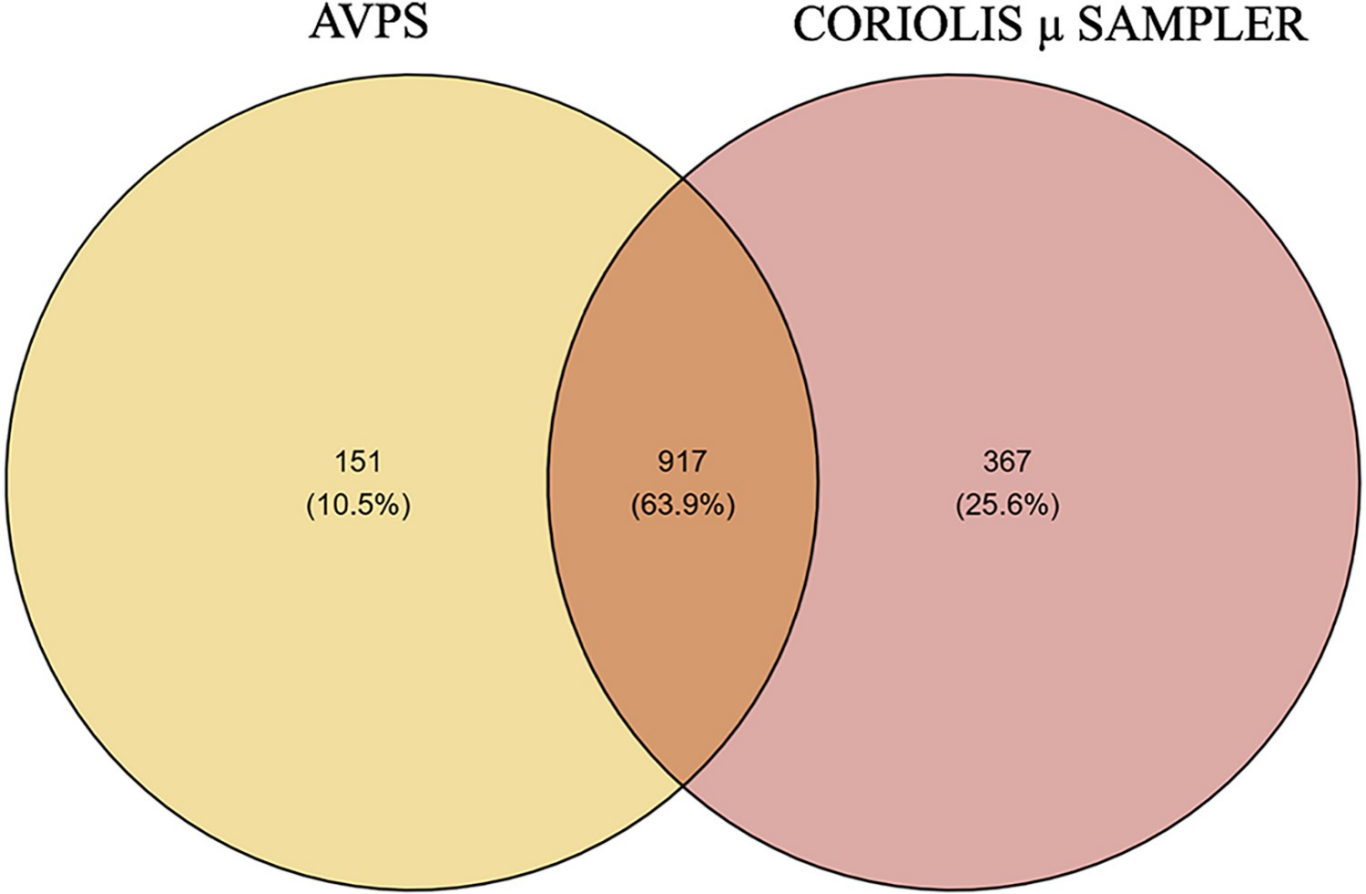
Venn diagram with the percentage of genera of bacteria registered with AVPS and Coriolis μ sampler (CųS-Sampler). A higher percentage of bacterial genera were collected with both samplers (63.9%).

The CųS-Sampler determined a 25.6% relative abundance of bacteria not collected with the AVPS. With the AVPS, only 10.5% contained bacteria different from those obtained with the CųS-Sampler (Fig 10).

#### Main species of identified bacteria in the air in the different areas of the hospital and at outside

**Fig 11:**
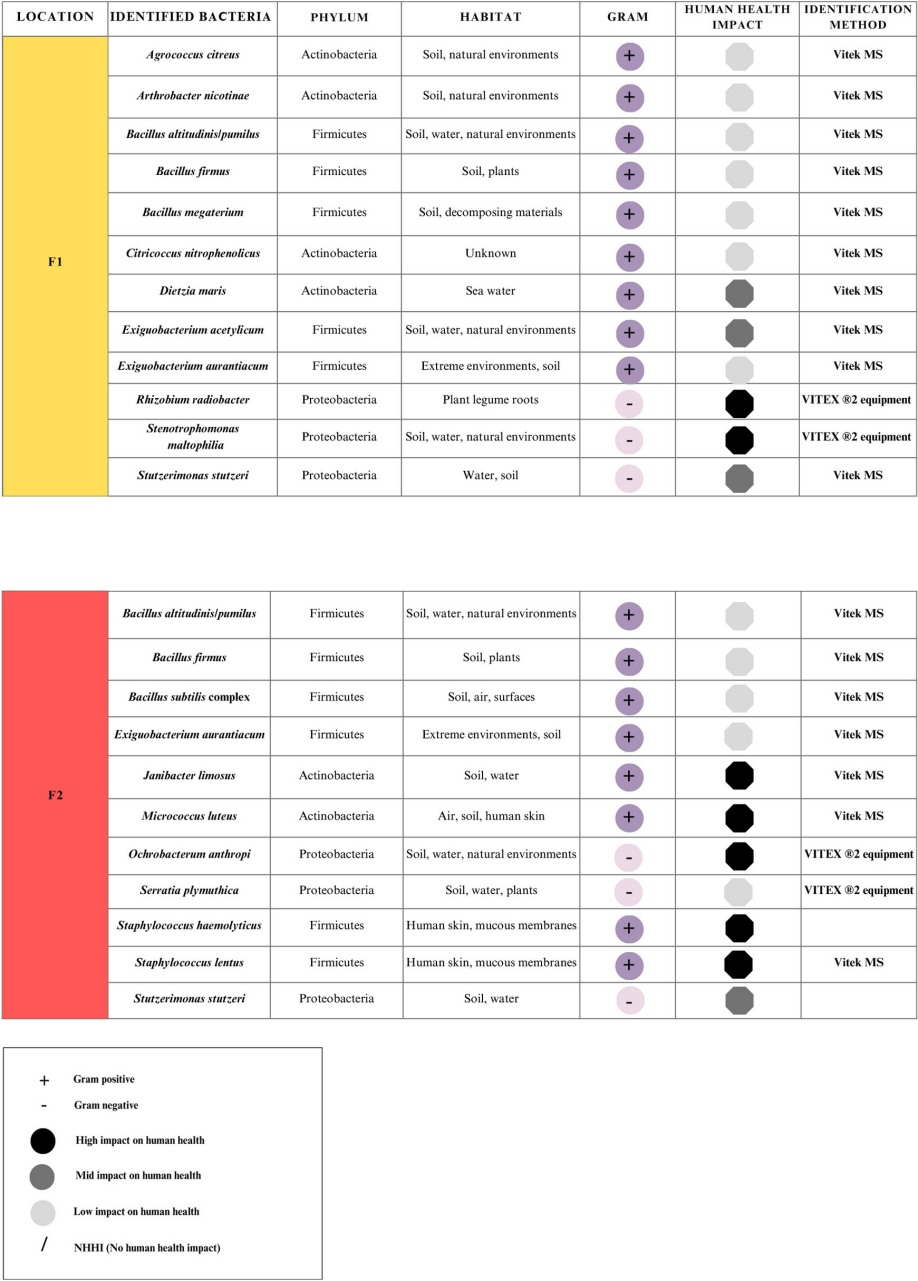
Species of bacteria collected in the TSA and BA medium with the AVPS sampler on Floor 1 and Floor 2 from the hospital.

**Fig 12:**
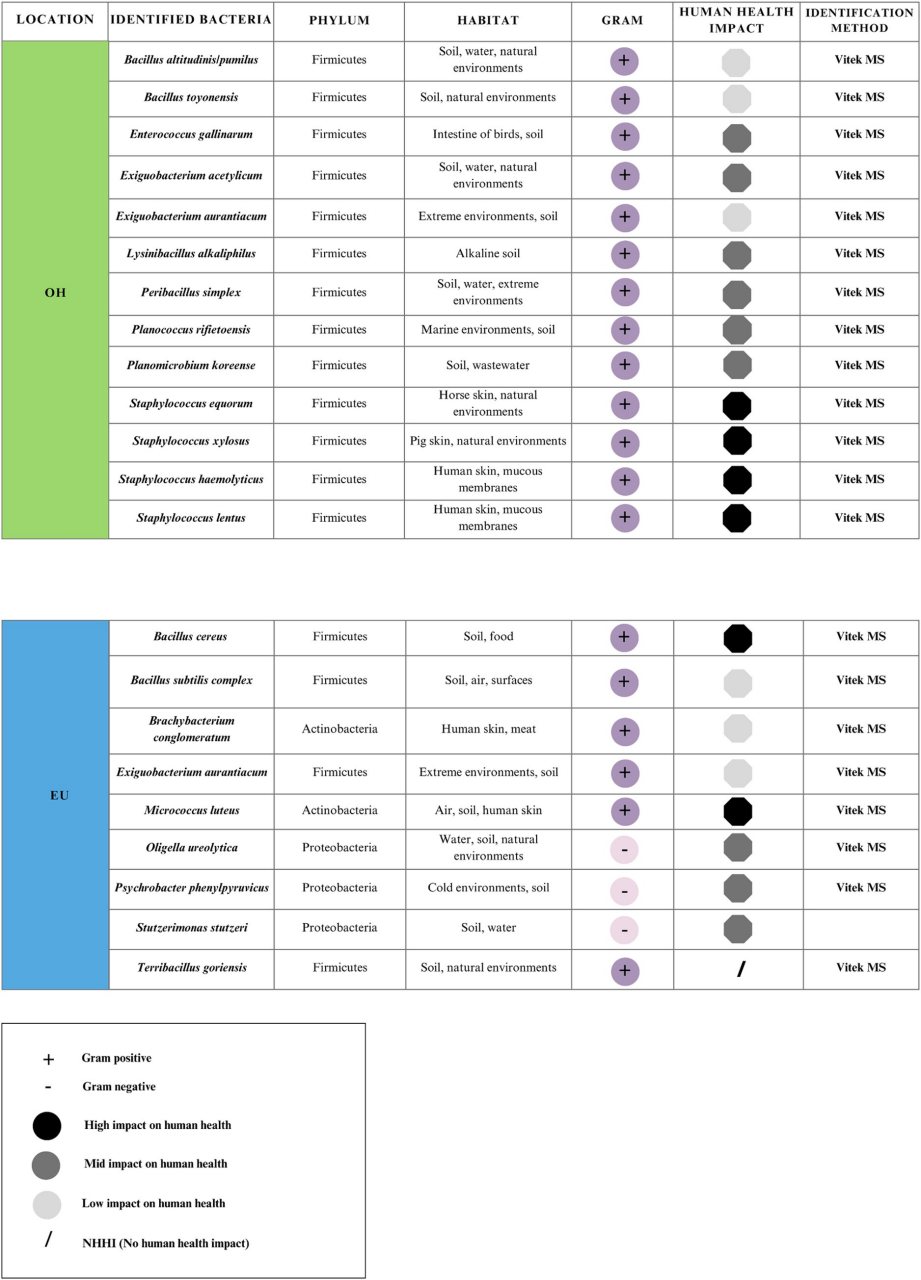
Species of bacteria collected in the TSA and BA medium with the AVPS sampler in the Emergency Unit and at outside of the hospital.

A total of 32 species were identified, 11 on floor one, 11 on floor two, 9 in the emergency unit, and 14 at outside the hospital (outside the hospital entrance). Figs 11 and 12 present a list of the identified bacterial species by Vitex system or by Sanger Sequencing, indicating mainly the phylum to which they belong, the habitat where they are most frequently found in the environment and their impact on human health.

**Fig 13:**
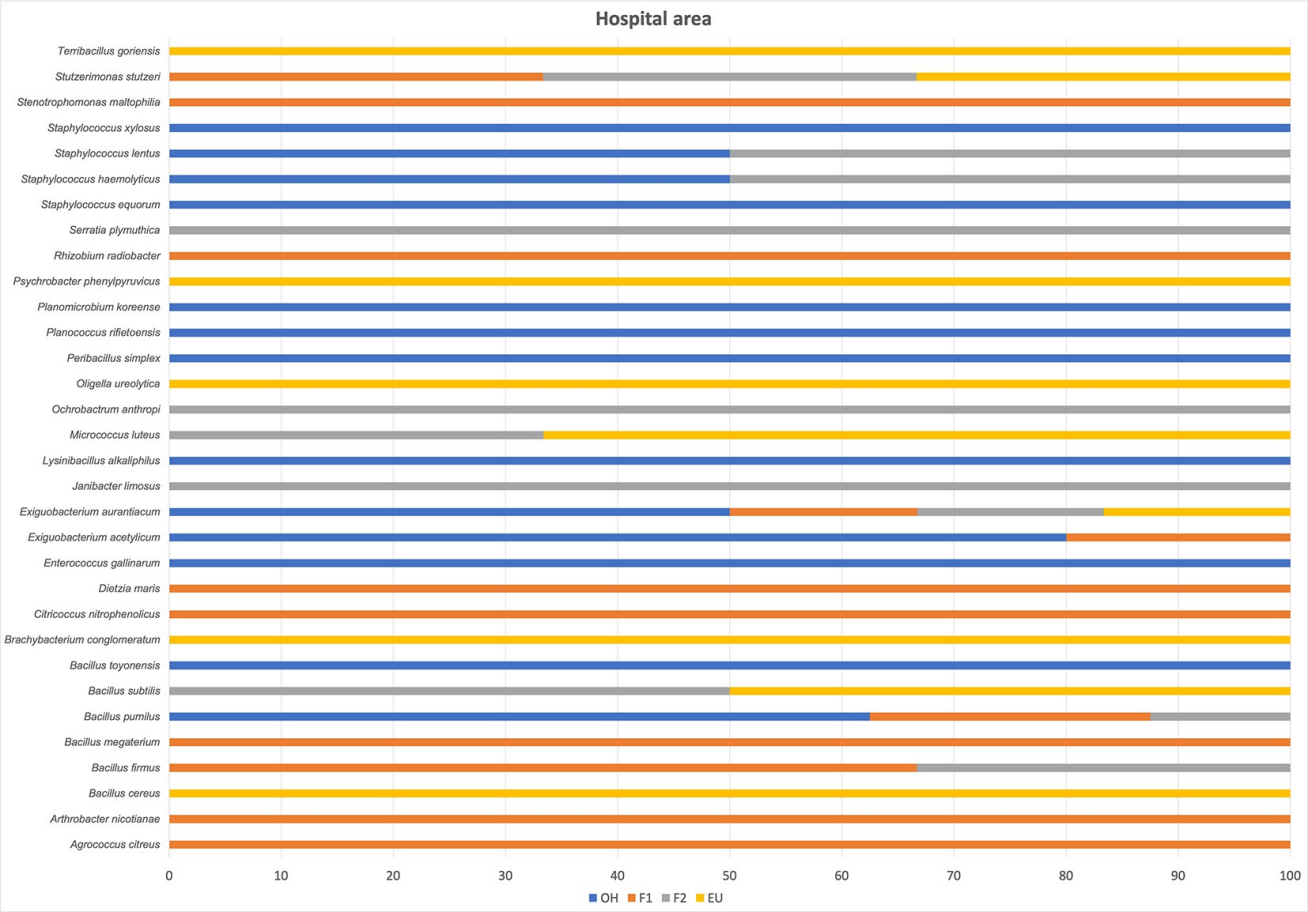
Airborne bacteria species isolated from different areas of the hospital. The x-axis indicates the specie’s relative abundance.

Fig 13 shows the relative abundance of airborne bacterial species at outside and in the hospital.

#### Microclimatic conditions and suspended particles in airborne indoor and outdoor hospital

Table 2 shows the microclimatic conditions and air quality inside and outside the hospital during bioaerosols sampling. The average temperature in F1 and F2 fluctuated between 26 and 27°C, while in the EU and at OH, an average of 24°C was recorded. The highest relative humidity values were found in these last two areas (41.9 and 47.3, respectively). The highest relative humidity values were found in these last two areas (41.9 and 47.3, respectively) with an average of 48%.

**Table 2:** Airborne particulate matter, temperature and relative humidity recorded with the airborne particle counter (model 8306, Particles Plus® brand) at the outside and inside air of the hospital.

Sampling Zone		Temperature °C	Relative Humidity %	PM* 1.00 ųg/m^3^	PM* 1.00 ųg/m^3^	PM* 2.50 ųg/m^3^	PM* 5.00 ųg/m^3^	PM* 10.00 ųg/m^3^	TPM** ų g/m^3^
**OH**									
	Mean	24.8	41.9	8.7	11.4	17.2	35.0	66.8	188.4
	Interval	22–29.4	34–49	0.76–13.6	1.4–18.7	4.9–28.1	13.5–72.8	24.8–208	47.1–753.0˚
								
	Standard deviation	1.66	5.16	3.00	3.90	5.37	13.08	33.22	118.70
**F1**									
	Mean	26.0	43.5	5.9	9.4	24.1	58.6	101.6	303.3
	Interval	20.4–27.0	37–58	1.55–18.3	4.42–23.9	9.2–73.1	24.1–244	43.8–553	129.6–1642
								
	Standard deviation	1.35	4.20	4.93	5.56	10.16	28.52	53.11	174.98
**F2**									
	Mean	27.00	39.8	8.1	12.1	22.0	44.1	73.3	195.0
	Interval	24.4–27.9	37–43	2.2–22.3	3.8–31.6	7.2–44.9	14.02–73	25.7–130	79.6–653.0
								
	Standard deviation	.91	1.25	7.20	9.67	11.31	16.58	22.02	75.31
**EU**									
	Mean	24.00	47.3	1.8	3.3	9.9	31.4	6.8	235.80
	Interval	20.1–24.9	44–56	0.79–4.1	2.08–6.35	6.32–19.2	15.3–64.4	30.16–135	80.02–591.1
								
	Standard deviation	1.14	2.7	.78	1.12	3.00	11.29	25.80	105.75

* PM = Particulate Matter.

**TPM = Total Particulate Matter.

Regarding the concentration of particles quantified in the air, from 0.5 to < 2.5 μg/m^3^ were obtained in the EU, and the highest values were in F1 and F2. Regarding particles of 2.5 and 10 μm, these were recorded on both monitored floors, presenting the highest values in F1. Likewise, the highest value of particles was recorded in F1, followed by F2.

## Discussion

The outdoor environment affects the composition of micro and nano-sized bioaerosols. Studies carried out worldwide have related intense human activity and the presence of internal sources such as water leaks, air conditioning, humidifiers, and ambient airflow with the increased production of bacteria in these environments [12]. In this study, we evaluate and determined the presence and diversity of airborne bacteria inside and outside one of the country’s most important public health hospitals, located in a city highly contaminated by gases and particles, such as Mexico City to characterize the airborne bacterial community composition from the hospital environment.

Airborne bacteria’s greatest diversity and richness were recorded on floors one and two from the hospital. This could be attributed to the intense daily activity in both areas since there are patients who are undergoing treatment, are going to surgery or are in recovering and are visited by doctors, nurses, medical residents, cleaning and nutrition staff, and relatives. In contrast, the Emergency Unit area showed a lower diversity of airborne bacteria since access is restricted to medical personnel.

The sampling carried out at the area outside the hospital, with the AVPS sampler, captured a great richness of bacterial species, which is explained because it is an area with a high density of particles in the environment, which come from both the traffic of people who continually enter and leave the hospital, as well as the patients and relatives who are there. Likewise, the presence of street vendors, vehicular traffic, and the dust emanating from the nearby construction of buildings for new hospitals causes particles to disperse in the air outside the hospital and his intrusion into it.

The use of two different samplers showed differences between them, CųS-Sampler presented relative abundance values of 89.55% while AVPS 74.40%. It should be noted that CųS-Sampler, showed 25.6% of bacteria undetected by the AVPS sampler.

These results indicate that CųS-Sampler has a greater capacity to capture a broad spectrum of airborne bacteria, one of the main factors being the high flow rate of 250 L/min of air it sucks, unlike the AVPS, which has a flow rate of 28.3 L/min. Based on these results, it is advisable to use both samplers simultaneously to achieve broader detection of airborne bacterial communities. This finding may provide a deeper understanding of microbial communities in indoor and outdoor environments and the use of different samplers could result in a better air monitoring strategy.

Regarding the size of particles suspended in the hospital air and their association with airborne bacteria, it was found that the highest particle concentrations fluctuate mainly between 2.5 μm and 10 μm and even up to total particles (TPM), predominating on floors 1 and 2 and EU. The presence of airborne particles probably favors their adhesion to bacteria suspended in air, increasing the exposure to bacteria associated with these particles and not only to aggregates of bacteria dispersed in the air. This is consistent with studies indicating that a great diversity of bacteria is associated with particles ranging from 0.65 μm to 2.5 μm and up to 5 μm [26,27]. Smaller bacteria can easily adhere to particles as fine as 2.5 μm and even smaller. Bacteria are often adsorbed onto solid or liquid particles suspended in the air, forming bioaerosols or droplets that patients can inhale [2]. Particle size is an essential characteristic that determines the degree of inhalation, which significantly and directly affects human health [28].

The highest temperatures were recorded in F1 and F2. In these floors, an average temperature of 27°C and a relative humidity of 42% was obtained. These warm and humid environmental conditions could be suitable for bacterial proliferation. However, it is essential to consider that in the hospital environment, there is a combination of factors capable of influencing bacterial outbreak, such as hygiene practices, environmental control measures, and effectiveness in managing the risk of infection.

The overall analysis of airborne bacteria in different hospital areas showed that the phyla Firmicutes, Proteobacteria and Actinobacteria had the highest relative abundances with both samplers. Only variations were seen in the order of appearance and some genera collected. The use of both samplers allowed us to collect a broad spectrum of airborne bacteria, which provides an opportunity to characterize better the composition of the community of airborne bacteria that are present in the hospital environment, determining both pathogenic species commonly found in these environments and some rare disease-causing species. Some pathogenic species isolated from air and considered to be of high health risk were *Stenotrophomonas maltophilia*, *Micrococcus luteus*, *Staphylococcus lentus*, *S*. *haemolyticus*, *Enterococcus gallinarum*, *Bacillus cereus*, among others. Our results coincide with previous hospital studies, which found that the most prevalent airborne bacteria belonged to the phyla Actinobacteria and Firmicutes, each with abundances greater than 1% [29]. In another study, Actinobacteria were more abundant during the summer, while Firmicutes increased during the winter [30]. Additionally, findings correspond with studies on the dominant microbiome in the air of urban areas, showing similar bacterial compositions across different locations, seasons, and levels of pollution as the one that characterizes Mexico City [31–35].

Thirty-two species of bacteria were identified, with the highest number found on floors 1, 2 and Emergency unit of the hospital. The presence of potentially pathogenic species in these areas indicates a significant risk to patients with weakened immune systems, who are especially vulnerable due to the various treatments they are subjected to, such as preoperative care, surgery, postoperative, and recovery.

The emergency unit presented a lower bacterial diversity due to restricted access. Gram-positive bacteria of the Firmicutes phylum were identified on the first and second floors, with abundant genera such as *Bacillus*, *Staphylococcus*, and *Clostridium*. These bacteria are common in hospital environments on surfaces and in the air [27–37]. Furthermore, these genera have been reported in hospitals in several countries [38–45].

*Bacillus* strains were isolated both inside and outside the hospital. These genera are very diverse, and their species are widely found in the environment, such as soil, dust, and air. According to several authors, these strains can be dispersed to different hospitals or clinical area [46–49]. Six Bacillus species were identified in all hospital areas, *B*. *toyonensis* was isolated outside the hospital, while the *B*. *cereus* group was found in the emergency unit. This agrees with previous studies that have isolated *Bacillus cereus* from dust, clean towels and gowns in hospitals, highlighting its presence in hospital materials [50]. Although many *Bacillus* species are harmless, their presence in hospital environment is associated with the potential risk of nosocomial infections, especially in people with compromised immune systems [25,51].

Staphylococcus species, especially those associated with nosocomial infections and the hospital environment, are considered high-risk factors and constitute significant concerns about infection control and patient safety [52]. On the second floor, *Staphylococcus haemolyticus* was the predominant species. This species is linked to joint infections, meningitis, bacteremia, and septicemia [53]. Also, *S*. *lentus* species is associated to infections in various parts of the body, including the spleen, peritoneum, blood, urine, and cerebrospinal fluid, highlighting the potential risks of these bacteria in healthcare settings [30,53]. The presence of *Staphylococcus gallinarum*, *S*. *haemolyticus*, *S*. *xylosus* and *S*. *equorum* outside the hospital can represent a risk to vulnerable individuals with weakened immune systems, even without being hospital patients [29,54].

Although in this study, it was only possible to identify a genus level to Clostridium bacteria, it is essential to consider that several studies have documented the presence of this type of bacteria in hospital settings, including pathogenic species capable of disseminating spores through the air, such as *C*. *botulinum*, *C*. *tetani*, *C*. *perfringens*, *C*. *welchii*, and *C*. *difficile*, being concerns due to their potential to cause nosocomial infections [55–58]; In particular, *C*. *difficile* is a common pathogen in a healthcare environment, responsible for nosocomial diarrhea and severe complications such as colitis, megacolon, intestinal perforation, and even death [58]. The potential transmission of *Clostridium* species, including its transmission through the air in these settings, highlights the need to implement strict infection control measures to protect patient safety [59].

Within the Firmicutes (Bacillota), the genera *Exiguobacterium*, *Lysinibacillus* and *Planococcus* were found. *E*. *acetylicum and E*. *aurantiacum* were isolated both inside and outside of the hospital, and although *Exiguobacterium* is rarely associated with human infections, bacteremia and community-acquired pneumonia cases have been reported, especially in immunocompromised patients [60]. Furthermore, *E*. *aurantiacum* has recently been identified as a novel virulent pigment-producing pathogen associated with corneal ulcers, suggesting its potential pathogenicity in human infections [61].

This study revealed the highest abundance of Proteobacteria in the emergency unit, possibly due to the high patient flow with great diversity of medical conditions and healthcare activities. It is crucial to distinguish between the Proteobacteria; some species have environmental functions, and others cause disease, especially in healthcare settings or when acting as opportunistic pathogens in immunocompromised individuals. Several genera of Proteobacteria were isolated from the air in sampled areas of the hospital, including species from the families Enterobacteriaceae and Pseudomonadaceae, which can cause severe diseases including sepsis, pneumonia, endocarditis, meningitis, skin and wound infections, urinary tract infections, peritonitis, eye and respiratory infections, gastrointestinal and bloodstream infections, and arthritis [62,63]. These bacteria are common in these environments and pose a high risk to patients’ health.

The emergence of multidrog resistance bacteria in healthcare environments and opportunistic infections underlines the urgent need for effective infection control measures and the development of new therapeutic strategies to combat such challenging pathogens. In particular, bacteria such as *Stenotrophomonas maltophilia*, isolated from the air of floor one, have been linked to a high risk of immunosuppressed patients due to their high resistance to broad-spectrum antibiotics and difficulty in treating [64].

Actinobacteria were particularly abundant on the second floor and outside the hospital. This phylum includes genera such as *Corynebacterium*, *Mycobacterium*, *Kocuria*, *Micrococcus* and *Nocardioides*, which are usually found both in healthcare environments and natural sources such as soil and vegetation. Some identified genera are opportunistic pathogens, causing severe infections such as septic shock, endocarditis, skin, and deep infections.

The isolation of *Psychrobacter phenylpyruvicus* and *Terribacillus goriensis* in emergencies (EU) may indicate potential environmental contamination. It is interesting to note that these types of bacteria are known as extremephilic environmental bacteria, which are adapted to and thrive in extreme environmental conditions.

This study allowed us to determine the main genera and species present in the hospital air, detect the presence of pathogenic bacteria, and determine the areas with the greatest abundance of bacteria, depending on the activities carried out in each area and its environmental characteristics such as temperature, relative humidity, concentration and size of suspended particles.

The information generated will provide guidelines for future specific studies to link the presence of pathogenic bacteria to nosocomial infections, as well as to investigate the relationship between patient well-being and environmental quality. This can contribute, for example, to research such as that conducted by Murray and colleagues [65] on antimicrobial resistance (AMR), a significant threat to human health worldwide. Based on the findings obtained, the researchers assessed and determined that AMR is one of the leading causes of death globally, underlining the importance of understanding the microbiota and quantifying the burden of antimicrobial resistance. This is crucial for developing infection prevention and control programs and the research and development of new antibiotics.

The results of the microbial load in the hospital highlight the importance of carrying out regular monitoring of air bioaerosols to carry out epidemiological surveillance of air quality. This would allow the identification of the presence of pathogenic or opportunistic bacteria, as well as other bioaerosols such as fungi and viruses. With this information, the hospital could improve and strengthen its health programs, for the benefit of patients.

## Conclusions

The study highlights that the most active hospital areas have a higher diversity and richness of airborne bacterial species. Using at least two air samplers is recommended to collect these microorganisms thoroughly. Airborne particles between 2.5 and 10 μm predominate in these active areas of the hospital, and 32 bacterial species were identified. This reflects a higher diversity of potentially pathogenic species in areas of high activity and high levels of airborne particles. Notably, the presence of potentially infectious bacteria such as *Bacillus* spp., *B*. *cereus*, *B*. *pumilus*, *Clostridium* spp., *Enterococcus gallinarum*, *Micrococcus luteus*, *Staphylococcus* spp., *S*. *haemolyticus*, *S*. *lentus*, *Stenotrophomonas maltophilia* and *Stutzerimonas stutzeri* in hospital air represents a significant risk for patients in these areas. These findings suggest the need to implement periodic epidemiological surveillance of air quality to improve and strengthen health programs that benefit patients.

**Highligts**

1. The areas inside the hospital with most activity present a greater diversity and richness of airborne bacterial species.

2. In order to achieve a broader collection of airborne microorganisms, it is recommended the use of at least two types of air samplers.

3. Air particles between 2.5 μm and 10 μm predominated among the most active areas of the hospital.

4. A total of 32 species were identified.

6. The greater diversity of potentially pathogenic species showed that the most significant risk for patients occurred in the areas of greatest activity and higher levels of air particles.

7. Potentially infectious bacteria such as *Bacillus* spp, *B*. *cereus*, *B*. *pumilus*, *Clostridium* spp., *Enterococcus gallinarum*, *Micrococcus luteus*, *Staphylococcus* spp., *S*. *haemolyticus*, *S*. *lentus*, *Stenotrophomonas maltophilia*, *Stutzerimonas stutzeri*, were identified in the hospital air.

## Supporting information

S1 Text Quality control report for each sample in HTML format. (ZIP)

S1 Fig KRONA graph shows all phyla and genera recorded with AVPS and CųS-Sampler in each sampled area. (ZIP)
