# Supplementary material for: Assessment of airborne bacteria from a public health institution in Mexico City
Source: PLOS Glob Public Health. 2024 Nov 7;4(11):e0003672. doi: 10.1371/journal.pgph.0003672 (PMC11542838; doi:10.1371/journal.pgph.0003672)
Supplement: S1 Text — (ZIP) [file pgph.0003672.s001.zip › Hospital_16S_QC/21022023_BED2_16S_S21_L001_R1_001_fastqc.html]

21022023\_BED2\_16S\_S21\_L001\_R1\_001.fastq.gz FastQC Report 

FastQC Report

Tue 14 Mar 2023  
21022023\_BED2\_16S\_S21\_L001\_R1\_001.fastq.gz

## Summary

- Basic Statistics
- Per base sequence quality
- Per tile sequence quality
- Per sequence quality scores
- Per base sequence content
- Per sequence GC content
- Per base N content
- Sequence Length Distribution
- Sequence Duplication Levels
- Overrepresented sequences
- Adapter Content
- Kmer Content

## Basic Statistics

| Measure | Value |
| --- | --- |
| Filename | 21022023\_BED2\_16S\_S21\_L001\_R1\_001.fastq.gz |
| File type | Conventional base calls |
| Encoding | Sanger / Illumina 1.9 |
| Total Sequences | 1188656 |
| Sequences flagged as poor quality | 0 |
| Sequence length | 35-301 |
| %GC | 55 |

## Per base sequence quality

## Per tile sequence quality

## Per sequence quality scores

## Per base sequence content

## Per sequence GC content

## Per base N content

## Sequence Length Distribution

## Sequence Duplication Levels

## Overrepresented sequences

| Sequence | Count | Percentage | Possible Source |
| --- | --- | --- | --- |
| CCTACGGGTGGCAGCAGTAGGGAATCTTCCACAATGGACGAAAGTCTGAT | 79759 | 6.7100153450619855 | No Hit |
| CCTACGGGAGGCAGCAGTAGGGAATCTTCCACAATGGACGAAAGTCTGAT | 79197 | 6.662735055390289 | No Hit |
| CCTACGGGAGGCAGCAGTGGGGAATATTGCACAATGGGCGCAAGCCTGAT | 71527 | 6.017468468589735 | No Hit |
| CCTACGGGTGGCAGCAGTGGGGAATATTGCACAATGGGCGCAAGCCTGAT | 70327 | 5.916514113418852 | No Hit |
| CCTACGGGGGGCAGCAGTAGGGAATCTTCCACAATGGACGAAAGTCTGAT | 67042 | 5.640151566138563 | No Hit |
| CCTACGGGGGGCAGCAGTGGGGAATATTGCACAATGGGCGCAAGCCTGAT | 61301 | 5.157169105275202 | No Hit |
| CCTACGGGCGGCAGCAGTAGGGAATCTTCCACAATGGACGAAAGTCTGAT | 52971 | 4.4563776231306615 | No Hit |
| CCTACGGGCGGCAGCAGTGGGGAATATTGCACAATGGGCGCAAGCCTGAT | 47507 | 3.996698792585912 | No Hit |
| CCTACGGGAGGCTGCAGTGGGGAATATTGCACAATGGGCGCAAGCCTGAT | 46645 | 3.924179914121495 | No Hit |
| CCTACGGGTGGCTGCAGTGGGGAATATTGCACAATGGGCGCAAGCCTGAT | 41871 | 3.522549837800003 | No Hit |
| CCTACGGGAGGCTGCAGTAGGGAATCTTCCACAATGGACGAAAGTCTGAT | 36497 | 3.0704425838930693 | No Hit |
| CCTACGGGGGGCTGCAGTGGGGAATATTGCACAATGGGCGCAAGCCTGAT | 33500 | 2.818309081853791 | No Hit |
| CCTACGGGTGGCAGCAGTAGGGAATCTTCCGCAATGGACGAAAGTCTGAC | 31864 | 2.6806746443041556 | No Hit |
| CCTACGGGAGGCAGCAGTAGGGAATCTTCCGCAATGGACGAAAGTCTGAC | 31843 | 2.678907943088665 | No Hit |
| CCTACGGGTGGCTGCAGTAGGGAATCTTCCACAATGGACGAAAGTCTGAT | 30971 | 2.6055477783311574 | No Hit |
| CCTACGGGGGGCAGCAGTAGGGAATCTTCCGCAATGGACGAAAGTCTGAC | 27137 | 2.282998613560189 | No Hit |
| CCTACGGGCGGCTGCAGTGGGGAATATTGCACAATGGGCGCAAGCCTGAT | 27032 | 2.274165107482737 | No Hit |
| CCTACGGGGGGCTGCAGTAGGGAATCTTCCACAATGGACGAAAGTCTGAT | 25500 | 2.1452800473812443 | No Hit |
| CCTACGGGCGGCAGCAGTAGGGAATCTTCCGCAATGGACGAAAGTCTGAC | 21446 | 1.8042225841622808 | No Hit |
| CCTACGGGCGGCTGCAGTAGGGAATCTTCCACAATGGACGAAAGTCTGAT | 21302 | 1.792108061541775 | No Hit |
| CCTACGGGAGGCTGCAGTAGGGAATCTTCCGCAATGGACGAAAGTCTGAC | 14548 | 1.223903299188327 | No Hit |
| CCTACGGGAGGCAGCAGTGGGGAATATTGGACAATGGGGGGAACCCTGAT | 13069 | 1.0994770564402148 | No Hit |
| CCTACGGGTGGCAGCAGTGGGGAATATTGGACAATGGGGGGAACCCTGAT | 12709 | 1.0691907498889501 | No Hit |
| CCTACGGGTGGCTGCAGTAGGGAATCTTCCGCAATGGACGAAAGTCTGAC | 12384 | 1.041848945363503 | No Hit |
| CCTACGGGGGGCAGCAGTGGGGAATATTGGACAATGGGGGGAACCCTGAT | 10968 | 0.9227228062618621 | No Hit |
| CCTACGGGGGGCTGCAGTAGGGAATCTTCCGCAATGGACGAAAGTCTGAC | 10303 | 0.8667772677713317 | No Hit |
| CCTACGGGAGGCAGCAGTAGGGAATCTTCCGCAATGGGCGAAAGCCTGAC | 9044 | 0.7608593234712145 | No Hit |
| CCTACGGGTGGCAGCAGTAGGGAATCTTCCGCAATGGGCGAAAGCCTGAC | 8897 | 0.7484924149627815 | No Hit |
| CCTACGGGCGGCAGCAGTGGGGAATATTGGACAATGGGGGGAACCCTGAT | 8786 | 0.739154137109475 | No Hit |
| CCTACGGGAGGCTGCAGTGGGGAATATTGGACAATGGGGGGAACCCTGAT | 8583 | 0.722076025359734 | No Hit |
| CCTACGGGCGGCTGCAGTAGGGAATCTTCCGCAATGGACGAAAGTCTGAC | 8366 | 0.7038201127996662 | No Hit |
| CCTACGGGTGGCTGCAGTGGGGAATATTGGACAATGGGGGGAACCCTGAT | 7724 | 0.6498095327832443 | No Hit |
| CCTACGGGGGGCAGCAGTAGGGAATCTTCCGCAATGGGCGAAAGCCTGAC | 7673 | 0.6455189726884818 | No Hit |
| CCTACGGGGGGCTGCAGTGGGGAATATTGGACAATGGGGGGAACCCTGAT | 6093 | 0.5125957383801537 | No Hit |
| CCTACGGGCGGCAGCAGTAGGGAATCTTCCGCAATGGGCGAAAGCCTGAC | 6037 | 0.5078845351388459 | No Hit |
| CCTACGGGCGGCTGCAGTGGGGAATATTGGACAATGGGGGGAACCCTGAT | 5138 | 0.4322528973899934 | No Hit |
| CCTACGGGAGGCAGCAGTGGGGAATATTGGACAATGGGCGAAAGCCTGAT | 4408 | 0.3708389979943735 | No Hit |
| CCTACGGGTGGCAGCAGTGGGGAATATTGGACAATGGGCGAAAGCCTGAT | 4321 | 0.3635198072444845 | No Hit |
| CCTACGGGAGGCTGCAGTAGGGAATCTTCCGCAATGGGCGAAAGCCTGAC | 3936 | 0.3311302849604932 | No Hit |
| CCTACGGGGGGCAGCAGTGGGGAATATTGGACAATGGGCGAAAGCCTGAT | 3770 | 0.31716493249518785 | No Hit |
| CCTACGGGTGGCTGCAGTAGGGAATCTTCCGCAATGGGCGAAAGCCTGAC | 3359 | 0.28258806584916074 | No Hit |
| CCTACGGGAGGCTGCAGTGGGGAATATTGGACAATGGGCGAAAGCCTGAT | 2887 | 0.24287935281528042 | No Hit |
| CCTACGGGGGGCTGCAGTAGGGAATCTTCCGCAATGGGCGAAAGCCTGAC | 2801 | 0.23564429069470055 | No Hit |
| CCTACGGGCGGCAGCAGTGGGGAATATTGGACAATGGGCGAAAGCCTGAT | 2799 | 0.23547603343608242 | No Hit |
| CCTACGGGTGGCTGCAGTGGGGAATATTGGACAATGGGCGAAAGCCTGAT | 2611 | 0.2196598511259776 | No Hit |
| CCTACGGGAGGCAGCAGTGGGGAATATTGCACAATGGGCGAAAGCCTGAT | 2475 | 0.20821835753994425 | No Hit |
| CCTACGGGTGGCAGCAGTGGGGAATATTGCACAATGGGCGAAAGCCTGAT | 2437 | 0.20502146962619966 | No Hit |
| CCTACGGGGGGCAGCAGTGGGGAATATTGCACAATGGGCGAAAGCCTGAT | 2205 | 0.1855036276264958 | No Hit |
| CCTACGGGGGGCTGCAGTGGGGAATATTGGACAATGGGCGAAAGCCTGAT | 2162 | 0.18188609656620586 | No Hit |
| CCTACGGGCGGCTGCAGTAGGGAATCTTCCGCAATGGGCGAAAGCCTGAC | 2132 | 0.17936223768693382 | No Hit |
| CCTACGGGCGGCTGCAGTGGGGAATATTGGACAATGGGCGAAAGCCTGAT | 1862 | 0.15664750777348535 | No Hit |
| CCTACGGGAGGCTGCAGTGGGGAATATTGCACAATGGGCGAAAGCCTGAT | 1744 | 0.1467203295150153 | No Hit |
| CCTACGGGCGGCAGCAGTGGGGAATATTGCACAATGGGCGAAAGCCTGAT | 1717 | 0.14444885652367043 | No Hit |
| CCTACGGGTGGCTGCAGTGGGGAATATTGCACAATGGGCGAAAGCCTGAT | 1646 | 0.13847572384272658 | No Hit |
| GCTACGGGAGGCAGCAGTAGGGAATCTTCCACAATGGACGAAAGTCTGAT | 1471 | 0.12375321371363962 | No Hit |
| GCTACGGGGGGCAGCAGTAGGGAATCTTCCACAATGGACGAAAGTCTGAT | 1286 | 0.10818941729146196 | No Hit |

## Adapter Content

## Kmer Content

| Sequence | Count | PValue | Obs/Exp Max | Max Obs/Exp Position |
| --- | --- | --- | --- | --- |
| TGGAGAG | 25 | 5.16593E-10 | 296.4703 | 295 |
| TTTCGAT | 20 | 6.033406E-8 | 296.4703 | 295 |
| ATTCGCA | 10 | 8.339945E-4 | 296.4703 | 295 |
| ATTGCTA | 10 | 8.339945E-4 | 296.4703 | 295 |
| AGTACAG | 60 | 0.0 | 296.4703 | 295 |
| AGTTCGG | 310 | 0.0 | 296.47028 | 295 |
| ATTGGCA | 85 | 0.0 | 296.47028 | 295 |
| CTCGGTC | 45 | 0.0 | 294.1505 | 1 |
| CTAGGCT | 20 | 6.274604E-8 | 294.1505 | 1 |
| GGTGACT | 20 | 6.2773324E-8 | 294.1258 | 7 |
| TAGGCTA | 20 | 6.2773324E-8 | 294.1258 | 2 |
| TACAGGT | 20 | 6.2773324E-8 | 294.1258 | 3 |
| GGACAGC | 55 | 0.0 | 294.1258 | 9 |
| AAGGCAG | 10 | 8.5406407E-4 | 294.1258 | 8 |
| ACGAGCG | 10 | 8.5406407E-4 | 294.1258 | 4 |
| ATTTAGA | 180 | 0.0 | 294.12576 | 8 |
| ACAGGAG | 30 | 5.456968E-12 | 294.12576 | 4 |
| TACAGGC | 30 | 5.456968E-12 | 294.12576 | 3 |
| CATTTAG | 155 | 0.0 | 294.12576 | 7 |
| CCTACGG | 116295 | 0.0 | 293.94818 | 1 |

Produced by FastQC (version 0.11.7)
