# Supplementary material for: Assessment of airborne bacteria from a public health institution in Mexico City
Source: PLOS Glob Public Health. 2024 Nov 7;4(11):e0003672. doi: 10.1371/journal.pgph.0003672 (PMC11542838; doi:10.1371/journal.pgph.0003672)
Supplement: S1 Text — (ZIP) [file pgph.0003672.s001.zip › Hospital_16S_QC/21022023_BED2_16S_S21_L001_R2_001_fastqc.html]

21022023\_BED2\_16S\_S21\_L001\_R2\_001.fastq.gz FastQC Report 

FastQC Report

Tue 14 Mar 2023  
21022023\_BED2\_16S\_S21\_L001\_R2\_001.fastq.gz

## Summary

- Basic Statistics
- Per base sequence quality
- Per tile sequence quality
- Per sequence quality scores
- Per base sequence content
- Per sequence GC content
- Per base N content
- Sequence Length Distribution
- Sequence Duplication Levels
- Overrepresented sequences
- Adapter Content
- Kmer Content

## Basic Statistics

| Measure | Value |
| --- | --- |
| Filename | 21022023\_BED2\_16S\_S21\_L001\_R2\_001.fastq.gz |
| File type | Conventional base calls |
| Encoding | Sanger / Illumina 1.9 |
| Total Sequences | 1188656 |
| Sequences flagged as poor quality | 0 |
| Sequence length | 35-301 |
| %GC | 54 |

## Per base sequence quality

## Per tile sequence quality

## Per sequence quality scores

## Per base sequence content

## Per sequence GC content

## Per base N content

## Sequence Length Distribution

## Sequence Duplication Levels

## Overrepresented sequences

| Sequence | Count | Percentage | Possible Source |
| --- | --- | --- | --- |
| GACTACTGGGGTATCTAATCCTGTTTGCTCCCCACGCTTTCGCGCCTCAG | 57948 | 4.875085811201895 | No Hit |
| GACTACTAGGGTATCTAATCCTGTTTGCTCCCCACGCTTTCGCGCCTCAG | 51846 | 4.36173291515796 | No Hit |
| GACTACAGGGGTATCTAATCCTGTTTGCTCCCCACGCTTTCGCGCCTCAG | 50638 | 4.260105530952606 | No Hit |
| GACTACTCGGGTATCTAATCCTGTTTGCTCCCCACGCTTTCGCGCCTCAG | 49073 | 4.128444226083913 | No Hit |
| GACTACTGGGGTATCTAATCCTGTTTGCTCCCCACGCTTTCGCACCTGAG | 48446 | 4.075695575507128 | No Hit |
| GACTACCAGGGTATCTAATCCTGTTTGCTCCCCACGCTTTCGCGCCTCAG | 46639 | 3.923675142345641 | No Hit |
| GACTACAAGGGTATCTAATCCTGTTTGCTCCCCACGCTTTCGCGCCTCAG | 46444 | 3.907270059630372 | No Hit |
| GACTACCGGGGTATCTAATCCTGTTTGCTCCCCACGCTTTCGCGCCTCAG | 46423 | 3.905503358414882 | No Hit |
| GACTACTAGGGTATCTAATCCTGTTTGCTCCCCACGCTTTCGCACCTGAG | 43311 | 3.6436950640050614 | No Hit |
| GACTACAGGGGTATCTAATCCTGTTTGCTCCCCACGCTTTCGCACCTGAG | 42178 | 3.548377326997887 | No Hit |
| GACTACACGGGTATCTAATCCTGTTTGCTCCCCACGCTTTCGCGCCTCAG | 41672 | 3.5058082405674984 | No Hit |
| GACTACTCGGGTATCTAATCCTGTTTGCTCCCCACGCTTTCGCACCTGAG | 41037 | 3.4523865609562394 | No Hit |
| GACTACCCGGGTATCTAATCCTGTTTGCTCCCCACGCTTTCGCGCCTCAG | 40498 | 3.407041229758652 | No Hit |
| GACTACCAGGGTATCTAATCCTGTTTGCTCCCCACGCTTTCGCACCTGAG | 39037 | 3.2841293023381026 | No Hit |
| GACTACCGGGGTATCTAATCCTGTTTGCTCCCCACGCTTTCGCACCTGAG | 39011 | 3.281941957976067 | No Hit |
| GACTACAAGGGTATCTAATCCTGTTTGCTCCCCACGCTTTCGCACCTGAG | 38863 | 3.269490920838325 | No Hit |
| GACTACACGGGTATCTAATCCTGTTTGCTCCCCACGCTTTCGCACCTGAG | 34638 | 2.914047462007511 | No Hit |
| GACTACCCGGGTATCTAATCCTGTTTGCTCCCCACGCTTTCGCACCTGAG | 33694 | 2.8346300359397505 | No Hit |
| GACTACTGGGGTATCTAATCCTGTTCGCTCCCCACGCTTTCGCTCCTCAG | 14435 | 1.2143967640764022 | No Hit |
| GACTACTAGGGTATCTAATCCTGTTCGCTCCCCACGCTTTCGCTCCTCAG | 12783 | 1.0754162684578212 | No Hit |
| GACTACAGGGGTATCTAATCCTGTTCGCTCCCCACGCTTTCGCTCCTCAG | 12663 | 1.065320832940733 | No Hit |
| GACTACTCGGGTATCTAATCCTGTTCGCTCCCCACGCTTTCGCTCCTCAG | 12259 | 1.0313328666998693 | No Hit |
| GACTACTGGGGTATCTAATCCTGTTTGCTCCCCATGCTTTCGCACCTCAG | 12215 | 1.0276312070102704 | No Hit |
| GACTACCAGGGTATCTAATCCTGTTCGCTCCCCACGCTTTCGCTCCTCAG | 11766 | 0.9898574524504986 | No Hit |
| GACTACAAGGGTATCTAATCCTGTTCGCTCCCCACGCTTTCGCTCCTCAG | 11498 | 0.9673109797956684 | No Hit |
| GACTACCGGGGTATCTAATCCTGTTCGCTCCCCACGCTTTCGCTCCTCAG | 11484 | 0.9661331789853413 | No Hit |
| GACTACTAGGGTATCTAATCCTGTTTGCTCCCCATGCTTTCGCACCTCAG | 10845 | 0.9123749848568468 | No Hit |
| GACTACACGGGTATCTAATCCTGTTCGCTCCCCACGCTTTCGCTCCTCAG | 10500 | 0.8833506077452181 | No Hit |
| GACTACAGGGGTATCTAATCCTGTTTGCTCCCCATGCTTTCGCACCTCAG | 10489 | 0.8824251928228184 | No Hit |
| GACTACTCGGGTATCTAATCCTGTTTGCTCCCCATGCTTTCGCACCTCAG | 10325 | 0.8686280976161311 | No Hit |
| GACTACCCGGGTATCTAATCCTGTTCGCTCCCCACGCTTTCGCTCCTCAG | 10186 | 0.8569342181421706 | No Hit |
| GACTACCGGGGTATCTAATCCTGTTTGCTCCCCATGCTTTCGCACCTCAG | 9796 | 0.824124052711634 | No Hit |
| GACTACAAGGGTATCTAATCCTGTTTGCTCCCCATGCTTTCGCACCTCAG | 9781 | 0.8228621232719979 | No Hit |
| GACTACCAGGGTATCTAATCCTGTTTGCTCCCCATGCTTTCGCACCTCAG | 9593 | 0.8070459409618931 | No Hit |
| GACTACACGGGTATCTAATCCTGTTTGCTCCCCATGCTTTCGCACCTCAG | 8704 | 0.7322555895061313 | No Hit |
| GACTACCCGGGTATCTAATCCTGTTTGCTCCCCATGCTTTCGCACCTCAG | 8129 | 0.683881627653417 | No Hit |
| GACTACTGGGGTATCTAATCCTGTTTGCTCCCCACGCTTTCGAGCCTCAG | 6931 | 0.583095529741153 | No Hit |
| GACTACTAGGGTATCTAATCCTGTTTGCTCCCCACGCTTTCGAGCCTCAG | 6090 | 0.5123433524922265 | No Hit |
| GACTACAGGGGTATCTAATCCTGTTTGCTCCCCACGCTTTCGAGCCTCAG | 5769 | 0.4853380624840155 | No Hit |
| GACTACTCGGGTATCTAATCCTGTTTGCTCCCCACGCTTTCGAGCCTCAG | 5640 | 0.4744854693031457 | No Hit |
| GACTACAAGGGTATCTAATCCTGTTTGCTCCCCACGCTTTCGAGCCTCAG | 5585 | 0.46985839469114693 | No Hit |
| GACTACCAGGGTATCTAATCCTGTTTGCTCCCCACGCTTTCGAGCCTCAG | 5480 | 0.4610248886136948 | No Hit |
| GACTACCGGGGTATCTAATCCTGTTTGCTCCCCACGCTTTCGAGCCTCAG | 5441 | 0.4577438720706411 | No Hit |
| GACTACACGGGTATCTAATCCTGTTTGCTCCCCACGCTTTCGAGCCTCAG | 4857 | 0.40861275255414514 | No Hit |
| GACTACCCGGGTATCTAATCCTGTTTGCTCCCCACGCTTTCGAGCCTCAG | 4556 | 0.38329003513211557 | No Hit |
| GACTACTGGGGTATCTAATCCTGTTTGCTCCCCACGCTTTCGCACCTCAG | 4024 | 0.3385336043396912 | No Hit |
| GACTACTGGGGTATCTAATCCTGTTCGCTCCCCACGCTTTCGCGCCTCAG | 3714 | 0.31245372925388004 | No Hit |
| GACTACTAGGGTATCTAATCCTGTTTGCTCCCCACGCTTTCGCACCTCAG | 3589 | 0.30193765059024646 | No Hit |
| GACTACAGGGGTATCTAATCCTGTTTGCTCCCCACGCTTTCGCACCTCAG | 3482 | 0.29293588725417613 | No Hit |
| GACTACTCGGGTATCTAATCCTGTTTGCTCCCCACGCTTTCGCACCTCAG | 3418 | 0.2875516549783958 | No Hit |
| GACTACAAGGGTATCTAATCCTGTTTGCTCCCCACGCTTTCGCACCTCAG | 3392 | 0.28536431061636 | No Hit |
| GACTACTAGGGTATCTAATCCTGTTCGCTCCCCACGCTTTCGCGCCTCAG | 3284 | 0.27627841865098063 | No Hit |
| GACTACCGGGGTATCTAATCCTGTTTGCTCCCCACGCTTTCGCACCTCAG | 3245 | 0.27299740210792695 | No Hit |
| GACTACCAGGGTATCTAATCCTGTTTGCTCCCCACGCTTTCGCACCTCAG | 3199 | 0.2691274851597098 | No Hit |
| GACTACTCGGGTATCTAATCCTGTTCGCTCCCCACGCTTTCGCGCCTCAG | 3104 | 0.26113526537534826 | No Hit |
| GACTACAGGGGTATCTAATCCTGTTCGCTCCCCACGCTTTCGCGCCTCAG | 3057 | 0.2571812197978221 | No Hit |
| GACTACCGGGGTATCTAATCCTGTTCGCTCCCCACGCTTTCGCGCCTCAG | 2963 | 0.24927312864276965 | No Hit |
| GACTACCAGGGTATCTAATCCTGTTCGCTCCCCACGCTTTCGCGCCTCAG | 2962 | 0.2491890000134606 | No Hit |
| GACTACAAGGGTATCTAATCCTGTTCGCTCCCCACGCTTTCGCGCCTCAG | 2957 | 0.24876835686691523 | No Hit |
| GACTACACGGGTATCTAATCCTGTTTGCTCCCCACGCTTTCGCACCTCAG | 2922 | 0.24582385484109784 | No Hit |
| GACTACCCGGGTATCTAATCCTGTTTGCTCCCCACGCTTTCGCACCTCAG | 2815 | 0.23682209150502753 | No Hit |
| GACTACACGGGTATCTAATCCTGTTCGCTCCCCACGCTTTCGCGCCTCAG | 2681 | 0.22554885517761233 | No Hit |
| GACTACCCGGGTATCTAATCCTGTTCGCTCCCCACGCTTTCGCGCCTCAG | 2538 | 0.21351846118641557 | No Hit |
| GACTACTGGGGTATCTAATCCTGTTCGCTCCCCATGCTTTCGCTCCTCAG | 1564 | 0.13157717623938295 | No Hit |
| GACTACTAGGGTATCTAATCCTGTTCGCTCCCCATGCTTTCGCTCCTCAG | 1311 | 0.11029263302418868 | No Hit |
| GACTACTCGGGTATCTAATCCTGTTCGCTCCCCATGCTTTCGCTCCTCAG | 1265 | 0.10642271607597152 | No Hit |
| GACTACCAGGGTATCTAATCCTGTTCGCTCCCCATGCTTTCGCTCCTCAG | 1233 | 0.10373059993808133 | No Hit |
| GACTACAGGGGTATCTAATCCTGTTCGCTCCCCATGCTTTCGCTCCTCAG | 1199 | 0.100870226541573 | No Hit |

## Adapter Content

## Kmer Content

| Sequence | Count | PValue | Obs/Exp Max | Max Obs/Exp Position |
| --- | --- | --- | --- | --- |
| ATTAGGT | 5 | 9.106568E-5 | 9477.8 | 295 |
| GTTAGGG | 105 | 0.0 | 9477.8 | 295 |
| CTTGGTG | 5 | 9.106568E-5 | 9477.8 | 295 |
| CTTATCG | 5 | 9.106568E-5 | 9477.8 | 295 |
| GTATGCG | 5 | 9.106568E-5 | 9477.8 | 295 |
| GTATGCA | 5 | 9.106568E-5 | 9477.8 | 295 |
| TATTCGA | 5 | 9.106568E-5 | 9477.8 | 295 |
| ACTGTGG | 5 | 9.106568E-5 | 9477.8 | 295 |
| TTTATGG | 5 | 9.106568E-5 | 9477.8 | 295 |
| CTATTGA | 5 | 9.106568E-5 | 9477.8 | 295 |
| CTTAGGG | 10 | 2.5549525E-8 | 9477.8 | 295 |
| GATAGCG | 10 | 2.5549525E-8 | 9477.8 | 295 |
| GGTAGGG | 5 | 9.106568E-5 | 9477.8 | 295 |
| GATAGAA | 5 | 9.106568E-5 | 9477.8 | 295 |
| GTTAGTG | 15 | 7.2759576E-12 | 9477.799 | 295 |
| GGTAGCG | 15 | 7.2759576E-12 | 9477.799 | 295 |
| GTTAGCG | 1660 | 0.0 | 9335.062 | 295 |
| GTTATCG | 50 | 0.0 | 8530.02 | 295 |
| GTTTGCG | 100 | 0.0 | 8056.13 | 295 |
| GTTAGCA | 55 | 0.0 | 7754.563 | 295 |

Produced by FastQC (version 0.11.7)
