# Supplementary material for: Assessment of airborne bacteria from a public health institution in Mexico City
Source: PLOS Glob Public Health. 2024 Nov 7;4(11):e0003672. doi: 10.1371/journal.pgph.0003672 (PMC11542838; doi:10.1371/journal.pgph.0003672)
Supplement: S1 Text — (ZIP) [file pgph.0003672.s001.zip › Hospital_16S_QC/21022023_BED3_16S_S22_L001_R1_001_fastqc.html]

21022023\_BED3\_16S\_S22\_L001\_R1\_001.fastq.gz FastQC Report 

FastQC Report

Tue 14 Mar 2023  
21022023\_BED3\_16S\_S22\_L001\_R1\_001.fastq.gz

## Summary

- Basic Statistics
- Per base sequence quality
- Per tile sequence quality
- Per sequence quality scores
- Per base sequence content
- Per sequence GC content
- Per base N content
- Sequence Length Distribution
- Sequence Duplication Levels
- Overrepresented sequences
- Adapter Content
- Kmer Content

## Basic Statistics

| Measure | Value |
| --- | --- |
| Filename | 21022023\_BED3\_16S\_S22\_L001\_R1\_001.fastq.gz |
| File type | Conventional base calls |
| Encoding | Sanger / Illumina 1.9 |
| Total Sequences | 1061391 |
| Sequences flagged as poor quality | 0 |
| Sequence length | 35-301 |
| %GC | 55 |

## Per base sequence quality

## Per tile sequence quality

## Per sequence quality scores

## Per base sequence content

## Per sequence GC content

## Per base N content

## Sequence Length Distribution

## Sequence Duplication Levels

## Overrepresented sequences

| Sequence | Count | Percentage | Possible Source |
| --- | --- | --- | --- |
| CCTACGGGAGGCAGCAGTGGGGAATATTGGACAATGGGCGAAAGCCTGAT | 48496 | 4.569098475491124 | No Hit |
| CCTACGGGAGGCAGCAGTAGGGAATCTTCCACAATGGACGAAAGTCTGAT | 47406 | 4.466403050336775 | No Hit |
| CCTACGGGTGGCAGCAGTGGGGAATATTGGACAATGGGCGAAAGCCTGAT | 47253 | 4.451988004420614 | No Hit |
| CCTACGGGTGGCAGCAGTAGGGAATCTTCCACAATGGACGAAAGTCTGAT | 46973 | 4.425607528234176 | No Hit |
| CCTACGGGGGGCAGCAGTGGGGAATATTGGACAATGGGCGAAAGCCTGAT | 40178 | 3.785409900781145 | No Hit |
| CCTACGGGGGGCAGCAGTAGGGAATCTTCCACAATGGACGAAAGTCTGAT | 40130 | 3.7808875334348984 | No Hit |
| CCTACGGGTGGCAGCAGTAGGGAATCTTCCGCAATGGACGAAAGTCTGAC | 36882 | 3.474874009672213 | No Hit |
| CCTACGGGAGGCAGCAGTAGGGAATCTTCCGCAATGGACGAAAGTCTGAC | 36527 | 3.4414273345072646 | No Hit |
| CCTACGGGAGGCTGCAGTGGGGAATATTGGACAATGGGCGAAAGCCTGAT | 32683 | 3.079261082862018 | No Hit |
| CCTACGGGCGGCAGCAGTGGGGAATATTGGACAATGGGCGAAAGCCTGAT | 32365 | 3.0493003991931342 | No Hit |
| CCTACGGGCGGCAGCAGTAGGGAATCTTCCACAATGGACGAAAGTCTGAT | 31703 | 2.9869294162094837 | No Hit |
| CCTACGGGGGGCAGCAGTAGGGAATCTTCCGCAATGGACGAAAGTCTGAC | 31216 | 2.941046230842357 | No Hit |
| CCTACGGGTGGCTGCAGTGGGGAATATTGGACAATGGGCGAAAGCCTGAT | 29393 | 2.769290487671367 | No Hit |
| CCTACGGGCGGCAGCAGTAGGGAATCTTCCGCAATGGACGAAAGTCTGAC | 24447 | 2.3032982190352094 | No Hit |
| CCTACGGGGGGCTGCAGTGGGGAATATTGGACAATGGGCGAAAGCCTGAT | 22783 | 2.1465228176986617 | No Hit |
| CCTACGGGAGGCTGCAGTAGGGAATCTTCCACAATGGACGAAAGTCTGAT | 22132 | 2.0851882105651924 | No Hit |
| CCTACGGGAGGCAGCAGTGGGGAATATTGCACAATGGGCGCAAGCCTGAT | 22118 | 2.0838691867558703 | No Hit |
| CCTACGGGTGGCAGCAGTGGGGAATATTGCACAATGGGCGCAAGCCTGAT | 21843 | 2.0579597905013327 | No Hit |
| CCTACGGGCGGCTGCAGTGGGGAATATTGGACAATGGGCGAAAGCCTGAT | 19143 | 1.8035766272749627 | No Hit |
| CCTACGGGGGGCAGCAGTGGGGAATATTGCACAATGGGCGCAAGCCTGAT | 19085 | 1.7981121000649147 | No Hit |
| CCTACGGGTGGCTGCAGTAGGGAATCTTCCACAATGGACGAAAGTCTGAT | 18817 | 1.7728622157150382 | No Hit |
| CCTACGGGAGGCTGCAGTAGGGAATCTTCCGCAATGGACGAAAGTCTGAC | 16937 | 1.5957361613203804 | No Hit |
| CCTACGGGAGGCTGCAGTGGGGAATATTGCACAATGGGCGCAAGCCTGAT | 15938 | 1.5016143909266235 | No Hit |
| CCTACGGGGGGCTGCAGTAGGGAATCTTCCACAATGGACGAAAGTCTGAT | 15601 | 1.4698636035165173 | No Hit |
| CCTACGGGCGGCAGCAGTGGGGAATATTGCACAATGGGCGCAAGCCTGAT | 15101 | 1.422755610326449 | No Hit |
| CCTACGGGTGGCTGCAGTAGGGAATCTTCCGCAATGGACGAAAGTCTGAC | 14455 | 1.3618920831248804 | No Hit |
| CCTACGGGTGGCTGCAGTGGGGAATATTGCACAATGGGCGCAAGCCTGAT | 14396 | 1.3563333399284525 | No Hit |
| CCTACGGGCGGCTGCAGTAGGGAATCTTCCACAATGGACGAAAGTCTGAT | 12775 | 1.2036092260062503 | No Hit |
| CCTACGGGTGGCAGCAGTGGGGAATATTGCACAATGGGCGAAAGCCTGAT | 12143 | 1.1440647226140037 | No Hit |
| CCTACGGGGGGCTGCAGTAGGGAATCTTCCGCAATGGACGAAAGTCTGAC | 12047 | 1.1350199879215106 | No Hit |
| CCTACGGGAGGCAGCAGTGGGGAATATTGCACAATGGGCGGAAGCCTGAT | 11955 | 1.1263521171745379 | No Hit |
| CCTACGGGAGGCAGCAGTGGGGAATATTGCACAATGGGCGAAAGCCTGAT | 11932 | 1.1241851494877948 | No Hit |
| CCTACGGGTGGCAGCAGTGGGGAATATTGCACAATGGGCGGAAGCCTGAT | 11703 | 1.1026096886067434 | No Hit |
| CCTACGGGGGGCTGCAGTGGGGAATATTGCACAATGGGCGCAAGCCTGAT | 11221 | 1.0571975831715172 | No Hit |
| CCTACGGGGGGCAGCAGTGGGGAATATTGCACAATGGGCGAAAGCCTGAT | 10163 | 0.9575170695813324 | No Hit |
| CCTACGGGGGGCAGCAGTGGGGAATATTGCACAATGGGCGGAAGCCTGAT | 10128 | 0.9542195100580276 | No Hit |
| CCTACGGGCGGCTGCAGTAGGGAATCTTCCGCAATGGACGAAAGTCTGAC | 9804 | 0.9236935304708631 | No Hit |
| CCTACGGGCGGCTGCAGTGGGGAATATTGCACAATGGGCGCAAGCCTGAT | 9484 | 0.8935444148292193 | No Hit |
| CCTACGGGAGGCTGCAGTGGGGAATATTGCACAATGGGCGAAAGCCTGAT | 8510 | 0.8017780440949659 | No Hit |
| CCTACGGGAGGCTGCAGTGGGGAATATTGCACAATGGGCGGAAGCCTGAT | 8421 | 0.7933928213071338 | No Hit |
| CCTACGGGCGGCAGCAGTGGGGAATATTGCACAATGGGCGAAAGCCTGAT | 8194 | 0.7720057923988426 | No Hit |
| CCTACGGGCGGCAGCAGTGGGGAATATTGCACAATGGGCGGAAGCCTGAT | 7839 | 0.738559117233894 | No Hit |
| CCTACGGGTGGCTGCAGTGGGGAATATTGCACAATGGGCGAAAGCCTGAT | 7776 | 0.7326235100919454 | No Hit |
| CCTACGGGTGGCTGCAGTGGGGAATATTGCACAATGGGCGGAAGCCTGAT | 7589 | 0.7150051206388597 | No Hit |
| CCTACGGGGGGCTGCAGTGGGGAATATTGCACAATGGGCGAAAGCCTGAT | 6160 | 0.5803704761016439 | No Hit |
| CCTACGGGGGGCTGCAGTGGGGAATATTGCACAATGGGCGGAAGCCTGAT | 6091 | 0.5738695730414145 | No Hit |
| CCTACGGGAGGCAGCAGTAGGGAATCTTCCGCAATGGGCGAAAGCCTGAC | 5878 | 0.5538015679424454 | No Hit |
| CCTACGGGTGGCAGCAGTAGGGAATCTTCCGCAATGGGCGAAAGCCTGAC | 5765 | 0.5431551614814898 | No Hit |
| CCTACGGGGGGCAGCAGTAGGGAATCTTCCGCAATGGGCGAAAGCCTGAC | 5197 | 0.489640481217572 | No Hit |
| CCTACGGGCGGCTGCAGTGGGGAATATTGCACAATGGGCGGAAGCCTGAT | 5050 | 0.4757907312196919 | No Hit |
| CCTACGGGCGGCTGCAGTGGGGAATATTGCACAATGGGCGAAAGCCTGAT | 5015 | 0.4724931716963871 | No Hit |
| CCTACGGGCGGCAGCAGTAGGGAATCTTCCGCAATGGGCGAAAGCCTGAC | 3845 | 0.3622604676316268 | No Hit |
| CCTACGGGAGGCTGCAGTAGGGAATCTTCCGCAATGGGCGAAAGCCTGAC | 2713 | 0.2556079710493117 | No Hit |
| CCTACGGGTGGCTGCAGTAGGGAATCTTCCGCAATGGGCGAAAGCCTGAC | 2337 | 0.2201827601703802 | No Hit |
| CCTACGGGGGGCTGCAGTAGGGAATCTTCCGCAATGGGCGAAAGCCTGAC | 1975 | 0.18607657310077058 | No Hit |
| CCTACGGGCGGCTGCAGTAGGGAATCTTCCGCAATGGGCGAAAGCCTGAC | 1549 | 0.14594056290283222 | No Hit |

## Adapter Content

## Kmer Content

| Sequence | Count | PValue | Obs/Exp Max | Max Obs/Exp Position |
| --- | --- | --- | --- | --- |
| ATTCGAT | 25 | 5.002221E-10 | 298.0069 | 295 |
| GAGAGAG | 10 | 8.2116865E-4 | 298.00687 | 295 |
| GGTGCTA | 15 | 6.9529306E-6 | 298.00687 | 295 |
| ATTTGTG | 20 | 5.8795194E-8 | 298.00687 | 295 |
| ATTTGAG | 10 | 8.2116865E-4 | 298.00687 | 295 |
| AGAGCGG | 10 | 8.2116865E-4 | 298.00687 | 295 |
| AGTGCAG | 21220 | 0.0 | 295.61948 | 295 |
| CCTACCG | 15 | 7.3272604E-6 | 294.11807 | 1 |
| GGGACAG | 30 | 5.456968E-12 | 294.11807 | 8 |
| CTCGGTC | 30 | 5.456968E-12 | 294.11807 | 1 |
| ACGTCAT | 25 | 5.4023985E-10 | 294.11807 | 3 |
| GGACAGC | 30 | 5.456968E-12 | 294.11807 | 9 |
| TCGGTCA | 30 | 5.456968E-12 | 294.11807 | 2 |
| TACGGAT | 20 | 6.277696E-8 | 294.11804 | 3 |
| CTAGGCT | 35 | 0.0 | 294.11804 | 1 |
| AGTACAG | 1880 | 0.0 | 294.044 | 295 |
| CCTACGG | 103790 | 0.0 | 293.93387 | 1 |
| CTACGGG | 105155 | 0.0 | 293.36285 | 2 |
| TACGGGA | 30710 | 0.0 | 293.35187 | 3 |
| ACGGGAG | 30685 | 0.0 | 293.35126 | 4 |

Produced by FastQC (version 0.11.7)
