# Supplementary material for: Assessment of airborne bacteria from a public health institution in Mexico City
Source: PLOS Glob Public Health. 2024 Nov 7;4(11):e0003672. doi: 10.1371/journal.pgph.0003672 (PMC11542838; doi:10.1371/journal.pgph.0003672)
Supplement: S1 Text — (ZIP) [file pgph.0003672.s001.zip › Hospital_16S_QC/21022023_BP1D1_16S_S12_L001_R1_001_fastqc.html]

21022023\_BP1D1\_16S\_S12\_L001\_R1\_001.fastq.gz FastQC Report 

FastQC Report

Tue 14 Mar 2023  
21022023\_BP1D1\_16S\_S12\_L001\_R1\_001.fastq.gz

## Summary

- Basic Statistics
- Per base sequence quality
- Per tile sequence quality
- Per sequence quality scores
- Per base sequence content
- Per sequence GC content
- Per base N content
- Sequence Length Distribution
- Sequence Duplication Levels
- Overrepresented sequences
- Adapter Content
- Kmer Content

## Basic Statistics

| Measure | Value |
| --- | --- |
| Filename | 21022023\_BP1D1\_16S\_S12\_L001\_R1\_001.fastq.gz |
| File type | Conventional base calls |
| Encoding | Sanger / Illumina 1.9 |
| Total Sequences | 902763 |
| Sequences flagged as poor quality | 0 |
| Sequence length | 35-301 |
| %GC | 53 |

## Per base sequence quality

## Per tile sequence quality

## Per sequence quality scores

## Per base sequence content

## Per sequence GC content

## Per base N content

## Sequence Length Distribution

## Sequence Duplication Levels

## Overrepresented sequences

| Sequence | Count | Percentage | Possible Source |
| --- | --- | --- | --- |
| CCTACGGGAGGCAGCAGTAGGGAATCTTCCGCAATGGACGAAAGTCTGAC | 61465 | 6.808542219829568 | No Hit |
| CCTACGGGTGGCAGCAGTAGGGAATCTTCCGCAATGGACGAAAGTCTGAC | 59898 | 6.634963993872146 | No Hit |
| CCTACGGGAGGCAGCAGTAGGGAATCTTCCGCAATGGGCGAAAGCCTGAC | 58104 | 6.436240740925358 | No Hit |
| CCTACGGGTGGCAGCAGTAGGGAATCTTCCGCAATGGGCGAAAGCCTGAC | 57686 | 6.389938444530846 | No Hit |
| CCTACGGGGGGCAGCAGTAGGGAATCTTCCGCAATGGACGAAAGTCTGAC | 51957 | 5.755331133420399 | No Hit |
| CCTACGGGGGGCAGCAGTAGGGAATCTTCCGCAATGGGCGAAAGCCTGAC | 50286 | 5.570232718886352 | No Hit |
| CCTACGGGCGGCAGCAGTAGGGAATCTTCCGCAATGGACGAAAGTCTGAC | 38843 | 4.302679662325549 | No Hit |
| CCTACGGGCGGCAGCAGTAGGGAATCTTCCGCAATGGGCGAAAGCCTGAC | 37275 | 4.128990665324122 | No Hit |
| CCTACGGGAGGCAGCAGTGGGGAATATTGGACAATGGGGGGAACCCTGAT | 28016 | 3.1033615688724505 | No Hit |
| CCTACGGGTGGCAGCAGTGGGGAATATTGGACAATGGGGGGAACCCTGAT | 27226 | 3.0158524441077006 | No Hit |
| CCTACGGGAGGCTGCAGTAGGGAATCTTCCGCAATGGACGAAAGTCTGAC | 24145 | 2.6745668575251758 | No Hit |
| CCTACGGGGGGCAGCAGTGGGGAATATTGGACAATGGGGGGAACCCTGAT | 24004 | 2.658948140320328 | No Hit |
| CCTACGGGAGGCTGCAGTAGGGAATCTTCCGCAATGGGCGAAAGCCTGAC | 23042 | 2.5523863959865434 | No Hit |
| CCTACGGGTGGCTGCAGTAGGGAATCTTCCGCAATGGACGAAAGTCTGAC | 19990 | 2.2143131696801928 | No Hit |
| CCTACGGGTGGCTGCAGTAGGGAATCTTCCGCAATGGGCGAAAGCCTGAC | 18678 | 2.068981559944304 | No Hit |
| CCTACGGGCGGCAGCAGTGGGGAATATTGGACAATGGGGGGAACCCTGAT | 18270 | 2.0237869739898513 | No Hit |
| CCTACGGGAGGCTGCAGTGGGGAATATTGGACAATGGGGGGAACCCTGAT | 17377 | 1.9248684316924818 | No Hit |
| CCTACGGGGGGCTGCAGTAGGGAATCTTCCGCAATGGACGAAAGTCTGAC | 16964 | 1.8791199905179987 | No Hit |
| CCTACGGGGGGCTGCAGTAGGGAATCTTCCGCAATGGGCGAAAGCCTGAC | 15944 | 1.7661335256318658 | No Hit |
| CCTACGGGTGGCTGCAGTGGGGAATATTGGACAATGGGGGGAACCCTGAT | 15202 | 1.6839414109794042 | No Hit |
| CCTACGGGCGGCTGCAGTAGGGAATCTTCCGCAATGGACGAAAGTCTGAC | 13114 | 1.45265147109485 | No Hit |
| CCTACGGGGGGCTGCAGTGGGGAATATTGGACAATGGGGGGAACCCTGAT | 12160 | 1.3469758951131139 | No Hit |
| CCTACGGGCGGCTGCAGTAGGGAATCTTCCGCAATGGGCGAAAGCCTGAC | 12156 | 1.3465328109370898 | No Hit |
| CCTACGGGCGGCTGCAGTGGGGAATATTGGACAATGGGGGGAACCCTGAT | 9684 | 1.0727067901542267 | No Hit |
| CCTACGGGTGGCAGCAGTGGGGAATATTGCACAATGGGCGCAAGCCTGAT | 8100 | 0.8972454564487025 | No Hit |
| CCTACGGGAGGCAGCAGTGGGGAATATTGCACAATGGGCGCAAGCCTGAT | 8068 | 0.89370078304051 | No Hit |
| CCTACGGGGGGCAGCAGTGGGGAATATTGCACAATGGGCGCAAGCCTGAT | 7450 | 0.8252442778447941 | No Hit |
| CCTACGGGAGGCAGCAGTAGGGAATCTTCCACAATGGGCGAAAGCCTGAT | 7315 | 0.8102901869039826 | No Hit |
| CCTACGGGTGGCAGCAGTAGGGAATCTTCCACAATGGGCGAAAGCCTGAT | 7185 | 0.7958899511832009 | No Hit |
| CCTACGGGGGGCAGCAGTAGGGAATCTTCCACAATGGGCGAAAGCCTGAT | 6522 | 0.7224487490072146 | No Hit |
| CCTACGGGAGGCTGCAGTGGGGAATATTGCACAATGGGCGCAAGCCTGAT | 5392 | 0.5972774692804202 | No Hit |
| CCTACGGGCGGCAGCAGTGGGGAATATTGCACAATGGGCGCAAGCCTGAT | 5318 | 0.5890804120239752 | No Hit |
| CCTACGGGAGGCAGCAGTAGGGAATCTTCCACAATGGACGAAAGTCTGAT | 4824 | 0.5343595162850051 | No Hit |
| CCTACGGGCGGCAGCAGTAGGGAATCTTCCACAATGGGCGAAAGCCTGAT | 4730 | 0.5239470381484398 | No Hit |
| CCTACGGGTGGCTGCAGTGGGGAATATTGCACAATGGGCGCAAGCCTGAT | 4711 | 0.5218423883123255 | No Hit |
| CCTACGGGTGGCAGCAGTAGGGAATCTTCCACAATGGACGAAAGTCTGAT | 4666 | 0.5168576913320551 | No Hit |
| CCTACGGGGGGCAGCAGTAGGGAATCTTCCACAATGGACGAAAGTCTGAT | 4191 | 0.46424144542919904 | No Hit |
| CCTACGGGAGGCAGCAGTGGGGAATATTGCACAATGGGCGAAAGCCTGAT | 4015 | 0.44474574168414077 | No Hit |
| CCTACGGGTGGCAGCAGTGGGGAATATTGCACAATGGGCGAAAGCCTGAT | 4008 | 0.44397034437609867 | No Hit |
| CCTACGGGGGGCTGCAGTGGGGAATATTGCACAATGGGCGCAAGCCTGAT | 3956 | 0.4382102500877861 | No Hit |
| CCTACGGGGGGCAGCAGTGGGGAATATTGCACAATGGGCGAAAGCCTGAT | 3681 | 0.4077482129861326 | No Hit |
| CCTACGGGCGGCAGCAGTAGGGAATCTTCCACAATGGACGAAAGTCTGAT | 3160 | 0.350036499059 | No Hit |
| CCTACGGGCGGCTGCAGTGGGGAATATTGCACAATGGGCGCAAGCCTGAT | 3103 | 0.3437225495506573 | No Hit |
| CCTACGGGAGGCTGCAGTAGGGAATCTTCCACAATGGGCGAAAGCCTGAT | 2978 | 0.3298761690499057 | No Hit |
| CCTACGGGCGGCAGCAGTGGGGAATATTGCACAATGGGCGAAAGCCTGAT | 2698 | 0.29886027672822213 | No Hit |
| CCTACGGGTGGCTGCAGTAGGGAATCTTCCACAATGGGCGAAAGCCTGAT | 2668 | 0.29553714540804177 | No Hit |
| CCTACGGGAGGCTGCAGTGGGGAATATTGCACAATGGGCGAAAGCCTGAT | 2636 | 0.29199247199984935 | No Hit |
| CCTACGGGTGGCTGCAGTGGGGAATATTGCACAATGGGCGAAAGCCTGAT | 2350 | 0.2603119534141297 | No Hit |
| CCTACGGGGGGCTGCAGTAGGGAATCTTCCACAATGGGCGAAAGCCTGAT | 2163 | 0.23959776818500536 | No Hit |
| CCTACGGGAGGCTGCAGTAGGGAATCTTCCACAATGGACGAAAGTCTGAT | 1919 | 0.2125696334475383 | No Hit |
| CCTACGGGGGGCTGCAGTGGGGAATATTGCACAATGGGCGAAAGCCTGAT | 1898 | 0.21024344152341204 | No Hit |
| CCTACGGGCGGCTGCAGTAGGGAATCTTCCACAATGGGCGAAAGCCTGAT | 1676 | 0.1856522697540772 | No Hit |
| CCTACGGGTGGCTGCAGTAGGGAATCTTCCACAATGGACGAAAGTCTGAT | 1650 | 0.18277222260992088 | No Hit |
| CCTACGGGCGGCTGCAGTGGGGAATATTGCACAATGGGCGAAAGCCTGAT | 1485 | 0.16449500034892878 | No Hit |
| CCTACGGGGGGCTGCAGTAGGGAATCTTCCACAATGGACGAAAGTCTGAT | 1304 | 0.1444454413838405 | No Hit |
| CTTGGTCATTTAGAGGAAGTAAAAGTCGTAACAAGGTTTCCGTAGGTGAA | 1144 | 0.12672207434287847 | No Hit |
| GCTACGGGAGGCAGCAGTAGGGAATCTTCCGCAATGGACGAAAGTCTGAC | 1102 | 0.12206969049462595 | No Hit |
| CCTACGGGCGGCTGCAGTAGGGAATCTTCCACAATGGACGAAAGTCTGAT | 1067 | 0.1181927039544155 | No Hit |
| GCTACGGGTGGCAGCAGTAGGGAATCTTCCGCAATGGACGAAAGTCTGAC | 1058 | 0.11719576455836139 | No Hit |
| GCTACGGGGGGCAGCAGTAGGGAATCTTCCGCAATGGACGAAAGTCTGAC | 983 | 0.10888793625791043 | No Hit |
| CCTACGGGAGGCAGCAGTAGGGAATCTTCCGCAATGGGCGCAAGCCTGAC | 964 | 0.1067832864217962 | No Hit |
| CCTACGGGTGGCAGCAGTAGGGAATCTTCCGCAATGGGCGCAAGCCTGAC | 955 | 0.10578634702574209 | No Hit |

## Adapter Content

## Kmer Content

| Sequence | Count | PValue | Obs/Exp Max | Max Obs/Exp Position |
| --- | --- | --- | --- | --- |
| CATGGCA | 10 | 8.2825375E-4 | 297.15082 | 295 |
| GAATATG | 15 | 7.0328915E-6 | 297.15082 | 295 |
| AGTATAG | 60 | 0.0 | 297.15082 | 295 |
| AGTGCAG | 5870 | 0.0 | 295.37906 | 295 |
| CCTACTG | 20 | 6.278606E-8 | 294.10608 | 1 |
| TCGGGAG | 10 | 8.542014E-4 | 294.10608 | 3 |
| TACAGGC | 10 | 8.542014E-4 | 294.10608 | 3 |
| ACGAGGG | 10 | 8.542014E-4 | 294.10608 | 4 |
| CATTTAG | 285 | 0.0 | 294.10608 | 7 |
| GAGGGCT | 10 | 8.542014E-4 | 294.10608 | 7 |
| TTTAGAG | 345 | 0.0 | 294.10605 | 9 |
| CTATGGG | 15 | 7.3280917E-6 | 294.10605 | 2 |
| GAGTGGC | 15 | 7.3280917E-6 | 294.10605 | 6 |
| GGGTGAC | 30 | 5.456968E-12 | 294.10605 | 6 |
| ATGGCAG | 15 | 7.3280917E-6 | 294.10605 | 8 |
| CCTACGA | 45 | 0.0 | 294.10605 | 1 |
| TAGTTAT | 25 | 5.4023985E-10 | 294.10605 | 3 |
| GAGACAG | 30 | 5.456968E-12 | 294.10605 | 8 |
| CTCGGTC | 70 | 0.0 | 294.10605 | 1 |
| GATGGCA | 15 | 7.3280917E-6 | 294.10605 | 7 |

Produced by FastQC (version 0.11.7)
