# Supplementary material for: Assessment of airborne bacteria from a public health institution in Mexico City
Source: PLOS Glob Public Health. 2024 Nov 7;4(11):e0003672. doi: 10.1371/journal.pgph.0003672 (PMC11542838; doi:10.1371/journal.pgph.0003672)
Supplement: S1 Text — (ZIP) [file pgph.0003672.s001.zip › Hospital_16S_QC/21022023_BP1D1_16S_S12_L001_R2_001_fastqc.html]

21022023\_BP1D1\_16S\_S12\_L001\_R2\_001.fastq.gz FastQC Report 

FastQC Report

Tue 14 Mar 2023  
21022023\_BP1D1\_16S\_S12\_L001\_R2\_001.fastq.gz

## Summary

- Basic Statistics
- Per base sequence quality
- Per tile sequence quality
- Per sequence quality scores
- Per base sequence content
- Per sequence GC content
- Per base N content
- Sequence Length Distribution
- Sequence Duplication Levels
- Overrepresented sequences
- Adapter Content
- Kmer Content

## Basic Statistics

| Measure | Value |
| --- | --- |
| Filename | 21022023\_BP1D1\_16S\_S12\_L001\_R2\_001.fastq.gz |
| File type | Conventional base calls |
| Encoding | Sanger / Illumina 1.9 |
| Total Sequences | 902763 |
| Sequences flagged as poor quality | 0 |
| Sequence length | 35-301 |
| %GC | 53 |

## Per base sequence quality

## Per tile sequence quality

## Per sequence quality scores

## Per base sequence content

## Per sequence GC content

## Per base N content

## Sequence Length Distribution

## Sequence Duplication Levels

## Overrepresented sequences

| Sequence | Count | Percentage | Possible Source |
| --- | --- | --- | --- |
| GACTACTGGGGTATCTAATCCTGTTTGATCCCCACGCTTTCGCACATCAG | 39544 | 4.380330164173764 | No Hit |
| GACTACTAGGGTATCTAATCCTGTTTGATCCCCACGCTTTCGCACATCAG | 34789 | 3.853613849925174 | No Hit |
| GACTACAGGGGTATCTAATCCTGTTTGATCCCCACGCTTTCGCACATCAG | 34189 | 3.7871512235215663 | No Hit |
| GACTACTCGGGTATCTAATCCTGTTTGATCCCCACGCTTTCGCACATCAG | 33034 | 3.659210667694622 | No Hit |
| GACTACCGGGGTATCTAATCCTGTTTGATCCCCACGCTTTCGCACATCAG | 31682 | 3.509448216198493 | No Hit |
| GACTACCAGGGTATCTAATCCTGTTTGATCCCCACGCTTTCGCACATCAG | 31528 | 3.4923894754215667 | No Hit |
| GACTACAAGGGTATCTAATCCTGTTTGATCCCCACGCTTTCGCACATCAG | 31129 | 3.4481918288631683 | No Hit |
| GACTACTGGGGTATCTAATCCTGTTCGCTCCCCACGCTTTCGCTCCTCAG | 30131 | 3.337642326945167 | No Hit |
| GACTACACGGGTATCTAATCCTGTTTGATCCCCACGCTTTCGCACATCAG | 27787 | 3.0779949997950737 | No Hit |
| GACTACCCGGGTATCTAATCCTGTTTGATCCCCACGCTTTCGCACATCAG | 26898 | 2.9795195416737283 | No Hit |
| GACTACAGGGGTATCTAATCCTGTTCGCTCCCCACGCTTTCGCTCCTCAG | 26489 | 2.934214184675269 | No Hit |
| GACTACTAGGGTATCTAATCCTGTTCGCTCCCCACGCTTTCGCTCCTCAG | 26186 | 2.9006505583414475 | No Hit |
| GACTACTCGGGTATCTAATCCTGTTCGCTCCCCACGCTTTCGCTCCTCAG | 25139 | 2.784673275267152 | No Hit |
| GACTACCGGGGTATCTAATCCTGTTCGCTCCCCACGCTTTCGCTCCTCAG | 24310 | 2.692844079786168 | No Hit |
| GACTACCAGGGTATCTAATCCTGTTCGCTCCCCACGCTTTCGCTCCTCAG | 24151 | 2.6752314837892115 | No Hit |
| GACTACAAGGGTATCTAATCCTGTTCGCTCCCCACGCTTTCGCTCCTCAG | 23722 | 2.627710705910632 | No Hit |
| GACTACACGGGTATCTAATCCTGTTCGCTCCCCACGCTTTCGCTCCTCAG | 21499 | 2.381466675085266 | No Hit |
| GACTACTGGGGTATCTAATCCTGTTTGCTCCCCATGCTTTCGCACCTCAG | 21496 | 2.381134361953248 | No Hit |
| GACTACCCGGGTATCTAATCCTGTTCGCTCCCCACGCTTTCGCTCCTCAG | 20837 | 2.3081362439532858 | No Hit |
| GACTACTAGGGTATCTAATCCTGTTTGCTCCCCATGCTTTCGCACCTCAG | 18922 | 2.0960096946817717 | No Hit |
| GACTACAGGGGTATCTAATCCTGTTTGCTCCCCATGCTTTCGCACCTCAG | 18378 | 2.0357502467425004 | No Hit |
| GACTACTCGGGTATCTAATCCTGTTTGCTCCCCATGCTTTCGCACCTCAG | 17842 | 1.976376967155278 | No Hit |
| GACTACAAGGGTATCTAATCCTGTTTGCTCCCCATGCTTTCGCACCTCAG | 16892 | 1.8711444753495658 | No Hit |
| GACTACCAGGGTATCTAATCCTGTTTGCTCCCCATGCTTTCGCACCTCAG | 16863 | 1.8679321150733914 | No Hit |
| GACTACCGGGGTATCTAATCCTGTTTGCTCCCCATGCTTTCGCACCTCAG | 16692 | 1.8489902665483633 | No Hit |
| GACTACACGGGTATCTAATCCTGTTTGCTCCCCATGCTTTCGCACCTCAG | 14853 | 1.645282316621306 | No Hit |
| GACTACCCGGGTATCTAATCCTGTTTGCTCCCCATGCTTTCGCACCTCAG | 14585 | 1.6155956768276944 | No Hit |
| GACTACTGGGGTATCTAATCCTGTTTGCTCCCCACGCTTTCGCGCCTCAG | 14501 | 1.6062909091311897 | No Hit |
| GACTACTAGGGTATCTAATCCTGTTTGCTCCCCACGCTTTCGCGCCTCAG | 12748 | 1.4121092689886494 | No Hit |
| GACTACTCGGGTATCTAATCCTGTTTGCTCCCCACGCTTTCGCGCCTCAG | 12587 | 1.3942751309036814 | No Hit |
| GACTACAGGGGTATCTAATCCTGTTTGCTCCCCACGCTTTCGCGCCTCAG | 12462 | 1.3804287504029296 | No Hit |
| GACTACCGGGGTATCTAATCCTGTTTGCTCCCCACGCTTTCGCGCCTCAG | 11765 | 1.303221332730739 | No Hit |
| GACTACCAGGGTATCTAATCCTGTTTGCTCCCCACGCTTTCGCGCCTCAG | 11654 | 1.2909257468460715 | No Hit |
| GACTACAAGGGTATCTAATCCTGTTTGCTCCCCACGCTTTCGCGCCTCAG | 11453 | 1.268660767000863 | No Hit |
| GACTACACGGGTATCTAATCCTGTTTGCTCCCCACGCTTTCGCGCCTCAG | 10254 | 1.1358462852376536 | No Hit |
| GACTACCCGGGTATCTAATCCTGTTTGCTCCCCACGCTTTCGCGCCTCAG | 9929 | 1.0998456959356997 | No Hit |
| GACTACTGGGGTATCTAATCCTGTTCGCTCCCCATGCTTTCGCTCCTCAG | 6931 | 0.7677541060056737 | No Hit |
| GACTACTAGGGTATCTAATCCTGTTCGCTCCCCATGCTTTCGCTCCTCAG | 6238 | 0.6909897725095069 | No Hit |
| GACTACAGGGGTATCTAATCCTGTTCGCTCCCCATGCTTTCGCTCCTCAG | 6051 | 0.6702755872803825 | No Hit |
| GACTACTCGGGTATCTAATCCTGTTCGCTCCCCATGCTTTCGCTCCTCAG | 5876 | 0.6508906545793304 | No Hit |
| GACTACCGGGGTATCTAATCCTGTTCGCTCCCCATGCTTTCGCTCCTCAG | 5787 | 0.6410320316627952 | No Hit |
| GACTACAAGGGTATCTAATCCTGTTCGCTCCCCATGCTTTCGCTCCTCAG | 5755 | 0.6374873582546028 | No Hit |
| GACTACCAGGGTATCTAATCCTGTTCGCTCCCCATGCTTTCGCTCCTCAG | 5691 | 0.6303980114382179 | No Hit |
| GACTACACGGGTATCTAATCCTGTTCGCTCCCCATGCTTTCGCTCCTCAG | 5019 | 0.5559598698661775 | No Hit |
| GACTACCCGGGTATCTAATCCTGTTCGCTCCCCATGCTTTCGCTCCTCAG | 4977 | 0.551307486017925 | No Hit |

## Adapter Content

## Kmer Content

| Sequence | Count | PValue | Obs/Exp Max | Max Obs/Exp Position |
| --- | --- | --- | --- | --- |
| GTTCTCG | 5 | 5.2212767E-5 | 12510.723 | 295 |
| GGTCGCG | 5 | 5.2212767E-5 | 12510.723 | 295 |
| ATAGCCG | 10 | 1.10758265E-8 | 12510.723 | 295 |
| GTTAGTG | 10 | 1.10758265E-8 | 12510.723 | 295 |
| GTTAGGT | 5 | 5.2212767E-5 | 12510.723 | 295 |
| ATTCGAT | 5 | 5.2212767E-5 | 12510.723 | 295 |
| GTTAGAA | 5 | 5.2212767E-5 | 12510.723 | 295 |
| TCTAGCG | 5 | 5.2212767E-5 | 12510.723 | 295 |
| CGTAGCG | 5 | 5.2212767E-5 | 12510.723 | 295 |
| GGTATGG | 5 | 5.2212767E-5 | 12510.723 | 295 |
| CTTTGAT | 5 | 5.2212767E-5 | 12510.723 | 295 |
| CGTACGA | 5 | 5.2212767E-5 | 12510.723 | 295 |
| ATTGGAA | 5 | 5.2212767E-5 | 12510.723 | 295 |
| TGTAGCG | 5 | 5.2212767E-5 | 12510.723 | 295 |
| GTAAGTG | 5 | 5.2212767E-5 | 12510.723 | 295 |
| CTTAGCA | 5 | 5.2212767E-5 | 12510.723 | 295 |
| GGTAGCG | 10 | 1.10758265E-8 | 12510.723 | 295 |
| GTTATCG | 30 | 0.0 | 12510.722 | 295 |
| GTTAGCG | 840 | 0.0 | 12361.784 | 295 |
| GTTAGGG | 85 | 0.0 | 11774.797 | 295 |

Produced by FastQC (version 0.11.7)
