# Supplementary material for: Assessment of airborne bacteria from a public health institution in Mexico City
Source: PLOS Glob Public Health. 2024 Nov 7;4(11):e0003672. doi: 10.1371/journal.pgph.0003672 (PMC11542838; doi:10.1371/journal.pgph.0003672)
Supplement: S1 Text — (ZIP) [file pgph.0003672.s001.zip › Hospital_16S_QC/21022023_BP1D2_16S_S13_L001_R1_001_fastqc.html]

21022023\_BP1D2\_16S\_S13\_L001\_R1\_001.fastq.gz FastQC Report 

FastQC Report

Tue 14 Mar 2023  
21022023\_BP1D2\_16S\_S13\_L001\_R1\_001.fastq.gz

## Summary

- Basic Statistics
- Per base sequence quality
- Per tile sequence quality
- Per sequence quality scores
- Per base sequence content
- Per sequence GC content
- Per base N content
- Sequence Length Distribution
- Sequence Duplication Levels
- Overrepresented sequences
- Adapter Content
- Kmer Content

## Basic Statistics

| Measure | Value |
| --- | --- |
| Filename | 21022023\_BP1D2\_16S\_S13\_L001\_R1\_001.fastq.gz |
| File type | Conventional base calls |
| Encoding | Sanger / Illumina 1.9 |
| Total Sequences | 880998 |
| Sequences flagged as poor quality | 0 |
| Sequence length | 35-301 |
| %GC | 52 |

## Per base sequence quality

## Per tile sequence quality

## Per sequence quality scores

## Per base sequence content

## Per sequence GC content

## Per base N content

## Sequence Length Distribution

## Sequence Duplication Levels

## Overrepresented sequences

| Sequence | Count | Percentage | Possible Source |
| --- | --- | --- | --- |
| CCTACGGGAGGCAGCAGTAGGGAATCTTCCGCAATGGGCGAAAGCCTGAC | 84196 | 9.556888891915758 | No Hit |
| CCTACGGGTGGCAGCAGTAGGGAATCTTCCGCAATGGGCGAAAGCCTGAC | 82749 | 9.392643343117692 | No Hit |
| CCTACGGGGGGCAGCAGTAGGGAATCTTCCGCAATGGGCGAAAGCCTGAC | 71210 | 8.082878735252521 | No Hit |
| CCTACGGGCGGCAGCAGTAGGGAATCTTCCGCAATGGGCGAAAGCCTGAC | 54665 | 6.2048949032801435 | No Hit |
| CCTACGGGAGGCAGCAGTGGGGAATATTGGACAATGGGGGGAACCCTGAT | 40499 | 4.596945736539697 | No Hit |
| CCTACGGGTGGCAGCAGTGGGGAATATTGGACAATGGGGGGAACCCTGAT | 38854 | 4.410225675881216 | No Hit |
| CCTACGGGGGGCAGCAGTGGGGAATATTGGACAATGGGGGGAACCCTGAT | 34481 | 3.9138567851459367 | No Hit |
| CCTACGGGAGGCTGCAGTAGGGAATCTTCCGCAATGGGCGAAAGCCTGAC | 33939 | 3.852335646618948 | No Hit |
| CCTACGGGTGGCTGCAGTAGGGAATCTTCCGCAATGGGCGAAAGCCTGAC | 27897 | 3.1665225119693803 | No Hit |
| CCTACGGGCGGCAGCAGTGGGGAATATTGGACAATGGGGGGAACCCTGAT | 26887 | 3.051879799954143 | No Hit |
| CCTACGGGAGGCTGCAGTGGGGAATATTGGACAATGGGGGGAACCCTGAT | 25764 | 2.924410725109478 | No Hit |
| CCTACGGGGGGCTGCAGTAGGGAATCTTCCGCAATGGGCGAAAGCCTGAC | 23607 | 2.6795747549937685 | No Hit |
| CCTACGGGTGGCTGCAGTGGGGAATATTGGACAATGGGGGGAACCCTGAT | 22089 | 2.5072701640639368 | No Hit |
| CCTACGGGCGGCTGCAGTAGGGAATCTTCCGCAATGGGCGAAAGCCTGAC | 18009 | 2.0441590105766414 | No Hit |
| CCTACGGGGGGCTGCAGTGGGGAATATTGGACAATGGGGGGAACCCTGAT | 17568 | 1.9941021432511765 | No Hit |
| CCTACGGGAGGCAGCAGTAGGGAATCTTCGGCAATGGACGAAAGTCTGAC | 16238 | 1.843136987825171 | No Hit |
| CCTACGGGTGGCAGCAGTAGGGAATCTTCGGCAATGGACGAAAGTCTGAC | 15840 | 1.797960948833028 | No Hit |
| CCTACGGGCGGCTGCAGTGGGGAATATTGGACAATGGGGGGAACCCTGAT | 14104 | 1.6009116933296104 | No Hit |
| CCTACGGGGGGCAGCAGTAGGGAATCTTCGGCAATGGACGAAAGTCTGAC | 14007 | 1.5899014526707211 | No Hit |
| CCTACGGGCGGCAGCAGTAGGGAATCTTCGGCAATGGACGAAAGTCTGAC | 10600 | 1.203180937981698 | No Hit |
| CCTACGGGAGGCAGCAGTGGGGAATATTGCACAATGGGCGCAAGCCTGAT | 7616 | 0.8644741531762842 | No Hit |
| CCTACGGGTGGCAGCAGTGGGGAATATTGCACAATGGGCGCAAGCCTGAT | 7542 | 0.856074588137544 | No Hit |
| CCTACGGGGGGCAGCAGTGGGGAATATTGCACAATGGGCGCAAGCCTGAT | 6929 | 0.7864944074787911 | No Hit |
| CCTACGGGAGGCTGCAGTAGGGAATCTTCGGCAATGGACGAAAGTCTGAC | 6585 | 0.7474477808122153 | No Hit |
| CCTACGGGTGGCTGCAGTAGGGAATCTTCGGCAATGGACGAAAGTCTGAC | 5501 | 0.6244055037582378 | No Hit |
| CCTACGGGCGGCAGCAGTGGGGAATATTGCACAATGGGCGCAAGCCTGAT | 5177 | 0.587629029804835 | No Hit |
| CCTACGGGAGGCTGCAGTGGGGAATATTGCACAATGGGCGCAAGCCTGAT | 5131 | 0.582407678564537 | No Hit |
| CCTACGGGAGGCAGCAGTGGGGAATATTGCACAATGGGCGAAAGCCTGAT | 5043 | 0.5724190066265757 | No Hit |
| CCTACGGGTGGCAGCAGTGGGGAATATTGCACAATGGGCGAAAGCCTGAT | 4948 | 0.5616357812390039 | No Hit |
| CCTACGGGGGGCTGCAGTAGGGAATCTTCGGCAATGGACGAAAGTCTGAC | 4644 | 0.5271294599987741 | No Hit |
| CCTACGGGTGGCTGCAGTGGGGAATATTGCACAATGGGCGCAAGCCTGAT | 4442 | 0.5042009175957267 | No Hit |
| CCTACGGGGGGCAGCAGTGGGGAATATTGCACAATGGGCGAAAGCCTGAT | 4334 | 0.49194209294459235 | No Hit |
| CCTACGGGAGGCAGCAGTAGGGAATCTTCCGCAATGGACGAAAGTCTGAC | 3842 | 0.4360963362005362 | No Hit |
| CCTACGGGGGGCTGCAGTGGGGAATATTGCACAATGGGCGCAAGCCTGAT | 3767 | 0.4275832635261374 | No Hit |
| CCTACGGGCGGCTGCAGTAGGGAATCTTCGGCAATGGACGAAAGTCTGAC | 3745 | 0.4250860955416471 | No Hit |
| CCTACGGGTGGCAGCAGTAGGGAATCTTCCACAATGGACGAAAGTCTGAT | 3705 | 0.4205457901153011 | No Hit |
| CCTACGGGTGGCAGCAGTAGGGAATCTTCCGCAATGGACGAAAGTCTGAC | 3698 | 0.4197512366656905 | No Hit |
| CCTACGGGAGGCAGCAGTAGGGAATCTTCCACAATGGACGAAAGTCTGAT | 3687 | 0.41850265267344533 | No Hit |
| CCTACGGGGGGCAGCAGTAGGGAATCTTCCGCAATGGACGAAAGTCTGAC | 3286 | 0.37298609077432643 | No Hit |
| CCTACGGGAGGCTGCAGTGGGGAATATTGCACAATGGGCGAAAGCCTGAT | 3257 | 0.36969436934022554 | No Hit |
| CCTACGGGGGGCAGCAGTAGGGAATCTTCCACAATGGACGAAAGTCTGAT | 3247 | 0.36855929298363904 | No Hit |
| CCTACGGGCGGCAGCAGTGGGGAATATTGCACAATGGGCGAAAGCCTGAT | 3245 | 0.3683322777123217 | No Hit |
| CCTACGGGAGGCAGCAGTGAGGAATATTGGACAATGGGTGAGAGCCTGAT | 3072 | 0.34869545674337515 | No Hit |
| CCTACGGGTGGCTGCAGTGGGGAATATTGCACAATGGGCGAAAGCCTGAT | 3051 | 0.34631179639454346 | No Hit |
| CCTACGGGCGGCTGCAGTGGGGAATATTGCACAATGGGCGCAAGCCTGAT | 2944 | 0.33416647937906785 | No Hit |
| CCTACGGGTGGCAGCAGTGAGGAATATTGGACAATGGGTGAGAGCCTGAT | 2890 | 0.32803706705350066 | No Hit |
| CCTACGGGGGGCAGCAGTGAGGAATATTGGACAATGGGTGAGAGCCTGAT | 2719 | 0.3086272613558714 | No Hit |
| CCTACGGGCGGCAGCAGTAGGGAATCTTCCGCAATGGACGAAAGTCTGAC | 2529 | 0.28706081058072774 | No Hit |
| CCTACGGGCGGCAGCAGTAGGGAATCTTCCACAATGGACGAAAGTCTGAT | 2483 | 0.28183945934042987 | No Hit |
| CCTACGGGGGGCTGCAGTGGGGAATATTGCACAATGGGCGAAAGCCTGAT | 2461 | 0.2793422913559395 | No Hit |
| CCTACGGGAGGCTGCAGTGAGGAATATTGGACAATGGGTGAGAGCCTGAT | 2379 | 0.27003466523193015 | No Hit |
| CCTACGGGCGGCAGCAGTGAGGAATATTGGACAATGGGTGAGAGCCTGAT | 2035 | 0.23098803856535427 | No Hit |
| CCTACGGGTGGCTGCAGTGAGGAATATTGGACAATGGGTGAGAGCCTGAT | 2016 | 0.22883139348783993 | No Hit |
| CCTACGGGCGGCTGCAGTGGGGAATATTGCACAATGGGCGAAAGCCTGAT | 1794 | 0.20363269837161946 | No Hit |
| CCTACGGGAGGCTGCAGTAGGGAATCTTCCACAATGGACGAAAGTCTGAT | 1689 | 0.19171439662746115 | No Hit |
| CCTACGGGGGGCTGCAGTGAGGAATATTGGACAATGGGTGAGAGCCTGAT | 1622 | 0.18410938503833152 | No Hit |
| CCTACGGGAGGCTGCAGTAGGGAATCTTCCGCAATGGACGAAAGTCTGAC | 1536 | 0.17434772837168758 | No Hit |
| CCTACGGGCGGCTGCAGTGAGGAATATTGGACAATGGGTGAGAGCCTGAT | 1367 | 0.1551649379453756 | No Hit |
| CCTACGGGTGGCTGCAGTAGGGAATCTTCCACAATGGACGAAAGTCTGAT | 1359 | 0.1542568768601064 | No Hit |
| CCTACGGGTGGCTGCAGTAGGGAATCTTCCGCAATGGACGAAAGTCTGAC | 1320 | 0.149830079069419 | No Hit |
| CCTACGGGGGGCTGCAGTAGGGAATCTTCCACAATGGACGAAAGTCTGAT | 1143 | 0.12973922755783782 | No Hit |
| CTTGGTCATTTAGAGGAAGTAAAAGTCGTAACAAGGTTTCCGTAGGTGAA | 1093 | 0.12406384577490527 | No Hit |
| CCTACGGGGGGCTGCAGTAGGGAATCTTCCGCAATGGACGAAAGTCTGAC | 1085 | 0.12315578468963606 | No Hit |
| CCTACGGGCGGCTGCAGTAGGGAATCTTCCACAATGGACGAAAGTCTGAT | 916 | 0.10397299426332408 | No Hit |

## Adapter Content

## Kmer Content

| Sequence | Count | PValue | Obs/Exp Max | Max Obs/Exp Position |
| --- | --- | --- | --- | --- |
| AGTCCAG | 15 | 6.7045785E-6 | 300.72992 | 295 |
| ATAGGAA | 25 | 4.7293724E-10 | 300.72992 | 295 |
| GCTTGTG | 15 | 6.7045785E-6 | 300.72992 | 295 |
| AGTATAG | 45 | 0.0 | 300.72992 | 295 |
| GCTGGGT | 10 | 7.9907535E-4 | 300.7299 | 295 |
| GAATATG | 40 | 0.0 | 300.7299 | 295 |
| GATTCGG | 10 | 7.9907535E-4 | 300.7299 | 295 |
| AGTGCAG | 6605 | 0.0 | 297.3151 | 295 |
| CCTACTG | 15 | 7.3262454E-6 | 294.12402 | 1 |
| CTTGGTC | 160 | 0.0 | 294.12402 | 1 |
| CTCGGTC | 30 | 5.456968E-12 | 294.12402 | 1 |
| CATACTC | 10 | 8.5404183E-4 | 294.12402 | 1 |
| GAGTGGC | 10 | 8.54187E-4 | 294.10733 | 6 |
| GGGGACA | 25 | 5.4023985E-10 | 294.10733 | 7 |
| GAGTCAG | 10 | 8.54187E-4 | 294.10733 | 8 |
| GCTATTT | 50 | 0.0 | 294.10733 | 5 |
| TCGGGAG | 10 | 8.54187E-4 | 294.10733 | 3 |
| CGGGGTG | 10 | 8.54187E-4 | 294.10733 | 5 |
| TAGCAGC | 25 | 5.4023985E-10 | 294.10733 | 9 |
| GGGACAG | 25 | 5.4023985E-10 | 294.10733 | 8 |

Produced by FastQC (version 0.11.7)
