# Supplementary material for: Assessment of airborne bacteria from a public health institution in Mexico City
Source: PLOS Glob Public Health. 2024 Nov 7;4(11):e0003672. doi: 10.1371/journal.pgph.0003672 (PMC11542838; doi:10.1371/journal.pgph.0003672)
Supplement: S1 Text — (ZIP) [file pgph.0003672.s001.zip › Hospital_16S_QC/21022023_BP1D2_16S_S13_L001_R2_001_fastqc.html]

21022023\_BP1D2\_16S\_S13\_L001\_R2\_001.fastq.gz FastQC Report 

FastQC Report

Tue 14 Mar 2023  
21022023\_BP1D2\_16S\_S13\_L001\_R2\_001.fastq.gz

## Summary

- Basic Statistics
- Per base sequence quality
- Per tile sequence quality
- Per sequence quality scores
- Per base sequence content
- Per sequence GC content
- Per base N content
- Sequence Length Distribution
- Sequence Duplication Levels
- Overrepresented sequences
- Adapter Content
- Kmer Content

## Basic Statistics

| Measure | Value |
| --- | --- |
| Filename | 21022023\_BP1D2\_16S\_S13\_L001\_R2\_001.fastq.gz |
| File type | Conventional base calls |
| Encoding | Sanger / Illumina 1.9 |
| Total Sequences | 880998 |
| Sequences flagged as poor quality | 0 |
| Sequence length | 35-301 |
| %GC | 52 |

## Per base sequence quality

## Per tile sequence quality

## Per sequence quality scores

## Per base sequence content

## Per sequence GC content

## Per base N content

## Sequence Length Distribution

## Sequence Duplication Levels

## Overrepresented sequences

| Sequence | Count | Percentage | Possible Source |
| --- | --- | --- | --- |
| GACTACTGGGGTATCTAATCCTGTTTGATCCCCACGCTTTCGCACATCAG | 52896 | 6.004099895799991 | No Hit |
| GACTACTAGGGTATCTAATCCTGTTTGATCCCCACGCTTTCGCACATCAG | 46376 | 5.264030111305588 | No Hit |
| GACTACAGGGGTATCTAATCCTGTTTGATCCCCACGCTTTCGCACATCAG | 45557 | 5.171067357701153 | No Hit |
| GACTACTCGGGTATCTAATCCTGTTTGATCCCCACGCTTTCGCACATCAG | 44426 | 5.042690221771219 | No Hit |
| GACTACCAGGGTATCTAATCCTGTTTGATCCCCACGCTTTCGCACATCAG | 42541 | 4.8287283285546625 | No Hit |
| GACTACCGGGGTATCTAATCCTGTTTGATCCCCACGCTTTCGCACATCAG | 42274 | 4.798421789833802 | No Hit |
| GACTACAAGGGTATCTAATCCTGTTTGATCCCCACGCTTTCGCACATCAG | 42028 | 4.770498911461774 | No Hit |
| GACTACACGGGTATCTAATCCTGTTTGATCCCCACGCTTTCGCACATCAG | 37021 | 4.202166179718909 | No Hit |
| GACTACCCGGGTATCTAATCCTGTTTGATCCCCACGCTTTCGCACATCAG | 35868 | 4.071291875804485 | No Hit |
| GACTACTGGGGTATCTAATCCTGTTTGCTCCCCATGCTTTCGCACCTCAG | 32376 | 3.6749232120844773 | No Hit |
| GACTACTAGGGTATCTAATCCTGTTTGCTCCCCATGCTTTCGCACCTCAG | 28155 | 3.1958074819693123 | No Hit |
| GACTACAGGGGTATCTAATCCTGTTTGCTCCCCATGCTTTCGCACCTCAG | 27963 | 3.174014015922851 | No Hit |
| GACTACTCGGGTATCTAATCCTGTTTGCTCCCCATGCTTTCGCACCTCAG | 26857 | 3.0484745708843834 | No Hit |
| GACTACCAGGGTATCTAATCCTGTTTGCTCCCCATGCTTTCGCACCTCAG | 25894 | 2.9391667177451026 | No Hit |
| GACTACAAGGGTATCTAATCCTGTTTGCTCCCCATGCTTTCGCACCTCAG | 25709 | 2.918167805148252 | No Hit |
| GACTACCGGGGTATCTAATCCTGTTTGCTCCCCATGCTTTCGCACCTCAG | 25443 | 2.8879747740630513 | No Hit |
| GACTACACGGGTATCTAATCCTGTTTGCTCCCCATGCTTTCGCACCTCAG | 22667 | 2.5728775774746366 | No Hit |
| GACTACCCGGGTATCTAATCCTGTTTGCTCCCCATGCTTTCGCACCTCAG | 22056 | 2.5035244120872013 | No Hit |
| GACTACTGGGGTATCTAATCCTGTTCGCTCCCCACGCTTTCGAGCCTCAG | 10269 | 1.1656099105786846 | No Hit |
| GACTACAGGGGTATCTAATCCTGTTCGCTCCCCACGCTTTCGAGCCTCAG | 8897 | 1.0098774344550157 | No Hit |
| GACTACTAGGGTATCTAATCCTGTTCGCTCCCCACGCTTTCGAGCCTCAG | 8839 | 1.0032939915868142 | No Hit |
| GACTACTGGGGTATCTAATCCTGTTCGCTCCCCATGCTTTCGCTCCTCAG | 8682 | 0.9854732927884059 | No Hit |
| GACTACTCGGGTATCTAATCCTGTTCGCTCCCCACGCTTTCGAGCCTCAG | 8491 | 0.9637933343776035 | No Hit |
| GACTACCAGGGTATCTAATCCTGTTCGCTCCCCACGCTTTCGAGCCTCAG | 8243 | 0.9356434407342582 | No Hit |
| GACTACAAGGGTATCTAATCCTGTTCGCTCCCCACGCTTTCGAGCCTCAG | 8082 | 0.9173687113932153 | No Hit |
| GACTACCGGGGTATCTAATCCTGTTCGCTCCCCACGCTTTCGAGCCTCAG | 8080 | 0.9171416961218981 | No Hit |
| GACTACTAGGGTATCTAATCCTGTTCGCTCCCCATGCTTTCGCTCCTCAG | 7820 | 0.8876297108506489 | No Hit |
| GACTACAGGGGTATCTAATCCTGTTCGCTCCCCATGCTTTCGCTCCTCAG | 7558 | 0.8578907103080824 | No Hit |
| GACTACTCGGGTATCTAATCCTGTTCGCTCCCCATGCTTTCGCTCCTCAG | 7288 | 0.8272436486802468 | No Hit |
| GACTACCAGGGTATCTAATCCTGTTCGCTCCCCATGCTTTCGCTCCTCAG | 7196 | 0.8168009461996509 | No Hit |
| GACTACACGGGTATCTAATCCTGTTCGCTCCCCACGCTTTCGAGCCTCAG | 7153 | 0.8119201178663289 | No Hit |
| GACTACAAGGGTATCTAATCCTGTTCGCTCCCCATGCTTTCGCTCCTCAG | 7039 | 0.7989802474012427 | No Hit |
| GACTACCCGGGTATCTAATCCTGTTCGCTCCCCACGCTTTCGAGCCTCAG | 7028 | 0.7977316634089975 | No Hit |
| GACTACCGGGGTATCTAATCCTGTTCGCTCCCCATGCTTTCGCTCCTCAG | 6992 | 0.793645388525286 | No Hit |
| GACTACACGGGTATCTAATCCTGTTCGCTCCCCATGCTTTCGCTCCTCAG | 6371 | 0.723157146781264 | No Hit |
| GACTACCCGGGTATCTAATCCTGTTCGCTCCCCATGCTTTCGCTCCTCAG | 6156 | 0.6987530051146541 | No Hit |
| GACTACTGGGGTATCTAATCCTGTTCGCTCCCCACGCTTTCGTCCATCAG | 2087 | 0.23689043561960413 | No Hit |
| GACTACTGGGGTATCTAATCCTGTTTGCTCCCCACGCTTTCGCGCCTCAG | 1928 | 0.21884272154987866 | No Hit |
| GACTACAGGGGTATCTAATCCTGTTTGCTCCCCACGCTTTCGCGCCTCAG | 1756 | 0.19931940821659072 | No Hit |
| GACTACTAGGGTATCTAATCCTGTTCGCTCCCCACGCTTTCGTCCATCAG | 1752 | 0.1988653776739561 | No Hit |
| GACTACAGGGGTATCTAATCCTGTTCGCTCCCCACGCTTTCGTCCATCAG | 1723 | 0.19557365623985526 | No Hit |
| GACTACTAGGGTATCTAATCCTGTTTGCTCCCCACGCTTTCGCGCCTCAG | 1701 | 0.19307648825536494 | No Hit |
| GACTACTGGGGTATCTAATCCTGTTCGCTCCCCACGCTTTCGCGCCTCAG | 1673 | 0.18989827445692273 | No Hit |
| GACTACCAGGGTATCTAATCCTGTTCGCTCCCCACGCTTTCGTCCATCAG | 1650 | 0.18728759883677373 | No Hit |
| GACTACTCGGGTATCTAATCCTGTTCGCTCCCCACGCTTTCGTCCATCAG | 1642 | 0.18637953775150454 | No Hit |
| GACTACTCGGGTATCTAATCCTGTTTGCTCCCCACGCTTTCGCGCCTCAG | 1638 | 0.18592550720886994 | No Hit |
| GACTACCGGGGTATCTAATCCTGTTTGCTCCCCACGCTTTCGCGCCTCAG | 1626 | 0.18456341558096612 | No Hit |
| GACTACAAGGGTATCTAATCCTGTTTGCTCCCCACGCTTTCGCGCCTCAG | 1573 | 0.17854751089105764 | No Hit |
| GACTACCGGGGTATCTAATCCTGTTCGCTCCCCACGCTTTCGTCCATCAG | 1565 | 0.17763944980578844 | No Hit |
| GACTACCAGGGTATCTAATCCTGTTTGCTCCCCACGCTTTCGCGCCTCAG | 1547 | 0.17559631236393272 | No Hit |
| GACTACAAGGGTATCTAATCCTGTTCGCTCCCCACGCTTTCGTCCATCAG | 1525 | 0.1730991443794424 | No Hit |
| GACTACACGGGTATCTAATCCTGTTTGCTCCCCACGCTTTCGCGCCTCAG | 1474 | 0.16731025496085122 | No Hit |
| GACTACACGGGTATCTAATCCTGTTCGCTCCCCACGCTTTCGTCCATCAG | 1441 | 0.16356450298411573 | No Hit |
| GACTACAGGGGTATCTAATCCTGTTCGCTCCCCACGCTTTCGCGCCTCAG | 1402 | 0.15913770519342837 | No Hit |
| GACTACTAGGGTATCTAATCCTGTTCGCTCCCCACGCTTTCGCGCCTCAG | 1352 | 0.15346232341049582 | No Hit |
| GACTACTCGGGTATCTAATCCTGTTCGCTCCCCACGCTTTCGCGCCTCAG | 1350 | 0.15323530813917852 | No Hit |
| GACTACCCGGGTATCTAATCCTGTTCGCTCCCCACGCTTTCGTCCATCAG | 1331 | 0.15107866306166418 | No Hit |
| GACTACCCGGGTATCTAATCCTGTTTGCTCCCCACGCTTTCGCGCCTCAG | 1310 | 0.14869500271283248 | No Hit |
| GACTACAAGGGTATCTAATCCTGTTCGCTCCCCACGCTTTCGCGCCTCAG | 1280 | 0.14528977364307297 | No Hit |
| GACTACCGGGGTATCTAATCCTGTTCGCTCCCCACGCTTTCGCGCCTCAG | 1280 | 0.14528977364307297 | No Hit |
| GACTACCAGGGTATCTAATCCTGTTCGCTCCCCACGCTTTCGCGCCTCAG | 1237 | 0.14040894530975098 | No Hit |
| GACTACACGGGTATCTAATCCTGTTCGCTCCCCACGCTTTCGCGCCTCAG | 1164 | 0.13212288790666948 | No Hit |
| GACTACCCGGGTATCTAATCCTGTTCGCTCCCCACGCTTTCGCGCCTCAG | 1108 | 0.12576646030978506 | No Hit |

## Adapter Content

## Kmer Content

| Sequence | Count | PValue | Obs/Exp Max | Max Obs/Exp Position |
| --- | --- | --- | --- | --- |
| GTTAGGG | 55 | 0.0 | 12941.681 | 295 |
| TTAGACG | 5 | 4.8786456E-5 | 12941.68 | 295 |
| GTTAGAA | 5 | 4.8786456E-5 | 12941.68 | 295 |
| CTGACGA | 5 | 4.8786456E-5 | 12941.68 | 295 |
| TTATCCG | 10 | 1.0000804E-8 | 12941.68 | 295 |
| CATAGCA | 5 | 4.8786456E-5 | 12941.68 | 295 |
| CTTTGCA | 5 | 4.8786456E-5 | 12941.68 | 295 |
| TTTATGG | 5 | 4.8786456E-5 | 12941.68 | 295 |
| TGCTAGA | 5 | 4.8786456E-5 | 12941.68 | 295 |
| TTTGCCG | 10 | 1.0000804E-8 | 12941.68 | 295 |
| ATTATCA | 5 | 4.8786456E-5 | 12941.68 | 295 |
| TAGGAGG | 5 | 4.8786456E-5 | 12941.68 | 295 |
| GTAAGCG | 5 | 4.8786456E-5 | 12941.68 | 295 |
| TATACAG | 5 | 4.8786456E-5 | 12941.68 | 295 |
| CTTAGGG | 5 | 4.8786456E-5 | 12941.68 | 295 |
| GTTATCA | 5 | 4.8786456E-5 | 12941.68 | 295 |
| GGTAGTG | 5 | 4.8786456E-5 | 12941.68 | 295 |
| GATAGAG | 5 | 4.8786456E-5 | 12941.68 | 295 |
| GGTAGCG | 5 | 4.8786456E-5 | 12941.68 | 295 |
| GTTAGCG | 730 | 0.0 | 12853.038 | 295 |

Produced by FastQC (version 0.11.7)
