# Supplementary material for: Assessment of airborne bacteria from a public health institution in Mexico City
Source: PLOS Glob Public Health. 2024 Nov 7;4(11):e0003672. doi: 10.1371/journal.pgph.0003672 (PMC11542838; doi:10.1371/journal.pgph.0003672)
Supplement: S1 Text — (ZIP) [file pgph.0003672.s001.zip › Hospital_16S_QC/21022023_BP1D3_16S_S14_L001_R1_001_fastqc.html]

21022023\_BP1D3\_16S\_S14\_L001\_R1\_001.fastq.gz FastQC Report 

FastQC Report

Tue 14 Mar 2023  
21022023\_BP1D3\_16S\_S14\_L001\_R1\_001.fastq.gz

## Summary

- Basic Statistics
- Per base sequence quality
- Per tile sequence quality
- Per sequence quality scores
- Per base sequence content
- Per sequence GC content
- Per base N content
- Sequence Length Distribution
- Sequence Duplication Levels
- Overrepresented sequences
- Adapter Content
- Kmer Content

## Basic Statistics

| Measure | Value |
| --- | --- |
| Filename | 21022023\_BP1D3\_16S\_S14\_L001\_R1\_001.fastq.gz |
| File type | Conventional base calls |
| Encoding | Sanger / Illumina 1.9 |
| Total Sequences | 914140 |
| Sequences flagged as poor quality | 0 |
| Sequence length | 35-301 |
| %GC | 53 |

## Per base sequence quality

## Per tile sequence quality

## Per sequence quality scores

## Per base sequence content

## Per sequence GC content

## Per base N content

## Sequence Length Distribution

## Sequence Duplication Levels

## Overrepresented sequences

| Sequence | Count | Percentage | Possible Source |
| --- | --- | --- | --- |
| CCTACGGGAGGCAGCAGTAGGGAATCTTCCGCAATGGACGAAAGTCTGAC | 130448 | 14.270024285120442 | No Hit |
| CCTACGGGTGGCAGCAGTAGGGAATCTTCCGCAATGGACGAAAGTCTGAC | 128388 | 14.044675870216816 | No Hit |
| CCTACGGGGGGCAGCAGTAGGGAATCTTCCGCAATGGACGAAAGTCTGAC | 112758 | 12.334872120244164 | No Hit |
| CCTACGGGCGGCAGCAGTAGGGAATCTTCCGCAATGGACGAAAGTCTGAC | 86413 | 9.452928435469403 | No Hit |
| CCTACGGGAGGCTGCAGTAGGGAATCTTCCGCAATGGACGAAAGTCTGAC | 58182 | 6.3646706193799645 | No Hit |
| CCTACGGGTGGCTGCAGTAGGGAATCTTCCGCAATGGACGAAAGTCTGAC | 48581 | 5.3143938565208835 | No Hit |
| CCTACGGGGGGCTGCAGTAGGGAATCTTCCGCAATGGACGAAAGTCTGAC | 40462 | 4.426236681471109 | No Hit |
| CCTACGGGCGGCTGCAGTAGGGAATCTTCCGCAATGGACGAAAGTCTGAC | 31488 | 3.4445489749928893 | No Hit |
| CCTACGGGAGGCAGCAGTGGGGAATATTGGACAATGGGCGAAAGCCTGAT | 11600 | 1.2689522392631325 | No Hit |
| CCTACGGGTGGCAGCAGTGGGGAATATTGGACAATGGGCGAAAGCCTGAT | 11334 | 1.2398538517076159 | No Hit |
| CCTACGGGGGGCAGCAGTGGGGAATATTGGACAATGGGCGAAAGCCTGAT | 10489 | 1.147417244623362 | No Hit |
| CCTACGGGTGGCAGCAGTAGGGAATCTTCCACAATGGGCGAAAGCCTGAT | 10275 | 1.1240072636576455 | No Hit |
| CCTACGGGAGGCAGCAGTAGGGAATCTTCCACAATGGGCGAAAGCCTGAT | 10247 | 1.1209442754939067 | No Hit |
| CCTACGGGGGGCAGCAGTAGGGAATCTTCCACAATGGGCGAAAGCCTGAT | 9247 | 1.0115518410746713 | No Hit |
| CCTACGGGAGGCAGCAGTGGGGAATATTGGACAATGGGGGGAACCCTGAT | 8384 | 0.9171461701708709 | No Hit |
| CCTACGGGAGGCTGCAGTGGGGAATATTGGACAATGGGCGAAAGCCTGAT | 7969 | 0.8717483098868881 | No Hit |
| CCTACGGGTGGCAGCAGTGGGGAATATTGGACAATGGGGGGAACCCTGAT | 7905 | 0.8647471940840571 | No Hit |
| CCTACGGGCGGCAGCAGTGGGGAATATTGGACAATGGGCGAAAGCCTGAT | 7598 | 0.8311637167173518 | No Hit |
| CCTACGGGGGGCAGCAGTGGGGAATATTGGACAATGGGGGGAACCCTGAT | 7353 | 0.8043625702846391 | No Hit |
| CCTACGGGTGGCTGCAGTGGGGAATATTGGACAATGGGCGAAAGCCTGAT | 7145 | 0.7816089439254381 | No Hit |
| CCTACGGGCGGCAGCAGTAGGGAATCTTCCACAATGGGCGAAAGCCTGAT | 7046 | 0.7707790929179338 | No Hit |
| CCTACGGGAGGCTGCAGTGGGGAATATTGGACAATGGGGGGAACCCTGAT | 5948 | 0.6506661999256131 | No Hit |
| CCTACGGGCGGCAGCAGTGGGGAATATTGGACAATGGGGGGAACCCTGAT | 5783 | 0.6326164482464393 | No Hit |
| CCTACGGGGGGCTGCAGTGGGGAATATTGGACAATGGGCGAAAGCCTGAT | 5754 | 0.6294440676482814 | No Hit |
| CCTACGGGAGGCTGCAGTAGGGAATCTTCCACAATGGGCGAAAGCCTGAT | 4970 | 0.5436803990636008 | No Hit |
| CCTACGGGTGGCTGCAGTGGGGAATATTGGACAATGGGGGGAACCCTGAT | 4883 | 0.5341632572691273 | No Hit |
| CCTACGGGCGGCTGCAGTGGGGAATATTGGACAATGGGCGAAAGCCTGAT | 4485 | 0.49062506837027153 | No Hit |
| CCTACGGGGGGCTGCAGTGGGGAATATTGGACAATGGGGGGAACCCTGAT | 4106 | 0.44916533572538125 | No Hit |
| CCTACGGGTGGCTGCAGTAGGGAATCTTCCACAATGGGCGAAAGCCTGAT | 4023 | 0.44008576366858465 | No Hit |
| CCTACGGGAGGCAGCAGTAGGGAATCTTCCGCAATGGGCGAAAGCCTGAC | 3921 | 0.4289277353578227 | No Hit |
| CCTACGGGTGGCAGCAGTAGGGAATCTTCCGCAATGGGCGAAAGCCTGAC | 3840 | 0.4200669481698646 | No Hit |
| CCTACGGGGGGCAGCAGTAGGGAATCTTCCGCAATGGGCGAAAGCCTGAC | 3594 | 0.39315640930273266 | No Hit |
| CCTACGGGGGGCTGCAGTAGGGAATCTTCCACAATGGGCGAAAGCCTGAT | 3346 | 0.3660270855667622 | No Hit |
| CCTACGGGCGGCTGCAGTGGGGAATATTGGACAATGGGGGGAACCCTGAT | 3267 | 0.35738508324764257 | No Hit |
| CCTACGGGCGGCTGCAGTAGGGAATCTTCCACAATGGGCGAAAGCCTGAT | 2726 | 0.29820377622683614 | No Hit |
| CCTACGGGAGGCAGCAGTGGGGAATATTGCACAATGGGCGAAAGCCTGAT | 2680 | 0.2931717242435513 | No Hit |
| CCTACGGGCGGCAGCAGTAGGGAATCTTCCGCAATGGGCGAAAGCCTGAC | 2625 | 0.28715514035049333 | No Hit |
| CCTACGGGTGGCAGCAGTGGGGAATATTGCACAATGGGCGAAAGCCTGAT | 2557 | 0.2797164548099853 | No Hit |
| GCTACGGGAGGCAGCAGTAGGGAATCTTCCGCAATGGACGAAAGTCTGAC | 2510 | 0.27457501039228127 | No Hit |
| CCTACGGGGGGCAGCAGTGGGGAATATTGCACAATGGGCGAAAGCCTGAT | 2347 | 0.2567440435819459 | No Hit |
| GCTACGGGGGGCAGCAGTAGGGAATCTTCCGCAATGGACGAAAGTCTGAC | 2290 | 0.2505086748200494 | No Hit |
| GCTACGGGTGGCAGCAGTAGGGAATCTTCCGCAATGGACGAAAGTCTGAC | 2231 | 0.24405452118931456 | No Hit |
| CCTACGGGAGGCTGCAGTAGGGAATCTTCCGCAATGGGCGAAAGCCTGAC | 1829 | 0.20007876255278184 | No Hit |
| CCTACGGGAGGCTGCAGTGGGGAATATTGCACAATGGGCGAAAGCCTGAT | 1822 | 0.19931301551184719 | No Hit |
| CCTACGGGAGGCAGCAGTGGGGAATATTGCACAATGGGCGCAAGCCTGAT | 1821 | 0.19920362307742795 | No Hit |
| CCTACGGGCGGCAGCAGTGGGGAATATTGCACAATGGGCGAAAGCCTGAT | 1732 | 0.189467696414116 | No Hit |
| CCTACGGGTGGCAGCAGTGGGGAATATTGCACAATGGGCGCAAGCCTGAT | 1667 | 0.1823571881768657 | No Hit |
| CCTACGGGTGGCTGCAGTGGGGAATATTGCACAATGGGCGAAAGCCTGAT | 1663 | 0.18191961843918875 | No Hit |
| CCTACGGGGGGCAGCAGTGGGGAATATTGCACAATGGGCGCAAGCCTGAT | 1580 | 0.1728400463823922 | No Hit |
| CCTACGGGTGGCTGCAGTAGGGAATCTTCCGCAATGGGCGAAAGCCTGAC | 1432 | 0.15664996608834533 | No Hit |
| CCTACGGGGGGCTGCAGTGGGGAATATTGCACAATGGGCGAAAGCCTGAT | 1375 | 0.1504145973264489 | No Hit |
| CCTACGGGAGGCAGCAGTAGGGAATCTTCGGCAATGGACGAAAGTCTGAC | 1339 | 0.14647646968735642 | No Hit |
| CCTACGGGTGGCAGCAGTAGGGAATCTTCGGCAATGGACGAAAGTCTGAC | 1292 | 0.14133502526965236 | No Hit |
| CCTACGGGAGGCTGCAGTGGGGAATATTGCACAATGGGCGCAAGCCTGAT | 1279 | 0.13991292362220228 | No Hit |
| CCTACGGGGGGCTGCAGTAGGGAATCTTCCGCAATGGGCGAAAGCCTGAC | 1248 | 0.13652175815520598 | No Hit |
| CTTGGTCATTTAGAGGAAGTAAAAGTCGTAACAAGGTTTCCGTAGGTGAA | 1230 | 0.13455269433565975 | No Hit |
| CCTACGGGGGGCAGCAGTAGGGAATCTTCGGCAATGGACGAAAGTCTGAC | 1196 | 0.13083335156540574 | No Hit |
| GCTACGGGAGGCTGCAGTAGGGAATCTTCCGCAATGGACGAAAGTCTGAC | 1178 | 0.1288642877458595 | No Hit |
| CCTACGGGCGGCAGCAGTGGGGAATATTGCACAATGGGCGCAAGCCTGAT | 1170 | 0.12798914827050561 | No Hit |
| CCTACGGGTGGCTGCAGTGGGGAATATTGCACAATGGGCGCAAGCCTGAT | 1136 | 0.1242698055002516 | No Hit |
| CCTACGGGCGGCTGCAGTGGGGAATATTGCACAATGGGCGAAAGCCTGAT | 1075 | 0.11759686700067824 | No Hit |
| CCTACGGGCGGCTGCAGTAGGGAATCTTCCGCAATGGGCGAAAGCCTGAC | 919 | 0.10053164723127749 | No Hit |

## Adapter Content

## Kmer Content

| Sequence | Count | PValue | Obs/Exp Max | Max Obs/Exp Position |
| --- | --- | --- | --- | --- |
| CATGGAA | 10 | 8.2077604E-4 | 298.0522 | 295 |
| AATGGCA | 10 | 8.2077604E-4 | 298.0522 | 295 |
| CTCCAAA | 10 | 8.2077604E-4 | 298.0522 | 295 |
| GCTTGTG | 10 | 8.2077604E-4 | 298.0522 | 295 |
| CTTGGTA | 10 | 8.2077604E-4 | 298.0522 | 295 |
| ATTCCAA | 10 | 8.2077604E-4 | 298.0522 | 295 |
| GATTGGG | 20 | 5.8746082E-8 | 298.0522 | 295 |
| ATTTGCA | 15 | 6.948394E-6 | 298.05215 | 295 |
| AGTGCAG | 1300 | 0.0 | 294.6131 | 295 |
| CCTACTG | 10 | 8.540567E-4 | 294.12292 | 1 |
| CTTGGTC | 145 | 0.0 | 294.12292 | 1 |
| CATACGG | 30 | 5.456968E-12 | 294.12292 | 1 |
| ATTTAGA | 360 | 0.0 | 294.10684 | 8 |
| TTTAGAG | 360 | 0.0 | 294.10684 | 9 |
| GAGTGGC | 30 | 5.456968E-12 | 294.10684 | 6 |
| GGAGCTG | 10 | 8.541966E-4 | 294.10684 | 8 |
| GAGTCAG | 10 | 8.541966E-4 | 294.10684 | 8 |
| TCGGGAG | 15 | 7.3280444E-6 | 294.10684 | 3 |
| TACGGAT | 15 | 7.3280444E-6 | 294.10684 | 3 |
| GGGAGCT | 10 | 8.541966E-4 | 294.10684 | 7 |

Produced by FastQC (version 0.11.7)
