# Supplementary material for: Assessment of airborne bacteria from a public health institution in Mexico City
Source: PLOS Glob Public Health. 2024 Nov 7;4(11):e0003672. doi: 10.1371/journal.pgph.0003672 (PMC11542838; doi:10.1371/journal.pgph.0003672)
Supplement: S1 Text — (ZIP) [file pgph.0003672.s001.zip › Hospital_16S_QC/21022023_BP1D3_16S_S14_L001_R2_001_fastqc.html]

21022023\_BP1D3\_16S\_S14\_L001\_R2\_001.fastq.gz FastQC Report 

FastQC Report

Tue 14 Mar 2023  
21022023\_BP1D3\_16S\_S14\_L001\_R2\_001.fastq.gz

## Summary

- Basic Statistics
- Per base sequence quality
- Per tile sequence quality
- Per sequence quality scores
- Per base sequence content
- Per sequence GC content
- Per base N content
- Sequence Length Distribution
- Sequence Duplication Levels
- Overrepresented sequences
- Adapter Content
- Kmer Content

## Basic Statistics

| Measure | Value |
| --- | --- |
| Filename | 21022023\_BP1D3\_16S\_S14\_L001\_R2\_001.fastq.gz |
| File type | Conventional base calls |
| Encoding | Sanger / Illumina 1.9 |
| Total Sequences | 914140 |
| Sequences flagged as poor quality | 0 |
| Sequence length | 35-301 |
| %GC | 54 |

## Per base sequence quality

## Per tile sequence quality

## Per sequence quality scores

## Per base sequence content

## Per sequence GC content

## Per base N content

## Sequence Length Distribution

## Sequence Duplication Levels

## Overrepresented sequences

| Sequence | Count | Percentage | Possible Source |
| --- | --- | --- | --- |
| GACTACTGGGGTATCTAATCCTGTTTGCTCCCCACGCTTTCGCGCCTCAG | 56957 | 6.2306648872164 | No Hit |
| GACTACTAGGGTATCTAATCCTGTTTGCTCCCCACGCTTTCGCGCCTCAG | 50413 | 5.514800796376922 | No Hit |
| GACTACAGGGGTATCTAATCCTGTTTGCTCCCCACGCTTTCGCGCCTCAG | 49653 | 5.431662546218304 | No Hit |
| GACTACTCGGGTATCTAATCCTGTTTGCTCCCCACGCTTTCGCGCCTCAG | 48463 | 5.3014855492594135 | No Hit |
| GACTACCAGGGTATCTAATCCTGTTTGCTCCCCACGCTTTCGCGCCTCAG | 45984 | 5.0303017043341285 | No Hit |
| GACTACCGGGGTATCTAATCCTGTTTGCTCCCCACGCTTTCGCGCCTCAG | 45870 | 5.017830966810336 | No Hit |
| GACTACAAGGGTATCTAATCCTGTTTGCTCCCCACGCTTTCGCGCCTCAG | 45414 | 4.967948016715164 | No Hit |
| GACTACACGGGTATCTAATCCTGTTTGCTCCCCACGCTTTCGCGCCTCAG | 40450 | 4.424923972258079 | No Hit |
| GACTACCCGGGTATCTAATCCTGTTTGCTCCCCACGCTTTCGCGCCTCAG | 39915 | 4.366399019843787 | No Hit |
| GACTACTGGGGTATCTAATCCTGTTCGCTCCCCACGCTTTCGCGCCTCAG | 37618 | 4.1151245979828035 | No Hit |
| GACTACTAGGGTATCTAATCCTGTTCGCTCCCCACGCTTTCGCGCCTCAG | 33054 | 3.6158575272934126 | No Hit |
| GACTACAGGGGTATCTAATCCTGTTCGCTCCCCACGCTTTCGCGCCTCAG | 32417 | 3.546174546568359 | No Hit |
| GACTACTCGGGTATCTAATCCTGTTCGCTCCCCACGCTTTCGCGCCTCAG | 31833 | 3.482289364867526 | No Hit |
| GACTACCGGGGTATCTAATCCTGTTCGCTCCCCACGCTTTCGCGCCTCAG | 30603 | 3.347736670531866 | No Hit |
| GACTACCAGGGTATCTAATCCTGTTCGCTCCCCACGCTTTCGCGCCTCAG | 30009 | 3.28275756448684 | No Hit |
| GACTACAAGGGTATCTAATCCTGTTCGCTCCCCACGCTTTCGCGCCTCAG | 28877 | 3.158925328724265 | No Hit |
| GACTACACGGGTATCTAATCCTGTTCGCTCCCCACGCTTTCGCGCCTCAG | 26677 | 2.9182619730019472 | No Hit |
| GACTACCCGGGTATCTAATCCTGTTCGCTCCCCACGCTTTCGCGCCTCAG | 26205 | 2.8666287439560683 | No Hit |
| GACTACTGGGGTATCTAATCCTGTTTGCTCCCCACGCTTTCGCACCTCAG | 8585 | 0.9391340494891374 | No Hit |
| GACTACTAGGGTATCTAATCCTGTTTGCTCCCCACGCTTTCGCACCTCAG | 7610 | 0.8324764259303827 | No Hit |
| GACTACAGGGGTATCTAATCCTGTTTGCTCCCCACGCTTTCGCACCTCAG | 7463 | 0.816395738070755 | No Hit |
| GACTACTCGGGTATCTAATCCTGTTTGCTCCCCACGCTTTCGCACCTCAG | 7196 | 0.7871879580808192 | No Hit |
| GACTACCGGGGTATCTAATCCTGTTTGCTCCCCACGCTTTCGCACCTCAG | 6857 | 0.7501039228126982 | No Hit |
| GACTACAAGGGTATCTAATCCTGTTTGCTCCCCACGCTTTCGCACCTCAG | 6831 | 0.7472597195177981 | No Hit |
| GACTACCAGGGTATCTAATCCTGTTTGCTCCCCACGCTTTCGCACCTCAG | 6730 | 0.7362110836414554 | No Hit |
| GACTACACGGGTATCTAATCCTGTTTGCTCCCCACGCTTTCGCACCTCAG | 6008 | 0.6572297459907673 | No Hit |
| GACTACCCGGGTATCTAATCCTGTTTGCTCCCCACGCTTTCGCACCTCAG | 5890 | 0.6443214387292975 | No Hit |
| GACTACTGGGGTATCTAATCCTGTTTGCTCCCCATGCTTTCGCACCTCAG | 5589 | 0.6113943159691075 | No Hit |
| GACTACTAGGGTATCTAATCCTGTTTGCTCCCCATGCTTTCGCACCTCAG | 4812 | 0.5263963944253616 | No Hit |
| GACTACTCGGGTATCTAATCCTGTTTGCTCCCCATGCTTTCGCACCTCAG | 4687 | 0.512722340122957 | No Hit |
| GACTACAGGGGTATCTAATCCTGTTTGCTCCCCATGCTTTCGCACCTCAG | 4637 | 0.5072527184019954 | No Hit |
| GACTACAAGGGTATCTAATCCTGTTTGCTCCCCATGCTTTCGCACCTCAG | 4350 | 0.4758570897236747 | No Hit |
| GACTACCAGGGTATCTAATCCTGTTTGCTCCCCATGCTTTCGCACCTCAG | 4311 | 0.4715907847813245 | No Hit |
| GACTACCGGGGTATCTAATCCTGTTTGCTCCCCATGCTTTCGCACCTCAG | 4292 | 0.469512328527359 | No Hit |
| GACTACACGGGTATCTAATCCTGTTTGCTCCCCATGCTTTCGCACCTCAG | 3839 | 0.4199575557354453 | No Hit |
| GACTACCCGGGTATCTAATCCTGTTTGCTCCCCATGCTTTCGCACCTCAG | 3821 | 0.4179884919158991 | No Hit |
| GACTACTGGGGTATCTAATCCTGTTCGCTCCCCATGCTTTCGCTCCTCAG | 3212 | 0.35136849935458464 | No Hit |
| GACTACTCGGGTATCTAATCCTGTTCGCTCCCCATGCTTTCGCTCCTCAG | 2771 | 0.30312643577570175 | No Hit |
| GACTACAGGGGTATCTAATCCTGTTCGCTCCCCATGCTTTCGCTCCTCAG | 2714 | 0.2968910670138053 | No Hit |
| GACTACTAGGGTATCTAATCCTGTTCGCTCCCCATGCTTTCGCTCCTCAG | 2692 | 0.29448443345658215 | No Hit |
| GACTACCAGGGTATCTAATCCTGTTCGCTCCCCATGCTTTCGCTCCTCAG | 2622 | 0.28682696304723565 | No Hit |
| GACTACCGGGGTATCTAATCCTGTTCGCTCCCCATGCTTTCGCTCCTCAG | 2582 | 0.28245126567046624 | No Hit |
| GACTACAAGGGTATCTAATCCTGTTCGCTCCCCATGCTTTCGCTCCTCAG | 2566 | 0.28070098671975846 | No Hit |
| GACTACACGGGTATCTAATCCTGTTCGCTCCCCATGCTTTCGCTCCTCAG | 2331 | 0.25499376463123813 | No Hit |
| GACTACTGGGGTATCTAATCCTGTTTGATCCCCACGCTTTCGCACATCAG | 2259 | 0.24711750935305313 | No Hit |
| GACTACCCGGGTATCTAATCCTGTTCGCTCCCCATGCTTTCGCTCCTCAG | 2225 | 0.2433981665827991 | No Hit |
| GACTACTAGGGTATCTAATCCTGTTTGATCCCCACGCTTTCGCACATCAG | 2055 | 0.22480145273152907 | No Hit |
| GACTACTGGGGTATCTAATCCTGTTTGCTCCCCACGCTTTCGAGCCTCAG | 1953 | 0.21364342442076706 | No Hit |
| GACTACAGGGGTATCTAATCCTGTTTGATCCCCACGCTTTCGCACATCAG | 1926 | 0.21068982869144767 | No Hit |
| GACTACTCGGGTATCTAATCCTGTTTGATCCCCACGCTTTCGCACATCAG | 1925 | 0.21058043625702846 | No Hit |
| GACTACCGGGGTATCTAATCCTGTTTGATCCCCACGCTTTCGCACATCAG | 1778 | 0.19449974839740083 | No Hit |
| GACTACCAGGGTATCTAATCCTGTTTGATCCCCACGCTTTCGCACATCAG | 1766 | 0.19318703918437 | No Hit |
| GACTACAAGGGTATCTAATCCTGTTTGATCCCCACGCTTTCGCACATCAG | 1744 | 0.19078040562714682 | No Hit |
| GACTACTAGGGTATCTAATCCTGTTTGCTCCCCACGCTTTCGAGCCTCAG | 1714 | 0.18749863259456975 | No Hit |
| GACTACACGGGTATCTAATCCTGTTTGATCCCCACGCTTTCGCACATCAG | 1622 | 0.17743452862800008 | No Hit |
| GACTACTCGGGTATCTAATCCTGTTTGCTCCCCACGCTTTCGAGCCTCAG | 1586 | 0.1734964009889076 | No Hit |
| GACTACAGGGGTATCTAATCCTGTTTGCTCCCCACGCTTTCGAGCCTCAG | 1546 | 0.16912070361213818 | No Hit |
| GACTACCGGGGTATCTAATCCTGTTTGCTCCCCACGCTTTCGAGCCTCAG | 1524 | 0.166714070054915 | No Hit |
| GACTACCCGGGTATCTAATCCTGTTTGATCCCCACGCTTTCGCACATCAG | 1506 | 0.16474500623536878 | No Hit |
| GACTACAAGGGTATCTAATCCTGTTTGCTCCCCACGCTTTCGAGCCTCAG | 1506 | 0.16474500623536878 | No Hit |
| GACTACCAGGGTATCTAATCCTGTTTGCTCCCCACGCTTTCGAGCCTCAG | 1455 | 0.15916599207998774 | No Hit |
| GACTACACGGGTATCTAATCCTGTTTGCTCCCCACGCTTTCGAGCCTCAG | 1354 | 0.14811735620364497 | No Hit |
| GACTACCCGGGTATCTAATCCTGTTTGCTCCCCACGCTTTCGAGCCTCAG | 1309 | 0.14319469665477935 | No Hit |
| GACTACTGGGGTATCTAATCCTGTTTGCTCCCCATGCTTTCGTACCTCAG | 1157 | 0.12656704662305554 | No Hit |
| GACTACTAGGGTATCTAATCCTGTTTGCTCCCCATGCTTTCGTACCTCAG | 1058 | 0.11573719561555124 | No Hit |
| GACTACAGGGGTATCTAATCCTGTTTGCTCCCCATGCTTTCGTACCTCAG | 1009 | 0.11037696632900867 | No Hit |
| GACTACCGGGGTATCTAATCCTGTTTGCTCCCCATGCTTTCGTACCTCAG | 967 | 0.10578248408340078 | No Hit |
| GACTACTCGGGTATCTAATCCTGTTTGCTCCCCATGCTTTCGTACCTCAG | 951 | 0.10403220513269304 | No Hit |

## Adapter Content

## Kmer Content

| Sequence | Count | PValue | Obs/Exp Max | Max Obs/Exp Position |
| --- | --- | --- | --- | --- |
| TTTAGAT | 5 | 5.0682105E-5 | 12698.206 | 295 |
| ACTGCTT | 5 | 5.0682105E-5 | 12698.206 | 295 |
| GTTAGTG | 5 | 5.0682105E-5 | 12698.206 | 295 |
| GTTAGCA | 50 | 0.0 | 12698.206 | 295 |
| GTTAGAA | 5 | 5.0682105E-5 | 12698.206 | 295 |
| TGCGGTT | 5 | 5.0682105E-5 | 12698.206 | 295 |
| GTGTGAT | 5 | 5.0682105E-5 | 12698.206 | 295 |
| GTTCGGG | 10 | 1.0591975E-8 | 12698.206 | 295 |
| GTAAGGG | 5 | 5.0682105E-5 | 12698.206 | 295 |
| GTAAGCG | 15 | 1.8189894E-12 | 12698.206 | 295 |
| CTTAGCA | 5 | 5.0682105E-5 | 12698.206 | 295 |
| GATAGCA | 5 | 5.0682105E-5 | 12698.206 | 295 |
| GGGATCG | 5 | 5.0682105E-5 | 12698.206 | 295 |
| GGTAGCG | 5 | 5.0682105E-5 | 12698.206 | 295 |
| GTTAGCG | 840 | 0.0 | 12471.453 | 295 |
| GTTAGGG | 65 | 0.0 | 11721.421 | 295 |
| GTTTGCG | 60 | 0.0 | 10581.838 | 295 |
| GTTATCG | 30 | 0.0 | 10581.838 | 295 |
| GTTCGCG | 40 | 0.0 | 9523.654 | 295 |
| GTTATGG | 15 | 3.574678E-8 | 8465.471 | 295 |

Produced by FastQC (version 0.11.7)
