# Supplementary material for: Assessment of airborne bacteria from a public health institution in Mexico City
Source: PLOS Glob Public Health. 2024 Nov 7;4(11):e0003672. doi: 10.1371/journal.pgph.0003672 (PMC11542838; doi:10.1371/journal.pgph.0003672)
Supplement: S1 Text — (ZIP) [file pgph.0003672.s001.zip › Hospital_16S_QC/21022023_BP2D1_16S_S15_L001_R1_001_fastqc.html]

21022023\_BP2D1\_16S\_S15\_L001\_R1\_001.fastq.gz FastQC Report 

FastQC Report

Tue 14 Mar 2023  
21022023\_BP2D1\_16S\_S15\_L001\_R1\_001.fastq.gz

## Summary

- Basic Statistics
- Per base sequence quality
- Per tile sequence quality
- Per sequence quality scores
- Per base sequence content
- Per sequence GC content
- Per base N content
- Sequence Length Distribution
- Sequence Duplication Levels
- Overrepresented sequences
- Adapter Content
- Kmer Content

## Basic Statistics

| Measure | Value |
| --- | --- |
| Filename | 21022023\_BP2D1\_16S\_S15\_L001\_R1\_001.fastq.gz |
| File type | Conventional base calls |
| Encoding | Sanger / Illumina 1.9 |
| Total Sequences | 882711 |
| Sequences flagged as poor quality | 0 |
| Sequence length | 35-301 |
| %GC | 53 |

## Per base sequence quality

## Per tile sequence quality

## Per sequence quality scores

## Per base sequence content

## Per sequence GC content

## Per base N content

## Sequence Length Distribution

## Sequence Duplication Levels

## Overrepresented sequences

| Sequence | Count | Percentage | Possible Source |
| --- | --- | --- | --- |
| CCTACGGGAGGCAGCAGTAGGGAATCTTCCGCAATGGGCGAAAGCCTGAC | 87730 | 9.938700208788607 | No Hit |
| CCTACGGGTGGCAGCAGTAGGGAATCTTCCGCAATGGGCGAAAGCCTGAC | 86238 | 9.76967546569602 | No Hit |
| CCTACGGGGGGCAGCAGTAGGGAATCTTCCGCAATGGGCGAAAGCCTGAC | 75990 | 8.608706586867049 | No Hit |
| CCTACGGGCGGCAGCAGTAGGGAATCTTCCGCAATGGGCGAAAGCCTGAC | 57380 | 6.500428792662605 | No Hit |
| CCTACGGGTGGCAGCAGTAGGGAATCTTCCGCAATGGACGAAAGTCTGAC | 55559 | 6.294132507695044 | No Hit |
| CCTACGGGAGGCAGCAGTAGGGAATCTTCCGCAATGGACGAAAGTCTGAC | 55339 | 6.2692092882041806 | No Hit |
| CCTACGGGGGGCAGCAGTAGGGAATCTTCCGCAATGGACGAAAGTCTGAC | 48111 | 5.450368240567977 | No Hit |
| CCTACGGGAGGCTGCAGTAGGGAATCTTCCGCAATGGGCGAAAGCCTGAC | 38308 | 4.339812237527345 | No Hit |
| CCTACGGGCGGCAGCAGTAGGGAATCTTCCGCAATGGACGAAAGTCTGAC | 36819 | 4.171127356518724 | No Hit |
| CCTACGGGTGGCTGCAGTAGGGAATCTTCCGCAATGGGCGAAAGCCTGAC | 32462 | 3.677534323238297 | No Hit |
| CCTACGGGGGGCTGCAGTAGGGAATCTTCCGCAATGGGCGAAAGCCTGAC | 27366 | 3.10022192994083 | No Hit |
| CCTACGGGAGGCTGCAGTAGGGAATCTTCCGCAATGGACGAAAGTCTGAC | 24980 | 2.8299182858262784 | No Hit |
| CCTACGGGTGGCTGCAGTAGGGAATCTTCCGCAATGGACGAAAGTCTGAC | 20741 | 2.3496931611818592 | No Hit |
| CCTACGGGCGGCTGCAGTAGGGAATCTTCCGCAATGGGCGAAAGCCTGAC | 20716 | 2.3468609771488063 | No Hit |
| CCTACGGGGGGCTGCAGTAGGGAATCTTCCGCAATGGACGAAAGTCTGAC | 17803 | 2.0168548936175035 | No Hit |
| CCTACGGGAGGCAGCAGTGGGGAATATTGCACAATGGGCGCAAGCCTGAT | 15622 | 1.769775158573984 | No Hit |
| CCTACGGGTGGCAGCAGTGGGGAATATTGCACAATGGGCGCAAGCCTGAT | 15581 | 1.7651303767597777 | No Hit |
| CCTACGGGGGGCAGCAGTGGGGAATATTGCACAATGGGCGCAAGCCTGAT | 13951 | 1.5804719778047402 | No Hit |
| CCTACGGGCGGCTGCAGTAGGGAATCTTCCGCAATGGACGAAAGTCTGAC | 13829 | 1.5666509197234428 | No Hit |
| CCTACGGGAGGCTGCAGTGGGGAATATTGCACAATGGGCGCAAGCCTGAT | 11435 | 1.2954409767183144 | No Hit |
| CCTACGGGCGGCAGCAGTGGGGAATATTGCACAATGGGCGCAAGCCTGAT | 10312 | 1.1682192699535863 | No Hit |
| CCTACGGGTGGCTGCAGTGGGGAATATTGCACAATGGGCGCAAGCCTGAT | 10277 | 1.1642542123073123 | No Hit |
| CCTACGGGGGGCTGCAGTGGGGAATATTGCACAATGGGCGCAAGCCTGAT | 8637 | 0.9784629397390538 | No Hit |
| CCTACGGGCGGCTGCAGTGGGGAATATTGCACAATGGGCGCAAGCCTGAT | 6725 | 0.7618575048911818 | No Hit |
| CCTACGGGTGGCAGCAGTGGGGAATATTGGACAATGGGCGAAAGCCTGAT | 3870 | 0.43842208831656115 | No Hit |
| CCTACGGGAGGCAGCAGTGGGGAATATTGGACAATGGGCGAAAGCCTGAT | 3769 | 0.4269800648230282 | No Hit |
| CCTACGGGGGGCAGCAGTGGGGAATATTGGACAATGGGCGAAAGCCTGAT | 3447 | 0.3905015344773091 | No Hit |
| CCTACGGGAGGCTGCAGTGGGGAATATTGGACAATGGGCGAAAGCCTGAT | 2779 | 0.31482557711414044 | No Hit |
| CCTACGGGCGGCAGCAGTGGGGAATATTGGACAATGGGCGAAAGCCTGAT | 2481 | 0.281065943440152 | No Hit |
| CCTACGGGTGGCTGCAGTGGGGAATATTGGACAATGGGCGAAAGCCTGAT | 2352 | 0.26645187382959995 | No Hit |
| CCTACGGGGGGCTGCAGTGGGGAATATTGGACAATGGGCGAAAGCCTGAT | 1884 | 0.213433388730853 | No Hit |
| CCTACGGGCGGCTGCAGTGGGGAATATTGGACAATGGGCGAAAGCCTGAT | 1539 | 0.1743492490747255 | No Hit |
| CCTACGGGTGGCAGCAGTAGGGAATCTTCCGCAATGGGCGCAAGCCTGAC | 1260 | 0.1427420752658571 | No Hit |
| CCTACGGGAGGCAGCAGTAGGGAATCTTCCGCAATGGGCGCAAGCCTGAC | 1230 | 0.13934345442619386 | No Hit |
| CTTGGTCATTTAGAGGAAGTAAAAGTCGTAACAAGGTTTCCGTAGGTGAA | 1154 | 0.13073361496571356 | No Hit |
| GCTACGGGAGGCAGCAGTAGGGAATCTTCCGCAATGGACGAAAGTCTGAC | 1124 | 0.12733499412605032 | No Hit |
| CCTACGGGGGGCAGCAGTAGGGAATCTTCCGCAATGGGCGCAAGCCTGAC | 1094 | 0.12393637328638706 | No Hit |
| CCTACGGGTGGCAGCAGTGGGGAATATTGCACAATGGGCGAAAGCCTGAT | 1016 | 0.11509995910326257 | No Hit |
| GCTACGGGGGGCAGCAGTAGGGAATCTTCCGCAATGGACGAAAGTCTGAC | 957 | 0.10841600478525815 | No Hit |
| CCTACGGGAGGCAGCAGTGGGGAATATTGCACAATGGGCGAAAGCCTGAT | 953 | 0.10796285533996972 | No Hit |
| GCTACGGGTGGCAGCAGTAGGGAATCTTCCGCAATGGACGAAAGTCTGAC | 949 | 0.10750970589468126 | No Hit |
| CCTACGGGGGGCAGCAGTGGGGAATATTGCACAATGGGCGAAAGCCTGAT | 909 | 0.10297821144179692 | No Hit |

## Adapter Content

## Kmer Content

| Sequence | Count | PValue | Obs/Exp Max | Max Obs/Exp Position |
| --- | --- | --- | --- | --- |
| AGTCCAG | 25 | 4.965841E-10 | 298.3496 | 295 |
| CATGGAA | 20 | 5.8453224E-8 | 298.34958 | 295 |
| AATGGTA | 10 | 8.1832317E-4 | 298.34958 | 295 |
| CTCCAAA | 10 | 8.1832317E-4 | 298.34958 | 295 |
| GCTTGTG | 10 | 8.1832317E-4 | 298.34958 | 295 |
| CTTGGAA | 90 | 0.0 | 298.34958 | 295 |
| ATTGGCA | 145 | 0.0 | 298.34958 | 295 |
| ATTGGAG | 20 | 5.8453224E-8 | 298.34958 | 295 |
| AGTGCAG | 10165 | 0.0 | 296.1483 | 295 |
| CCTTACG | 10 | 8.538921E-4 | 294.14127 | 1 |
| CCTACCG | 15 | 7.3245355E-6 | 294.14127 | 1 |
| ATGGCAG | 15 | 7.3261926E-6 | 294.12463 | 8 |
| CTGGTGG | 15 | 7.3261926E-6 | 294.12463 | 5 |
| GAGACTG | 15 | 7.3261926E-6 | 294.12463 | 8 |
| GATGGCA | 15 | 7.3261926E-6 | 294.12463 | 7 |
| CTACAGG | 55 | 0.0 | 294.12463 | 2 |
| AGAGGCA | 15 | 7.3261926E-6 | 294.12463 | 7 |
| GAGGGCA | 25 | 5.4023985E-10 | 294.12463 | 7 |
| ATTTAGA | 310 | 0.0 | 294.1246 | 8 |
| TTTAGAG | 310 | 0.0 | 294.1246 | 9 |

Produced by FastQC (version 0.11.7)
