# Supplementary material for: Assessment of airborne bacteria from a public health institution in Mexico City
Source: PLOS Glob Public Health. 2024 Nov 7;4(11):e0003672. doi: 10.1371/journal.pgph.0003672 (PMC11542838; doi:10.1371/journal.pgph.0003672)
Supplement: S1 Text — (ZIP) [file pgph.0003672.s001.zip › Hospital_16S_QC/21022023_BP2D1_16S_S15_L001_R2_001_fastqc.html]

21022023\_BP2D1\_16S\_S15\_L001\_R2\_001.fastq.gz FastQC Report 

FastQC Report

Tue 14 Mar 2023  
21022023\_BP2D1\_16S\_S15\_L001\_R2\_001.fastq.gz

## Summary

- Basic Statistics
- Per base sequence quality
- Per tile sequence quality
- Per sequence quality scores
- Per base sequence content
- Per sequence GC content
- Per base N content
- Sequence Length Distribution
- Sequence Duplication Levels
- Overrepresented sequences
- Adapter Content
- Kmer Content

## Basic Statistics

| Measure | Value |
| --- | --- |
| Filename | 21022023\_BP2D1\_16S\_S15\_L001\_R2\_001.fastq.gz |
| File type | Conventional base calls |
| Encoding | Sanger / Illumina 1.9 |
| Total Sequences | 882711 |
| Sequences flagged as poor quality | 0 |
| Sequence length | 35-301 |
| %GC | 53 |

## Per base sequence quality

## Per tile sequence quality

## Per sequence quality scores

## Per base sequence content

## Per sequence GC content

## Per base N content

## Sequence Length Distribution

## Sequence Duplication Levels

## Overrepresented sequences

| Sequence | Count | Percentage | Possible Source |
| --- | --- | --- | --- |
| GACTACTGGGGTATCTAATCCTGTTTGATCCCCACGCTTTCGCACATCAG | 59617 | 6.7538526199401625 | No Hit |
| GACTACTAGGGTATCTAATCCTGTTTGATCCCCACGCTTTCGCACATCAG | 52770 | 5.978174056967682 | No Hit |
| GACTACAGGGGTATCTAATCCTGTTTGATCCCCACGCTTTCGCACATCAG | 51822 | 5.8707776384343235 | No Hit |
| GACTACTCGGGTATCTAATCCTGTTTGATCCCCACGCTTTCGCACATCAG | 50113 | 5.67716953793484 | No Hit |
| GACTACCGGGGTATCTAATCCTGTTTGATCCCCACGCTTTCGCACATCAG | 48323 | 5.474385161168264 | No Hit |
| GACTACAAGGGTATCTAATCCTGTTTGATCCCCACGCTTTCGCACATCAG | 48081 | 5.446969619728314 | No Hit |
| GACTACCAGGGTATCTAATCCTGTTTGATCCCCACGCTTTCGCACATCAG | 48059 | 5.444477297779228 | No Hit |
| GACTACACGGGTATCTAATCCTGTTTGATCCCCACGCTTTCGCACATCAG | 42196 | 4.780273498347704 | No Hit |
| GACTACCCGGGTATCTAATCCTGTTTGATCCCCACGCTTTCGCACATCAG | 41307 | 4.679561034132349 | No Hit |
| GACTACTGGGGTATCTAATCCTGTTCGCTCCCCACGCTTTCGCGCCTCAG | 36903 | 4.1806434948697815 | No Hit |
| GACTACTAGGGTATCTAATCCTGTTCGCTCCCCACGCTTTCGCGCCTCAG | 32274 | 3.6562362993097404 | No Hit |
| GACTACAGGGGTATCTAATCCTGTTCGCTCCCCACGCTTTCGCGCCTCAG | 32030 | 3.6285941831471455 | No Hit |
| GACTACTCGGGTATCTAATCCTGTTCGCTCCCCACGCTTTCGCGCCTCAG | 30716 | 3.4797345903698944 | No Hit |
| GACTACCGGGGTATCTAATCCTGTTCGCTCCCCACGCTTTCGCGCCTCAG | 29881 | 3.385139643665934 | No Hit |
| GACTACCAGGGTATCTAATCCTGTTCGCTCCCCACGCTTTCGCGCCTCAG | 29178 | 3.3054986286564914 | No Hit |
| GACTACAAGGGTATCTAATCCTGTTCGCTCCCCACGCTTTCGCGCCTCAG | 29076 | 3.293943317801636 | No Hit |
| GACTACACGGGTATCTAATCCTGTTCGCTCCCCACGCTTTCGCGCCTCAG | 25760 | 2.918282427657523 | No Hit |
| GACTACCCGGGTATCTAATCCTGTTCGCTCCCCACGCTTTCGCGCCTCAG | 25292 | 2.8652639425587765 | No Hit |
| GACTACTGGGGTATCTAATCCTGTTCGCTCCCCATGCTTTCGCTCCTCAG | 12183 | 1.3801799229872518 | No Hit |
| GACTACTAGGGTATCTAATCCTGTTCGCTCCCCATGCTTTCGCTCCTCAG | 10870 | 1.231433617571323 | No Hit |
| GACTACAGGGGTATCTAATCCTGTTCGCTCCCCATGCTTTCGCTCCTCAG | 10645 | 1.2059439612738485 | No Hit |
| GACTACTCGGGTATCTAATCCTGTTCGCTCCCCATGCTTTCGCTCCTCAG | 10370 | 1.1747899369102686 | No Hit |
| GACTACCAGGGTATCTAATCCTGTTCGCTCCCCATGCTTTCGCTCCTCAG | 10192 | 1.154624786594933 | No Hit |
| GACTACCGGGGTATCTAATCCTGTTCGCTCCCCATGCTTTCGCTCCTCAG | 9901 | 1.1216581644501995 | No Hit |
| GACTACAAGGGTATCTAATCCTGTTCGCTCCCCATGCTTTCGCTCCTCAG | 9864 | 1.1174665320812813 | No Hit |
| GACTACACGGGTATCTAATCCTGTTCGCTCCCCATGCTTTCGCTCCTCAG | 8726 | 0.9885455148967217 | No Hit |
| GACTACCCGGGTATCTAATCCTGTTCGCTCCCCATGCTTTCGCTCCTCAG | 8420 | 0.9538795823321563 | No Hit |
| GACTACTGGGGTATCTAATCCTGTTTGCTCCCCACGCTTTCGCACCTCAG | 2421 | 0.27426870176082546 | No Hit |
| GACTACTAGGGTATCTAATCCTGTTTGCTCCCCACGCTTTCGCACCTCAG | 2151 | 0.24368111420385608 | No Hit |
| GACTACAGGGGTATCTAATCCTGTTTGCTCCCCACGCTTTCGCACCTCAG | 2081 | 0.23575099891130846 | No Hit |
| GACTACTCGGGTATCTAATCCTGTTTGCTCCCCACGCTTTCGCACCTCAG | 2037 | 0.2307663550131357 | No Hit |
| GACTACCGGGGTATCTAATCCTGTTTGCTCCCCACGCTTTCGCACCTCAG | 2025 | 0.22940690667727037 | No Hit |
| GACTACAAGGGTATCTAATCCTGTTTGCTCCCCACGCTTTCGCACCTCAG | 2008 | 0.22748102153479452 | No Hit |
| GACTACCAGGGTATCTAATCCTGTTTGCTCCCCACGCTTTCGCACCTCAG | 1947 | 0.22057049249414587 | No Hit |
| GACTACACGGGTATCTAATCCTGTTTGCTCCCCACGCTTTCGCACCTCAG | 1831 | 0.20742915858078126 | No Hit |
| GACTACCCGGGTATCTAATCCTGTTTGCTCCCCACGCTTTCGCACCTCAG | 1737 | 0.196780146616503 | No Hit |
| GACTACTGGGGTATCTAATCCTGTTTGATCCCCACGCTTTCGCGCCTCAG | 1007 | 0.11408037285136358 | No Hit |
| GACTACTGGGGTATCTAATCCTGTTCGCTCCCCACGCTTTCGCACATCAG | 969 | 0.10977545312112345 | No Hit |
| GACTACTAGGGTATCTAATCCTGTTCGCTCCCCACGCTTTCGCACATCAG | 929 | 0.10524395866823909 | No Hit |

## Adapter Content

## Kmer Content

| Sequence | Count | PValue | Obs/Exp Max | Max Obs/Exp Position |
| --- | --- | --- | --- | --- |
| GTTCGCG | 25 | 0.0 | 10072.313 | 295 |
| GTTTGGG | 15 | 5.456968E-12 | 10072.3125 | 295 |
| CGTGCTT | 5 | 8.058584E-5 | 10072.3125 | 295 |
| TTATGCG | 5 | 8.058584E-5 | 10072.3125 | 295 |
| ATTCGTT | 5 | 8.058584E-5 | 10072.3125 | 295 |
| GTTAGTG | 5 | 8.058584E-5 | 10072.3125 | 295 |
| GTTAGGT | 5 | 8.058584E-5 | 10072.3125 | 295 |
| GTTAGGG | 75 | 0.0 | 10072.3125 | 295 |
| CGTAGCG | 5 | 8.058584E-5 | 10072.3125 | 295 |
| CTTTGGG | 5 | 8.058584E-5 | 10072.3125 | 295 |
| TTAGTCG | 5 | 8.058584E-5 | 10072.3125 | 295 |
| GTGAGGG | 10 | 2.1251253E-8 | 10072.3125 | 295 |
| TATAGCG | 5 | 8.058584E-5 | 10072.3125 | 295 |
| GTGAGCG | 5 | 8.058584E-5 | 10072.3125 | 295 |
| TAGGCGT | 5 | 8.058584E-5 | 10072.3125 | 295 |
| GTAAGCA | 5 | 8.058584E-5 | 10072.3125 | 295 |
| GTTGGCG | 5 | 8.058584E-5 | 10072.3125 | 295 |
| TAAGAGT | 5 | 8.058584E-5 | 10072.3125 | 295 |
| GTTATCA | 10 | 2.1251253E-8 | 10072.3125 | 295 |
| GATAGCG | 10 | 2.1251253E-8 | 10072.3125 | 295 |

Produced by FastQC (version 0.11.7)
