# Supplementary material for: Assessment of airborne bacteria from a public health institution in Mexico City
Source: PLOS Glob Public Health. 2024 Nov 7;4(11):e0003672. doi: 10.1371/journal.pgph.0003672 (PMC11542838; doi:10.1371/journal.pgph.0003672)
Supplement: S1 Text — (ZIP) [file pgph.0003672.s001.zip › Hospital_16S_QC/21022023_BP2D2_16S_S16_L001_R1_001_fastqc.html]

21022023\_BP2D2\_16S\_S16\_L001\_R1\_001.fastq.gz FastQC Report 

FastQC Report

Tue 14 Mar 2023  
21022023\_BP2D2\_16S\_S16\_L001\_R1\_001.fastq.gz

## Summary

- Basic Statistics
- Per base sequence quality
- Per tile sequence quality
- Per sequence quality scores
- Per base sequence content
- Per sequence GC content
- Per base N content
- Sequence Length Distribution
- Sequence Duplication Levels
- Overrepresented sequences
- Adapter Content
- Kmer Content

## Basic Statistics

| Measure | Value |
| --- | --- |
| Filename | 21022023\_BP2D2\_16S\_S16\_L001\_R1\_001.fastq.gz |
| File type | Conventional base calls |
| Encoding | Sanger / Illumina 1.9 |
| Total Sequences | 886713 |
| Sequences flagged as poor quality | 0 |
| Sequence length | 35-301 |
| %GC | 54 |

## Per base sequence quality

## Per tile sequence quality

## Per sequence quality scores

## Per base sequence content

## Per sequence GC content

## Per base N content

## Sequence Length Distribution

## Sequence Duplication Levels

## Overrepresented sequences

| Sequence | Count | Percentage | Possible Source |
| --- | --- | --- | --- |
| CCTACGGGAGGCAGCAGTAGGGAATCTTCCGCAATGGACGAAAGTCTGAC | 150812 | 17.00798341740789 | No Hit |
| CCTACGGGTGGCAGCAGTAGGGAATCTTCCGCAATGGACGAAAGTCTGAC | 149087 | 16.81344471097187 | No Hit |
| CCTACGGGGGGCAGCAGTAGGGAATCTTCCGCAATGGACGAAAGTCTGAC | 125542 | 14.158132338197365 | No Hit |
| CCTACGGGCGGCAGCAGTAGGGAATCTTCCGCAATGGACGAAAGTCTGAC | 98839 | 11.14667316256782 | No Hit |
| CCTACGGGAGGCTGCAGTAGGGAATCTTCCGCAATGGACGAAAGTCTGAC | 66524 | 7.502314728666434 | No Hit |
| CCTACGGGTGGCTGCAGTAGGGAATCTTCCGCAATGGACGAAAGTCTGAC | 56305 | 6.349856154133299 | No Hit |
| CCTACGGGGGGCTGCAGTAGGGAATCTTCCGCAATGGACGAAAGTCTGAC | 46486 | 5.24250800428098 | No Hit |
| CCTACGGGCGGCTGCAGTAGGGAATCTTCCGCAATGGACGAAAGTCTGAC | 37334 | 4.21038148758392 | No Hit |
| CCTACGGGTGGCAGCAGTAGGGAATCTTCCGCAATGGGCGAAAGCCTGAC | 15635 | 1.7632537247113778 | No Hit |
| CCTACGGGAGGCAGCAGTAGGGAATCTTCCGCAATGGGCGAAAGCCTGAC | 15401 | 1.7368641262731008 | No Hit |
| CCTACGGGGGGCAGCAGTAGGGAATCTTCCGCAATGGGCGAAAGCCTGAC | 13422 | 1.5136803001647658 | No Hit |
| CCTACGGGCGGCAGCAGTAGGGAATCTTCCGCAATGGGCGAAAGCCTGAC | 10321 | 1.1639617328267433 | No Hit |
| CCTACGGGAGGCTGCAGTAGGGAATCTTCCGCAATGGGCGAAAGCCTGAC | 7180 | 0.8097321230206391 | No Hit |
| CCTACGGGTGGCTGCAGTAGGGAATCTTCCGCAATGGGCGAAAGCCTGAC | 5997 | 0.6763180420271272 | No Hit |
| CCTACGGGGGGCTGCAGTAGGGAATCTTCCGCAATGGGCGAAAGCCTGAC | 5055 | 0.5700829919038065 | No Hit |
| CCTACGGGAGGCAGCAGTGGGGAATATTGCACAATGGGCGCAAGCCTGAT | 4334 | 0.4887714514166365 | No Hit |
| CCTACGGGTGGCAGCAGTGGGGAATATTGCACAATGGGCGCAAGCCTGAT | 4287 | 0.4834709765166406 | No Hit |
| CCTACGGGCGGCTGCAGTAGGGAATCTTCCGCAATGGGCGAAAGCCTGAC | 3898 | 0.43960108851454754 | No Hit |
| CCTACGGGGGGCAGCAGTGGGGAATATTGCACAATGGGCGCAAGCCTGAT | 3781 | 0.42640628929540897 | No Hit |
| CCTACGGGAGGCTGCAGTGGGGAATATTGCACAATGGGCGCAAGCCTGAT | 3189 | 0.359642860767802 | No Hit |
| CCTACGGGTGGCTGCAGTGGGGAATATTGCACAATGGGCGCAAGCCTGAT | 2928 | 0.33020830866356987 | No Hit |
| CCTACGGGCGGCAGCAGTGGGGAATATTGCACAATGGGCGCAAGCCTGAT | 2896 | 0.3265994746891046 | No Hit |
| GCTACGGGTGGCAGCAGTAGGGAATCTTCCGCAATGGACGAAAGTCTGAC | 2829 | 0.319043478555068 | No Hit |
| GCTACGGGAGGCAGCAGTAGGGAATCTTCCGCAATGGACGAAAGTCTGAC | 2741 | 0.3091191851252886 | No Hit |
| GCTACGGGGGGCAGCAGTAGGGAATCTTCCGCAATGGACGAAAGTCTGAC | 2467 | 0.2782185442189299 | No Hit |
| CCTACGGGGGGCTGCAGTGGGGAATATTGCACAATGGGCGCAAGCCTGAT | 2283 | 0.25746774886575474 | No Hit |
| CCTACGGGCGGCTGCAGTGGGGAATATTGCACAATGGGCGCAAGCCTGAT | 1964 | 0.2214921851828044 | No Hit |
| GCTACGGGAGGCTGCAGTAGGGAATCTTCCGCAATGGACGAAAGTCTGAC | 1468 | 0.16555525857859307 | No Hit |
| GCTACGGGTGGCTGCAGTAGGGAATCTTCCGCAATGGACGAAAGTCTGAC | 1227 | 0.1383762277084017 | No Hit |
| GCTACGGGGGGCTGCAGTAGGGAATCTTCCGCAATGGACGAAAGTCTGAC | 1122 | 0.1265347412296876 | No Hit |
| CTTGGTCATTTAGAGGAAGTAAAAGTCGTAACAAGGTTTCCGTAGGTGAA | 981 | 0.11063331652970015 | No Hit |
| GCTACGGGCGGCAGCAGTAGGGAATCTTCCGCAATGGACGAAAGTCTGAC | 900 | 0.101498455531835 | No Hit |

## Adapter Content

## Kmer Content

| Sequence | Count | PValue | Obs/Exp Max | Max Obs/Exp Position |
| --- | --- | --- | --- | --- |
| ACATGGA | 15 | 6.6973535E-6 | 300.8113 | 295 |
| ATTGGTA | 15 | 6.6973535E-6 | 300.8113 | 295 |
| CCATGGA | 15 | 6.6973535E-6 | 300.8113 | 295 |
| GATTGGT | 30 | 3.6379788E-12 | 300.8113 | 295 |
| GCTTGTG | 15 | 6.6973535E-6 | 300.8113 | 295 |
| CATGGCA | 15 | 6.6973535E-6 | 300.8113 | 295 |
| CATTGCG | 10 | 7.984289E-4 | 300.81128 | 295 |
| ATTGGCA | 25 | 4.7293724E-10 | 300.81128 | 295 |
| CCTTGGG | 45 | 0.0 | 300.81128 | 295 |
| CATTTGG | 20 | 5.610491E-8 | 300.81128 | 295 |
| AATGGGA | 10 | 7.984289E-4 | 300.81128 | 295 |
| CTTTGTA | 10 | 7.984289E-4 | 300.81128 | 295 |
| GCTTTGG | 10 | 7.984289E-4 | 300.81128 | 295 |
| CATCGGA | 10 | 7.984289E-4 | 300.81128 | 295 |
| AGTGCAG | 2830 | 0.0 | 294.96512 | 295 |
| GATGGCT | 10 | 8.541548E-4 | 294.11115 | 7 |
| CCTACTG | 10 | 8.541548E-4 | 294.11115 | 1 |
| CCTACGA | 65 | 0.0 | 294.11115 | 1 |
| CCTACCG | 10 | 8.541548E-4 | 294.11115 | 1 |
| GTGCAGC | 10 | 8.541548E-4 | 294.11115 | 9 |

Produced by FastQC (version 0.11.7)
