# Supplementary material for: Assessment of airborne bacteria from a public health institution in Mexico City
Source: PLOS Glob Public Health. 2024 Nov 7;4(11):e0003672. doi: 10.1371/journal.pgph.0003672 (PMC11542838; doi:10.1371/journal.pgph.0003672)
Supplement: S1 Text — (ZIP) [file pgph.0003672.s001.zip › Hospital_16S_QC/21022023_BP2D2_16S_S16_L001_R2_001_fastqc.html]

21022023\_BP2D2\_16S\_S16\_L001\_R2\_001.fastq.gz FastQC Report 

FastQC Report

Tue 14 Mar 2023  
21022023\_BP2D2\_16S\_S16\_L001\_R2\_001.fastq.gz

## Summary

- Basic Statistics
- Per base sequence quality
- Per tile sequence quality
- Per sequence quality scores
- Per base sequence content
- Per sequence GC content
- Per base N content
- Sequence Length Distribution
- Sequence Duplication Levels
- Overrepresented sequences
- Adapter Content
- Kmer Content

## Basic Statistics

| Measure | Value |
| --- | --- |
| Filename | 21022023\_BP2D2\_16S\_S16\_L001\_R2\_001.fastq.gz |
| File type | Conventional base calls |
| Encoding | Sanger / Illumina 1.9 |
| Total Sequences | 886713 |
| Sequences flagged as poor quality | 0 |
| Sequence length | 35-301 |
| %GC | 54 |

## Per base sequence quality

## Per tile sequence quality

## Per sequence quality scores

## Per base sequence content

## Per sequence GC content

## Per base N content

## Sequence Length Distribution

## Sequence Duplication Levels

## Overrepresented sequences

| Sequence | Count | Percentage | Possible Source |
| --- | --- | --- | --- |
| GACTACTGGGGTATCTAATCCTGTTCGCTCCCCACGCTTTCGCTCCTCAG | 97888 | 11.039423127889183 | No Hit |
| GACTACTAGGGTATCTAATCCTGTTCGCTCCCCACGCTTTCGCTCCTCAG | 87578 | 9.876701931741161 | No Hit |
| GACTACAGGGGTATCTAATCCTGTTCGCTCCCCACGCTTTCGCTCCTCAG | 85818 | 9.678216063145573 | No Hit |
| GACTACTCGGGTATCTAATCCTGTTCGCTCCCCACGCTTTCGCTCCTCAG | 82616 | 9.317107113575645 | No Hit |
| GACTACCGGGGTATCTAATCCTGTTCGCTCCCCACGCTTTCGCTCCTCAG | 79445 | 8.95949422191848 | No Hit |
| GACTACCAGGGTATCTAATCCTGTTCGCTCCCCACGCTTTCGCTCCTCAG | 79183 | 8.929946893752543 | No Hit |
| GACTACAAGGGTATCTAATCCTGTTCGCTCCCCACGCTTTCGCTCCTCAG | 78338 | 8.834651121614321 | No Hit |
| GACTACACGGGTATCTAATCCTGTTCGCTCCCCACGCTTTCGCTCCTCAG | 70163 | 7.912706817200153 | No Hit |
| GACTACCCGGGTATCTAATCCTGTTCGCTCCCCACGCTTTCGCTCCTCAG | 67837 | 7.650389697681212 | No Hit |
| GACTACTGGGGTATCTAATCCTGTTTGATCCCCACGCTTTCGCACATCAG | 10408 | 1.1737732501948206 | No Hit |
| GACTACTAGGGTATCTAATCCTGTTTGATCCCCACGCTTTCGCACATCAG | 9451 | 1.0658465591459694 | No Hit |
| GACTACAGGGGTATCTAATCCTGTTTGATCCCCACGCTTTCGCACATCAG | 9178 | 1.0350586943013127 | No Hit |
| GACTACTCGGGTATCTAATCCTGTTTGATCCCCACGCTTTCGCACATCAG | 8677 | 0.9785578873885913 | No Hit |
| GACTACCAGGGTATCTAATCCTGTTTGATCCCCACGCTTTCGCACATCAG | 8526 | 0.9615287020715836 | No Hit |
| GACTACCGGGGTATCTAATCCTGTTTGATCCCCACGCTTTCGCACATCAG | 8494 | 0.9579198680971183 | No Hit |
| GACTACAAGGGTATCTAATCCTGTTTGATCCCCACGCTTTCGCACATCAG | 8282 | 0.9340113430162861 | No Hit |
| GACTACACGGGTATCTAATCCTGTTTGATCCCCACGCTTTCGCACATCAG | 7551 | 0.8515720419120956 | No Hit |
| GACTACCCGGGTATCTAATCCTGTTTGATCCCCACGCTTTCGCACATCAG | 7143 | 0.8055594087376636 | No Hit |
| GACTACTGGGGTATCTAATCCTGTTCGCTCCCCATGCTTTCGCTCCTCAG | 3217 | 0.36280059049545904 | No Hit |
| GACTACTAGGGTATCTAATCCTGTTCGCTCCCCATGCTTTCGCTCCTCAG | 2891 | 0.3260355943805944 | No Hit |
| GACTACTCGGGTATCTAATCCTGTTCGCTCCCCATGCTTTCGCTCCTCAG | 2754 | 0.3105852739274151 | No Hit |
| GACTACAGGGGTATCTAATCCTGTTCGCTCCCCATGCTTTCGCTCCTCAG | 2630 | 0.29660104227636225 | No Hit |
| GACTACCAGGGTATCTAATCCTGTTCGCTCCCCATGCTTTCGCTCCTCAG | 2579 | 0.2908494631295583 | No Hit |
| GACTACCGGGGTATCTAATCCTGTTCGCTCCCCATGCTTTCGCTCCTCAG | 2560 | 0.2887067179572195 | No Hit |
| GACTACAAGGGTATCTAATCCTGTTCGCTCCCCATGCTTTCGCTCCTCAG | 2538 | 0.2862256445997747 | No Hit |
| GACTACACGGGTATCTAATCCTGTTCGCTCCCCATGCTTTCGCTCCTCAG | 2305 | 0.25994882222319965 | No Hit |
| GACTACCCGGGTATCTAATCCTGTTCGCTCCCCATGCTTTCGCTCCTCAG | 2231 | 0.25160339365724876 | No Hit |
| GACTACTGGGGTATCTAATCCTGTTTGCTCCCCACGCTTTCGAGCCTCAG | 1446 | 0.16307418522114822 | No Hit |
| GACTACAGGGGTATCTAATCCTGTTTGCTCCCCACGCTTTCGAGCCTCAG | 1286 | 0.145030015348822 | No Hit |
| GACTACTAGGGTATCTAATCCTGTTTGCTCCCCACGCTTTCGAGCCTCAG | 1252 | 0.1411956292509527 | No Hit |
| GACTACTCGGGTATCTAATCCTGTTTGCTCCCCACGCTTTCGAGCCTCAG | 1136 | 0.12811360609351616 | No Hit |
| GACTACCAGGGTATCTAATCCTGTTTGCTCCCCACGCTTTCGAGCCTCAG | 1127 | 0.12709862153819781 | No Hit |
| GACTACAAGGGTATCTAATCCTGTTTGCTCCCCACGCTTTCGAGCCTCAG | 1120 | 0.12630918910628355 | No Hit |
| GACTACCGGGGTATCTAATCCTGTTTGCTCCCCACGCTTTCGAGCCTCAG | 1046 | 0.11796376054033267 | No Hit |
| GACTACACGGGTATCTAATCCTGTTTGCTCCCCACGCTTTCGAGCCTCAG | 971 | 0.10950555591267976 | No Hit |
| GACTACCCGGGTATCTAATCCTGTTTGCTCCCCACGCTTTCGAGCCTCAG | 970 | 0.10939277985097771 | No Hit |

## Adapter Content

## Kmer Content

| Sequence | Count | PValue | Obs/Exp Max | Max Obs/Exp Position |
| --- | --- | --- | --- | --- |
| CTCGTTT | 5 | 8.236905E-5 | 9962.834 | 295 |
| GTTCTCG | 5 | 8.236905E-5 | 9962.834 | 295 |
| TTCAGTG | 5 | 8.236905E-5 | 9962.834 | 295 |
| TTAGCAG | 5 | 8.236905E-5 | 9962.834 | 295 |
| TTAGACG | 10 | 2.1960659E-8 | 9962.834 | 295 |
| ATAGCCG | 5 | 8.236905E-5 | 9962.834 | 295 |
| TGAACAA | 10 | 2.1960659E-8 | 9962.834 | 295 |
| GTTAGTA | 5 | 8.236905E-5 | 9962.834 | 295 |
| GTTAGGT | 5 | 8.236905E-5 | 9962.834 | 295 |
| GTTAGGG | 90 | 0.0 | 9962.834 | 295 |
| TGTATCA | 5 | 8.236905E-5 | 9962.834 | 295 |
| GTGTGGG | 10 | 2.1960659E-8 | 9962.834 | 295 |
| GTATGCG | 5 | 8.236905E-5 | 9962.834 | 295 |
| CGTAGCG | 10 | 2.1960659E-8 | 9962.834 | 295 |
| GATATGG | 5 | 8.236905E-5 | 9962.834 | 295 |
| GATATCA | 5 | 8.236905E-5 | 9962.834 | 295 |
| CTTTGGA | 5 | 8.236905E-5 | 9962.834 | 295 |
| AGTCTCG | 5 | 8.236905E-5 | 9962.834 | 295 |
| GGAGTAG | 5 | 8.236905E-5 | 9962.834 | 295 |
| CGTACCG | 5 | 8.236905E-5 | 9962.834 | 295 |

Produced by FastQC (version 0.11.7)
