# Supplementary material for: Assessment of airborne bacteria from a public health institution in Mexico City
Source: PLOS Glob Public Health. 2024 Nov 7;4(11):e0003672. doi: 10.1371/journal.pgph.0003672 (PMC11542838; doi:10.1371/journal.pgph.0003672)
Supplement: S1 Text — (ZIP) [file pgph.0003672.s001.zip › Hospital_16S_QC/21022023_BUD1_16S_S18_L001_R1_001_fastqc.html]

21022023\_BUD1\_16S\_S18\_L001\_R1\_001.fastq.gz FastQC Report 

FastQC Report

Tue 14 Mar 2023  
21022023\_BUD1\_16S\_S18\_L001\_R1\_001.fastq.gz

## Summary

- Basic Statistics
- Per base sequence quality
- Per tile sequence quality
- Per sequence quality scores
- Per base sequence content
- Per sequence GC content
- Per base N content
- Sequence Length Distribution
- Sequence Duplication Levels
- Overrepresented sequences
- Adapter Content
- Kmer Content

## Basic Statistics

| Measure | Value |
| --- | --- |
| Filename | 21022023\_BUD1\_16S\_S18\_L001\_R1\_001.fastq.gz |
| File type | Conventional base calls |
| Encoding | Sanger / Illumina 1.9 |
| Total Sequences | 1128248 |
| Sequences flagged as poor quality | 0 |
| Sequence length | 35-301 |
| %GC | 53 |

## Per base sequence quality

## Per tile sequence quality

## Per sequence quality scores

## Per base sequence content

## Per sequence GC content

## Per base N content

## Sequence Length Distribution

## Sequence Duplication Levels

## Overrepresented sequences

| Sequence | Count | Percentage | Possible Source |
| --- | --- | --- | --- |
| CCTACGGGTGGCAGCAGTAGGGAATCTTCCGCAATGGACGAAAGTCTGAC | 144739 | 12.828651147620027 | No Hit |
| CCTACGGGAGGCAGCAGTAGGGAATCTTCCGCAATGGACGAAAGTCTGAC | 144256 | 12.785841410753665 | No Hit |
| CCTACGGGGGGCAGCAGTAGGGAATCTTCCGCAATGGACGAAAGTCTGAC | 123118 | 10.912317150130113 | No Hit |
| CCTACGGGCGGCAGCAGTAGGGAATCTTCCGCAATGGACGAAAGTCTGAC | 96982 | 8.59580517758507 | No Hit |
| CCTACGGGAGGCTGCAGTAGGGAATCTTCCGCAATGGACGAAAGTCTGAC | 65808 | 5.832760173295233 | No Hit |
| CCTACGGGTGGCTGCAGTAGGGAATCTTCCGCAATGGACGAAAGTCTGAC | 54838 | 4.860456211754862 | No Hit |
| CCTACGGGGGGCTGCAGTAGGGAATCTTCCGCAATGGACGAAAGTCTGAC | 45957 | 4.0733065779864 | No Hit |
| CCTACGGGAGGCAGCAGTGGGGAATATTGGACAATGGGCGAAAGCCTGAT | 40514 | 3.5908771830306816 | No Hit |
| CCTACGGGTGGCAGCAGTGGGGAATATTGGACAATGGGCGAAAGCCTGAT | 40271 | 3.569339365104126 | No Hit |
| CCTACGGGCGGCTGCAGTAGGGAATCTTCCGCAATGGACGAAAGTCTGAC | 36818 | 3.263289631357645 | No Hit |
| CCTACGGGGGGCAGCAGTGGGGAATATTGGACAATGGGCGAAAGCCTGAT | 35553 | 3.1511688919457423 | No Hit |
| CCTACGGGAGGCTGCAGTGGGGAATATTGGACAATGGGCGAAAGCCTGAT | 29185 | 2.586753976076182 | No Hit |
| CCTACGGGCGGCAGCAGTGGGGAATATTGGACAATGGGCGAAAGCCTGAT | 27836 | 2.467188065035347 | No Hit |
| CCTACGGGTGGCTGCAGTGGGGAATATTGGACAATGGGCGAAAGCCTGAT | 25818 | 2.2883266799497983 | No Hit |
| CCTACGGGGGGCTGCAGTGGGGAATATTGGACAATGGGCGAAAGCCTGAT | 20406 | 1.808644907857138 | No Hit |
| CCTACGGGAGGCAGCAGTGGGGAATATTGGACAATGGGGGGAACCCTGAT | 16884 | 1.4964794974154618 | No Hit |
| CCTACGGGCGGCTGCAGTGGGGAATATTGGACAATGGGCGAAAGCCTGAT | 16632 | 1.474143982528664 | No Hit |
| CCTACGGGTGGCAGCAGTGGGGAATATTGGACAATGGGGGGAACCCTGAT | 16629 | 1.4738780835419163 | No Hit |
| CCTACGGGGGGCAGCAGTGGGGAATATTGGACAATGGGGGGAACCCTGAT | 14499 | 1.2850898029511242 | No Hit |
| CCTACGGGAGGCTGCAGTGGGGAATATTGGACAATGGGGGGAACCCTGAT | 12140 | 1.0760045663719324 | No Hit |
| CCTACGGGCGGCAGCAGTGGGGAATATTGGACAATGGGGGGAACCCTGAT | 11686 | 1.035765186377463 | No Hit |
| CCTACGGGTGGCTGCAGTGGGGAATATTGGACAATGGGGGGAACCCTGAT | 10603 | 0.9397756521615815 | No Hit |
| CCTACGGGGGGCTGCAGTGGGGAATATTGGACAATGGGGGGAACCCTGAT | 8331 | 0.7384014861980699 | No Hit |
| CCTACGGGCGGCTGCAGTGGGGAATATTGGACAATGGGGGGAACCCTGAT | 7135 | 0.6323964234813623 | No Hit |
| GCTACGGGTGGCAGCAGTAGGGAATCTTCCGCAATGGACGAAAGTCTGAC | 2642 | 0.23416837432904825 | No Hit |
| GCTACGGGAGGCAGCAGTAGGGAATCTTCCGCAATGGACGAAAGTCTGAC | 2636 | 0.23363657635555304 | No Hit |
| GCTACGGGGGGCAGCAGTAGGGAATCTTCCGCAATGGACGAAAGTCTGAC | 2360 | 0.20917386957477435 | No Hit |
| GCTACGGGAGGCTGCAGTAGGGAATCTTCCGCAATGGACGAAAGTCTGAC | 1512 | 0.13401308932078762 | No Hit |
| CCTACGGGAGGCAGCAGTAGGGAATCTTCCGCAATGGGCGAAAGCCTGAC | 1455 | 0.12896100857258333 | No Hit |
| CCTACGGGTGGCAGCAGTAGGGAATCTTCCGCAATGGGCGAAAGCCTGAC | 1448 | 0.12834057760350562 | No Hit |
| CCTACGGGGGGCAGCAGTAGGGAATCTTCCGCAATGGGCGAAAGCCTGAC | 1321 | 0.11708418716452412 | No Hit |
| GCTACGGGTGGCTGCAGTAGGGAATCTTCCGCAATGGACGAAAGTCTGAC | 1228 | 0.10884131857534869 | No Hit |

## Adapter Content

## Kmer Content

| Sequence | Count | PValue | Obs/Exp Max | Max Obs/Exp Position |
| --- | --- | --- | --- | --- |
| GACCGCA | 10 | 8.06368E-4 | 299.8229 | 295 |
| ATTCGAG | 10 | 8.06368E-4 | 299.8229 | 295 |
| CATTCGT | 10 | 8.06368E-4 | 299.8229 | 295 |
| ATGCACA | 10 | 8.06368E-4 | 299.8229 | 295 |
| GTTTGTG | 30 | 3.6379788E-12 | 299.82288 | 295 |
| CATTGGT | 175 | 0.0 | 299.82288 | 295 |
| GATTTGG | 15 | 6.786435E-6 | 299.82288 | 295 |
| ATCCAAA | 19755 | 0.0 | 295.6492 | 295 |
| CCTACTG | 10 | 8.540912E-4 | 294.12198 | 1 |
| CATACTC | 20 | 6.277514E-8 | 294.12198 | 1 |
| CTACAGG | 80 | 0.0 | 294.12198 | 2 |
| TTCGTCA | 15 | 7.326993E-6 | 294.12195 | 2 |
| CTCGGTC | 30 | 5.456968E-12 | 294.12195 | 1 |
| TTTAGAG | 345 | 0.0 | 294.10895 | 9 |
| GAGTGGC | 30 | 5.456968E-12 | 294.10895 | 6 |
| AGCGGCA | 10 | 8.542046E-4 | 294.10895 | 7 |
| GGGACAG | 10 | 8.542046E-4 | 294.10895 | 8 |
| GATGGCT | 20 | 6.2789695E-8 | 294.10895 | 7 |
| GAGCGGC | 10 | 8.542046E-4 | 294.10895 | 6 |
| TGGGGCA | 10 | 8.542046E-4 | 294.10895 | 7 |

Produced by FastQC (version 0.11.7)
