# Supplementary material for: Assessment of airborne bacteria from a public health institution in Mexico City
Source: PLOS Glob Public Health. 2024 Nov 7;4(11):e0003672. doi: 10.1371/journal.pgph.0003672 (PMC11542838; doi:10.1371/journal.pgph.0003672)
Supplement: S1 Text — (ZIP) [file pgph.0003672.s001.zip › Hospital_16S_QC/21022023_BUD1_16S_S18_L001_R2_001_fastqc.html]

21022023\_BUD1\_16S\_S18\_L001\_R2\_001.fastq.gz FastQC Report 

FastQC Report

Tue 14 Mar 2023  
21022023\_BUD1\_16S\_S18\_L001\_R2\_001.fastq.gz

## Summary

- Basic Statistics
- Per base sequence quality
- Per tile sequence quality
- Per sequence quality scores
- Per base sequence content
- Per sequence GC content
- Per base N content
- Sequence Length Distribution
- Sequence Duplication Levels
- Overrepresented sequences
- Adapter Content
- Kmer Content

## Basic Statistics

| Measure | Value |
| --- | --- |
| Filename | 21022023\_BUD1\_16S\_S18\_L001\_R2\_001.fastq.gz |
| File type | Conventional base calls |
| Encoding | Sanger / Illumina 1.9 |
| Total Sequences | 1128248 |
| Sequences flagged as poor quality | 0 |
| Sequence length | 35-301 |
| %GC | 54 |

## Per base sequence quality

## Per tile sequence quality

## Per sequence quality scores

## Per base sequence content

## Per sequence GC content

## Per base N content

## Sequence Length Distribution

## Sequence Duplication Levels

## Overrepresented sequences

| Sequence | Count | Percentage | Possible Source |
| --- | --- | --- | --- |
| GACTACTGGGGTATCTAATCCTGTTCGCTCCCCACGCTTTCGCTCCTCAG | 89926 | 7.970410760754727 | No Hit |
| GACTACTAGGGTATCTAATCCTGTTCGCTCCCCACGCTTTCGCTCCTCAG | 79054 | 7.0067928327814455 | No Hit |
| GACTACAGGGGTATCTAATCCTGTTCGCTCCCCACGCTTTCGCTCCTCAG | 76955 | 6.820752175053711 | No Hit |
| GACTACTCGGGTATCTAATCCTGTTCGCTCCCCACGCTTTCGCTCCTCAG | 75447 | 6.687093617715253 | No Hit |
| GACTACCGGGGTATCTAATCCTGTTCGCTCCCCACGCTTTCGCTCCTCAG | 71838 | 6.367217136657898 | No Hit |
| GACTACCAGGGTATCTAATCCTGTTCGCTCCCCACGCTTTCGCTCCTCAG | 71196 | 6.3103147534939135 | No Hit |
| GACTACAAGGGTATCTAATCCTGTTCGCTCCCCACGCTTTCGCTCCTCAG | 70966 | 6.28992916450993 | No Hit |
| GACTACACGGGTATCTAATCCTGTTCGCTCCCCACGCTTTCGCTCCTCAG | 63854 | 5.6595712999269665 | No Hit |
| GACTACCCGGGTATCTAATCCTGTTCGCTCCCCACGCTTTCGCTCCTCAG | 61712 | 5.469719423389184 | No Hit |
| GACTACTGGGGTATCTAATCCTGTTTGCTCCCCACGCTTTCGCACCTCAG | 28497 | 2.5257744751154 | No Hit |
| GACTACTAGGGTATCTAATCCTGTTTGCTCCCCACGCTTTCGCACCTCAG | 25536 | 2.2633321751955244 | No Hit |
| GACTACAGGGGTATCTAATCCTGTTTGCTCCCCACGCTTTCGCACCTCAG | 24787 | 2.1969460615042085 | No Hit |
| GACTACTCGGGTATCTAATCCTGTTTGCTCCCCACGCTTTCGCACCTCAG | 24125 | 2.1382710184285725 | No Hit |
| GACTACAAGGGTATCTAATCCTGTTTGCTCCCCACGCTTTCGCACCTCAG | 22941 | 2.033329551658855 | No Hit |
| GACTACCGGGGTATCTAATCCTGTTTGCTCCCCACGCTTTCGCACCTCAG | 22893 | 2.0290751678708934 | No Hit |
| GACTACCAGGGTATCTAATCCTGTTTGCTCCCCACGCTTTCGCACCTCAG | 22603 | 2.0033715991519596 | No Hit |
| GACTACACGGGTATCTAATCCTGTTTGCTCCCCACGCTTTCGCACCTCAG | 20332 | 1.8020860661840303 | No Hit |
| GACTACCCGGGTATCTAATCCTGTTTGCTCCCCACGCTTTCGCACCTCAG | 19784 | 1.7535151846048032 | No Hit |
| GACTACTGGGGTATCTAATCCTGTTTGCTCCCCATGCTTTCGCACCTCAG | 13961 | 1.2374052513277223 | No Hit |
| GACTACTAGGGTATCTAATCCTGTTTGCTCCCCATGCTTTCGCACCTCAG | 12253 | 1.0860200948727585 | No Hit |
| GACTACAGGGGTATCTAATCCTGTTTGCTCCCCATGCTTTCGCACCTCAG | 12001 | 1.0636845799859604 | No Hit |
| GACTACTCGGGTATCTAATCCTGTTTGCTCCCCATGCTTTCGCACCTCAG | 11676 | 1.0348788564216378 | No Hit |
| GACTACCGGGGTATCTAATCCTGTTTGCTCCCCATGCTTTCGCACCTCAG | 11134 | 0.9868397728159056 | No Hit |
| GACTACAAGGGTATCTAATCCTGTTTGCTCCCCATGCTTTCGCACCTCAG | 11025 | 0.9771787762974098 | No Hit |
| GACTACCAGGGTATCTAATCCTGTTTGCTCCCCATGCTTTCGCACCTCAG | 10972 | 0.9724812275315357 | No Hit |
| GACTACACGGGTATCTAATCCTGTTTGCTCCCCATGCTTTCGCACCTCAG | 9909 | 0.8782643532273047 | No Hit |
| GACTACCCGGGTATCTAATCCTGTTTGCTCCCCATGCTTTCGCACCTCAG | 9476 | 0.8398862661400684 | No Hit |
| GACTACTGGGGTATCTAATCCTGTTTGCTCCCCACGCTTTCGCGCCTCAG | 6861 | 0.6081109826917486 | No Hit |
| GACTACAGGGGTATCTAATCCTGTTTGCTCCCCACGCTTTCGCGCCTCAG | 6048 | 0.5360523572831505 | No Hit |
| GACTACTAGGGTATCTAATCCTGTTTGCTCCCCACGCTTTCGCGCCTCAG | 5994 | 0.5312661755216939 | No Hit |
| GACTACTCGGGTATCTAATCCTGTTTGCTCCCCACGCTTTCGCGCCTCAG | 5774 | 0.5117669164935369 | No Hit |
| GACTACAAGGGTATCTAATCCTGTTTGCTCCCCACGCTTTCGCGCCTCAG | 5577 | 0.4943062163637782 | No Hit |
| GACTACCGGGGTATCTAATCCTGTTTGCTCCCCACGCTTTCGCGCCTCAG | 5557 | 0.49253355645212754 | No Hit |
| GACTACCAGGGTATCTAATCCTGTTTGCTCCCCACGCTTTCGCGCCTCAG | 5503 | 0.48774737469067087 | No Hit |
| GACTACACGGGTATCTAATCCTGTTTGCTCCCCACGCTTTCGCGCCTCAG | 4914 | 0.4355425402925598 | No Hit |
| GACTACCCGGGTATCTAATCCTGTTTGCTCCCCACGCTTTCGCGCCTCAG | 4626 | 0.4100162375647907 | No Hit |
| GACTACTGGGGTATCTAATCCTGTTCGCTCCCCACGCTTTCGCACCTCAG | 2147 | 0.19029504151569515 | No Hit |
| GACTACTAGGGTATCTAATCCTGTTCGCTCCCCACGCTTTCGCACCTCAG | 2091 | 0.18533159376307337 | No Hit |
| GACTACAAGGGTATCTAATCCTGTTCGCTCCCCACGCTTTCGCACCTCAG | 1904 | 0.16875722358913997 | No Hit |
| GACTACAGGGGTATCTAATCCTGTTCGCTCCCCACGCTTTCGCACCTCAG | 1904 | 0.16875722358913997 | No Hit |
| GACTACTCGGGTATCTAATCCTGTTCGCTCCCCACGCTTTCGCACCTCAG | 1880 | 0.16663003169515922 | No Hit |
| GACTACCAGGGTATCTAATCCTGTTCGCTCCCCACGCTTTCGCACCTCAG | 1814 | 0.16078025398671214 | No Hit |
| GACTACCGGGGTATCTAATCCTGTTCGCTCCCCACGCTTTCGCACCTCAG | 1634 | 0.14482631478185648 | No Hit |
| GACTACACGGGTATCTAATCCTGTTCGCTCCCCACGCTTTCGCACCTCAG | 1633 | 0.14473768178627394 | No Hit |
| GACTACCCGGGTATCTAATCCTGTTCGCTCCCCACGCTTTCGCACCTCAG | 1509 | 0.13374719033404003 | No Hit |
| GACTACTGGGGTATCTAATCCTGTTTGCTCCCCACGCTTTCGCTCCTCAG | 1367 | 0.12116130496132055 | No Hit |
| GACTACTAGGGTATCTAATCCTGTTTGCTCCCCACGCTTTCGCTCCTCAG | 1310 | 0.11610922421311626 | No Hit |
| GACTACTGGGGTATCTAATCCTGTTCGCTCCCCATGCTTTCGCTCCTCAG | 1299 | 0.1151342612617084 | No Hit |
| GACTACTCGGGTATCTAATCCTGTTTGCTCCCCACGCTTTCGCTCCTCAG | 1265 | 0.11212073941190234 | No Hit |
| GACTACAGGGGTATCTAATCCTGTTTGCTCCCCACGCTTTCGCTCCTCAG | 1216 | 0.1077777226283583 | No Hit |
| GACTACTAGGGTATCTAATCCTGTTCGCTCCCCATGCTTTCGCTCCTCAG | 1209 | 0.10715729165928058 | No Hit |
| GACTACTGGGGTATCTAATCCTGTTTGATCCCCACGCTTTCGCACATCAG | 1206 | 0.10689139267253299 | No Hit |
| GACTACCAGGGTATCTAATCCTGTTTGCTCCCCACGCTTTCGCTCCTCAG | 1162 | 0.10299154086690161 | No Hit |
| GACTACTAGGGTATCTAATCCTGTTTGATCCCCACGCTTTCGCACATCAG | 1144 | 0.10139614694641604 | No Hit |

## Adapter Content

## Kmer Content

| Sequence | Count | PValue | Obs/Exp Max | Max Obs/Exp Position |
| --- | --- | --- | --- | --- |
| GGTAGCG | 25 | 0.0 | 8543.249 | 295 |
| TTTAGAG | 5 | 1.1208566E-4 | 8543.248 | 295 |
| GGTACGG | 5 | 1.1208566E-4 | 8543.248 | 295 |
| GTCTGCG | 5 | 1.1208566E-4 | 8543.248 | 295 |
| GGACTCG | 5 | 1.1208566E-4 | 8543.248 | 295 |
| ATAGCCG | 5 | 1.1208566E-4 | 8543.248 | 295 |
| GTTAGTG | 20 | 0.0 | 8543.248 | 295 |
| CGATTCG | 5 | 1.1208566E-4 | 8543.248 | 295 |
| TGCATTG | 5 | 1.1208566E-4 | 8543.248 | 295 |
| TGTTGCG | 5 | 1.1208566E-4 | 8543.248 | 295 |
| CCATCAT | 5 | 1.1208566E-4 | 8543.248 | 295 |
| CGTAGCG | 5 | 1.1208566E-4 | 8543.248 | 295 |
| GGTATCG | 5 | 1.1208566E-4 | 8543.248 | 295 |
| TTTATGG | 5 | 1.1208566E-4 | 8543.248 | 295 |
| GTGAGCG | 10 | 3.4891855E-8 | 8543.248 | 295 |
| GTAAGCG | 5 | 1.1208566E-4 | 8543.248 | 295 |
| ATCATAT | 5 | 1.1208566E-4 | 8543.248 | 295 |
| GATAGCG | 20 | 0.0 | 8543.248 | 295 |
| GATAGAG | 5 | 1.1208566E-4 | 8543.248 | 295 |
| GGTAGGG | 20 | 0.0 | 8543.248 | 295 |

Produced by FastQC (version 0.11.7)
