# Supplementary material for: Assessment of airborne bacteria from a public health institution in Mexico City
Source: PLOS Glob Public Health. 2024 Nov 7;4(11):e0003672. doi: 10.1371/journal.pgph.0003672 (PMC11542838; doi:10.1371/journal.pgph.0003672)
Supplement: S1 Text — (ZIP) [file pgph.0003672.s001.zip › Hospital_16S_QC/21022023_CED1_16S_S40_L001_R1_001_fastqc.html]

21022023\_CED1\_16S\_S40\_L001\_R1\_001.fastq.gz FastQC Report 

FastQC Report

Tue 14 Mar 2023  
21022023\_CED1\_16S\_S40\_L001\_R1\_001.fastq.gz

## Summary

- Basic Statistics
- Per base sequence quality
- Per tile sequence quality
- Per sequence quality scores
- Per base sequence content
- Per sequence GC content
- Per base N content
- Sequence Length Distribution
- Sequence Duplication Levels
- Overrepresented sequences
- Adapter Content
- Kmer Content

## Basic Statistics

| Measure | Value |
| --- | --- |
| Filename | 21022023\_CED1\_16S\_S40\_L001\_R1\_001.fastq.gz |
| File type | Conventional base calls |
| Encoding | Sanger / Illumina 1.9 |
| Total Sequences | 178309 |
| Sequences flagged as poor quality | 0 |
| Sequence length | 80-301 |
| %GC | 56 |

## Per base sequence quality

## Per tile sequence quality

## Per sequence quality scores

## Per base sequence content

## Per sequence GC content

## Per base N content

## Sequence Length Distribution

## Sequence Duplication Levels

## Overrepresented sequences

| Sequence | Count | Percentage | Possible Source |
| --- | --- | --- | --- |
| CCTACGGGTGGCTGCAGTGGGGAATATTGGACAATGGGCGCAAGCCTGAT | 3376 | 1.8933424560734455 | No Hit |
| CCTACGGGAGGCTGCAGTGGGGAATATTGGACAATGGGCGCAAGCCTGAT | 3333 | 1.869227016022747 | No Hit |
| CCTACGGGGGGCTGCAGTGGGGAATATTGGACAATGGGCGCAAGCCTGAT | 3046 | 1.7082704742890154 | No Hit |
| CCTACGGGGGGCAGCAGTGGGGAATATTGGACAATGGGCGCAAGCCTGAT | 2996 | 1.6802292649277377 | No Hit |
| CCTACGGGTGGCTGCAGTGGGGAATATTGCACAATGGGCGCAAGCCTGAT | 2994 | 1.6791076165532868 | No Hit |
| CCTACGGGAGGCTGCAGTGGGGAATATTGCACAATGGGCGCAAGCCTGAT | 2904 | 1.6286334397029874 | No Hit |
| CCTACGGGGGGCTGCAGTGGGGAATATTGCACAATGGGCGCAAGCCTGAT | 2894 | 1.6230251978307322 | No Hit |
| CCTACGGGAGGCTGCAGTGGGGAATATTGCACAATGGGCGAAAGCCTGAT | 2699 | 1.5136644813217506 | No Hit |
| CCTACGGGTGGCTGCAGTGGGGAATATTGCACAATGGGCGAAAGCCTGAT | 2630 | 1.4749676124031876 | No Hit |
| CCTACGGGAGGCAGCAGTGGGGAATATTGGACAATGGGCGCAAGCCTGAT | 2573 | 1.4430006337313315 | No Hit |
| CCTACGGGTGGCTGCAGTGGGGAATCTTAGACAATGGGGGCAACCCTGAT | 2555 | 1.4329057983612716 | No Hit |
| CCTACGGGGGGCTGCAGTGGGGAATATTGCACAATGGGCGAAAGCCTGAT | 2527 | 1.4172027211189564 | No Hit |
| CCTACGGGGGGCAGCAGTGGGGAATATTGCACAATGGGCGCAAGCCTGAT | 2514 | 1.4099120066850244 | No Hit |
| CCTACGGGAGGCTGCAGTGGGGAATATTGGACAATGGGCGAAAGCCTGAT | 2445 | 1.3712151377664616 | No Hit |
| CCTACGGGTGGCAGCAGTGGGGAATATTGGACAATGGGCGCAAGCCTGAT | 2411 | 1.352147115400793 | No Hit |
| CCTACGGGAGGCTGCAGTGGGGAATCTTAGACAATGGGGGCAACCCTGAT | 2393 | 1.342052280030733 | No Hit |
| CCTACGGGGGGCTGCAGTGGGGAATCTTAGACAATGGGGGCAACCCTGAT | 2389 | 1.339808983281831 | No Hit |
| CCTACGGGCGGCTGCAGTGGGGAATATTGGACAATGGGCGCAAGCCTGAT | 2387 | 1.3386873349073798 | No Hit |
| CCTACGGGTGGCTGCAGTGGGGAATATTGGACAATGGGCGAAAGCCTGAT | 2383 | 1.3364440381584777 | No Hit |
| CCTACGGGGGGCAGCAGTGGGGAATATTGCACAATGGGCGAAAGCCTGAT | 2293 | 1.2859698613081785 | No Hit |
| CCTACGGGGGGCTGCAGTGGGGAATATTGGACAATGGGCGAAAGCCTGAT | 2257 | 1.2657801905680588 | No Hit |
| CCTACGGGAGGCAGCAGTGGGGAATATTGCACAATGGGCGCAAGCCTGAT | 2226 | 1.248394640764067 | No Hit |
| CCTACGGGTGGCAGCAGTGGGGAATATTGCACAATGGGCGCAAGCCTGAT | 2111 | 1.183899859233129 | No Hit |
| CCTACGGGGGGCAGCAGTGGGGAATATTGGACAATGGGCGAAAGCCTGAT | 2093 | 1.173805023863069 | No Hit |
| CCTACGGGGGGCAGCAGTGGGGAATCTTAGACAATGGGGGCAACCCTGAT | 2062 | 1.1564194740590772 | No Hit |
| CCTACGGGAGGCAGCAGTGGGGAATATTGCACAATGGGCGAAAGCCTGAT | 2054 | 1.1519328805612727 | No Hit |
| CCTACGGGCGGCTGCAGTGGGGAATATTGCACAATGGGCGCAAGCCTGAT | 2013 | 1.1289390888850255 | No Hit |
| CCTACGGGCGGCAGCAGTGGGGAATATTGGACAATGGGCGCAAGCCTGAT | 1939 | 1.087438099030335 | No Hit |
| CCTACGGGCGGCTGCAGTGGGGAATATTGCACAATGGGCGAAAGCCTGAT | 1930 | 1.082390681345305 | No Hit |
| CCTACGGGAGGCTGCAGTGGGGAATATTGCACAATGGGCGGAAGCCTGAT | 1913 | 1.0728566701624709 | No Hit |
| CCTACGGGAGGCAGCAGTGGGGAATATTGGACAATGGGCGAAAGCCTGAT | 1910 | 1.0711741976007942 | No Hit |
| CCTACGGGTGGCTGCAGTGGGGAATATTGCACAATGGGCGGAAGCCTGAT | 1904 | 1.0678092524774407 | No Hit |
| CCTACGGGTGGCAGCAGTGGGGAATATTGCACAATGGGCGAAAGCCTGAT | 1897 | 1.063883483166862 | No Hit |
| CCTACGGGCGGCTGCAGTGGGGAATCTTAGACAATGGGGGCAACCCTGAT | 1831 | 1.026869086809976 | No Hit |
| CCTACGGGAGGCAGCAGTGGGGAATCTTAGACAATGGGGGCAACCCTGAT | 1820 | 1.020700020750495 | No Hit |
| CCTACGGGGGGCTGCAGTGGGGAATATTGCACAATGGGCGGAAGCCTGAT | 1817 | 1.0190175481888184 | No Hit |
| CCTACGGGCGGCTGCAGTGGGGAATATTGGACAATGGGCGAAAGCCTGAT | 1740 | 0.9758340857724512 | No Hit |
| CCTACGGGTGGCAGCAGTGGGGAATATTGGACAATGGGCGAAAGCCTGAT | 1721 | 0.9651784262151658 | No Hit |
| CCTACGGGCGGCAGCAGTGGGGAATATTGCACAATGGGCGCAAGCCTGAT | 1634 | 0.9163867219265434 | No Hit |
| CCTACGGGTGGCAGCAGTGGGGAATCTTAGACAATGGGGGCAACCCTGAT | 1602 | 0.8984403479353257 | No Hit |
| CCTACGGGCGGCAGCAGTGGGGAATATTGCACAATGGGCGAAAGCCTGAT | 1523 | 0.8541352371445077 | No Hit |
| CCTACGGGGGGCAGCAGTGGGGAATATTGCACAATGGGCGGAAGCCTGAT | 1492 | 0.8367496873405156 | No Hit |
| CCTACGGGAGGCAGCAGTGGGGAATATTGCACAATGGGCGGAAGCCTGAT | 1410 | 0.7907621039880208 | No Hit |
| CCTACGGGCGGCAGCAGTGGGGAATCTTAGACAATGGGGGCAACCCTGAT | 1396 | 0.7829105653668631 | No Hit |
| CCTACGGGCGGCAGCAGTGGGGAATATTGGACAATGGGCGAAAGCCTGAT | 1376 | 0.7716940816223522 | No Hit |
| CCTACGGGCGGCTGCAGTGGGGAATATTGCACAATGGGCGGAAGCCTGAT | 1339 | 0.750943586695007 | No Hit |
| CCTACGGGTGGCAGCAGTGGGGAATATTGCACAATGGGCGGAAGCCTGAT | 1244 | 0.69766528890858 | No Hit |
| CCTACGGGTGGCTGCAGTGGGGAATTTTGGACAATGGGCGCAAGCCTGAT | 1031 | 0.5782097370295386 | No Hit |
| CCTACGGGAGGCTGCAGTGGGGAATTTTGGACAATGGGCGCAAGCCTGAT | 1028 | 0.576527264467862 | No Hit |
| CCTACGGGGGGCTGCAGTGGGGAATTTTGGACAATGGGCGCAAGCCTGAT | 1026 | 0.5754056160934109 | No Hit |
| CCTACGGGCGGCAGCAGTGGGGAATATTGCACAATGGGCGGAAGCCTGAT | 949 | 0.5322221536770438 | No Hit |
| CCTACGGGGGGCAGCAGTGGGGAATTTTGGACAATGGGCGCAAGCCTGAT | 916 | 0.5137149554986007 | No Hit |
| CCTACGGGAGGCAGCAGTGGGGAATTTTGGACAATGGGCGCAAGCCTGAT | 827 | 0.4638016028355271 | No Hit |
| CCTACGGGTGGCAGCAGTGGGGAATTTTGGACAATGGGCGCAAGCCTGAT | 754 | 0.4228614371680621 | No Hit |
| CCTACGGGTGGCTGCAGTGGGGAATATTGCGCAATGGGCGAAAGCCTGAC | 711 | 0.3987459971173637 | No Hit |
| CCTACGGGCGGCTGCAGTGGGGAATTTTGGACAATGGGCGCAAGCCTGAT | 699 | 0.3920161068706571 | No Hit |
| CCTACGGGGGGCTGCAGTGGGGAATATTGCGCAATGGGCGAAAGCCTGAC | 597 | 0.33481203977365137 | No Hit |
| CCTACGGGAGGCTGCAGTGGGGAATATTGCGCAATGGGCGAAAGCCTGAC | 593 | 0.33256874302474915 | No Hit |
| CCTACGGGGGGCAGCAGTAGGGAATCTTCCGCAATGGACGAAAGTCTGAC | 570 | 0.3196697867185616 | No Hit |
| CCTACGGGCGGCAGCAGTGGGGAATTTTGGACAATGGGCGCAAGCCTGAT | 563 | 0.3157440174079828 | No Hit |
| CCTACGGGTGGCAGCAGTAGGGAATCTTCCGCAATGGACGAAAGTCTGAC | 551 | 0.3090141271612762 | No Hit |
| CCTACGGGAGGCAGCAGTAGGGAATCTTCCGCAATGGACGAAAGTCTGAC | 531 | 0.29779764341676523 | No Hit |
| CCTACGGGGGGCAGCAGTGGGGAATATTGCGCAATGGGCGAAAGCCTGAC | 514 | 0.288263632233931 | No Hit |
| CCTACGGGAGGCTGCAGTAGGGAATCTTCCGCAATGGACGAAAGTCTGAC | 508 | 0.2848986871105777 | No Hit |
| CCTACGGGCGGCTGCAGTGGGGAATATTGCGCAATGGGCGAAAGCCTGAC | 471 | 0.2641481921832325 | No Hit |
| CCTACGGGTGGCTGCAGTGGGGAATCTTGCGCAATGGGCGAAAGCCTGAC | 443 | 0.24844511494091717 | No Hit |
| CCTACGGGAGGCAGCAGTGGGGAATATTGCGCAATGGGCGAAAGCCTGAC | 429 | 0.2405935763197595 | No Hit |
| CCTACGGGAGGCTGCAGTGGGGAATCTTGCGCAATGGGCGAAAGCCTGAC | 420 | 0.23554615863472958 | No Hit |
| CCTACGGGAGGCTGCAGTGGGGAATATTGCGCAATGGGCGGAAGCCTGAC | 419 | 0.23498533444750402 | No Hit |
| CCTACGGGTGGCTGCAGTGGGGAATATTGCGCAATGGGCGGAAGCCTGAC | 413 | 0.2316203893241508 | No Hit |
| CCTACGGGTGGCTGCAGTAGGGAATCTTCCGCAATGGACGAAAGTCTGAC | 411 | 0.2304987409496997 | No Hit |
| CCTACGGGTGGCAGCAGTGGGGAATATTGCGCAATGGGCGAAAGCCTGAC | 403 | 0.22601214745189532 | No Hit |
| CCTACGGGGGGCTGCAGTAGGGAATCTTCCGCAATGGACGAAAGTCTGAC | 402 | 0.22545132326466977 | No Hit |
| CCTACGGGGGGCTGCAGTGGGGAATATTGCGCAATGGGCGGAAGCCTGAC | 394 | 0.2209647297668654 | No Hit |
| CCTACGGGTGGCTGCAGTGGGGAATATTGGACAATGGGGGGAACCCTGAT | 393 | 0.22040390557963985 | No Hit |
| CCTACGGGGGGCTGCAGTGGGGAATCTTGCGCAATGGGCGAAAGCCTGAC | 389 | 0.21816060883073765 | No Hit |
| CCTACGGGGGGCAGCAGTGGGGAATATTGGACAATGGGGGGAACCCTGAT | 382 | 0.21423483952015882 | No Hit |
| CTTGGTCATTTAGAGGAAGTAAAAGTCGTAACAAGGTTTCCGTAGGTGAA | 371 | 0.20806577346067784 | No Hit |
| CCTACGGGAGGCTGCAGTGGGGAATATTGGACAATGGGGGGAACCCTGAT | 370 | 0.20750494927345228 | No Hit |
| CCTACGGGAGGCAGCAGTGGGGAATATTGGACAATGGGGGGAACCCTGAT | 369 | 0.20694412508622673 | No Hit |
| CCTACGGGCGGCAGCAGTAGGGAATCTTCCGCAATGGACGAAAGTCTGAC | 368 | 0.20638330089900117 | No Hit |
| CCTACGGGTGGCTGCAGTGGGGAATATTGGACAATGGGCGGAAGCCTGAT | 367 | 0.20582247671177564 | No Hit |
| CCTACGGGAGGCTGCAGTGGGGAATATTGGACAATGGGCGGAAGCCTGAT | 366 | 0.2052616525245501 | No Hit |
| CCTACGGGGGGCAGCAGTGGGGAATATTGCGCAATGGGCGGAAGCCTGAC | 362 | 0.20301835577564792 | No Hit |
| CCTACGGGAGGCAGCAGTGGGGAATATTGCGCAATGGGCGGAAGCCTGAC | 360 | 0.2018967074011968 | No Hit |
| CCTACGGGGGGCTGCAGTGGGGAATATTGGACAATGGGCGGAAGCCTGAT | 359 | 0.20133588321397125 | No Hit |
| CCTACGGGAGGCTGCAGTGGGGAATATTGCACAATGGGGGAAACCCTGAT | 357 | 0.20021423483952017 | No Hit |
| CCTACGGGCGGCAGCAGTGGGGAATATTGCGCAATGGGCGAAAGCCTGAC | 353 | 0.197970938090618 | No Hit |
| CCTACGGGAGGCTGCAGTGGGGAATCTTAGACAATGGGCGCAAGCCTGAT | 352 | 0.19741011390339244 | No Hit |
| CCTACGGGGGGCTGCAGTGGGGAATATTGGACAATGGGGGCAACCCTGAT | 352 | 0.19741011390339244 | No Hit |
| CCTACGGGTGGCTGCAGTGGGGAATATTGCACAATGGGGGAAACCCTGAT | 351 | 0.1968492897161669 | No Hit |
| CCTACGGGGGGCTGCAGTGGGGAATATTGGACAATGGGGGGAACCCTGAT | 350 | 0.19628846552894133 | No Hit |
| CCTACGGGTGGCTGCAGTGGGGAATATTGGACAATGGGGGCAACCCTGAT | 343 | 0.1923626962183625 | No Hit |
| CCTACGGGGGGCAGCAGTGGGGAATATTGGACAATGGGCGGAAGCCTGAT | 343 | 0.1923626962183625 | No Hit |
| CCTACGGGAGGCTGCAGTGGGGAATATTGGACAATGGGGGCAACCCTGAT | 341 | 0.1912410478439114 | No Hit |
| CCTACGGGGGGCAGCAGTGGGGAATCTTGCGCAATGGGCGAAAGCCTGAC | 337 | 0.18899775109500921 | No Hit |
| CCTACGGGAGGCAGCAGTGGGGAATCTTGCGCAATGGGCGAAAGCCTGAC | 334 | 0.18731527853333257 | No Hit |
| CCTACGGGGGGCAGCAGTAGGGAATATTGGGCAATGGGCGAGAGCCTGAC | 331 | 0.18563280597165593 | No Hit |
| CCTACGGGTGGCTGCAGTGGGGAATCTTAGACAATGGGCGCAAGCCTGAT | 327 | 0.18338950922275377 | No Hit |
| CCTACGGGGGGCTGCAGTGGGGAATCTTAGACAATGGGCGCAAGCCTGAT | 310 | 0.17385549803991945 | No Hit |
| CCTACGGGCGGCTGCAGTGGGGAATCTTGCGCAATGGGCGAAAGCCTGAC | 304 | 0.17049055291656617 | No Hit |
| CCTACGGGTGGCAGCAGTGGGGAATATTGGACAATGGGGGGAACCCTGAT | 303 | 0.16992972872934062 | No Hit |
| CCTACGGGGGGCAGCAGTGGGGAATATTGGACAATGGGGGCAACCCTGAT | 302 | 0.1693689045421151 | No Hit |
| CCTACGGGGGGCTGCAGTGGGGAATATTGCACAATGGGGGAAACCCTGAT | 295 | 0.16544313523153628 | No Hit |
| CCTACGGGTGGCAGCAGTGGGGAATATTGGACAATGGGCGGAAGCCTGAT | 294 | 0.16488231104431073 | No Hit |
| CCTACGGGCGGCTGCAGTGGGGAATATTGCGCAATGGGCGGAAGCCTGAC | 293 | 0.16432148685708517 | No Hit |
| CCTACGGGTGGCAGCAGTGGGGAATATTGCGCAATGGGCGGAAGCCTGAC | 289 | 0.16207819010818297 | No Hit |
| CCTACGGGTGGCAGCAGTGGGGAATCTTGCGCAATGGGCGAAAGCCTGAC | 287 | 0.1609565417337319 | No Hit |
| CCTACGGGTGGCAGCAGTGGGGAATATTGGACAATGGGGGCAACCCTGAT | 285 | 0.1598348933592808 | No Hit |
| CCTACGGGAGGCAGCAGTGGGGAATATTGGACAATGGGCGGAAGCCTGAT | 285 | 0.1598348933592808 | No Hit |
| CCTACGGGGGGCAGCAGTAGGGAATCTTCCGCAATGGGCGAAAGCCTGAC | 283 | 0.1587132449848297 | No Hit |
| CCTACGGGGGGCAGCAGTGGGGAATCTTAGACAATGGGCGCAAGCCTGAT | 282 | 0.15815242079760414 | No Hit |
| CCTACGGGCGGCTGCAGTGGGGAATATTGGACAATGGGGGCAACCCTGAT | 279 | 0.15646994823592753 | No Hit |
| CCTACGGGAGGCAGCAGTGGGGAATATTGGACAATGGGGGCAACCCTGAT | 277 | 0.15534829986147644 | No Hit |
| CCTACGGGAGGCAGCAGTAGGGAATATTGGGCAATGGGCGAGAGCCTGAC | 274 | 0.1536658272997998 | No Hit |
| CCTACGGGCGGCTGCAGTGGGGAATATTGGACAATGGGGGGAACCCTGAT | 273 | 0.15310500311257424 | No Hit |
| CCTACGGGCGGCTGCAGTGGGGAATATTGGACAATGGGCGGAAGCCTGAT | 267 | 0.14974005798922096 | No Hit |
| CCTACGGGCGGCTGCAGTAGGGAATCTTCCGCAATGGACGAAAGTCTGAC | 265 | 0.14861840961476988 | No Hit |
| CCTACGGGAGGCAGCAGTGGGGAATCTTAGACAATGGGCGCAAGCCTGAT | 262 | 0.1469359370530932 | No Hit |
| CCTACGGGCGGCTGCAGTGGGGAATCTTAGACAATGGGCGCAAGCCTGAT | 262 | 0.1469359370530932 | No Hit |
| CCTACGGGGGGCAGCAGTGGGGAATATTGCACAATGGGGGAAACCCTGAT | 260 | 0.14581428867864213 | No Hit |
| CCTACGGGGGGCTGCAGTGGGGAATTTTCCGCAATGGGCGAAAGCCTGAC | 254 | 0.14244934355528885 | No Hit |
| CCTACGGGGGGCAGCAGTAAGGAATATTGGTCAATGGACGCAAGTCTGAA | 252 | 0.14132769518083776 | No Hit |
| CCTACGGGCGGCAGCAGTGGGGAATCTTGCGCAATGGGCGAAAGCCTGAC | 246 | 0.13796275005748448 | No Hit |
| CCTACGGGAGGCTGCAGTAAGGAATATTGGTCAATGGACGCAAGTCTGAA | 245 | 0.13740192587025893 | No Hit |
| CCTACGGGAGGCAGCAGTGGGGAATATTGCACAATGGGGGAAACCCTGAT | 243 | 0.13628027749580784 | No Hit |
| CCTACGGGTGGCTGCAGTAAGGAATATTGGTCAATGGACGCAAGTCTGAA | 241 | 0.13515862912135676 | No Hit |
| CCTACGGGTGGCAGCAGTAGGGAATATTGGGCAATGGGCGAGAGCCTGAC | 237 | 0.13291533237245456 | No Hit |
| CCTACGGGCGGCAGCAGTGGGGAATATTGGACAATGGGGGGAACCCTGAT | 236 | 0.132354508185229 | No Hit |
| CCTACGGGTGGCAGCAGTGGGGAATCTTAGACAATGGGCGCAAGCCTGAT | 230 | 0.12898956306187573 | No Hit |
| CCTACGGGCGGCAGCAGTGGGGAATATTGCGCAATGGGCGGAAGCCTGAC | 228 | 0.12786791468742462 | No Hit |
| CCTACGGGCGGCTGCAGTGGGGAATATTGCACAATGGGGGAAACCCTGAT | 226 | 0.12674626631297353 | No Hit |
| CCTACGGGCGGCAGCAGTGGGGAATATTGGACAATGGGCGGAAGCCTGAT | 226 | 0.12674626631297353 | No Hit |
| CCTACGGGGGGCTGCAGTAAGGAATATTGGTCAATGGACGCAAGTCTGAA | 223 | 0.12506379375129692 | No Hit |
| CCTACGGGTGGCAGCAGTAGGGAATCTTCCGCAATGGGCGAAAGCCTGAC | 222 | 0.12450296956407136 | No Hit |
| CCTACGGGTGGCAGCAGTAAGGAATATTGGTCAATGGACGCAAGTCTGAA | 221 | 0.12394214537684581 | No Hit |
| CCTACGGGTGGCTGCAGTGGGGAATTTTCCGCAATGGGCGAAAGCCTGAC | 220 | 0.12338132118962027 | No Hit |
| CCTACGGGGGGCTGCAGTAGGGAATATTGGGCAATGGGCGAGAGCCTGAC | 218 | 0.12225967281516918 | No Hit |
| CCTACGGGTGGCTGCAGTAGGGAATCTTCCGCAATGGGCGAAAGCCTGAC | 217 | 0.12169884862794363 | No Hit |
| CCTACGGGAGGCAGCAGTAAGGAATATTGGTCAATGGACGCAAGTCTGAA | 216 | 0.12113802444071808 | No Hit |
| CCTACGGGAGGCTGCAGTAGGGAATCTTCCGCAATGGGCGAAAGCCTGAC | 212 | 0.11889472769181589 | No Hit |
| CCTACGGGAGGCAGCAGTAGGGAATCTTCCGCAATGGGCGAAAGCCTGAC | 211 | 0.11833390350459035 | No Hit |
| CCTACGGGGGGCTGCAGTAGGGAATCTTCCGCAATGGGCGAAAGCCTGAC | 208 | 0.1166514309429137 | No Hit |
| CCTACGGGAGGCTGCAGTGGGGAATTTTCCGCAATGGGCGAAAGCCTGAC | 205 | 0.11496895838123705 | No Hit |
| CCTACGGGCGGCAGCAGTGGGGAATATTGGACAATGGGGGCAACCCTGAT | 204 | 0.11440813419401152 | No Hit |
| CCTACGGGTGGCTGCAGTAGGGAATATTGGGCAATGGGCGAGAGCCTGAC | 204 | 0.11440813419401152 | No Hit |
| CCTACGGGTGGCAGCAGTGGGGAATATTGCACAATGGGGGAAACCCTGAT | 203 | 0.11384731000678597 | No Hit |
| CCTACGGGAGGCAGCAGTGGGGAATTTTCCGCAATGGGCGAAAGCCTGAC | 194 | 0.10879989232175605 | No Hit |
| CCTACGGGCGGCAGCAGTGGGGAATATTGCACAATGGGGGAAACCCTGAT | 189 | 0.10599577138562831 | No Hit |
| CCTACGGGAGGCTGCAGTAGGGAATATTGGGCAATGGGCGAGAGCCTGAC | 188 | 0.10543494719840278 | No Hit |
| CCTACGGGTGGCTGCAGTGGGGAATTTTGGACAATGGGCGAAAGCCTGAT | 187 | 0.10487412301117723 | No Hit |
| CCTACGGGTGGCTGCAGTGGGGAATCTTAGACAATGGGCGAAAGCCTGAT | 183 | 0.10263082626227504 | No Hit |

## Adapter Content

## Kmer Content

| Sequence | Count | PValue | Obs/Exp Max | Max Obs/Exp Position |
| --- | --- | --- | --- | --- |
| GTATTGG | 20 | 3.9655788E-8 | 322.31644 | 295 |
| ATGTGAT | 65 | 0.0 | 322.31644 | 295 |
| GATTCGA | 10 | 6.4877135E-4 | 322.31644 | 295 |
| AGTGGAT | 15 | 5.0761337E-6 | 322.3164 | 295 |
| AGTGTTG | 430 | 0.0 | 322.3164 | 295 |
| AGTGTGG | 660 | 0.0 | 319.87463 | 295 |
| GAGAGAG | 2255 | 0.0 | 318.74307 | 295 |
| ATTTGAA | 320 | 0.0 | 317.28024 | 295 |
| ATCTGAT | 165 | 0.0 | 312.54926 | 295 |
| AGGTATG | 230 | 0.0 | 308.30267 | 295 |
| ATTGGAA | 655 | 0.0 | 297.7121 | 295 |
| CGACAGC | 10 | 8.551145E-4 | 293.93524 | 9 |
| GGGAGAC | 10 | 8.551145E-4 | 293.93524 | 6 |
| CTCGGTC | 10 | 8.551145E-4 | 293.93524 | 1 |
| GGCGACA | 10 | 8.551145E-4 | 293.93524 | 7 |
| ATTTCGA | 55 | 0.0 | 293.01492 | 295 |
| CCTACGG | 17325 | 0.0 | 292.57794 | 1 |
| TGGAGAG | 700 | 0.0 | 292.38702 | 295 |
| CGGCTGC | 1945 | 0.0 | 291.66837 | 9 |
| ATCCAAA | 315 | 0.0 | 291.6196 | 295 |

Produced by FastQC (version 0.11.7)
