# Supplementary material for: Assessment of airborne bacteria from a public health institution in Mexico City
Source: PLOS Glob Public Health. 2024 Nov 7;4(11):e0003672. doi: 10.1371/journal.pgph.0003672 (PMC11542838; doi:10.1371/journal.pgph.0003672)
Supplement: S1 Text — (ZIP) [file pgph.0003672.s001.zip › Hospital_16S_QC/21022023_CED2_16S_S41_L001_R1_001_fastqc.html]

21022023\_CED2\_16S\_S41\_L001\_R1\_001.fastq.gz FastQC Report 

FastQC Report

Tue 14 Mar 2023  
21022023\_CED2\_16S\_S41\_L001\_R1\_001.fastq.gz

## Summary

- Basic Statistics
- Per base sequence quality
- Per tile sequence quality
- Per sequence quality scores
- Per base sequence content
- Per sequence GC content
- Per base N content
- Sequence Length Distribution
- Sequence Duplication Levels
- Overrepresented sequences
- Adapter Content
- Kmer Content

## Basic Statistics

| Measure | Value |
| --- | --- |
| Filename | 21022023\_CED2\_16S\_S41\_L001\_R1\_001.fastq.gz |
| File type | Conventional base calls |
| Encoding | Sanger / Illumina 1.9 |
| Total Sequences | 446993 |
| Sequences flagged as poor quality | 0 |
| Sequence length | 80-301 |
| %GC | 57 |

## Per base sequence quality

## Per tile sequence quality

## Per sequence quality scores

## Per base sequence content

## Per sequence GC content

## Per base N content

## Sequence Length Distribution

## Sequence Duplication Levels

## Overrepresented sequences

| Sequence | Count | Percentage | Possible Source |
| --- | --- | --- | --- |
| CCTACGGGAGGCTGCAGTGGGGAATATTGCACAATGGGCGCAAGCCTGAT | 25688 | 5.746846147478819 | No Hit |
| CCTACGGGTGGCTGCAGTGGGGAATATTGCACAATGGGCGCAAGCCTGAT | 25579 | 5.722460978136123 | No Hit |
| CCTACGGGGGGCTGCAGTGGGGAATATTGCACAATGGGCGCAAGCCTGAT | 24824 | 5.553554529936711 | No Hit |
| CCTACGGGGGGCAGCAGTGGGGAATATTGCACAATGGGCGCAAGCCTGAT | 22400 | 5.011264158499126 | No Hit |
| CCTACGGGAGGCAGCAGTGGGGAATATTGCACAATGGGCGCAAGCCTGAT | 20081 | 4.492464087804507 | No Hit |
| CCTACGGGCGGCTGCAGTGGGGAATATTGCACAATGGGCGCAAGCCTGAT | 18165 | 4.063822028532885 | No Hit |
| CCTACGGGTGGCAGCAGTGGGGAATATTGCACAATGGGCGCAAGCCTGAT | 17843 | 3.9917851062544605 | No Hit |
| CCTACGGGCGGCAGCAGTGGGGAATATTGCACAATGGGCGCAAGCCTGAT | 14608 | 3.268060126221216 | No Hit |
| CCTACGGGAGGCTGCAGTGGGGAATCTTGCGCAATGGGCGAAAGCCTGAC | 6809 | 1.5232900738937747 | No Hit |
| CCTACGGGTGGCTGCAGTGGGGAATCTTGCGCAATGGGCGAAAGCCTGAC | 6413 | 1.434698082520308 | No Hit |
| CCTACGGGGGGCTGCAGTGGGGAATCTTGCGCAATGGGCGAAAGCCTGAC | 6407 | 1.43335577962071 | No Hit |
| CCTACGGGGGGCAGCAGTGGGGAATCTTGCGCAATGGGCGAAAGCCTGAC | 6079 | 1.359976554442687 | No Hit |
| CCTACGGGAGGCAGCAGTGGGGAATCTTGCGCAATGGGCGAAAGCCTGAC | 5428 | 1.2143366898363062 | No Hit |
| CCTACGGGTGGCAGCAGTGGGGAATCTTGCGCAATGGGCGAAAGCCTGAC | 4740 | 1.0604192906824044 | No Hit |
| CCTACGGGCGGCTGCAGTGGGGAATCTTGCGCAATGGGCGAAAGCCTGAC | 4603 | 1.029770041141584 | No Hit |
| CCTACGGGCGGCAGCAGTGGGGAATCTTGCGCAATGGGCGAAAGCCTGAC | 3931 | 0.87943211638661 | No Hit |
| CCTACGGGTGGCTGCAGTGGGGAATATTGCACAATGGGCGAAAGCCTGAT | 3244 | 0.7257384343826413 | No Hit |
| CCTACGGGAGGCTGCAGTGGGGAATATTGCACAATGGGCGAAAGCCTGAT | 3188 | 0.7132102739863935 | No Hit |
| CCTACGGGGGGCTGCAGTGGGGAATATTGCACAATGGGCGAAAGCCTGAT | 3103 | 0.6941943162420888 | No Hit |
| CCTACGGGAGGCTGCAGTGGGGAATCTTAGACAATGGGGGCAACCCTGAT | 2784 | 0.6228285454134629 | No Hit |
| CCTACGGGTGGCTGCAGTGGGGAATCTTAGACAATGGGGGCAACCCTGAT | 2778 | 0.6214862425138649 | No Hit |
| CCTACGGGGGGCAGCAGTGGGGAATATTGCACAATGGGCGAAAGCCTGAT | 2720 | 0.608510647817751 | No Hit |
| CCTACGGGGGGCTGCAGTGGGGAATCTTAGACAATGGGGGCAACCCTGAT | 2663 | 0.5957587702715703 | No Hit |
| CCTACGGGTGGCTGCAGTGGGGAATATTGGACAATGGGCGAAAGCCTGAT | 2564 | 0.5736107724282036 | No Hit |
| CCTACGGGAGGCTGCAGTGGGGAATATTGGACAATGGGCGAAAGCCTGAT | 2549 | 0.5702550151792086 | No Hit |
| CCTACGGGGGGCAGCAGTGGGGAATCTTAGACAATGGGGGCAACCCTGAT | 2447 | 0.5474358658860429 | No Hit |
| CCTACGGGAGGCAGCAGTGGGGAATATTGCACAATGGGCGAAAGCCTGAT | 2445 | 0.5469884315861769 | No Hit |
| CCTACGGGGGGCTGCAGTGGGGAATATTGGACAATGGGCGAAAGCCTGAT | 2367 | 0.5295384938914032 | No Hit |
| CCTACGGGGGGCAGCAGTGGGGAATATTGGACAATGGGCGAAAGCCTGAT | 2350 | 0.5257353023425423 | No Hit |
| CCTACGGGTGGCTGCAGTGGGGAATATTGCGCAATGGGCGGAAGCCTGAC | 2277 | 0.5094039503974335 | No Hit |
| CCTACGGGTGGCTGCAGTGGGGAATATTGGACAATGGGCGCAAGCCTGAT | 2271 | 0.5080616474978356 | No Hit |
| CCTACGGGTGGCAGCAGTGGGGAATATTGCACAATGGGCGAAAGCCTGAT | 2265 | 0.5067193445982375 | No Hit |
| CCTACGGGAGGCTGCAGTGGGGAATATTGGACAATGGGCGCAAGCCTGAT | 2209 | 0.49419118420198976 | No Hit |
| CCTACGGGCGGCTGCAGTGGGGAATATTGCACAATGGGCGAAAGCCTGAT | 2205 | 0.4932963156022578 | No Hit |
| CCTACGGGAGGCAGCAGTGGGGAATCTTAGACAATGGGGGCAACCCTGAT | 2176 | 0.48680851825420085 | No Hit |
| CCTACGGGAGGCTGCAGTGGGGAATATTGCGCAATGGGCGGAAGCCTGAC | 2147 | 0.48032072090614397 | No Hit |
| CCTACGGGGGGCAGCAGTGGGGAATATTGCGCAATGGGCGGAAGCCTGAC | 2117 | 0.473609206408154 | No Hit |
| CCTACGGGAGGCAGCAGTGGGGAATATTGGACAATGGGCGAAAGCCTGAT | 2096 | 0.46891114625956115 | No Hit |
| CCTACGGGGGGCTGCAGTGGGGAATATTGGACAATGGGCGCAAGCCTGAT | 2066 | 0.4621996317615712 | No Hit |
| CCTACGGGGGGCTGCAGTGGGGAATATTGCGCAATGGGCGGAAGCCTGAC | 2014 | 0.4505663399650554 | No Hit |
| CCTACGGGAGGCTGCAGTGGGGAATATTGGACAATGGGGGGAACCCTGAT | 1997 | 0.44676314841619447 | No Hit |
| CCTACGGGCGGCTGCAGTGGGGAATCTTAGACAATGGGGGCAACCCTGAT | 1990 | 0.4451971283666634 | No Hit |
| CCTACGGGGGGCTGCAGTGGGGAATATTGGACAATGGGGGGAACCCTGAT | 1972 | 0.4411702196678695 | No Hit |
| CCTACGGGTGGCTGCAGTGGGGAATATTGGACAATGGGGGGAACCCTGAT | 1952 | 0.4366958766692096 | No Hit |
| CCTACGGGTGGCAGCAGTGGGGAATCTTAGACAATGGGGGCAACCCTGAT | 1950 | 0.4362484423693436 | No Hit |
| CCTACGGGGGGCAGCAGTGGGGAATATTGGACAATGGGCGCAAGCCTGAT | 1929 | 0.4315503822207507 | No Hit |
| CCTACGGGTGGCAGCAGTGGGGAATATTGGACAATGGGCGAAAGCCTGAT | 1884 | 0.42148311047376585 | No Hit |
| CCTACGGGCGGCTGCAGTGGGGAATATTGGACAATGGGCGAAAGCCTGAT | 1878 | 0.4201408075741678 | No Hit |
| CCTACGGGCGGCAGCAGTGGGGAATATTGCACAATGGGCGAAAGCCTGAT | 1773 | 0.3966505068312032 | No Hit |
| CCTACGGGAGGCAGCAGTGGGGAATATTGCGCAATGGGCGGAAGCCTGAC | 1765 | 0.39486076963173916 | No Hit |
| CCTACGGGGGGCAGCAGTGGGGAATATTGGACAATGGGGGGAACCCTGAT | 1734 | 0.3879255379838163 | No Hit |
| CCTACGGGCGGCAGCAGTGGGGAATCTTAGACAATGGGGGCAACCCTGAT | 1689 | 0.37785826623683144 | No Hit |
| CCTACGGGAGGCAGCAGTGGGGAATATTGGACAATGGGCGCAAGCCTGAT | 1688 | 0.3776345490868984 | No Hit |
| CCTACGGGTGGCTGCAGTGGGGAATTTTGGACAATGGGCGCAAGCCTGAT | 1674 | 0.3745025089878365 | No Hit |
| CCTACGGGCGGCTGCAGTGGGGAATATTGCGCAATGGGCGGAAGCCTGAC | 1609 | 0.35996089424219174 | No Hit |
| CCTACGGGCGGCTGCAGTGGGGAATATTGGACAATGGGCGCAAGCCTGAT | 1569 | 0.35101220824487184 | No Hit |
| CCTACGGGTGGCAGCAGTGGGGAATATTGCGCAATGGGCGGAAGCCTGAC | 1567 | 0.35056477394500585 | No Hit |
| CCTACGGGAGGCAGCAGTGGGGAATATTGGACAATGGGGGGAACCCTGAT | 1566 | 0.3503410567950728 | No Hit |
| CCTACGGGAGGCTGCAGTGGGGAATATTGCACAATGGGCGGAAGCCTGAT | 1522 | 0.340497502198021 | No Hit |
| CCTACGGGTGGCAGCAGTGGGGAATATTGGACAATGGGCGCAAGCCTGAT | 1505 | 0.33669431064916006 | No Hit |
| CCTACGGGCGGCAGCAGTGGGGAATATTGGACAATGGGCGAAAGCCTGAT | 1496 | 0.3346808562997631 | No Hit |
| CCTACGGGAGGCTGCAGTGGGGAATTTTGGACAATGGGCGCAAGCCTGAT | 1487 | 0.33266740195036615 | No Hit |
| CCTACGGGGGGCTGCAGTGGGGAATTTTGGACAATGGGCGCAAGCCTGAT | 1469 | 0.3286404932515722 | No Hit |
| CCTACGGGTGGCTGCAGTGGGGAATATTGCACAATGGGCGGAAGCCTGAT | 1436 | 0.3212578273037833 | No Hit |
| CCTACGGGGGGCTGCAGTGGGGAATATTGCACAATGGGCGGAAGCCTGAT | 1426 | 0.3190206558044533 | No Hit |
| CCTACGGGGGGCAGCAGTGGGGAATTTTGGACAATGGGCGCAAGCCTGAT | 1420 | 0.31767835290485535 | No Hit |
| CCTACGGGTGGCAGCAGTGGGGAATATTGGACAATGGGGGGAACCCTGAT | 1417 | 0.31700720145505634 | No Hit |
| CCTACGGGCGGCTGCAGTGGGGAATATTGGACAATGGGGGGAACCCTGAT | 1367 | 0.3058213439584065 | No Hit |
| CCTACGGGGGGCAGCAGTGGGGAATATTGCACAATGGGCGGAAGCCTGAT | 1294 | 0.2894899920132977 | No Hit |
| CCTACGGGCGGCAGCAGTGGGGAATATTGCGCAATGGGCGGAAGCCTGAC | 1275 | 0.2852393661645708 | No Hit |
| CCTACGGGAGGCAGCAGTGGGGAATTTTGGACAATGGGCGCAAGCCTGAT | 1252 | 0.2800938717161119 | No Hit |
| CCTACGGGCGGCAGCAGTGGGGAATATTGGACAATGGGCGCAAGCCTGAT | 1188 | 0.2657759741204001 | No Hit |
| CCTACGGGCGGCAGCAGTGGGGAATATTGGACAATGGGGGGAACCCTGAT | 1185 | 0.2651048226706011 | No Hit |
| CCTACGGGGGGCAGCAGTAGGGAATCTTCCGCAATGGACGAAAGTCTGAC | 1178 | 0.26353880262107016 | No Hit |
| CCTACGGGAGGCAGCAGTGGGGAATATTGCACAATGGGCGGAAGCCTGAT | 1163 | 0.2601830453720752 | No Hit |
| CCTACGGGTGGCAGCAGTGGGGAATTTTGGACAATGGGCGCAAGCCTGAT | 1161 | 0.2597356110722092 | No Hit |
| CCTACGGGCGGCTGCAGTGGGGAATTTTGGACAATGGGCGCAAGCCTGAT | 1148 | 0.2568272881230802 | No Hit |
| CCTACGGGAGGCAGCAGTAGGGAATCTTCCGCAATGGACGAAAGTCTGAC | 1139 | 0.2548138337736833 | No Hit |
| CCTACGGGGGGCAGCAGTAGGGAATCTTCCACAATGGACGAAAGTCTGAT | 1098 | 0.2456414306264304 | No Hit |
| CCTACGGGAGGCTGCAGTGGGGAATATTGCGCAATGGGCGAAAGCCTGAC | 1087 | 0.24318054197716743 | No Hit |
| CCTACGGGTGGCAGCAGTAGGGAATCTTCCGCAATGGACGAAAGTCTGAC | 1064 | 0.23803504752870852 | No Hit |
| CCTACGGGCGGCTGCAGTGGGGAATATTGCACAATGGGCGGAAGCCTGAT | 1055 | 0.23602159317931154 | No Hit |
| CCTACGGGTGGCTGCAGTGGGGAATATTGCGCAATGGGCGAAAGCCTGAC | 1048 | 0.23445557312978058 | No Hit |
| CCTACGGGGGGCTGCAGTGGGGAATATTGCGCAATGGGCGAAAGCCTGAC | 1039 | 0.2324421187803836 | No Hit |
| CCTACGGGGGGCAGCAGTGGGGAATATTGCGCAATGGGCGAAAGCCTGAC | 1031 | 0.23065238158091964 | No Hit |
| CCTACGGGAGGCAGCAGTAGGGAATCTTCCACAATGGACGAAAGTCTGAT | 997 | 0.22304599848319775 | No Hit |
| CCTACGGGTGGCAGCAGTGGGGAATATTGCACAATGGGCGGAAGCCTGAT | 992 | 0.22192741273353275 | No Hit |
| CCTACGGGAGGCTGCAGTAGGGAATCTTCCGCAATGGACGAAAGTCTGAC | 963 | 0.21543961538547582 | No Hit |
| CCTACGGGTGGCAGCAGTAGGGAATCTTCCACAATGGACGAAAGTCTGAT | 938 | 0.2098466866371509 | No Hit |
| CCTACGGGTGGCTGCAGTAGGGAATCTTCCGCAATGGACGAAAGTCTGAC | 911 | 0.20380632358896 | No Hit |
| CCTACGGGCGGCAGCAGTGGGGAATTTTGGACAATGGGCGCAAGCCTGAT | 906 | 0.202687737839295 | No Hit |
| CCTACGGGGGGCTGCAGTAGGGAATCTTCCGCAATGGACGAAAGTCTGAC | 894 | 0.20000313204009906 | No Hit |
| CCTACGGGCGGCAGCAGTAGGGAATCTTCCGCAATGGACGAAAGTCTGAC | 868 | 0.19418648614184114 | No Hit |
| CCTACGGGTGGCAGCAGTAGGGAATTTTCCGCAATGGGCGAAAGCCTGAC | 864 | 0.19329161754210916 | No Hit |
| CCTACGGGAGGCAGCAGTAGGGAATTTTCCGCAATGGGCGAAAGCCTGAC | 861 | 0.19262046609231018 | No Hit |
| CCTACGGGGGGCAGCAGTAGGGAATTTTCCGCAATGGGCGAAAGCCTGAC | 860 | 0.19239674894237715 | No Hit |
| CCTACGGGGGGCAGCAGTAGGGAATCTTCCGCAATGGGCGAAAGCCTGAC | 858 | 0.1919493146425112 | No Hit |
| CCTACGGGAGGCAGCAGTGGGGAATATTGCGCAATGGGCGAAAGCCTGAC | 838 | 0.18747497164385124 | No Hit |
| CCTACGGGAGGCTGCAGTAGGGAATCTTCCACAATGGACGAAAGTCTGAT | 828 | 0.1852378001445213 | No Hit |
| CCTACGGGTGGCAGCAGTGGGGAATATTGCGCAATGGGCGAAAGCCTGAC | 817 | 0.18277691149525832 | No Hit |
| CCTACGGGCGGCTGCAGTGGGGAATATTGCGCAATGGGCGAAAGCCTGAC | 801 | 0.17919743709633038 | No Hit |
| CCTACGGGCGGCAGCAGTGGGGAATATTGCACAATGGGCGGAAGCCTGAT | 789 | 0.1765128312971344 | No Hit |
| CCTACGGGAGGCAGCAGTAGGGAATCTTCCGCAATGGGCGAAAGCCTGAC | 787 | 0.1760653969972684 | No Hit |
| CCTACGGGGGGCTGCAGTAGGGAATTTTCCGCAATGGGCGAAAGCCTGAC | 771 | 0.17248592259834047 | No Hit |
| CCTACGGGGGGCTGCAGTAGGGAATCTTCCACAATGGACGAAAGTCTGAT | 764 | 0.17091990254880948 | No Hit |
| CCTACGGGTGGCTGCAGTAGGGAATCTTCCACAATGGACGAAAGTCTGAT | 760 | 0.1700250339490775 | No Hit |
| CCTACGGGTGGCAGCAGTAGGGAATCTTCCGCAATGGGCGAAAGCCTGAC | 742 | 0.16599812525028354 | No Hit |
| CCTACGGGCGGCAGCAGTAGGGAATCTTCCACAATGGACGAAAGTCTGAT | 729 | 0.1630898023011546 | No Hit |
| CCTACGGGTGGCTGCAGTAGGGAATTTTCCGCAATGGGCGAAAGCCTGAC | 729 | 0.1630898023011546 | No Hit |
| CCTACGGGAGGCTGCAGTAGGGAATTTTCCGCAATGGGCGAAAGCCTGAC | 716 | 0.16018147935202565 | No Hit |
| CCTACGGGTGGCTGCAGTAGGGAATCTTCCGCAATGGGCGAAAGCCTGAC | 684 | 0.15302253055416976 | No Hit |
| CCTACGGGAGGCTGCAGTAGGGAATCTTCCGCAATGGGCGAAAGCCTGAC | 672 | 0.1503379247549738 | No Hit |
| CCTACGGGCGGCAGCAGTGGGGAATATTGCGCAATGGGCGAAAGCCTGAC | 662 | 0.14810075325564384 | No Hit |
| CCTACGGGCGGCTGCAGTAGGGAATCTTCCGCAATGGACGAAAGTCTGAC | 660 | 0.14765331895577782 | No Hit |
| CCTACGGGGGGCTGCAGTAGGGAATCTTCCGCAATGGGCGAAAGCCTGAC | 639 | 0.1429552588071849 | No Hit |
| CCTACGGGGGGCAGCAGTAGGGAATATTGGGCAATGGACGAGAGTCTGAC | 626 | 0.14004693585805594 | No Hit |
| CCTACGGGAGGCAGCAGTAGGGAATATTGGGCAATGGACGAGAGTCTGAC | 581 | 0.1299796641110711 | No Hit |
| CCTACGGGCGGCAGCAGTAGGGAATTTTCCGCAATGGGCGAAAGCCTGAC | 575 | 0.1286373612114731 | No Hit |
| CCTACGGGCGGCAGCAGTAGGGAATCTTCCGCAATGGGCGAAAGCCTGAC | 555 | 0.12416301821281317 | No Hit |
| CCTACGGGGGGCTGCAGTGGGGAATATTGGACAATGGGCGGAAGCCTGAT | 550 | 0.12304443246314818 | No Hit |
| CCTACGGGCGGCTGCAGTAGGGAATCTTCCACAATGGACGAAAGTCTGAT | 548 | 0.1225969981632822 | No Hit |
| CCTACGGGAGGCTGCAGTGGGGAATATTGGACAATGGGCGGAAGCCTGAT | 545 | 0.12192584671348321 | No Hit |
| CCTACGGGCGGCTGCAGTAGGGAATTTTCCGCAATGGGCGAAAGCCTGAC | 524 | 0.11722778656489029 | No Hit |
| CCTACGGGTGGCAGCAGTAGGGAATATTGGGCAATGGACGAGAGTCTGAC | 513 | 0.11476689791562732 | No Hit |
| CCTACGGGTGGCTGCAGTGGGGAATATTGGACAATGGGCGGAAGCCTGAT | 512 | 0.11454318076569432 | No Hit |
| CCTACGGGCGGCTGCAGTAGGGAATCTTCCGCAATGGGCGAAAGCCTGAC | 494 | 0.11051627206690037 | No Hit |
| CCTACGGGGGGCAGCAGTGGGGAATATTGGACAATGGGCGGAAGCCTGAT | 488 | 0.1091739691673024 | No Hit |
| CCTACGGGAGGCTGCAGTAGGGAATATTGGGCAATGGACGAGAGTCTGAC | 486 | 0.10872653486743639 | No Hit |
| CCTACGGGTGGCTGCAGTGGGGAATTTTCCGCAATGGGCGAAAGCCTGAC | 472 | 0.10559449476837444 | No Hit |
| CCTACGGGAGGCTGCAGTGAGGAATTTTCCGCAATGGGCGAAAGCCTGAC | 470 | 0.10514706046850847 | No Hit |
| CCTACGGGGGGCAGCAGTGGGGAATTTTCCGCAATGGGCGAAAGCCTGAC | 465 | 0.10402847471884347 | No Hit |
| CCTACGGGAGGCAGCAGTGGGGAATTTTCCGCAATGGGCGAAAGCCTGAC | 464 | 0.10380475756891047 | No Hit |
| CCTACGGGGGGCTGCAGTAGGGAATATTGGGCAATGGACGAGAGTCTGAC | 458 | 0.1024624546693125 | No Hit |
| CCTACGGGTGGCTGCAGTGAGGAATTTTCCGCAATGGGCGAAAGCCTGAC | 455 | 0.1017913032195135 | No Hit |
| CCTACGGGGGGCTGCAGTGAGGAATTTTCCGCAATGGGCGAAAGCCTGAC | 450 | 0.10067271746984853 | No Hit |
| CCTACGGGTGGCAGCAGTGGGGAATATTGGACAATGGGCGGAAGCCTGAT | 447 | 0.10000156602004953 | No Hit |

## Adapter Content

## Kmer Content

| Sequence | Count | PValue | Obs/Exp Max | Max Obs/Exp Position |
| --- | --- | --- | --- | --- |
| ATCCATA | 10 | 5.695243E-4 | 336.70422 | 295 |
| GTATCAG | 20 | 3.194873E-8 | 336.70422 | 295 |
| ATTTGAG | 15 | 4.268468E-6 | 336.70422 | 295 |
| ATGTGAT | 100 | 0.0 | 336.7042 | 295 |
| GAGAGAG | 2620 | 0.0 | 333.4914 | 295 |
| AGTGTTG | 535 | 0.0 | 324.11713 | 295 |
| AGTTCGG | 9765 | 0.0 | 319.46384 | 295 |
| AGGTATT | 195 | 0.0 | 319.43735 | 295 |
| ATTTGTG | 225 | 0.0 | 314.2573 | 295 |
| ACAGTAG | 205 | 0.0 | 312.06732 | 295 |
| AGGTATG | 395 | 0.0 | 311.13174 | 295 |
| ATCCAAA | 315 | 0.0 | 309.98166 | 295 |
| ATTCGAA | 325 | 0.0 | 305.62384 | 295 |
| AGTGTGG | 870 | 0.0 | 298.0026 | 295 |
| CGGAGAG | 210 | 0.0 | 296.62036 | 295 |
| TGGTAGG | 40 | 0.0 | 294.61618 | 295 |
| GGGAGAG | 715 | 0.0 | 294.32187 | 295 |
| CCTACTG | 15 | 7.3446845E-6 | 293.91464 | 1 |
| ACGTCAT | 15 | 7.3446845E-6 | 293.91464 | 3 |
| CCGGTCA | 10 | 8.557244E-4 | 293.91464 | 2 |

Produced by FastQC (version 0.11.7)
