# Supplementary material for: Assessment of airborne bacteria from a public health institution in Mexico City
Source: PLOS Glob Public Health. 2024 Nov 7;4(11):e0003672. doi: 10.1371/journal.pgph.0003672 (PMC11542838; doi:10.1371/journal.pgph.0003672)
Supplement: S1 Text — (ZIP) [file pgph.0003672.s001.zip › Hospital_16S_QC/21022023_CED2_16S_S41_L001_R2_001_fastqc.html]

21022023\_CED2\_16S\_S41\_L001\_R2\_001.fastq.gz FastQC Report 

FastQC Report

Tue 14 Mar 2023  
21022023\_CED2\_16S\_S41\_L001\_R2\_001.fastq.gz

## Summary

- Basic Statistics
- Per base sequence quality
- Per tile sequence quality
- Per sequence quality scores
- Per base sequence content
- Per sequence GC content
- Per base N content
- Sequence Length Distribution
- Sequence Duplication Levels
- Overrepresented sequences
- Adapter Content
- Kmer Content

## Basic Statistics

| Measure | Value |
| --- | --- |
| Filename | 21022023\_CED2\_16S\_S41\_L001\_R2\_001.fastq.gz |
| File type | Conventional base calls |
| Encoding | Sanger / Illumina 1.9 |
| Total Sequences | 446993 |
| Sequences flagged as poor quality | 0 |
| Sequence length | 80-301 |
| %GC | 56 |

## Per base sequence quality

## Per tile sequence quality

## Per sequence quality scores

## Per base sequence content

## Per sequence GC content

## Per base N content

## Sequence Length Distribution

## Sequence Duplication Levels

## Overrepresented sequences

| Sequence | Count | Percentage | Possible Source |
| --- | --- | --- | --- |
| GACTACTGGGGTATCTAATCCTGTTCGCTCCCCACGCTTTCGCTCCTCAG | 27743 | 6.206584890591127 | No Hit |
| GACTACAGGGGTATCTAATCCTGTTCGCTCCCCACGCTTTCGCTCCTCAG | 24293 | 5.434760723322289 | No Hit |
| GACTACTAGGGTATCTAATCCTGTTCGCTCCCCACGCTTTCGCTCCTCAG | 23581 | 5.275474112569996 | No Hit |
| GACTACCGGGGTATCTAATCCTGTTCGCTCCCCACGCTTTCGCTCCTCAG | 23252 | 5.20187117024204 | No Hit |
| GACTACTCGGGTATCTAATCCTGTTCGCTCCCCACGCTTTCGCTCCTCAG | 22059 | 4.934976610371974 | No Hit |
| GACTACCAGGGTATCTAATCCTGTTCGCTCCCCACGCTTTCGCTCCTCAG | 21008 | 4.6998498857923945 | No Hit |
| GACTACAAGGGTATCTAATCCTGTTCGCTCCCCACGCTTTCGCTCCTCAG | 21004 | 4.698955017192663 | No Hit |
| GACTACCCGGGTATCTAATCCTGTTCGCTCCCCACGCTTTCGCTCCTCAG | 18738 | 4.192011955444492 | No Hit |
| GACTACACGGGTATCTAATCCTGTTCGCTCCCCACGCTTTCGCTCCTCAG | 18733 | 4.190893369694828 | No Hit |
| GACTACTGGGGTATCTAATCCTGTTCGCTCCCCATGCTTTCGCTCCTCAG | 6424 | 1.437158971169571 | No Hit |
| GACTACAGGGGTATCTAATCCTGTTCGCTCCCCATGCTTTCGCTCCTCAG | 5521 | 1.2351423847800749 | No Hit |
| GACTACTAGGGTATCTAATCCTGTTCGCTCCCCATGCTTTCGCTCCTCAG | 5268 | 1.1785419458470268 | No Hit |
| GACTACCGGGGTATCTAATCCTGTTCGCTCCCCATGCTTTCGCTCCTCAG | 5178 | 1.158407402353057 | No Hit |
| GACTACTCGGGTATCTAATCCTGTTCGCTCCCCATGCTTTCGCTCCTCAG | 5144 | 1.150801019255335 | No Hit |
| GACTACTGGGGTATCTAATCCTGTTTGCTCCCCACGCTTTCGCACCTCAG | 5000 | 1.1185857496649836 | No Hit |
| GACTACAAGGGTATCTAATCCTGTTCGCTCCCCATGCTTTCGCTCCTCAG | 4820 | 1.0783166626770442 | No Hit |
| GACTACCAGGGTATCTAATCCTGTTCGCTCCCCATGCTTTCGCTCCTCAG | 4794 | 1.0725000167787861 | No Hit |
| GACTACAGGGGTATCTAATCCTGTTTGCTCCCCACGCTTTCGCACCTCAG | 4417 | 0.9881586512540464 | No Hit |
| GACTACCCGGGTATCTAATCCTGTTCGCTCCCCATGCTTTCGCTCCTCAG | 4261 | 0.9532587758644989 | No Hit |
| GACTACCGGGGTATCTAATCCTGTTTGCTCCCCACGCTTTCGCACCTCAG | 4174 | 0.9337953838203282 | No Hit |
| GACTACTAGGGTATCTAATCCTGTTTGCTCCCCACGCTTTCGCACCTCAG | 4160 | 0.9306633437212664 | No Hit |
| GACTACACGGGTATCTAATCCTGTTCGCTCCCCATGCTTTCGCTCCTCAG | 4153 | 0.9290973236717354 | No Hit |
| GACTACTCGGGTATCTAATCCTGTTTGCTCCCCACGCTTTCGCACCTCAG | 4019 | 0.8991192255807138 | No Hit |
| GACTACCAGGGTATCTAATCCTGTTTGCTCCCCACGCTTTCGCACCTCAG | 3827 | 0.8561655327935784 | No Hit |
| GACTACTGGGGTATCTAATCCTGTTTGCTCCCCACGCTTTCGCGCCTCAG | 3825 | 0.8557180984937124 | No Hit |
| GACTACAAGGGTATCTAATCCTGTTTGCTCCCCACGCTTTCGCACCTCAG | 3741 | 0.8369258578993406 | No Hit |
| GACTACAGGGGTATCTAATCCTGTTTGCTCCCCACGCTTTCGCGCCTCAG | 3498 | 0.7825625904656224 | No Hit |
| GACTACTAGGGTATCTAATCCTGTTTGCTCCCCACGCTTTCGCGCCTCAG | 3418 | 0.7646652184709828 | No Hit |
| GACTACCCGGGTATCTAATCCTGTTTGCTCCCCACGCTTTCGCACCTCAG | 3366 | 0.753031926674467 | No Hit |
| GACTACACGGGTATCTAATCCTGTTTGCTCCCCACGCTTTCGCACCTCAG | 3364 | 0.752584492374601 | No Hit |
| GACTACCGGGGTATCTAATCCTGTTTGCTCCCCACGCTTTCGCGCCTCAG | 3246 | 0.7261858686825073 | No Hit |
| GACTACTCGGGTATCTAATCCTGTTTGCTCCCCACGCTTTCGCGCCTCAG | 3126 | 0.6993398106905477 | No Hit |
| GACTACAAGGGTATCTAATCCTGTTTGCTCCCCACGCTTTCGCGCCTCAG | 3052 | 0.682784741595506 | No Hit |
| GACTACCAGGGTATCTAATCCTGTTTGCTCCCCACGCTTTCGCGCCTCAG | 3008 | 0.6729411869984542 | No Hit |
| GACTACCCGGGTATCTAATCCTGTTTGCTCCCCACGCTTTCGCGCCTCAG | 2795 | 0.6252894340627259 | No Hit |
| GACTACACGGGTATCTAATCCTGTTTGCTCCCCACGCTTTCGCGCCTCAG | 2728 | 0.6103003850172151 | No Hit |
| GACTACTGGGGTATCTAATCCTGTTTGCTCCCCACGCTTTCGTGCATGAG | 1811 | 0.40515175852865704 | No Hit |
| GACTACTGGGGTATCTAATCCTGTTTGCTCCCCATGCTTTCGCACCTCAG | 1794 | 0.4013485669797961 | No Hit |
| GACTACCGGGGTATCTAATCCTGTTTGCTCCCCATGCTTTCGCACCTCAG | 1574 | 0.3521307939945368 | No Hit |
| GACTACAGGGGTATCTAATCCTGTTTGCTCCCCATGCTTTCGCACCTCAG | 1563 | 0.34966990534527387 | No Hit |
| GACTACAGGGGTATCTAATCCTGTTTGCTCCCCACGCTTTCGTGCATGAG | 1559 | 0.3487750367455419 | No Hit |
| GACTACTCGGGTATCTAATCCTGTTTGCTCCCCACGCTTTCGTGCATGAG | 1559 | 0.3487750367455419 | No Hit |
| GACTACCGGGGTATCTAATCCTGTTTGCTCCCCACGCTTTCGTGCATGAG | 1505 | 0.33669431064916006 | No Hit |
| GACTACTAGGGTATCTAATCCTGTTTGCTCCCCACGCTTTCGTGCATGAG | 1479 | 0.33087766475090213 | No Hit |
| GACTACTAGGGTATCTAATCCTGTTTGCTCCCCATGCTTTCGCACCTCAG | 1477 | 0.33043023045103614 | No Hit |
| GACTACTCGGGTATCTAATCCTGTTTGCTCCCCATGCTTTCGCACCTCAG | 1411 | 0.3156648985554583 | No Hit |
| GACTACAAGGGTATCTAATCCTGTTTGCTCCCCACGCTTTCGTGCATGAG | 1362 | 0.3047027582087415 | No Hit |
| GACTACCAGGGTATCTAATCCTGTTTGCTCCCCACGCTTTCGTGCATGAG | 1327 | 0.29687265796108664 | No Hit |
| GACTACAAGGGTATCTAATCCTGTTTGCTCCCCATGCTTTCGCACCTCAG | 1318 | 0.29485920361168966 | No Hit |
| GACTACCAGGGTATCTAATCCTGTTTGCTCCCCATGCTTTCGCACCTCAG | 1293 | 0.28926627486336476 | No Hit |
| GACTACCCGGGTATCTAATCCTGTTTGCTCCCCACGCTTTCGTGCATGAG | 1216 | 0.27204005431852396 | No Hit |
| GACTACACGGGTATCTAATCCTGTTTGCTCCCCACGCTTTCGTGCATGAG | 1198 | 0.26801314561973005 | No Hit |
| GACTACCCGGGTATCTAATCCTGTTTGCTCCCCATGCTTTCGCACCTCAG | 1179 | 0.2637625197710031 | No Hit |
| GACTACACGGGTATCTAATCCTGTTTGCTCCCCATGCTTTCGCACCTCAG | 1137 | 0.2543663994738173 | No Hit |
| GACTACTGGGGTATCTAATCCTGTTCGCTCCCCACGCTTTCGTGCCTCAG | 1122 | 0.25101064222482233 | No Hit |
| GACTACAGGGGTATCTAATCCTGTTCGCTCCCCACGCTTTCGTGCCTCAG | 993 | 0.22215112988346575 | No Hit |
| GACTACCGGGGTATCTAATCCTGTTCGCTCCCCACGCTTTCGTGCCTCAG | 968 | 0.21655820113514082 | No Hit |
| GACTACTGGGGTATCTAATCCTGTTCGCTCCCCATGCTTTCGCTTCTCAG | 953 | 0.21320244388614587 | No Hit |
| GACTACCAGGGTATCTAATCCTGTTCGCTCCCCACGCTTTCGTGCCTCAG | 941 | 0.21051783808694993 | No Hit |
| GACTACTCGGGTATCTAATCCTGTTCGCTCCCCACGCTTTCGTGCCTCAG | 924 | 0.206714646538089 | No Hit |
| GACTACTAGGGTATCTAATCCTGTTCGCTCCCCACGCTTTCGTGCCTCAG | 913 | 0.204253757888826 | No Hit |
| GACTACTGGGGTATCTAATCCTGTTTGCTCCCCACGCTTTCGAGCCTCAG | 867 | 0.19396276899190815 | No Hit |
| GACTACAAGGGTATCTAATCCTGTTCGCTCCCCACGCTTTCGTGCCTCAG | 851 | 0.1903832945929802 | No Hit |
| GACTACTAGGGTATCTAATCCTGTTCGCTCCCCATGCTTTCGCTTCTCAG | 812 | 0.18165832574559335 | No Hit |
| GACTACAGGGGTATCTAATCCTGTTCGCTCCCCATGCTTTCGCTTCTCAG | 809 | 0.18098717429579433 | No Hit |
| GACTACCCGGGTATCTAATCCTGTTCGCTCCCCACGCTTTCGTGCCTCAG | 784 | 0.17539424554746944 | No Hit |
| GACTACAGGGGTATCTAATCCTGTTTGCTCCCCACGCTTTCGAGCCTCAG | 780 | 0.17449937694773743 | No Hit |
| GACTACCGGGGTATCTAATCCTGTTCGCTCCCCATGCTTTCGCTTCTCAG | 768 | 0.1718147711485415 | No Hit |
| GACTACTGGGGTATCTAATCCTGTTCGCTACCCATGCTTTCGCTCCTCAG | 736 | 0.16465582235068557 | No Hit |
| GACTACTAGGGTATCTAATCCTGTTTGCTCCCCACGCTTTCGAGCCTCAG | 735 | 0.1644321052007526 | No Hit |
| GACTACACGGGTATCTAATCCTGTTCGCTCCCCACGCTTTCGTGCCTCAG | 725 | 0.1621949337014226 | No Hit |
| GACTACCGGGGTATCTAATCCTGTTCGCTCCCCACACTTTCGCTCCTCAG | 712 | 0.15928661075229367 | No Hit |
| GACTACCAGGGTATCTAATCCTGTTCGCTCCCCATGCTTTCGCTTCTCAG | 709 | 0.15861545930249465 | No Hit |
| GACTACTCGGGTATCTAATCCTGTTCGCTCCCCATGCTTTCGCTTCTCAG | 703 | 0.15727315640289669 | No Hit |
| GACTACCAGGGTATCTAATCCTGTTTGCTCCCCACGCTTTCGAGCCTCAG | 694 | 0.15525970205349973 | No Hit |
| GACTACAAGGGTATCTAATCCTGTTCGCTCCCCATGCTTTCGCTTCTCAG | 690 | 0.15436483345376775 | No Hit |
| GACTACAAGGGTATCTAATCCTGTTTGCTCCCCACGCTTTCGAGCCTCAG | 673 | 0.15056164190490678 | No Hit |
| GACTACTGGGGTATCTAATCCTGTTCGCTCCCCACACTTTCGCTCCTCAG | 670 | 0.1498904904551078 | No Hit |
| GACTACAGGGGTATCTAATCCTGTTCGCTACCCATGCTTTCGCTCCTCAG | 670 | 0.1498904904551078 | No Hit |
| GACTACCGGGGTATCTAATCCTGTTTGCTCCCCACGCTTTCGAGCCTCAG | 668 | 0.1494430561552418 | No Hit |
| GACTACAGGGGTATCTAATCCTGTTCGCTCCCCACACTTTCGCTCCTCAG | 667 | 0.14921933900530882 | No Hit |
| GACTACTCGGGTATCTAATCCTGTTTGCTCCCCACGCTTTCGAGCCTCAG | 661 | 0.14787703610571085 | No Hit |
| GACTACTAGGGTATCTAATCCTGTTCGCTACCCATGCTTTCGCTCCTCAG | 658 | 0.14720588465591183 | No Hit |
| GACTACCCGGGTATCTAATCCTGTTCGCTCCCCATGCTTTCGCTTCTCAG | 649 | 0.14519243030651488 | No Hit |
| GACTACTAGGGTATCTAATCCTGTTCGCTCCCCACACTTTCGCTCCTCAG | 636 | 0.1422841073573859 | No Hit |
| GACTACACGGGTATCTAATCCTGTTCGCTCCCCATGCTTTCGCTTCTCAG | 629 | 0.14071808730785493 | No Hit |
| GACTACCCGGGTATCTAATCCTGTTTGCTCCCCACGCTTTCGAGCCTCAG | 596 | 0.13333542136006604 | No Hit |
| GACTACCGGGGTATCTAATCCTGTTCGCTACCCATGCTTTCGCTCCTCAG | 595 | 0.13311170421013305 | No Hit |
| GACTACTGGGGTATCTAATCCTGTTTGCTCCCCACGCTTTCGCACCTGAG | 595 | 0.13311170421013305 | No Hit |
| GACTACTCGGGTATCTAATCCTGTTCGCTCCCCACACTTTCGCTCCTCAG | 583 | 0.13042709841093708 | No Hit |
| GACTACAAGGGTATCTAATCCTGTTCGCTCCCCACACTTTCGCTCCTCAG | 582 | 0.1302033812610041 | No Hit |
| GACTACTCGGGTATCTAATCCTGTTTGCTCCCCACGCTTTCGCACCTGAG | 576 | 0.12886107836140612 | No Hit |
| GACTACCAGGGTATCTAATCCTGTTCGCTCCCCACACTTTCGCTCCTCAG | 572 | 0.12796620976167414 | No Hit |
| GACTACTGGGGTATCTAATCCTGTTTGCTCCCCACGCTGTCGCGCCTCAG | 564 | 0.12617647256221015 | No Hit |
| GACTACACGGGTATCTAATCCTGTTTGCTCCCCACGCTTTCGAGCCTCAG | 560 | 0.12528160396247817 | No Hit |
| GACTACCCGGGTATCTAATCCTGTTCGCTACCCATGCTTTCGCTCCTCAG | 546 | 0.1221495638634162 | No Hit |
| GACTACCGGGGTATCTAATCCTGTTTGCTCCCCACGCTTTCGCACCTGAG | 537 | 0.12013610951401923 | No Hit |
| GACTACTCGGGTATCTAATCCTGTTCGCTACCCATGCTTTCGCTCCTCAG | 525 | 0.11745150371482327 | No Hit |
| GACTACCAGGGTATCTAATCCTGTTCGCTACCCATGCTTTCGCTCCTCAG | 520 | 0.1163329179651583 | No Hit |
| GACTACTAGGGTATCTAATCCTGTTTGCTCCCCACGCTTTCGCACCTGAG | 515 | 0.11521433221549332 | No Hit |
| GACTACCCGGGTATCTAATCCTGTTCGCTCCCCACACTTTCGCTCCTCAG | 512 | 0.11454318076569432 | No Hit |
| GACTACAGGGGTATCTAATCCTGTTTGCTCCCCACGCTTTCGCACCTGAG | 510 | 0.11409574646582832 | No Hit |
| GACTACTGGGGTATCTAATCCTGTTTGCTCCCCACGCTTTCGTGCCTCAG | 507 | 0.11342459501602935 | No Hit |
| GACTACAGGGGTATCTAATCCTGTTTGCTCCCCACGCTGTCGCGCCTCAG | 506 | 0.11320087786609632 | No Hit |
| GACTACAAGGGTATCTAATCCTGTTTGCTCCCCACGCTTTCGCACCTGAG | 492 | 0.11006883776703438 | No Hit |
| GACTACCAGGGTATCTAATCCTGTTTGCTCCCCACGCTTTCGCACCTGAG | 489 | 0.10939768631723538 | No Hit |
| GACTACACGGGTATCTAATCCTGTTCGCTACCCATGCTTTCGCTCCTCAG | 484 | 0.10827910056757041 | No Hit |
| GACTACACGGGTATCTAATCCTGTTCGCTCCCCACACTTTCGCTCCTCAG | 479 | 0.10716051481790544 | No Hit |
| GACTACCGGGGTATCTAATCCTGTTTGCTCCCCACGCTGTCGCGCCTCAG | 469 | 0.10492334331857545 | No Hit |
| GACTACTCGGGTATCTAATCCTGTTTGCTCCCCACGCTGTCGCGCCTCAG | 468 | 0.10469962616864247 | No Hit |
| GACTACTAGGGTATCTAATCCTGTTTGCTCCCCACGCTGTCGCGCCTCAG | 466 | 0.10425219186877648 | No Hit |
| GACTACACGGGTATCTAATCCTGTTTGCTCCCCACGCTTTCGCACCTGAG | 464 | 0.10380475756891047 | No Hit |
| GACTACTGGGGTATCTAATCCTGTTTGATCCCCACGCTTTCGCACATCAG | 464 | 0.10380475756891047 | No Hit |
| GACTACAAGGGTATCTAATCCTGTTCGCTACCCATGCTTTCGCTCCTCAG | 460 | 0.10290988896917849 | No Hit |
| GACTACAGGGGTATCTAATCCTGTTTGCTCCCCACGCTTTCGTGCCTCAG | 454 | 0.10156758606958052 | No Hit |
| GACTACTAGGGTATCTAATCCTGTTTGCTCCCCACGCTTTCGTGCCTCAG | 447 | 0.10000156602004953 | No Hit |

## Adapter Content

## Kmer Content

| Sequence | Count | PValue | Obs/Exp Max | Max Obs/Exp Position |
| --- | --- | --- | --- | --- |
| ACTTCGG | 5 | 1.10813955E-4 | 8583.614 | 295 |
| GTTAGTG | 10 | 3.419882E-8 | 8583.614 | 295 |
| GTTAGGG | 45 | 0.0 | 8583.614 | 295 |
| TTTTTTG | 5 | 1.10813955E-4 | 8583.614 | 295 |
| AGATTGG | 5 | 1.10813955E-4 | 8583.614 | 295 |
| GTAAGCG | 5 | 1.10813955E-4 | 8583.614 | 295 |
| GATAGGG | 5 | 1.10813955E-4 | 8583.614 | 295 |
| GATAGCG | 15 | 9.094947E-12 | 8583.614 | 295 |
| GGTAGCG | 10 | 3.419882E-8 | 8583.614 | 295 |
| GTTAGCG | 410 | 0.0 | 8374.258 | 295 |
| GTTAGCA | 40 | 0.0 | 7510.6626 | 295 |
| TTAGCCG | 490 | 0.0 | 7357.384 | 295 |
| AGTTGGG | 165 | 0.0 | 6502.738 | 295 |
| TTAGGCG | 35 | 0.0 | 6131.1533 | 295 |
| GTTGGCG | 70 | 0.0 | 6131.1533 | 295 |
| GTTTGTG | 10 | 4.4322162E-4 | 4291.807 | 295 |
| TTAGCGG | 10 | 4.4322162E-4 | 4291.807 | 295 |
| GTTAGAG | 30 | 1.6007107E-10 | 4291.807 | 295 |
| TTATCCG | 10 | 4.4322162E-4 | 4291.807 | 295 |
| AGAAAGG | 10 | 4.4322162E-4 | 4291.807 | 295 |

Produced by FastQC (version 0.11.7)
