# Supplementary material for: Assessment of airborne bacteria from a public health institution in Mexico City
Source: PLOS Glob Public Health. 2024 Nov 7;4(11):e0003672. doi: 10.1371/journal.pgph.0003672 (PMC11542838; doi:10.1371/journal.pgph.0003672)
Supplement: S1 Text — (ZIP) [file pgph.0003672.s001.zip › Hospital_16S_QC/21022023_CED3_16S_S42_L001_R1_001_fastqc.html]

21022023\_CED3\_16S\_S42\_L001\_R1\_001.fastq.gz FastQC Report 

FastQC Report

Tue 14 Mar 2023  
21022023\_CED3\_16S\_S42\_L001\_R1\_001.fastq.gz

## Summary

- Basic Statistics
- Per base sequence quality
- Per tile sequence quality
- Per sequence quality scores
- Per base sequence content
- Per sequence GC content
- Per base N content
- Sequence Length Distribution
- Sequence Duplication Levels
- Overrepresented sequences
- Adapter Content
- Kmer Content

## Basic Statistics

| Measure | Value |
| --- | --- |
| Filename | 21022023\_CED3\_16S\_S42\_L001\_R1\_001.fastq.gz |
| File type | Conventional base calls |
| Encoding | Sanger / Illumina 1.9 |
| Total Sequences | 7755 |
| Sequences flagged as poor quality | 0 |
| Sequence length | 186-301 |
| %GC | 54 |

## Per base sequence quality

## Per tile sequence quality

## Per sequence quality scores

## Per base sequence content

## Per sequence GC content

## Per base N content

## Sequence Length Distribution

## Sequence Duplication Levels

## Overrepresented sequences

| Sequence | Count | Percentage | Possible Source |
| --- | --- | --- | --- |
| CCTACGGGAGGCAGCAGTAGGGAATCTTCCGCAATGGACGAAAGTCTGAC | 249 | 3.210831721470019 | No Hit |
| CCTACGGGTGGCAGCAGTAGGGAATCTTCCGCAATGGACGAAAGTCTGAC | 232 | 2.991618310767247 | No Hit |
| CCTACGGGGGGCAGCAGTAGGGAATCTTCCGCAATGGACGAAAGTCTGAC | 226 | 2.9142488716956803 | No Hit |
| CCTACGGGCGGCAGCAGTAGGGAATCTTCCGCAATGGACGAAAGTCTGAC | 178 | 2.2952933591231464 | No Hit |
| CCTACGGGAGGCTGCAGTAGGGAATCTTCCGCAATGGACGAAAGTCTGAC | 162 | 2.0889748549323017 | No Hit |
| CCTACGGGTGGCTGCAGTAGGGAATCTTCCGCAATGGACGAAAGTCTGAC | 148 | 1.908446163765313 | No Hit |
| CTTGGTCATTTAGAGGAAGTAAAAGTCGTAACAAGGTTTCCGTAGGTGAA | 130 | 1.6763378465506125 | No Hit |
| CCTACGGGTGGCTGCAGTGGGGAATATTGCACAATGGGCGAAAGCCTGAT | 128 | 1.650548033526757 | No Hit |
| CCTACGGGAGGCAGCAGTGGGGAATATTGCACAATGGGCGAAAGCCTGAT | 127 | 1.6376531270148291 | No Hit |
| CCTACGGGGGGCTGCAGTAGGGAATCTTCCGCAATGGACGAAAGTCTGAC | 124 | 1.598968407479046 | No Hit |
| CCTACGGGAGGCAGCAGTGGGGAATATTGCACAATGGGCGCAAGCCTGAT | 114 | 1.4700193423597678 | No Hit |
| CCTACGGGAGGCTGCAGTGGGGAATATTGCACAATGGGCGAAAGCCTGAT | 112 | 1.4442295293359122 | No Hit |
| CCTACGGGTGGCAGCAGTGGGGAATATTGGACAATGGGCGAAAGCCTGAT | 106 | 1.3668600902643455 | No Hit |
| CCTACGGGGGGCAGCAGTGGGGAATATTGCACAATGGGCGAAAGCCTGAT | 105 | 1.3539651837524178 | No Hit |
| CCTACGGGTGGCAGCAGTGGGGAATATTGCACAATGGGCGCAAGCCTGAT | 103 | 1.3281753707285622 | No Hit |
| CCTACGGGTGGCAGCAGTGGGGAATATTGCACAATGGGCGAAAGCCTGAT | 102 | 1.3152804642166345 | No Hit |
| CCTACGGGCGGCAGCAGTGGGGAATATTGCACAATGGGCGAAAGCCTGAT | 100 | 1.2894906511927788 | No Hit |
| CCTACGGGCGGCTGCAGTAGGGAATCTTCCGCAATGGACGAAAGTCTGAC | 95 | 1.2250161186331399 | No Hit |
| CCTACGGGCGGCTGCAGTGGGGAATATTGCACAATGGGCGAAAGCCTGAT | 94 | 1.2121212121212122 | No Hit |
| CCTACGGGGGGCAGCAGTGGGGAATATTGCACAATGGGCGCAAGCCTGAT | 85 | 1.096067053513862 | No Hit |
| CCTACGGGCGGCAGCAGTGGGGAATATTGCACAATGGGCGCAAGCCTGAT | 82 | 1.0573823339780786 | No Hit |
| CCTACGGGAGGCTGCAGTGGGGAATATTGCACAATGGGCGCAAGCCTGAT | 82 | 1.0573823339780786 | No Hit |
| CCTACGGGGGGCTGCAGTGGGGAATATTGCACAATGGGCGAAAGCCTGAT | 78 | 1.0058027079303675 | No Hit |
| CCTACGGGTGGCTGCAGTGGGGAATATTGCACAATGGGCGCAAGCCTGAT | 74 | 0.9542230818826565 | No Hit |
| CCTACGGGAGGCAGCAGTGGGGAATATTGGACAATGGGCGAAAGCCTGAT | 69 | 0.8897485493230174 | No Hit |
| CCTACGGGAGGCTGCAGTGGGGAATATTGGACAATGGGCGAAAGCCTGAT | 68 | 0.8768536428110896 | No Hit |
| CCTACGGGGGGCTGCAGTGGGGAATATTGCACAATGGGCGCAAGCCTGAT | 68 | 0.8768536428110896 | No Hit |
| CCTACGGGAGGCAGCAGTAGGGAATCTTCCACAATGGACGAAAGTCTGAT | 65 | 0.8381689232753062 | No Hit |
| CCTACGGGGGGCAGCAGTGGGGAATATTGGACAATGGGCGAAAGCCTGAT | 63 | 0.8123791102514507 | No Hit |
| CCTACGGGCGGCTGCAGTGGGGAATATTGCACAATGGGCGCAAGCCTGAT | 61 | 0.7865892972275952 | No Hit |
| CCTACGGGTGGCTGCAGTGGGGAATATTGGACAATGGGCGAAAGCCTGAT | 55 | 0.7092198581560284 | No Hit |
| CCTACGGGCGGCAGCAGTGGGGAATATTGGACAATGGGCGAAAGCCTGAT | 55 | 0.7092198581560284 | No Hit |
| CCTACGGGAGGCAGCAGTGGGGAATATTGGACAATGGGGGGAACCCTGAT | 51 | 0.6576402321083172 | No Hit |
| CTCGGTCATTTAGAGGAAGTAAAAGTCGTAACAAGGTTTCCGTAGGTGAA | 47 | 0.6060606060606061 | No Hit |
| CTTAGTTATTTAGAGGAAGTAAAAGTCGTAACAAGGTTTCCGTAGGTGAA | 47 | 0.6060606060606061 | No Hit |
| CTACGTCATTTAGAGGAAGTAAAAGTCGTAACAAGGTTTCCGTAGGTGAA | 47 | 0.6060606060606061 | No Hit |
| CCTACGGGGGGCAGCAGTAGGGAATCTTCCGCAATGGACGCAAGTCTGAC | 46 | 0.5931656995486783 | No Hit |
| CCTACGGGGGGCTGCAGTGGGGAATATTGGACAATGGGCGAAAGCCTGAT | 46 | 0.5931656995486783 | No Hit |
| CCTACGGGAGGCAGCAGTAGGGAATCTTCCGCAATGGACGCAAGTCTGAC | 44 | 0.5673758865248227 | No Hit |
| CCTACGGGGGGCAGCAGTAGGGAATCTTCCACAATGGACGAAAGTCTGAT | 44 | 0.5673758865248227 | No Hit |
| CCTACGGGTGGCAGCAGTAGGGAATCTTCCACAATGGACGAAAGTCTGAT | 44 | 0.5673758865248227 | No Hit |
| CTTGGTCATTTAGAGGAAGTAAAAGTCGTAACAAGGTCTCCGTAGGTGAA | 43 | 0.5544809800128949 | No Hit |
| CCTACGGGTGGCAGCAGTAGGGAATCTTCCGCAATGGACGCAAGTCTGAC | 42 | 0.5415860735009671 | No Hit |
| CCTACGGGTGGCTGCAGTGGGGAATATTGGACAATGGGGGGAACCCTGAT | 42 | 0.5415860735009671 | No Hit |
| CCTACGGGAGGCTGCAGTGGGGAATATTGGACAATGGGGGGAACCCTGAT | 42 | 0.5415860735009671 | No Hit |
| CCTACGGGTGGCAGCAGTGGGGAATATTGGACAATGGGGGGAACCCTGAT | 39 | 0.5029013539651838 | No Hit |
| CCCGGTCATTTAGAGGAAGTAAAAGTCGTAACAAGGTTTCCGTAGGTGAA | 39 | 0.5029013539651838 | No Hit |
| CCTACGGGCGGCTGCAGTGGGGAATATTGGACAATGGGCGAAAGCCTGAT | 38 | 0.490006447453256 | No Hit |
| CCTACGGGCGGCAGCAGTAGGGAATCTTCCACAATGGACGAAAGTCTGAT | 38 | 0.490006447453256 | No Hit |
| CCTACGGGCGGCAGCAGTGGGGAATATTGGACAATGGGGGGAACCCTGAT | 38 | 0.490006447453256 | No Hit |
| CTAGGCTATTTAGAGGAAGTAAAAGTCGTAACAAGGTTTCCGTAGGTGAA | 36 | 0.46421663442940037 | No Hit |
| CCTACGGGGGGCAGCAGTGGGGAATATTGGACAATGGGGGGAACCCTGAT | 35 | 0.4513217279174726 | No Hit |
| CCTACGGGCGGCAGCAGTAGGGAATCTTCCGCAATGGACGCAAGTCTGAC | 34 | 0.4384268214055448 | No Hit |
| CCTACGGGTGGCAGCAGTGGGGAATATTGCACAATGGGCGGAAGCCTGAT | 34 | 0.4384268214055448 | No Hit |
| CCTACGGGGGGCAGCAGTGGGGAATATTGGACAATGGGGGCAACCCTGAT | 32 | 0.41263700838168926 | No Hit |
| CCTACGGGAGGCAGCAGTAGGGAATCTTCCGCAATGGGCGAAAGCCTGAC | 31 | 0.3997421018697615 | No Hit |
| CCTACGGGTGGCAGCAGTGGGGAATATTGGACAATGGGGGCAACCCTGAT | 31 | 0.3997421018697615 | No Hit |
| CCTACGGGTGGCAGCAGTAGGGAATCTTCCGCAATGGGCGAAAGCCTGAC | 30 | 0.3868471953578337 | No Hit |
| CCTACGGGAGGCAGCAGTGGGGAATATTGGACAATGGGGGCAACCCTGAT | 30 | 0.3868471953578337 | No Hit |
| CCTACGGGAGGCTGCAGTAGGGAATCTTCCGCAATGGACGCAAGTCTGAC | 30 | 0.3868471953578337 | No Hit |
| CCTACGGGGGGCAGCAGTGGGGAATCTTCCGCAATGGGCGAAAGCCTGAC | 30 | 0.3868471953578337 | No Hit |
| CCTACGGGTGGCTGCAGTAGGGAATCTTCCACAATGGACGAAAGTCTGAT | 29 | 0.37395228884590587 | No Hit |
| CCTACGGGAGGCAGCAGTGGGGAATATTGCACAATGGGCGGAAGCCTGAT | 27 | 0.3481624758220503 | No Hit |
| CCTACGGGAGGCTGCAGTGGGGAATATTGGACAATGGGGGCAACCCTGAT | 27 | 0.3481624758220503 | No Hit |
| CCTACGGGGGGCTGCAGTGGGGAATTTTGGACAATGGGCGCAAGCCTGAT | 26 | 0.3352675693101225 | No Hit |
| CCTACGGGGGGCTGCAGTGGGGAATATTGGACAATGGGGGGAACCCTGAT | 26 | 0.3352675693101225 | No Hit |
| CCTACGGGAGGCTGCAGTGGGGAATATTGCACAATGGGCGGAAGCCTGAT | 26 | 0.3352675693101225 | No Hit |
| CCTACGGGAGGCTGCAGTGGGGAATATTGGACAATGGGCGCAAGCCTGAT | 26 | 0.3352675693101225 | No Hit |
| CCTACGGGTGGCTGCAGTGGGGAATCTTCCGCAATGGGCGAAAGCCTGAC | 25 | 0.3223726627981947 | No Hit |
| CCTACGGGGGGCAGCAGTGGGGAATTTTGGACAATGGGCGCAAGCCTGAT | 25 | 0.3223726627981947 | No Hit |
| CCTACGGGTGGCTGCAGTGGGGAATATTGGACAATGGGGGCAACCCTGAT | 24 | 0.3094777562862669 | No Hit |
| CCTACGGGGGGCTGCAGTGGGGAATATTGCACAATGGGCGGAAGCCTGAT | 24 | 0.3094777562862669 | No Hit |
| CCTACGGGCGGCTGCAGTGGGGAATATTGGACAATGGGGGGAACCCTGAT | 24 | 0.3094777562862669 | No Hit |
| CCTACGGGAGGCAGCAGTGGGGAATTTTGGACAATGGGCGCAAGCCTGAT | 23 | 0.29658284977433913 | No Hit |
| CCTACGGGCGGCAGCAGTGGGGAATATTGGACAATGGGGGCAACCCTGAT | 22 | 0.28368794326241137 | No Hit |
| CCTACGGGGGGCTGCAGTAGGGAATCTTCCACAATGGACGAAAGTCTGAT | 22 | 0.28368794326241137 | No Hit |
| CCTACGGGTGGCAGCAGTGGGGAATCTTCCGCAATGGGCGAAAGCCTGAC | 21 | 0.27079303675048355 | No Hit |
| CCTACGGGGGGCTGCAGTGGGGAATCTTCCGCAATGGGCGAAAGCCTGAC | 21 | 0.27079303675048355 | No Hit |
| CCTACGGGTGGCTGCAGTGGGGAATATTGCGCAATGGGCGAAAGCCTGAC | 21 | 0.27079303675048355 | No Hit |
| CCTACGGGTGGCTGCAGTAGGGAATCTTCCGCAATGGACGCAAGTCTGAC | 20 | 0.2578981302385558 | No Hit |
| CCTACGGGTGGCTGCAGTGGGGAATATTGGACAATGGGCGCAAGCCTGAT | 20 | 0.2578981302385558 | No Hit |
| CCTACGGGAGGCAGCAGTGGGGAATCTTCCGCAATGGGCGAAAGCCTGAC | 20 | 0.2578981302385558 | No Hit |
| CCTACGGGGGGCTGCAGTAGGGAATCTTCCGCAATGGACGCAAGTCTGAC | 20 | 0.2578981302385558 | No Hit |
| CCTACGGGTGGCAGCAGTGGGGAATTTTGGACAATGGGCGCAAGCCTGAT | 20 | 0.2578981302385558 | No Hit |
| CCTACGGGGGGCAGCAGTGGGGAATATTGGACAATGGGCGCAAGCCTGAT | 20 | 0.2578981302385558 | No Hit |
| CCTACGGGGGGCTGCAGTGGGGAATATTGGACAATGGGGGCAACCCTGAT | 19 | 0.245003223726628 | No Hit |
| CCTACGGGCGGCTGCAGTGGGGAATATTGCACAATGGGCGGAAGCCTGAT | 18 | 0.23210831721470018 | No Hit |
| CCTACGGGGGGCAGCAGTGGGGAATATTGCACAATGGGCGGAAGCCTGAT | 18 | 0.23210831721470018 | No Hit |
| CCTACGGGAGGCAGCAGTGGGGAATATTGGACAATGGGCGCAAGCCTGAT | 18 | 0.23210831721470018 | No Hit |
| CCTACGGGCGGCAGCAGTGGGGAATATTGCACAATGGGCGGAAGCCTGAT | 18 | 0.23210831721470018 | No Hit |
| CCTACGGGTGGCTGCAGTGGGGAATTTTGGACAATGGGCGCAAGCCTGAT | 18 | 0.23210831721470018 | No Hit |
| CCTACGGGAGGCTGCAGTAGGGAATCTTCCACAATGGACGAAAGTCTGAT | 17 | 0.2192134107027724 | No Hit |
| CCTACGGGAGGCAGCAGTGGGGAATATTGCGCAATGGGCGAAAGCCTGAC | 17 | 0.2192134107027724 | No Hit |
| CCTACGGGGGGCTGCAGTAGGGAATCTTCCGCAATGGGCGAAAGCCTGAC | 17 | 0.2192134107027724 | No Hit |
| CCTACGGGTGGCTGCAGTAGGGAATCTTCCGCAATGGGCGAAAGCCTGAC | 16 | 0.20631850419084463 | No Hit |
| CCTACGGGTGGCAGCAGTGGGGAATCTTCCGCAATGGACGAAAGTCTGAC | 16 | 0.20631850419084463 | No Hit |
| CCTACGGGGGGCAGCAGTGGGGAATCTTCCGCAATGGACGAAAGTCTGAC | 16 | 0.20631850419084463 | No Hit |
| CCTACGGGTGGCTGCAGTGGGGAATATTGCACAATGGGCGGAAGCCTGAT | 16 | 0.20631850419084463 | No Hit |
| CTACGTCATTTAGAGGAAGTAAAAGTCGTAACAAGGTCTCCGTAGGTGAA | 16 | 0.20631850419084463 | No Hit |
| CCTACGGGGGGCAGCAGTAGGGAATCTTCCGCAATGGGCGAAAGCCTGAC | 15 | 0.19342359767891684 | No Hit |
| CCTACGGGCGGCAGCAGTGGGGAATTTTGGACAATGGGCGCAAGCCTGAT | 15 | 0.19342359767891684 | No Hit |
| CCTACGGGCGGCTGCAGTGGGGAATATTGGACAATGGGCGGAAGCCTGAT | 15 | 0.19342359767891684 | No Hit |
| CCTACGGGAGGCTGCAGTGGGGAATTTTGGACAATGGGCGCAAGCCTGAT | 15 | 0.19342359767891684 | No Hit |
| CCTACGGGCGGCTGCAGTGGGGAATATTGGACAATGGGCGCAAGCCTGAT | 15 | 0.19342359767891684 | No Hit |
| CCTACGGGCGGCAGCAGTGGGGAATTTTGGACAATGGGGGCAACCCTGAT | 14 | 0.18052869116698902 | No Hit |
| CCTACGGGGGGCTGCAGTGGGGAATATTGGACAATGGGCGCAAGCCTGAT | 14 | 0.18052869116698902 | No Hit |
| CCTACGGGCGGCTGCAGTAGGGAATCTTCCACAATGGACGAAAGTCTGAT | 14 | 0.18052869116698902 | No Hit |
| CCTACGGGAGGCTGCAGTAGGGAATCTTCCGCAATGGGCGAAAGCCTGAC | 14 | 0.18052869116698902 | No Hit |
| CCTACGGGCGGCAGCAGTAGGGAATCTTCCGCAATGGGCGAAAGCCTGAC | 13 | 0.16763378465506126 | No Hit |
| CCTACGGGCGGCTGCAGTAGGGAATCTTCCGCAATGGACGCAAGTCTGAC | 13 | 0.16763378465506126 | No Hit |
| CCTACGGGCGGCTGCAGTGGGGAATATTGGACAATGGGGGCAACCCTGAT | 13 | 0.16763378465506126 | No Hit |
| CCTACGGGAGGCTGCAGTGGGGAATCTTCCGCAATGGGCGAAAGCCTGAC | 13 | 0.16763378465506126 | No Hit |
| CCTACGGGTGGCTGCAGTGGGGAATCTTCCGCAATGGACGAAAGTCTGAC | 13 | 0.16763378465506126 | No Hit |
| CCTACGGGAGGCTGCAGTGGGGAATATTGCGCAATGGGCGAAAGCCTGAC | 13 | 0.16763378465506126 | No Hit |
| CCTACGGGTGGCTGCAGTGGGGAATTTTGGACAATGGGGGCAACCCTGAT | 12 | 0.15473887814313345 | No Hit |
| CCTACGGGGGGCAGCAGTGGGGAATTTTGGACAATGGGGGCAACCCTGAT | 12 | 0.15473887814313345 | No Hit |
| CCTACGGGCGGCTGCAGTGAGGAATATTGGTCAATGGGCGAGAGCCTGAA | 12 | 0.15473887814313345 | No Hit |
| CCTACGGGCGGCAGCAGTGGGGAATATTGGACAATGGGCGCAAGCCTGAT | 12 | 0.15473887814313345 | No Hit |
| CCTACGGGAGGCAGCAGTAGGGAATCTTCCCCAATGGACGAAAGTCTGAC | 11 | 0.14184397163120568 | No Hit |
| CTAGGCTATTTAGAGGAAGTAAAAGTCGTAACAAGGTCTCCGTAGGTGAA | 11 | 0.14184397163120568 | No Hit |
| CCTACGGGCGGCTGCAGTAGGGAATCTTCCGCAATGGGCGAAAGCCTGAC | 11 | 0.14184397163120568 | No Hit |
| CCTACGGGAGGCAGCAGTGGGGAATATTGGACAATGGGCGGAAGCCTGAT | 11 | 0.14184397163120568 | No Hit |
| CTTAGTTATTTAGAGGAAGTAAAAGTCGTAACAAGGTCTCCGTAGGTGAA | 11 | 0.14184397163120568 | No Hit |
| CTCGGTCATTTAGAGGAAGTAAAAGTCGTAACAAGGTCTCCGTAGGTGAA | 11 | 0.14184397163120568 | No Hit |
| CCTACGGGCGGCTGCAGTGGGGAATCTTCCGCAATGGGCGAAAGCCTGAC | 11 | 0.14184397163120568 | No Hit |
| CCTACGGGAGGCAGCAGTGGGGAATTTTGGACAATGGGGGCAACCCTGAT | 11 | 0.14184397163120568 | No Hit |
| CCTACGGGAGGCAGCAGTGGGGAATCTTCCGCAATGGACGAAAGTCTGAC | 10 | 0.1289490651192779 | No Hit |
| CCTACGGGTGGCTGCAGTGGGGAATATTGGACAATGGGCGGAAGCCTGAT | 10 | 0.1289490651192779 | No Hit |
| CTTGGTCATTTAGAGGAAGTAAAAGTCGTAACAAGGTCTCCGTTGGTGAA | 10 | 0.1289490651192779 | No Hit |
| CCTACGGGTGGCAGCAGTAGGGAATCTTCCCCAATGGACGAAAGTCTGAC | 10 | 0.1289490651192779 | No Hit |
| CCTACGGGTGGCTGCAGTGGGGAATCTTAGACAATGGGGGCAACCCTGAT | 10 | 0.1289490651192779 | No Hit |
| CCTACGGGAGGCTGCAGTGAGGAATATTGGTCAATGGGCGAGAGCCTGAA | 9 | 0.11605415860735009 | No Hit |
| CCTACGGGTGGCAGCAGTGGGGAATATTGGACAATGGGCGGAAGCCTGAT | 9 | 0.11605415860735009 | No Hit |
| CCTACGGGTGGCAGCAGTGGGGAATTTTGGACAATGGGGGCAACCCTGAT | 9 | 0.11605415860735009 | No Hit |
| CCTACGGGAGGCAGCAGTGAGGAATATTGGTCAATGGGCGAGAGCCTGAA | 9 | 0.11605415860735009 | No Hit |
| CCTACGGGTGGCAGCAGTGGGGAATATTGGACAATGGGCGCAAGCCTGAT | 9 | 0.11605415860735009 | No Hit |
| CCTACGGGCGGCAGCAGTGAGGAATATTGGTCAATGGGCGAGAGCCTGAA | 9 | 0.11605415860735009 | No Hit |
| CCTACGGGGGGCAGCAGTGGGGAATATTGCGCAATGGGCGAAAGCCTGAC | 9 | 0.11605415860735009 | No Hit |
| CCTACGGGAGGCTGCAGTGGGGAATCTTGCGCAATGGGCGAAAGCCTGAC | 9 | 0.11605415860735009 | No Hit |
| CCTACGGGAGGCTGCAGTGGGGAATCTTCCGCAATGGACGAAAGTCTGAC | 8 | 0.10315925209542232 | No Hit |
| CCTACGGGCGGCTGCAGTGGGGAATTTTGGACAATGGGCGCAAGCCTGAT | 8 | 0.10315925209542232 | No Hit |
| CCTACGGGCGGCAGCAGTGGGGAATCTTCCGCAATGGGCGAAAGCCTGAC | 8 | 0.10315925209542232 | No Hit |
| CCTACGGGGGGCTGCAGTGGGGAATTTTGGACAATGGGGGCAACCCTGAT | 8 | 0.10315925209542232 | No Hit |
| CCTACGGGCGGCTGCAGTGGGGAATATTGCGCAATGGGCGAAAGCCTGAC | 8 | 0.10315925209542232 | No Hit |
| CCTACGGGTGGCTGCAGTGGGGAATCTTGCGCAATGGGCGAAAGCCTGAC | 8 | 0.10315925209542232 | No Hit |
| CCTACGGGCGGCTGCAGTGGGGAATATTGCACAATGGGGGAAACCCTGAT | 8 | 0.10315925209542232 | No Hit |

## Adapter Content

## Kmer Content

| Sequence | Count | PValue | Obs/Exp Max | Max Obs/Exp Position |
| --- | --- | --- | --- | --- |
| ATCTGAT | 30 | 1.8189894E-12 | 316.01392 | 295 |
| CGTTTGA | 10 | 6.747165E-4 | 316.0139 | 295 |
| AGTACAG | 10 | 6.747165E-4 | 316.0139 | 295 |
| AGTGTGG | 10 | 6.747165E-4 | 316.0139 | 295 |
| GGAAGAG | 10 | 6.747165E-4 | 316.0139 | 295 |
| GGTAGAG | 10 | 6.747165E-4 | 316.0139 | 295 |
| GAGAGAG | 10 | 6.747165E-4 | 316.0139 | 295 |
| GGCAGGA | 10 | 6.747165E-4 | 316.0139 | 295 |
| TGGCAGC | 120 | 0.0 | 293.58713 | 9 |
| AGGCTGC | 60 | 0.0 | 293.58713 | 9 |
| GAGGCTG | 60 | 0.0 | 293.58713 | 8 |
| GAGGCAG | 105 | 0.0 | 293.5871 | 8 |
| TGGTCAT | 20 | 5.9400918E-8 | 293.5871 | 3 |
| CCTACGG | 710 | 0.0 | 293.5871 | 1 |
| TACGGGT | 210 | 0.0 | 293.5871 | 3 |
| TCGGTCA | 10 | 8.424049E-4 | 293.5871 | 2 |
| GCGGCAG | 95 | 0.0 | 293.5871 | 8 |
| ACGGGCG | 165 | 0.0 | 293.5871 | 4 |
| TTTAGAG | 40 | 0.0 | 293.5871 | 9 |
| CTCGGTC | 10 | 8.424049E-4 | 293.5871 | 1 |

Produced by FastQC (version 0.11.7)
