# Supplementary material for: Assessment of airborne bacteria from a public health institution in Mexico City
Source: PLOS Glob Public Health. 2024 Nov 7;4(11):e0003672. doi: 10.1371/journal.pgph.0003672 (PMC11542838; doi:10.1371/journal.pgph.0003672)
Supplement: S1 Text — (ZIP) [file pgph.0003672.s001.zip › Hospital_16S_QC/21022023_CED3_16S_S42_L001_R2_001_fastqc.html]

21022023\_CED3\_16S\_S42\_L001\_R2\_001.fastq.gz FastQC Report 

FastQC Report

Tue 14 Mar 2023  
21022023\_CED3\_16S\_S42\_L001\_R2\_001.fastq.gz

## Summary

- Basic Statistics
- Per base sequence quality
- Per tile sequence quality
- Per sequence quality scores
- Per base sequence content
- Per sequence GC content
- Per base N content
- Sequence Length Distribution
- Sequence Duplication Levels
- Overrepresented sequences
- Adapter Content
- Kmer Content

## Basic Statistics

| Measure | Value |
| --- | --- |
| Filename | 21022023\_CED3\_16S\_S42\_L001\_R2\_001.fastq.gz |
| File type | Conventional base calls |
| Encoding | Sanger / Illumina 1.9 |
| Total Sequences | 7755 |
| Sequences flagged as poor quality | 0 |
| Sequence length | 186-301 |
| %GC | 54 |

## Per base sequence quality

## Per tile sequence quality

## Per sequence quality scores

## Per base sequence content

## Per sequence GC content

## Per base N content

## Sequence Length Distribution

## Sequence Duplication Levels

## Overrepresented sequences

| Sequence | Count | Percentage | Possible Source |
| --- | --- | --- | --- |
| GACTACTGGGGTATCTAATCCTGTTTGCTCCCCACGCTTTCGCGCCTCAG | 239 | 3.0818826563507415 | No Hit |
| GACTACTGGGGTATCTAATCCTGTTCGCTCCCCATGCTTTCGCTCCTCAG | 213 | 2.746615087040619 | No Hit |
| GACTACAGGGGTATCTAATCCTGTTTGCTCCCCACGCTTTCGCGCCTCAG | 199 | 2.5660863958736297 | No Hit |
| GACTACTAGGGTATCTAATCCTGTTTGCTCCCCACGCTTTCGCGCCTCAG | 193 | 2.488716956802063 | No Hit |
| GACTACTAGGGTATCTAATCCTGTTCGCTCCCCATGCTTTCGCTCCTCAG | 184 | 2.372662798194713 | No Hit |
| GACTACTCGGGTATCTAATCCTGTTTGCTCCCCACGCTTTCGCGCCTCAG | 178 | 2.2952933591231464 | No Hit |
| GACTACCGGGGTATCTAATCCTGTTTGCTCCCCACGCTTTCGCGCCTCAG | 174 | 2.243713733075435 | No Hit |
| GACTACAGGGGTATCTAATCCTGTTCGCTCCCCATGCTTTCGCTCCTCAG | 170 | 2.192134107027724 | No Hit |
| GACTACACGGGTATCTAATCCTGTTTGCTCCCCACGCTTTCGCGCCTCAG | 170 | 2.192134107027724 | No Hit |
| GACTACACGGGTATCTAATCCTGTTCGCTCCCCATGCTTTCGCTCCTCAG | 164 | 2.114764667956157 | No Hit |
| GACTACAAGGGTATCTAATCCTGTTCGCTCCCCATGCTTTCGCTCCTCAG | 163 | 2.1018697614442297 | No Hit |
| GACTACCAGGGTATCTAATCCTGTTTGCTCCCCACGCTTTCGCGCCTCAG | 161 | 2.076079948420374 | No Hit |
| GACTACCGGGGTATCTAATCCTGTTCGCTCCCCATGCTTTCGCTCCTCAG | 156 | 2.011605415860735 | No Hit |
| GACTACCCGGGTATCTAATCCTGTTCGCTCCCCATGCTTTCGCTCCTCAG | 155 | 1.9987105093488073 | No Hit |
| GACTACTCGGGTATCTAATCCTGTTCGCTCCCCATGCTTTCGCTCCTCAG | 151 | 1.9471308833010963 | No Hit |
| GACTACCAGGGTATCTAATCCTGTTCGCTCCCCATGCTTTCGCTCCTCAG | 144 | 1.8568665377176015 | No Hit |
| GACTACCCGGGTATCTAATCCTGTTTGCTCCCCACGCTTTCGCGCCTCAG | 141 | 1.8181818181818181 | No Hit |
| GACTACAAGGGTATCTAATCCTGTTTGCTCCCCACGCTTTCGCGCCTCAG | 139 | 1.7923920051579625 | No Hit |
| GCTGCGTTCTTCATCGATGCCGGAACCAAGAGATCCATTGTTGAAAGTTT | 84 | 1.0831721470019342 | No Hit |
| GACTACCGGGGTATCTAATCCTGTTCGCTCCCCACGCTTTCGCTCCTCAG | 79 | 1.0186976144422952 | No Hit |
| GACTACTGGGGTATCTAATCCTGTTCGCTCCCCACGCTTTCGCTCCTCAG | 78 | 1.0058027079303675 | No Hit |
| GACTACTAGGGTATCTAATCCTGTTCGCTCCCCACGCTTTCGCTCCTCAG | 77 | 0.9929078014184398 | No Hit |
| GACTACTCGGGTATCTAATCCTGTTTGCTCCCCACGCTTTCGCACCTCAG | 70 | 0.9026434558349452 | No Hit |
| GACTACAAGGGTATCTAATCCTGTTTGCTCCCCACGCTTTCGCACCTCAG | 64 | 0.8252740167633785 | No Hit |
| GACTACTAGGGTATCTAATCCTGTTTGCTCCCCACGCTTTCGCACCTCAG | 63 | 0.8123791102514507 | No Hit |
| GACTACACGGGTATCTAATCCTGTTTGCTCCCCACGCTTTCGCACCTCAG | 63 | 0.8123791102514507 | No Hit |
| GACTACCGGGGTATCTAATCCTGTTTGCTCCCCACGCTTTCGCACCTCAG | 62 | 0.799484203739523 | No Hit |
| GACTACACGGGTATCTAATCCTGTTCGCTCCCCACGCTTTCGCTCCTCAG | 60 | 0.7736943907156674 | No Hit |
| GACTACAGGGGTATCTAATCCTGTTTGCTCCCCACGCTTTCGCACCTCAG | 60 | 0.7736943907156674 | No Hit |
| GACTACTGGGGTATCTAATCCTGTTTGCTCCCCACGCTTTCGCACCTCAG | 59 | 0.7607994842037396 | No Hit |
| GACTACAAGGGTATCTAATCCTGTTCGCTCCCCACGCTTTCGCTCCTCAG | 58 | 0.7479045776918117 | No Hit |
| GACTACCCGGGTATCTAATCCTGTTTGCTCCCCACGCTTTCGCACCTCAG | 58 | 0.7479045776918117 | No Hit |
| GACTACAGGGGTATCTAATCCTGTTCGCTCCCCACGCTTTCGCTCCTCAG | 55 | 0.7092198581560284 | No Hit |
| GACTACCAGGGTATCTAATCCTGTTTGCTCCCCACGCTTTCGCACCTCAG | 55 | 0.7092198581560284 | No Hit |
| GACTACCAGGGTATCTAATCCTGTTCGCTCCCCACGCTTTCGCTCCTCAG | 53 | 0.6834300451321728 | No Hit |
| GACTACAGGGGTATCTAATCCTGTTTGCTCCCCATGCTTTCGCACCTCAG | 52 | 0.670535138620245 | No Hit |
| GACTACTAGGGTATCTAATCCTGTTTGCTCCCCATGCTTTCGCACCTCAG | 47 | 0.6060606060606061 | No Hit |
| GACTACCCGGGTATCTAATCCTGTTCGCTCCCCACGCTTTCGCTCCTCAG | 47 | 0.6060606060606061 | No Hit |
| GACTACTCGGGTATCTAATCCTGTTCGCTCCCCACGCTTTCGCTCCTCAG | 46 | 0.5931656995486783 | No Hit |
| GACTACCGGGGTATCTAATCCTGTTTGCTCCCCATGCTTTCGCACCTCAG | 43 | 0.5544809800128949 | No Hit |
| GACTACAAGGGTATCTAATCCTGTTTGCTCCCCATGCTTTCGCACCTCAG | 42 | 0.5415860735009671 | No Hit |
| GACTACTGGGGTATCTAATCCTGTTTGCTCCCCATGCTTTCGCACCTCAG | 41 | 0.5286911669890393 | No Hit |
| GACTACAGGGGTATCTAATCCTGTTTGCTACCCACGCTTTCGAACCTCAG | 41 | 0.5286911669890393 | No Hit |
| GACTACTGGGGTATCTAATCCTGTTTGATCCCCACGCTTTCGCACATCAG | 40 | 0.5157962604771116 | No Hit |
| GACTACTGGGGTATCTAATCCTGTTTGCTACCCACGCTTTCGAACCTCAG | 40 | 0.5157962604771116 | No Hit |
| GCTGCGTTCTTCATCGATGCCGGAACCAAGAGATCCGTTGTTGAAAGTTT | 39 | 0.5029013539651838 | No Hit |
| GACTACTCGGGTATCTAATCCTGTTTGCTACCCACGCTTTCGAACCTCAG | 36 | 0.46421663442940037 | No Hit |
| GACTACCAGGGTATCTAATCCTGTTTGCTCCCCATGCTTTCGCACCTCAG | 36 | 0.46421663442940037 | No Hit |
| GCTGCGTTCTTCATCGATGCCAGAACCAAGAGATCCGTTGTTAAAAGTTT | 36 | 0.46421663442940037 | No Hit |
| GACTACACGGGTATCTAATCCTGTTTGCTCCCCATGCTTTCGCACCTCAG | 35 | 0.4513217279174726 | No Hit |
| GACTACTCGGGTATCTAATCCTGTTTGCTCCCCATGCTTTCGCACCTCAG | 35 | 0.4513217279174726 | No Hit |
| GACTACCGGGGTATCTAATCCTGTTTGCTACCCACGCTTTCGAACCTCAG | 34 | 0.4384268214055448 | No Hit |
| GACTACACGGGTATCTAATCCTGTTTGCTACCCACGCTTTCGAACCTCAG | 32 | 0.41263700838168926 | No Hit |
| GACTACACGGGTATCTAATCCTGTTTGCTCCCCACGCTTTCGTGCATGAG | 30 | 0.3868471953578337 | No Hit |
| GACTACAGGGGTATCTAATCCTGTTTGATCCCCACGCTTTCGCACATCAG | 28 | 0.36105738233397805 | No Hit |
| GACTACAAGGGTATCTAATCCTGTTTGCTACCCACGCTTTCGAACCTCAG | 28 | 0.36105738233397805 | No Hit |
| GACTACCCGGGTATCTAATCCTGTTTGCTCCCCACGCTTTCGTGCATGAG | 27 | 0.3481624758220503 | No Hit |
| GACTACCAGGGTATCTAATCCTGTTTGCTACCCACGCTTTCGAACCTCAG | 27 | 0.3481624758220503 | No Hit |
| GACTACCAGGGTATCTAATCCTGTTTGATCCCCACGCTTTCGCACATCAG | 27 | 0.3481624758220503 | No Hit |
| GCTGCGTTCTTCATCGTTGCCGGAACCAAGAGATCCATTGTTGAAAGTTT | 26 | 0.3352675693101225 | No Hit |
| GACTACCCGGGTATCTAATCCTGTTTGCTCCCCATGCTTTCGCACCTCAG | 26 | 0.3352675693101225 | No Hit |
| GACTACCGGGGTATCTAATCCTGTTTGATCCCCACGCTTTCGCACATCAG | 26 | 0.3352675693101225 | No Hit |
| GACTACTCGGGTATCTAATCCTGTTTGATCCCCACGCTTTCGCACATCAG | 25 | 0.3223726627981947 | No Hit |
| GACTACTAGGGTATCTAATCCTGTTTGCTACCCACGCTTTCGAACCTCAG | 25 | 0.3223726627981947 | No Hit |
| GACTACTAGGGTATCTAATCCTGTTTGATCCCCACGCTTTCGCACATCAG | 25 | 0.3223726627981947 | No Hit |
| GACTACCAGGGTATCTAATCCTGTTTGCTCCCCACGCTTTCGTGCATGAG | 24 | 0.3094777562862669 | No Hit |
| GACTACCGGGGTATCTAATCCTGTTTGCTCCCCACGCTTTCGTGCATGAG | 23 | 0.29658284977433913 | No Hit |
| GACTACCCGGGTATCTAATCCTGTTTGATCCCCACGCTTTCGCACATCAG | 22 | 0.28368794326241137 | No Hit |
| GACTACTGGGGTATCTAATCCTGTTTGCTCCCCACGCTTTCGTGCATGAG | 22 | 0.28368794326241137 | No Hit |
| ACTGTGTTCTTCATCGATGCCGGAACCAAGAGATCCATTGTTGAAAGTTT | 21 | 0.27079303675048355 | No Hit |
| GACTACTAGGGTATCTAATCCTGTTTGCTCCCCACGCTTTCGTGCATGAG | 21 | 0.27079303675048355 | No Hit |
| GACTACAGGGGTATCTAATCCTGTTTGCTCCCCACGCTTTCGTGCATGAG | 21 | 0.27079303675048355 | No Hit |
| GCGTTCTTCATCGATGCCGGAACCAAGAGATCCATTGTTGAAAGTTTTAA | 20 | 0.2578981302385558 | No Hit |
| GCTGCGTTCTTCATCGTTGCCGGAACCAAGAGATCCGTTGTTGAAAGTTT | 18 | 0.23210831721470018 | No Hit |
| GACTACAGGGGTATCTAATCCTGTTTGCTCCCCACGCTTTCGCACCTGAG | 18 | 0.23210831721470018 | No Hit |
| GACTACTGGGGTATCTAATCCTGTTTGCTCCCCATGCTTTCGTACCTCAG | 18 | 0.23210831721470018 | No Hit |
| GACTACAAGGGTATCTAATCCTGTTTGATCCCCACGCTTTCGCACATCAG | 18 | 0.23210831721470018 | No Hit |
| GCTACGTTCTTCATCGATGCCGGAACCAAGAGATCCATTGTTGAAAGTTT | 17 | 0.2192134107027724 | No Hit |
| GACTACCCGGGTATCTAATCCTGTTTGCTACCCACGCTTTCGAACCTCAG | 17 | 0.2192134107027724 | No Hit |
| GACTACTGGGGTATCTAATCCTGTTTGCTCCCCACGCTTTCGCACCTGAG | 16 | 0.20631850419084463 | No Hit |
| GACTACAAGGGTATCTAATCCTGTTTGCTCCCCACGCTTTCGTGCATGAG | 16 | 0.20631850419084463 | No Hit |
| GCGTTCTTCATCGATGCCAGAACCAAGAGATCCGTTGTTAAAAGTTTTAA | 15 | 0.19342359767891684 | No Hit |
| GACTACAGGGGTATCTAATCCTGTTTGCTCCCCATGCTTTCGTACCTCAG | 15 | 0.19342359767891684 | No Hit |
| GACTACTCGGGTATCTAATCCTGTTTGCTCCCCACGCTTTCGTGCATGAG | 15 | 0.19342359767891684 | No Hit |
| GACTACACGGGTATCTAATCCTGTTTGATCCCCACGCTTTCGCACATCAG | 15 | 0.19342359767891684 | No Hit |
| GCTGCGTTCTTCATCGATGCGAGAGCCAAGAGATCCGTTGCTGAAAGTTG | 15 | 0.19342359767891684 | No Hit |
| GACTACTCGGGTATCTAATCCTGTTTGCTCCCCACGCTTTCGCACCTGAG | 14 | 0.18052869116698902 | No Hit |
| GACTACACGGGTATCTAATCCTGTTCGCTCCCCATGCTTTCGCTTCTCAG | 13 | 0.16763378465506126 | No Hit |
| GACTACAAGGGTATCTAATCCTGTTTGCTCCCCACGCTTTCGCACCTGAG | 13 | 0.16763378465506126 | No Hit |
| GACTACCAGGGTATCTAATCCTGTTTGATCCCCACACTTTCGCACCTCAG | 13 | 0.16763378465506126 | No Hit |
| GACTACTAGGGTATCTAATCCTGTTTGCTCCCCACGCTTTCGCACCTGAG | 13 | 0.16763378465506126 | No Hit |
| GACTACACGGGTATCTAATCCTGTTTGCTCCCCATGCTTTCGCTCCTCAG | 13 | 0.16763378465506126 | No Hit |
| GACTACACGGGTATCTAATCCTGTTTGCTCCCCACGCTTTCGCACCTGAG | 12 | 0.15473887814313345 | No Hit |
| GCTGCGTTCTTCATCGATGCCAGAACCAAGAGATCCGTTGTTGAAAGTTT | 12 | 0.15473887814313345 | No Hit |
| GACTACTGGGGTATCTAATCCTGTTCGCTCCCCATGCTTTCGCTTCTCAG | 12 | 0.15473887814313345 | No Hit |
| GACTACCGGGGTATCTAATCCTGTTCGCTCCCCACGCTTTCGCGCCTCAG | 12 | 0.15473887814313345 | No Hit |
| GCTACGTTCTTCATCGATGCCAGAACCAAGAGATCCGTTGTTAAAAGTTT | 12 | 0.15473887814313345 | No Hit |
| GACTACTCGGGTATCTAATCCTGTTTGCTCCCCATGCTTTCGTACCTCAG | 12 | 0.15473887814313345 | No Hit |
| GCTGCGTTCTTCATCGTTGCCAGAACCAAGAGATCCGTTGTTAAAAGTTT | 12 | 0.15473887814313345 | No Hit |
| GACTACTAGGGTATCTAATCCTGTTTGCTCCCCACGCTTTCGCTCCTCAG | 11 | 0.14184397163120568 | No Hit |
| GACTACTGGGGTATCTAATCCTGTTTGATCCCCACGCTTTCGCGCCTCAG | 11 | 0.14184397163120568 | No Hit |
| GCTGCGTTCTTCATCGATGCGAGAACCAAGAGATCCATTGTTAAAAGTTG | 11 | 0.14184397163120568 | No Hit |
| GACTACCGGGGTATCTAATCCTGTTTGCTCCCCACGCTTTCGTGCCTCAG | 11 | 0.14184397163120568 | No Hit |
| GACTACCAGGGTATCTAATCCTGTTTGCTCCCCACGCTTTCGCACCTGAG | 10 | 0.1289490651192779 | No Hit |
| GACTACTGGGGTATCTAATCCTGTTCGCTCCCCACGCTTTCGTGCCTCAG | 10 | 0.1289490651192779 | No Hit |
| GACTACTGGGGTATCTAATCCTGTTTGCTCCCCACGCTTTCGTGCCTCAG | 10 | 0.1289490651192779 | No Hit |
| GACTACCGGGGTATCTAATCCTGTTTGATCCCCACGCTTTCGCGCCTCAG | 10 | 0.1289490651192779 | No Hit |
| GACTACTGGGGTATCTAATCCTGTTTGCTCCCCATGCTTTCGCTCCTCAG | 10 | 0.1289490651192779 | No Hit |
| GACTACCCGGGTATCTAATCCTGTTTGCTCCCCACGCTTTCGCACCTGAG | 10 | 0.1289490651192779 | No Hit |
| GACTACCGGGGTATCTAATCCTGTTTGCTCCCCACGCTTTCGCTCCTCAG | 10 | 0.1289490651192779 | No Hit |
| GACTACCGGGGTATCTAATCCTGTTCGCTACCCATGCTTTCGCTCCTCAG | 10 | 0.1289490651192779 | No Hit |
| GACTACTCGGGTATCTAATCCTGTTCGCTCCCCACGCTTTCGTGCCTCAG | 9 | 0.11605415860735009 | No Hit |
| GCTGCGTTCTTCATCGATGCCAGAACCAAGAGATCCGTTGTTGAAAGTTG | 9 | 0.11605415860735009 | No Hit |
| GACTACAGGGGTATCTAATCCTGTTTGCTCCCCACGCTTTCGCTCCTCAG | 9 | 0.11605415860735009 | No Hit |
| GACTACTGGGGTATCTAATCCTGTTTGCTCCCCACGCTTTCGCTCCTCAG | 9 | 0.11605415860735009 | No Hit |
| GACTACCAGGGTATCTAATCCTGTTCGCTCCCCACACTTTCGCTCCTCAG | 9 | 0.11605415860735009 | No Hit |
| GACTACAGGGGTATCTAATCCTGTTCGCTCCCCACGCTTTCGTGCCTCAG | 9 | 0.11605415860735009 | No Hit |
| GACTACTAGGGTATCTAATCCTGTTTGATCCCCACGCTTTCGTGCCTGAG | 9 | 0.11605415860735009 | No Hit |
| GACTACTAGGGTATCTAATCCTGTTTGCTCCCCACGCTTTCGTGCCTCAG | 9 | 0.11605415860735009 | No Hit |
| GACTACCGGGGTATCTAATCCTGTTTGCTCCCCACGCTTTCGCACCTGAG | 9 | 0.11605415860735009 | No Hit |
| GACTACCCGGGTATCTAATCCTGTTTGCTCCCCATGCTTTCGTACCTCAG | 9 | 0.11605415860735009 | No Hit |
| ACTGTGTTCTTCATCGATGCCAGAACCAAGAGATCCGTTGTTAAAAGTTT | 9 | 0.11605415860735009 | No Hit |
| GCTACGTTCTTCATCGATGCCGGAACCAAGAGATCCGTTGTTGAAAGTTT | 9 | 0.11605415860735009 | No Hit |
| GACTACAAGGGTATCTAATCCTGTTTGATCCCCACGCTTTCGCGCCTCAG | 9 | 0.11605415860735009 | No Hit |
| GACTACTCGGGTATCTAATCCTGTTTGATCCCCACACTTTCGCACCTCAG | 9 | 0.11605415860735009 | No Hit |
| GACTACCCGGGTATCTAATCCTGTTTGATCCCCACGCTTTCGCGCCTCAG | 9 | 0.11605415860735009 | No Hit |
| GACTACTAGGGTATCTAATCCTGTTTGATCCCCACGCTTTCGCGCCTCAG | 8 | 0.10315925209542232 | No Hit |
| GACTACCCGGGTATCTAAGCCTGTTTGCTCCCCACCCTTTCGCTCCTCAG | 8 | 0.10315925209542232 | No Hit |
| GACTACTAGGGTATCTAATCCTGTTCGCTCCCCACGCTTTCGCGCCTCAG | 8 | 0.10315925209542232 | No Hit |
| GACTACCGGGGTATCTAATCCTGTTTGCTCCCCATGCTTTCGTACCTCAG | 8 | 0.10315925209542232 | No Hit |
| GACTACACGGGTATCTAATCCTGTTTGCTCCCCATGCTTTCGTACCTCAG | 8 | 0.10315925209542232 | No Hit |
| GACTACAAGGGTATCTAATCCTGTTTGCTCCCCACGCTTTCGAGCCTCAG | 8 | 0.10315925209542232 | No Hit |
| GACTACTCGGGTATCTAATCCTGTTTGCTCCCCACGCTTTCGCTCCTCAG | 8 | 0.10315925209542232 | No Hit |
| GACTACTAGGGTATCTAATCCTGTTCGCTACCCATGCTTTCGCTCCTCAG | 8 | 0.10315925209542232 | No Hit |
| GACTACTGGGGTATCTAATCCTGTTTGCTCCCCACGCTTTCGAGCCTCAG | 8 | 0.10315925209542232 | No Hit |
| GACTACACGGGTATCTAATCCTGTTTGATCCCCACGCTTTCGCGCCTCAG | 8 | 0.10315925209542232 | No Hit |
| GACTACAAGGGTATCTAATCCTGTTTGCTCCCCATGCTTTCGCTCCTCAG | 8 | 0.10315925209542232 | No Hit |

## Adapter Content

## Kmer Content

| Sequence | Count | PValue | Obs/Exp Max | Max Obs/Exp Position |
| --- | --- | --- | --- | --- |
| GTTTGCG | 5 | 2.2179725E-4 | 5684.625 | 295 |
| TTAAGCG | 5 | 2.2179725E-4 | 5684.625 | 295 |
| TTTAGCA | 5 | 2.2179725E-4 | 5684.625 | 295 |
| ATAGACG | 5 | 2.2179725E-4 | 5684.625 | 295 |
| GTTAGCG | 5 | 2.2179725E-4 | 5684.625 | 295 |
| TTAGCCG | 5 | 2.2179725E-4 | 5684.625 | 295 |
| TTAGCAG | 5 | 2.2179725E-4 | 5684.625 | 295 |
| GTTAGCA | 10 | 8.8711095E-4 | 2842.3125 | 295 |
| AAGGGTA | 110 | 0.0 | 293.40002 | 7 |
| TACACGG | 50 | 0.0 | 293.40002 | 4 |
| TGGGGTA | 110 | 0.0 | 293.40002 | 7 |
| ACGGGTA | 50 | 0.0 | 293.40002 | 7 |
| TACTGGG | 110 | 0.0 | 293.40002 | 4 |
| ACAAGGG | 110 | 0.0 | 293.40002 | 5 |
| CAAGGGT | 110 | 0.0 | 293.40002 | 6 |
| CTACTGG | 110 | 0.0 | 293.40002 | 3 |
| ACTACTG | 110 | 0.0 | 293.40002 | 2 |
| GACTACC | 220 | 0.0 | 293.40002 | 1 |
| AGGGGTA | 75 | 0.0 | 293.4 | 7 |
| CTACAGG | 75 | 0.0 | 293.4 | 3 |

Produced by FastQC (version 0.11.7)
