# Supplementary material for: Assessment of airborne bacteria from a public health institution in Mexico City
Source: PLOS Glob Public Health. 2024 Nov 7;4(11):e0003672. doi: 10.1371/journal.pgph.0003672 (PMC11542838; doi:10.1371/journal.pgph.0003672)
Supplement: S1 Text — (ZIP) [file pgph.0003672.s001.zip › Hospital_16S_QC/21022023_CP1D2_16S_S35_L001_R1_001_fastqc.html]

21022023\_CP1D2\_16S\_S35\_L001\_R1\_001.fastq.gz FastQC Report 

FastQC Report

Wed 15 Mar 2023  
21022023\_CP1D2\_16S\_S35\_L001\_R1\_001.fastq.gz

## Summary

- Basic Statistics
- Per base sequence quality
- Per tile sequence quality
- Per sequence quality scores
- Per base sequence content
- Per sequence GC content
- Per base N content
- Sequence Length Distribution
- Sequence Duplication Levels
- Overrepresented sequences
- Adapter Content
- Kmer Content

## Basic Statistics

| Measure | Value |
| --- | --- |
| Filename | 21022023\_CP1D2\_16S\_S35\_L001\_R1\_001.fastq.gz |
| File type | Conventional base calls |
| Encoding | Sanger / Illumina 1.9 |
| Total Sequences | 473286 |
| Sequences flagged as poor quality | 0 |
| Sequence length | 40-301 |
| %GC | 55 |

## Per base sequence quality

## Per tile sequence quality

## Per sequence quality scores

## Per base sequence content

## Per sequence GC content

## Per base N content

## Sequence Length Distribution

## Sequence Duplication Levels

## Overrepresented sequences

| Sequence | Count | Percentage | Possible Source |
| --- | --- | --- | --- |
| CCTACGGGAGGCTGCAGTGGGGAATATTGGACAATGGGCGAAAGCCTGAT | 8550 | 1.8065186800370177 | No Hit |
| CCTACGGGTGGCTGCAGTGGGGAATATTGGACAATGGGCGAAAGCCTGAT | 8501 | 1.7961655320461622 | No Hit |
| CCTACGGGGGGCTGCAGTGGGGAATATTGGACAATGGGCGAAAGCCTGAT | 8077 | 1.7065791086150868 | No Hit |
| CCTACGGGTGGCTGCAGTGGGGAATATTGCACAATGGGCGCAAGCCTGAT | 7946 | 1.6789002843946366 | No Hit |
| CCTACGGGAGGCTGCAGTGGGGAATATTGCACAATGGGCGCAAGCCTGAT | 7945 | 1.6786889956601294 | No Hit |
| CCTACGGGGGGCTGCAGTGGGGAATATTGCACAATGGGCGCAAGCCTGAT | 7430 | 1.5698752973888939 | No Hit |
| CCTACGGGGGGCAGCAGTGGGGAATATTGGACAATGGGCGAAAGCCTGAT | 7017 | 1.4826130500373982 | No Hit |
| CCTACGGGGGGCAGCAGTGGGGAATATTGCACAATGGGCGCAAGCCTGAT | 6547 | 1.383307344818989 | No Hit |
| CCTACGGGTGGCTGCAGTGGGGAATATTGCACAATGGGCGAAAGCCTGAT | 6437 | 1.360065584023191 | No Hit |
| CCTACGGGAGGCAGCAGTGGGGAATATTGGACAATGGGCGAAAGCCTGAT | 6393 | 1.3507688797048718 | No Hit |
| CCTACGGGTGGCTGCAGTGGGGAATATTGGACAATGGGCGCAAGCCTGAT | 6356 | 1.3429511965281036 | No Hit |
| CCTACGGGAGGCAGCAGTGGGGAATATTGCACAATGGGCGCAAGCCTGAT | 6195 | 1.3089337102724357 | No Hit |
| CCTACGGGAGGCTGCAGTGGGGAATATTGCACAATGGGCGAAAGCCTGAT | 6090 | 1.286748393149174 | No Hit |
| CCTACGGGAGGCTGCAGTGGGGAATATTGGACAATGGGCGCAAGCCTGAT | 6050 | 1.278296843768884 | No Hit |
| CCTACGGGCGGCTGCAGTGGGGAATATTGGACAATGGGCGAAAGCCTGAT | 6017 | 1.2713243155301446 | No Hit |
| CCTACGGGGGGCTGCAGTGGGGAATATTGGACAATGGGCGCAAGCCTGAT | 5982 | 1.2639292098223907 | No Hit |
| CCTACGGGGGGCTGCAGTGGGGAATATTGCACAATGGGCGAAAGCCTGAT | 5709 | 1.2062473853019104 | No Hit |
| CCTACGGGGGGCAGCAGTAGGGAATCTTCCGCAATGGGCGAAAGCCTGAC | 5679 | 1.199908723266693 | No Hit |
| CCTACGGGTGGCAGCAGTGGGGAATATTGGACAATGGGCGAAAGCCTGAT | 5615 | 1.1863862442582287 | No Hit |
| CCTACGGGCGGCTGCAGTGGGGAATATTGCACAATGGGCGCAAGCCTGAT | 5542 | 1.1709621666391992 | No Hit |
| CCTACGGGTGGCAGCAGTGGGGAATATTGCACAATGGGCGCAAGCCTGAT | 5296 | 1.1189851379504148 | No Hit |
| CCTACGGGAGGCAGCAGTAGGGAATCTTCCGCAATGGGCGAAAGCCTGAC | 5228 | 1.1046175040039214 | No Hit |
| CCTACGGGGGGCAGCAGTGGGGAATATTGGACAATGGGCGCAAGCCTGAT | 5099 | 1.0773612572524858 | No Hit |
| CCTACGGGGGGCAGCAGTGGGGAATATTGCACAATGGGCGAAAGCCTGAT | 4938 | 1.043343770996818 | No Hit |
| CCTACGGGAGGCAGCAGTGGGGAATATTGGACAATGGGCGCAAGCCTGAT | 4825 | 1.0194681439974984 | No Hit |
| CCTACGGGTGGCAGCAGTAGGGAATCTTCCGCAATGGGCGAAAGCCTGAC | 4823 | 1.0190455665284839 | No Hit |
| CCTACGGGCGGCAGCAGTGGGGAATATTGGACAATGGGCGAAAGCCTGAT | 4688 | 0.9905215873700046 | No Hit |
| CCTACGGGCGGCTGCAGTGGGGAATATTGGACAATGGGCGCAAGCCTGAT | 4627 | 0.9776329745650622 | No Hit |
| CCTACGGGAGGCAGCAGTGGGGAATATTGCACAATGGGCGAAAGCCTGAT | 4558 | 0.9630540518840616 | No Hit |
| CCTACGGGAGGCTGCAGTAGGGAATCTTCCGCAATGGGCGAAAGCCTGAC | 4387 | 0.9269236782833213 | No Hit |
| CCTACGGGCGGCTGCAGTGGGGAATATTGCACAATGGGCGAAAGCCTGAT | 4381 | 0.9256559458762778 | No Hit |
| CCTACGGGTGGCTGCAGTAGGGAATCTTCCGCAATGGGCGAAAGCCTGAC | 4335 | 0.9159366640889441 | No Hit |
| CCTACGGGCGGCAGCAGTGGGGAATATTGCACAATGGGCGCAAGCCTGAT | 4247 | 0.8973432554523058 | No Hit |
| CCTACGGGTGGCAGCAGTGGGGAATATTGGACAATGGGCGCAAGCCTGAT | 4108 | 0.8679741213557977 | No Hit |
| CCTACGGGTGGCAGCAGTGGGGAATATTGCACAATGGGCGAAAGCCTGAT | 4105 | 0.8673402551522759 | No Hit |
| CCTACGGGGGGCTGCAGTAGGGAATCTTCCGCAATGGGCGAAAGCCTGAC | 4081 | 0.8622693255241016 | No Hit |
| CCTACGGGAGGCTGCAGTGGGGAATATTGCACAATGGGGGAAACCCTGAT | 3852 | 0.8138842053219406 | No Hit |
| CCTACGGGTGGCTGCAGTGGGGAATATTGCACAATGGGGGAAACCCTGAT | 3849 | 0.8132503391184188 | No Hit |
| CCTACGGGCGGCAGCAGTAGGGAATCTTCCGCAATGGGCGAAAGCCTGAC | 3810 | 0.805010078472636 | No Hit |
| CCTACGGGGGGCTGCAGTGGGGAATATTGCACAATGGGGGAAACCCTGAT | 3684 | 0.778387697924722 | No Hit |
| CCTACGGGCGGCAGCAGTGGGGAATATTGGACAATGGGCGCAAGCCTGAT | 3456 | 0.7302138664570682 | No Hit |
| CCTACGGGCGGCAGCAGTGGGGAATATTGCACAATGGGCGAAAGCCTGAT | 3407 | 0.7198607184662129 | No Hit |
| CCTACGGGGGGCAGCAGTGGGGAATATTGCACAATGGGGGAAACCCTGAT | 3165 | 0.6687288447154575 | No Hit |
| CCTACGGGCGGCTGCAGTAGGGAATCTTCCGCAATGGGCGAAAGCCTGAC | 3120 | 0.6592208516626311 | No Hit |
| CCTACGGGAGGCTGCAGTGGGGAATATTGCACAATGGGCGGAAGCCTGAT | 3033 | 0.6408387317605 | No Hit |
| CCTACGGGTGGCTGCAGTGGGGAATCTTAGACAATGGGGGCAACCCTGAT | 2993 | 0.6323871823802099 | No Hit |
| CCTACGGGTGGCTGCAGTGGGGAATATTGCACAATGGGCGGAAGCCTGAT | 2982 | 0.6300630063006301 | No Hit |
| CCTACGGGAGGCTGCAGTGGGGAATCTTAGACAATGGGGGCAACCCTGAT | 2870 | 0.6063986680358177 | No Hit |
| CCTACGGGCGGCTGCAGTGGGGAATATTGCACAATGGGGGAAACCCTGAT | 2841 | 0.6002712947351073 | No Hit |
| CCTACGGGAGGCAGCAGTGGGGAATATTGCACAATGGGGGAAACCCTGAT | 2797 | 0.5909745904167881 | No Hit |
| CCTACGGGGGGCTGCAGTGGGGAATATTGCACAATGGGCGGAAGCCTGAT | 2796 | 0.590763301682281 | No Hit |
| CCTACGGGGGGCTGCAGTGGGGAATCTTAGACAATGGGGGCAACCCTGAT | 2712 | 0.5730150479836716 | No Hit |
| CCTACGGGGGGCAGCAGTGGGGAATATTGCACAATGGGCGGAAGCCTGAT | 2553 | 0.5394201391970184 | No Hit |
| CCTACGGGTGGCAGCAGTGGGGAATATTGCACAATGGGGGAAACCCTGAT | 2552 | 0.5392088504625111 | No Hit |
| CCTACGGGTGGCTGCAGTGGGGAATTTTGGACAATGGGCGCAAGCCTGAT | 2400 | 0.5070929628174085 | No Hit |
| CCTACGGGAGGCTGCAGTGGGGAATTTTGGACAATGGGCGCAAGCCTGAT | 2393 | 0.5056139416758577 | No Hit |
| CCTACGGGGGGCAGCAGTGGGGAATCTTAGACAATGGGGGCAACCCTGAT | 2366 | 0.49990914584416185 | No Hit |
| CCTACGGGAGGCAGCAGTGGGGAATCTTAGACAATGGGGGCAACCCTGAT | 2333 | 0.4929366176054225 | No Hit |
| CCTACGGGAGGCAGCAGTGGGGAATATTGCACAATGGGCGGAAGCCTGAT | 2263 | 0.4781464061899147 | No Hit |
| CCTACGGGCGGCTGCAGTGGGGAATCTTAGACAATGGGGGCAACCCTGAT | 2220 | 0.46906099060610285 | No Hit |
| CCTACGGGCGGCTGCAGTGGGGAATATTGCACAATGGGCGGAAGCCTGAT | 2183 | 0.4612433074293345 | No Hit |
| CCTACGGGGGGCTGCAGTGGGGAATTTTGGACAATGGGCGCAAGCCTGAT | 2167 | 0.4578626876772184 | No Hit |
| CCTACGGGTGGCAGCAGTGGGGAATATTGCACAATGGGCGGAAGCCTGAT | 2086 | 0.4407483001821309 | No Hit |
| CCTACGGGTGGCAGCAGTGGGGAATCTTAGACAATGGGGGCAACCCTGAT | 2059 | 0.435043504350435 | No Hit |
| CCTACGGGGGGCAGCAGTGGGGAATTTTGGACAATGGGCGCAAGCCTGAT | 1991 | 0.42067587040394183 | No Hit |
| CCTACGGGCGGCAGCAGTGGGGAATATTGCACAATGGGGGAAACCCTGAT | 1948 | 0.41159045482012985 | No Hit |
| CCTACGGGAGGCAGCAGTGGGGAATTTTGGACAATGGGCGCAAGCCTGAT | 1815 | 0.38348905313066517 | No Hit |
| CCTACGGGTGGCTGCAGTGGGGAATATTGGACAATGGGGGCAACCCTGAT | 1710 | 0.36130373600740356 | No Hit |
| CCTACGGGCGGCAGCAGTGGGGAATATTGCACAATGGGCGGAAGCCTGAT | 1677 | 0.35433120776866417 | No Hit |
| CCTACGGGGGGCTGCAGTGGGGAATATTGGACAATGGGGGCAACCCTGAT | 1656 | 0.34989414434401184 | No Hit |
| CCTACGGGCGGCTGCAGTGGGGAATTTTGGACAATGGGCGCAAGCCTGAT | 1653 | 0.3492602781404901 | No Hit |
| CCTACGGGAGGCTGCAGTGGGGAATATTGGACAATGGGGGCAACCCTGAT | 1629 | 0.34418934851231603 | No Hit |
| CCTACGGGCGGCAGCAGTGGGGAATCTTAGACAATGGGGGCAACCCTGAT | 1597 | 0.3374281090080839 | No Hit |
| CCTACGGGTGGCAGCAGTGGGGAATTTTGGACAATGGGCGCAAGCCTGAT | 1575 | 0.33277975684892436 | No Hit |
| CCTACGGGGGGCAGCAGTAGGGAATCTTCCGCAATGGACGAAAGTCTGAC | 1505 | 0.31798954543341656 | No Hit |
| CCTACGGGAGGCAGCAGTAGGGAATCTTCCGCAATGGACGAAAGTCTGAC | 1430 | 0.30214289034537256 | No Hit |
| CCTACGGGTGGCAGCAGTAGGGAATCTTCCGCAATGGACGAAAGTCTGAC | 1316 | 0.27805597461154563 | No Hit |
| CCTACGGGCGGCAGCAGTGGGGAATTTTGGACAATGGGCGCAAGCCTGAT | 1294 | 0.2734076224523861 | No Hit |
| CCTACGGGGGGCAGCAGTGGGGAATATTGGACAATGGGGGCAACCCTGAT | 1234 | 0.2607302983819509 | No Hit |
| CCTACGGGAGGCAGCAGTGGGGAATATTGGACAATGGGGGCAACCCTGAT | 1213 | 0.25629323495729855 | No Hit |
| CCTACGGGTGGCTGCAGTGGGGAATATTGCGCAATGGGCGAAAGCCTGAC | 1207 | 0.255025502550255 | No Hit |
| CCTACGGGCGGCTGCAGTGGGGAATATTGGACAATGGGGGCAACCCTGAT | 1200 | 0.2535464814087042 | No Hit |
| CCTACGGGAGGCTGCAGTGGGGAATATTGCGCAATGGGCGAAAGCCTGAC | 1177 | 0.24868684051503742 | No Hit |
| CCTACGGGTGGCTGCAGTAGGGAATCTTCCGCAATGGACGAAAGTCTGAC | 1168 | 0.24678524190447212 | No Hit |
| CCTACGGGAGGCTGCAGTAGGGAATCTTCCGCAATGGACGAAAGTCTGAC | 1140 | 0.24086915733826905 | No Hit |
| CCTACGGGTGGCAGCAGTGGGGAATATTGGACAATGGGGGCAACCCTGAT | 1139 | 0.24065786860376176 | No Hit |
| CCTACGGGGGGCTGCAGTAGGGAATCTTCCGCAATGGACGAAAGTCTGAC | 1089 | 0.23009343187839912 | No Hit |
| CCTACGGGAGGCTGCAGTGGGGAATATTGCGCAATGGGCGGAAGCCTGAC | 1066 | 0.22523379098473226 | No Hit |
| CCTACGGGGGGCAGCAGTGGGGAATATTGCGCAATGGGCGAAAGCCTGAC | 1035 | 0.21868384021500742 | No Hit |
| CCTACGGGGGGCTGCAGTGGGGAATATTGCGCAATGGGCGAAAGCCTGAC | 1022 | 0.21593708666641315 | No Hit |
| CCTACGGGCGGCAGCAGTAGGGAATCTTCCGCAATGGACGAAAGTCTGAC | 1008 | 0.21297904438331156 | No Hit |
| CCTACGGGTGGCTGCAGTGGGGAATATTGCGCAATGGGCGGAAGCCTGAC | 992 | 0.20959842463119552 | No Hit |
| CCTACGGGAGGCAGCAGTGGGGAATATTGCGCAATGGGCGAAAGCCTGAC | 990 | 0.20917584716218102 | No Hit |
| CCTACGGGGGGCTGCAGTGGGGAATATTGCGCAATGGGCGGAAGCCTGAC | 916 | 0.19354048080864425 | No Hit |
| CCTACGGGTGGCTGCAGTGGGGAATATTGGACAATGGGGGGAACCCTGAT | 913 | 0.19290661460512248 | No Hit |
| CCTACGGGCGGCTGCAGTAGGGAATCTTCCGCAATGGACGAAAGTCTGAC | 900 | 0.19015986105652818 | No Hit |
| CCTACGGGCGGCAGCAGTGGGGAATATTGGACAATGGGGGCAACCCTGAT | 896 | 0.18931470611849918 | No Hit |
| CCTACGGGAGGCTGCAGTGGGGAATATTGGACAATGGGGGGAACCCTGAT | 864 | 0.18255346661426705 | No Hit |
| CCTACGGGTGGCAGCAGTGGGGAATATTGCGCAATGGGCGAAAGCCTGAC | 861 | 0.1819196004107453 | No Hit |
| CCTACGGGTGGCTGCAGTGGGGAATCTTGCGCAATGGGCGAAAGCCTGAC | 831 | 0.1755809383755277 | No Hit |
| CCTACGGGCGGCTGCAGTGGGGAATATTGCGCAATGGGCGAAAGCCTGAC | 800 | 0.16903098760580282 | No Hit |
| CCTACGGGGGGCAGCAGTGGGGAATATTGCGCAATGGGCGGAAGCCTGAC | 778 | 0.16438263544664325 | No Hit |
| CCTACGGGGGGCTGCAGTGGGGAATCTTGCGCAATGGGCGAAAGCCTGAC | 776 | 0.16396005797762875 | No Hit |
| CCTACGGGCGGCTGCAGTGGGGAATATTGCGCAATGGGCGGAAGCCTGAC | 770 | 0.16269232557058522 | No Hit |
| CCTACGGGAGGCTGCAGTGGGGAATCTTGCGCAATGGGCGAAAGCCTGAC | 767 | 0.16205845936706345 | No Hit |
| CCTACGGGGGGCAGCAGTAGGGAATCTTCCGCAATGGACGCAAGTCTGAC | 752 | 0.15888912834945468 | No Hit |
| CCTACGGGGGGCTGCAGTGGGGAATATTGGACAATGGGGGGAACCCTGAT | 749 | 0.15825526214593288 | No Hit |
| CCTACGGGGGGCAGCAGTGGGGAATCTTGCGCAATGGGCGAAAGCCTGAC | 705 | 0.14895855782761375 | No Hit |
| CCTACGGGCGGCAGCAGTGGGGAATATTGCGCAATGGGCGAAAGCCTGAC | 686 | 0.14494407187197592 | No Hit |
| CCTACGGGAGGCAGCAGTGGGGAATATTGCGCAATGGGCGGAAGCCTGAC | 685 | 0.14473278313746868 | No Hit |
| CCTACGGGGGGCAGCAGTGGGGAATATTGGACAATGGGGGGAACCCTGAT | 678 | 0.1432537619959179 | No Hit |
| CCTACGGGAGGCAGCAGTAGGGAATCTTCCGCAATGGACGCAAGTCTGAC | 678 | 0.1432537619959179 | No Hit |
| CCTACGGGAGGCAGCAGTGGGGAATCTTGCGCAATGGGCGAAAGCCTGAC | 636 | 0.13437963514661325 | No Hit |
| CCTACGGGTGGCAGCAGTGGGGAATATTGCGCAATGGGCGGAAGCCTGAC | 635 | 0.13416834641210598 | No Hit |
| CCTACGGGAGGCAGCAGTGGGGAATATTGGACAATGGGGGGAACCCTGAT | 632 | 0.13353448020858424 | No Hit |
| CCTACGGGCGGCTGCAGTGGGGAATATTGGACAATGGGGGGAACCCTGAT | 627 | 0.13247803653604798 | No Hit |
| CCTACGGGAGGCTGCAGTAGGGAATCTTCCGCAATGGACGCAAGTCTGAC | 614 | 0.12973128298745368 | No Hit |
| CCTACGGGGGGCTGCAGTAGGGAATCTTCCGCAATGGACGCAAGTCTGAC | 601 | 0.12698452943885938 | No Hit |
| CCTACGGGTGGCTGCAGTGGGGAATTTTCCGCAATGGGCGAAAGCCTGAC | 596 | 0.1259280857663231 | No Hit |
| CCTACGGGTGGCAGCAGTGGGGAATATTGGACAATGGGGGGAACCCTGAT | 590 | 0.12466035335927958 | No Hit |
| CCTACGGGCGGCTGCAGTGGGGAATCTTGCGCAATGGGCGAAAGCCTGAC | 587 | 0.12402648715575783 | No Hit |
| CCTACGGGTGGCAGCAGTGGGGAATCTTGCGCAATGGGCGAAAGCCTGAC | 579 | 0.1223361772796998 | No Hit |
| CCTACGGGTGGCTGCAGTAGGGAATCTTCCGCAATGGACGCAAGTCTGAC | 568 | 0.12001200120012002 | No Hit |
| CCTACGGGTGGCAGCAGTAGGGAATCTTCCGCAATGGACGCAAGTCTGAC | 567 | 0.11980071246561275 | No Hit |
| CCTACGGGTGGCTGCAGTGGGGAATATTGGACAATGGGCGGAAGCCTGAT | 560 | 0.11832169132406198 | No Hit |
| CCTACGGGAGGCTGCAGTGGGGAATATTGGACAATGGGCGGAAGCCTGAT | 558 | 0.11789911385504748 | No Hit |
| CCTACGGGCGGCAGCAGTGGGGAATATTGCGCAATGGGCGGAAGCCTGAC | 554 | 0.11705395891701846 | No Hit |
| CCTACGGGTGGCTGCAGTGGGGAATCTTGGACAATGGGGGCAACCCTGAT | 551 | 0.11642009271349671 | No Hit |
| CCTACGGGTGGCTGCAGTGAGGAATATTGGTCAATGGGCGAGAGCCTGAA | 549 | 0.1159975152444822 | No Hit |
| CCTACGGGGGGCTGCAGTGGGGAATTTTCCGCAATGGGCGAAAGCCTGAC | 546 | 0.11536364904096044 | No Hit |
| CCTACGGGAGGCTGCAGTGGGGAATCTTGGACAATGGGGGCAACCCTGAT | 543 | 0.11472978283743868 | No Hit |
| CCTACGGGGGGCTGCAGTGGGGAATATTGGACAATGGGCGGAAGCCTGAT | 541 | 0.11430720536842418 | No Hit |
| CCTACGGGGGGCAGCAGTGGGGAATATTGGACAATGGGCGGAAGCCTGAT | 533 | 0.11261689549236613 | No Hit |
| CCTACGGGAGGCTGCAGTGGGGAATTTTCCGCAATGGGCGAAAGCCTGAC | 519 | 0.10965885320926459 | No Hit |
| CCTACGGGGGGCAGCAGTAGGGAATCTTCCACAATGGACGCAAGTCTGAT | 511 | 0.10796854333320657 | No Hit |
| CCTACGGGAGGCAGCAGTAGGGAATCTTCCACAATGGACGCAAGTCTGAT | 499 | 0.10543307851911951 | No Hit |
| CCTACGGGAGGCTGCAGTGGGGAATCTTAGACAATGGGCGCAAGCCTGAT | 486 | 0.10268632497052521 | No Hit |
| CCTACGGGGGGCAGCAGTGGGGAATCTTGGACAATGGGGGCAACCCTGAT | 481 | 0.10162988129798894 | No Hit |
| CCTACGGGCGGCAGCAGTAGGGAATCTTCCGCAATGGACGCAAGTCTGAC | 480 | 0.1014185925634817 | No Hit |

## Adapter Content

## Kmer Content

| Sequence | Count | PValue | Obs/Exp Max | Max Obs/Exp Position |
| --- | --- | --- | --- | --- |
| ATGCAAA | 20 | 4.3186446E-8 | 316.9754 | 295 |
| TGCCGAG | 10 | 6.824635E-4 | 316.9754 | 295 |
| CCACTGA | 25 | 3.45608E-10 | 316.97537 | 295 |
| GATTCGA | 15 | 5.4326083E-6 | 316.97537 | 295 |
| GAGAGAG | 2625 | 0.0 | 314.56036 | 295 |
| ATGTGAT | 615 | 0.0 | 309.2443 | 295 |
| GTATCAG | 110 | 0.0 | 302.5674 | 295 |
| AGTGTTG | 840 | 0.0 | 298.10782 | 295 |
| AGGCATC | 10 | 8.549808E-4 | 294.0018 | 9 |
| ACGTCAT | 20 | 6.2864274E-8 | 294.0018 | 3 |
| GAGGCAT | 10 | 8.549808E-4 | 294.0018 | 8 |
| TAGTTAT | 30 | 5.456968E-12 | 294.00177 | 3 |
| CCTACGG | 46410 | 0.0 | 292.4814 | 1 |
| CATTCGA | 4820 | 0.0 | 292.31445 | 295 |
| CGGGTGG | 11770 | 0.0 | 292.25327 | 5 |
| TGGCTGC | 6845 | 0.0 | 291.85425 | 9 |
| CTACGGG | 47025 | 0.0 | 291.81357 | 2 |
| GTTTGAA | 310 | 0.0 | 291.41287 | 295 |
| GGGTGGC | 11805 | 0.0 | 291.26227 | 6 |
| GGGAGGC | 12890 | 0.0 | 291.03668 | 6 |

Produced by FastQC (version 0.11.7)
