# Supplementary material for: Assessment of airborne bacteria from a public health institution in Mexico City
Source: PLOS Glob Public Health. 2024 Nov 7;4(11):e0003672. doi: 10.1371/journal.pgph.0003672 (PMC11542838; doi:10.1371/journal.pgph.0003672)
Supplement: S1 Text — (ZIP) [file pgph.0003672.s001.zip › Hospital_16S_QC/21022023_CP1D2_16S_S35_L001_R2_001_fastqc.html]

21022023\_CP1D2\_16S\_S35\_L001\_R2\_001.fastq.gz FastQC Report 

FastQC Report

Wed 15 Mar 2023  
21022023\_CP1D2\_16S\_S35\_L001\_R2\_001.fastq.gz

## Summary

- Basic Statistics
- Per base sequence quality
- Per tile sequence quality
- Per sequence quality scores
- Per base sequence content
- Per sequence GC content
- Per base N content
- Sequence Length Distribution
- Sequence Duplication Levels
- Overrepresented sequences
- Adapter Content
- Kmer Content

## Basic Statistics

| Measure | Value |
| --- | --- |
| Filename | 21022023\_CP1D2\_16S\_S35\_L001\_R2\_001.fastq.gz |
| File type | Conventional base calls |
| Encoding | Sanger / Illumina 1.9 |
| Total Sequences | 473286 |
| Sequences flagged as poor quality | 0 |
| Sequence length | 40-301 |
| %GC | 54 |

## Per base sequence quality

## Per tile sequence quality

## Per sequence quality scores

## Per base sequence content

## Per sequence GC content

## Per base N content

## Sequence Length Distribution

## Sequence Duplication Levels

## Overrepresented sequences

| Sequence | Count | Percentage | Possible Source |
| --- | --- | --- | --- |
| GACTACTGGGGTATCTAATCCTGTTCGCTCCCCATGCTTTCGCTCCTCAG | 9085 | 1.9195581529983983 | No Hit |
| GACTACTGGGGTATCTAATCCTGTTTGCTCCCCACGCTTTCGCACCTCAG | 8394 | 1.7735576374538862 | No Hit |
| GACTACAGGGGTATCTAATCCTGTTCGCTCCCCATGCTTTCGCTCCTCAG | 7842 | 1.6569262560058824 | No Hit |
| GACTACTAGGGTATCTAATCCTGTTCGCTCCCCATGCTTTCGCTCCTCAG | 7718 | 1.6307264529269827 | No Hit |
| GACTACCGGGGTATCTAATCCTGTTCGCTCCCCATGCTTTCGCTCCTCAG | 7622 | 1.6104427344142864 | No Hit |
| GACTACAGGGGTATCTAATCCTGTTTGCTCCCCACGCTTTCGCACCTCAG | 7405 | 1.5645930790262126 | No Hit |
| GACTACTCGGGTATCTAATCCTGTTCGCTCCCCATGCTTTCGCTCCTCAG | 7147 | 1.510080585523341 | No Hit |
| GACTACTAGGGTATCTAATCCTGTTTGCTCCCCACGCTTTCGCACCTCAG | 7127 | 1.505854810833196 | No Hit |
| GACTACCGGGGTATCTAATCCTGTTTGCTCCCCACGCTTTCGCACCTCAG | 7015 | 1.4821904725683837 | No Hit |
| GACTACCAGGGTATCTAATCCTGTTCGCTCCCCATGCTTTCGCTCCTCAG | 6956 | 1.4697244372324556 | No Hit |
| GACTACTCGGGTATCTAATCCTGTTTGCTCCCCACGCTTTCGCACCTCAG | 6811 | 1.439087570728904 | No Hit |
| GACTACAAGGGTATCTAATCCTGTTCGCTCCCCATGCTTTCGCTCCTCAG | 6783 | 1.4331714861627007 | No Hit |
| GACTACAAGGGTATCTAATCCTGTTTGCTCCCCACGCTTTCGCACCTCAG | 6479 | 1.3689397108724957 | No Hit |
| GACTACCAGGGTATCTAATCCTGTTTGCTCCCCACGCTTTCGCACCTCAG | 6462 | 1.3653478023858725 | No Hit |
| GACTACCCGGGTATCTAATCCTGTTCGCTCCCCATGCTTTCGCTCCTCAG | 6156 | 1.3006934496266527 | No Hit |
| GACTACACGGGTATCTAATCCTGTTCGCTCCCCATGCTTTCGCTCCTCAG | 5960 | 1.2592808576632313 | No Hit |
| GACTACCCGGGTATCTAATCCTGTTTGCTCCCCACGCTTTCGCACCTCAG | 5800 | 1.2254746601420705 | No Hit |
| GACTACACGGGTATCTAATCCTGTTTGCTCCCCACGCTTTCGCACCTCAG | 5667 | 1.1973732584526058 | No Hit |
| GACTACTGGGGTATCTAATCCTGTTTGCTCCCCATGCTTTCGTACCTCAG | 5498 | 1.16166546232088 | No Hit |
| GACTACTGGGGTATCTAATCCTGTTTGATCCCCACGCTTTCGCACATCAG | 4916 | 1.0386954188376585 | No Hit |
| GACTACAGGGGTATCTAATCCTGTTTGCTCCCCATGCTTTCGTACCTCAG | 4704 | 0.9939022071221206 | No Hit |
| GACTACCGGGGTATCTAATCCTGTTTGCTCCCCATGCTTTCGTACCTCAG | 4640 | 0.9803797281136565 | No Hit |
| GACTACTAGGGTATCTAATCCTGTTTGCTCCCCATGCTTTCGTACCTCAG | 4625 | 0.9772103970960476 | No Hit |
| GACTACTCGGGTATCTAATCCTGTTTGCTCCCCATGCTTTCGTACCTCAG | 4556 | 0.962631474415047 | No Hit |
| GACTACAGGGGTATCTAATCCTGTTTGATCCCCACGCTTTCGCACATCAG | 4273 | 0.9028367625494945 | No Hit |
| GACTACTAGGGTATCTAATCCTGTTTGATCCCCACGCTTTCGCACATCAG | 4144 | 0.8755805157980587 | No Hit |
| GACTACCGGGGTATCTAATCCTGTTTGATCCCCACGCTTTCGCACATCAG | 4134 | 0.8734676284529862 | No Hit |
| GACTACCAGGGTATCTAATCCTGTTTGCTCCCCATGCTTTCGTACCTCAG | 4103 | 0.8669176776832612 | No Hit |
| GACTACAAGGGTATCTAATCCTGTTTGCTCCCCATGCTTTCGTACCTCAG | 4077 | 0.8614241705860728 | No Hit |
| GACTACTCGGGTATCTAATCCTGTTTGATCCCCACGCTTTCGCACATCAG | 3971 | 0.8390275647283039 | No Hit |
| GACTACCCGGGTATCTAATCCTGTTTGCTCCCCATGCTTTCGTACCTCAG | 3795 | 0.8018407474550272 | No Hit |
| GACTACCAGGGTATCTAATCCTGTTTGATCCCCACGCTTTCGCACATCAG | 3746 | 0.7914875994641718 | No Hit |
| GACTACTGGGGTATCTAATCCTGTTTGCTCCCCACGCTTTCGCGCCTCAG | 3711 | 0.7840924937564178 | No Hit |
| GACTACACGGGTATCTAATCCTGTTTGCTCCCCATGCTTTCGTACCTCAG | 3645 | 0.7701474372789391 | No Hit |
| GACTACAAGGGTATCTAATCCTGTTTGATCCCCACGCTTTCGCACATCAG | 3609 | 0.762541042836678 | No Hit |
| GACTACCCGGGTATCTAATCCTGTTTGATCCCCACGCTTTCGCACATCAG | 3320 | 0.7014785985640817 | No Hit |
| GACTACACGGGTATCTAATCCTGTTTGATCCCCACGCTTTCGCACATCAG | 3309 | 0.699154422484502 | No Hit |
| GACTACTGGGGTATCTAATCCTGTTCGCTCCCCACGCTTTCGCTCCTCAG | 3299 | 0.6970415351394295 | No Hit |
| GACTACAGGGGTATCTAATCCTGTTTGCTCCCCACGCTTTCGCGCCTCAG | 3135 | 0.6623901826802399 | No Hit |
| GACTACTAGGGTATCTAATCCTGTTTGCTCCCCACGCTTTCGCGCCTCAG | 2979 | 0.6294291400971084 | No Hit |
| GACTACTCGGGTATCTAATCCTGTTTGCTCCCCACGCTTTCGCGCCTCAG | 2931 | 0.6192872808407601 | No Hit |
| GACTACCGGGGTATCTAATCCTGTTTGCTCCCCACGCTTTCGCGCCTCAG | 2927 | 0.6184421259027312 | No Hit |
| GACTACTAGGGTATCTAATCCTGTTCGCTCCCCACGCTTTCGCTCCTCAG | 2853 | 0.6028067595491944 | No Hit |
| GACTACCGGGGTATCTAATCCTGTTCGCTCCCCACGCTTTCGCTCCTCAG | 2786 | 0.5886504143372084 | No Hit |
| GACTACAGGGGTATCTAATCCTGTTCGCTCCCCACGCTTTCGCTCCTCAG | 2784 | 0.5882278368681939 | No Hit |
| GACTACAAGGGTATCTAATCCTGTTTGCTCCCCACGCTTTCGCGCCTCAG | 2652 | 0.5603377239132363 | No Hit |
| GACTACCAGGGTATCTAATCCTGTTTGCTCCCCACGCTTTCGCGCCTCAG | 2608 | 0.5510410195949172 | No Hit |
| GACTACTCGGGTATCTAATCCTGTTCGCTCCCCACGCTTTCGCTCCTCAG | 2520 | 0.5324476109582789 | No Hit |
| GACTACCAGGGTATCTAATCCTGTTCGCTCCCCACGCTTTCGCTCCTCAG | 2476 | 0.5231509066399598 | No Hit |
| GACTACCCGGGTATCTAATCCTGTTTGCTCCCCACGCTTTCGCGCCTCAG | 2439 | 0.5153332234631913 | No Hit |
| GACTACAAGGGTATCTAATCCTGTTCGCTCCCCACGCTTTCGCTCCTCAG | 2401 | 0.5073042515519157 | No Hit |
| GACTACACGGGTATCTAATCCTGTTTGCTCCCCACGCTTTCGCGCCTCAG | 2399 | 0.5068816740829012 | No Hit |
| GACTACTGGGGTATCTAATCCTGTTTGCTCCCCACGCTTTCGTGCATGAG | 2366 | 0.49990914584416185 | No Hit |
| GACTACTGGGGTATCTAATCCTGTTTGCTCCCCACGCTTTCGAGCCTCAG | 2363 | 0.4992752796406401 | No Hit |
| GACTACAGGGGTATCTAATCCTGTTTGCTCCCCACGCTTTCGAGCCTCAG | 2260 | 0.477512539986393 | No Hit |
| GACTACCCGGGTATCTAATCCTGTTCGCTCCCCACGCTTTCGCTCCTCAG | 2197 | 0.46420134971243604 | No Hit |
| GACTACTAGGGTATCTAATCCTGTTTGCTCCCCACGCTTTCGAGCCTCAG | 2132 | 0.4504675819694645 | No Hit |
| GACTACACGGGTATCTAATCCTGTTCGCTCCCCACGCTTTCGCTCCTCAG | 2121 | 0.44814340588988477 | No Hit |
| GACTACTGGGGTATCTAATCCTGTTCGCTACCCATGCTTTCGCTCCTCAG | 2115 | 0.44687567348284124 | No Hit |
| GACTACAGGGGTATCTAATCCTGTTTGCTCCCCACGCTTTCGTGCATGAG | 2007 | 0.42405649015605784 | No Hit |
| GACTACCGGGGTATCTAATCCTGTTTGCTCCCCACGCTTTCGTGCATGAG | 1940 | 0.4099001449440719 | No Hit |
| GACTACTCGGGTATCTAATCCTGTTTGCTCCCCACGCTTTCGAGCCTCAG | 1912 | 0.40398406037786877 | No Hit |
| GACTACTAGGGTATCTAATCCTGTTTGCTCCCCACGCTTTCGTGCATGAG | 1887 | 0.39870184201518744 | No Hit |
| GACTACCGGGGTATCTAATCCTGTTTGCTCCCCACGCTTTCGAGCCTCAG | 1881 | 0.3974341096081439 | No Hit |
| GACTACCAGGGTATCTAATCCTGTTTGCTCCCCACGCTTTCGAGCCTCAG | 1876 | 0.39637766593560764 | No Hit |
| GACTACAAGGGTATCTAATCCTGTTTGCTCCCCACGCTTTCGAGCCTCAG | 1870 | 0.3951099335285641 | No Hit |
| GACTACAGGGGTATCTAATCCTGTTCGCTACCCATGCTTTCGCTCCTCAG | 1860 | 0.3929970461834916 | No Hit |
| GACTACTCGGGTATCTAATCCTGTTTGCTCCCCACGCTTTCGTGCATGAG | 1811 | 0.38264389819263617 | No Hit |
| GACTACCGGGGTATCTAATCCTGTTCGCTACCCATGCTTTCGCTCCTCAG | 1802 | 0.3807422995820709 | No Hit |
| GACTACTAGGGTATCTAATCCTGTTCGCTACCCATGCTTTCGCTCCTCAG | 1711 | 0.3615150247419108 | No Hit |
| GACTACCCGGGTATCTAATCCTGTTTGCTCCCCACGCTTTCGAGCCTCAG | 1678 | 0.35454249650317143 | No Hit |
| GACTACTCGGGTATCTAATCCTGTTCGCTACCCATGCTTTCGCTCCTCAG | 1668 | 0.3524296091580989 | No Hit |
| GACTACCAGGGTATCTAATCCTGTTTGCTCCCCACGCTTTCGTGCATGAG | 1657 | 0.35010543307851916 | No Hit |
| GACTACAAGGGTATCTAATCCTGTTTGCTCCCCACGCTTTCGTGCATGAG | 1619 | 0.34207646116724344 | No Hit |
| GACTACACGGGTATCTAATCCTGTTTGCTCCCCACGCTTTCGAGCCTCAG | 1599 | 0.3378506864770984 | No Hit |
| GACTACAAGGGTATCTAATCCTGTTCGCTACCCATGCTTTCGCTCCTCAG | 1555 | 0.3285539821587793 | No Hit |
| GACTACCAGGGTATCTAATCCTGTTCGCTACCCATGCTTTCGCTCCTCAG | 1536 | 0.32453949620314143 | No Hit |
| GACTACACGGGTATCTAATCCTGTTTGCTCCCCACGCTTTCGTGCATGAG | 1489 | 0.3146089256813005 | No Hit |
| GACTACCCGGGTATCTAATCCTGTTTGCTCCCCACGCTTTCGTGCATGAG | 1468 | 0.3101718622566482 | No Hit |
| GACTACCCGGGTATCTAATCCTGTTCGCTACCCATGCTTTCGCTCCTCAG | 1405 | 0.2968606719826912 | No Hit |
| GACTACACGGGTATCTAATCCTGTTCGCTACCCATGCTTTCGCTCCTCAG | 1319 | 0.2786898408150674 | No Hit |
| GACTACTGGGGTATCTAATCCTGTTTGCTCCCCACGCTTTCGAGCCTCAA | 1243 | 0.26263189699251616 | No Hit |
| GACTACTGGGGTATCTAATCCTGTTTGCTCCCCACGCTGTCGCGCCTCAG | 1154 | 0.2438271996213706 | No Hit |
| GACTACAGGGGTATCTAATCCTGTTTGCTCCCCACGCTTTCGAGCCTCAA | 1128 | 0.238333692524182 | No Hit |
| GACTACTAGGGTATCTAATCCTGTTTGCTCCCCACGCTTTCGAGCCTCAA | 1114 | 0.23537565024108045 | No Hit |
| GACTACCGGGGTATCTAATCCTGTTTGCTCCCCACGCTTTCGAGCCTCAA | 1102 | 0.23284018542699342 | No Hit |
| GACTACTGGGGTATCTAATCCTGTTTGCTACCCACGCTTTCGAATCTCAG | 1102 | 0.23284018542699342 | No Hit |
| GACTACTGGGGTATCTAATCCTGTTTGCTCCCCACGCTTTCGTGCCTCAG | 1062 | 0.22438863604670325 | No Hit |
| GACTACTCGGGTATCTAATCCTGTTTGCTCCCCACGCTTTCGAGCCTCAA | 1039 | 0.21952899515303645 | No Hit |
| GACTACCAGGGTATCTAATCCTGTTTGCTCCCCACGCTTTCGAGCCTCAA | 1036 | 0.21889512894951468 | No Hit |
| GACTACTAGGGTATCTAATCCTGTTTGCTCCCCACGCTGTCGCGCCTCAG | 1029 | 0.21741610780796392 | No Hit |
| GACTACAAGGGTATCTAATCCTGTTTGCTCCCCACGCTTTCGAGCCTCAA | 1003 | 0.2119226007107753 | No Hit |
| GACTACAGGGGTATCTAATCCTGTTTGCTCCCCACGCTGTCGCGCCTCAG | 990 | 0.20917584716218102 | No Hit |
| GACTACTCGGGTATCTAATCCTGTTTGCTCCCCACGCTGTCGCGCCTCAG | 959 | 0.20262589639245618 | No Hit |
| GACTACAGGGGTATCTAATCCTGTTTGCTCCCCACGCTTTCGTGCCTCAG | 941 | 0.19882269917132558 | No Hit |
| GACTACCGGGGTATCTAATCCTGTTTGCTCCCCACGCTGTCGCGCCTCAG | 937 | 0.19797754423329658 | No Hit |
| GACTACTGGGGTATCTAATCCTGTTTGCTCCCCACGCTTTCGCACCTGAG | 901 | 0.19037114979103545 | No Hit |
| GACTACAAGGGTATCTAATCCTGTTTGCTCCCCACGCTGTCGCGCCTCAG | 893 | 0.18868083991497742 | No Hit |
| GACTACCAGGGTATCTAATCCTGTTTGCTCCCCACGCTGTCGCGCCTCAG | 889 | 0.1878356849769484 | No Hit |
| GACTACTAGGGTATCTAATCCTGTTTGCTACCCACGCTTTCGAATCTCAG | 886 | 0.18720181877342662 | No Hit |
| GACTACTGGGGTATCTAATCCTGTTCGCTCCCCATGCTTTCGCTTCTCAG | 858 | 0.18128573420722352 | No Hit |
| GACTACACGGGTATCTAATCCTGTTTGCTCCCCACGCTGTCGCGCCTCAG | 857 | 0.18107444547271628 | No Hit |
| GACTACACGGGTATCTAATCCTGTTTGCTCCCCACGCTTTCGAGCCTCAA | 855 | 0.18065186800370178 | No Hit |
| GACTACAGGGGTATCTAATCCTGTTTGCTACCCACGCTTTCGAATCTCAG | 854 | 0.18044057926919455 | No Hit |
| GACTACCGGGGTATCTAATCCTGTTTGCTCCCCACGCTTTCGTGCCTCAG | 851 | 0.17980671306567275 | No Hit |
| GACTACCGGGGTATCTAATCCTGTTTGCTACCCACGCTTTCGAATCTCAG | 847 | 0.17896155812764375 | No Hit |
| GACTACAGGGGTATCTAATCCTGTTCGCTCCCCATGCTTTCGCTTCTCAG | 844 | 0.17832769192412198 | No Hit |
| GACTACCAGGGTATCTAATCCTGTTTGCTACCCACGCTTTCGAATCTCAG | 836 | 0.17663738204806398 | No Hit |
| GACTACTAGGGTATCTAATCCTGTTTGCTCCCCACGCTTTCGTGCCTCAG | 825 | 0.17431320596848418 | No Hit |
| GACTACCCGGGTATCTAATCCTGTTTGCTCCCCACGCTTTCGAGCCTCAA | 824 | 0.17410191723397692 | No Hit |
| GACTACTCGGGTATCTAATCCTGTTTGCTCCCCACGCTTTCGTGCCTCAG | 815 | 0.17220031862341165 | No Hit |
| GACTACCCGGGTATCTAATCCTGTTTGCTCCCCACGCTGTCGCGCCTCAG | 815 | 0.17220031862341165 | No Hit |
| GACTACAGGGGTATCTAATCCTGTTTGCTCCCCACGCTTTCGCACCTGAG | 790 | 0.16691810026073028 | No Hit |
| GACTACTAGGGTATCTAATCCTGTTTGCTCCCCACGCTTTCGCACCTGAG | 785 | 0.16586165658819405 | No Hit |
| GACTACCGGGGTATCTAATCCTGTTTGCTCCCCACGCTTTCGCACCTGAG | 773 | 0.163326191774107 | No Hit |
| GACTACTGGGGTATCTAATCCTGTTTGCTCCCCATGCTTTCGCACCTCAG | 759 | 0.16036814949100545 | No Hit |
| GACTACTCGGGTATCTAATCCTGTTTGCTACCCACGCTTTCGAATCTCAG | 751 | 0.1586778396149474 | No Hit |
| GACTACCCGGGTATCTAATCCTGTTTGCTACCCACGCTTTCGAATCTCAG | 749 | 0.15825526214593288 | No Hit |
| GACTACCAGGGTATCTAATCCTGTTTGCTCCCCACGCTTTCGTGCCTCAG | 729 | 0.15402948745578784 | No Hit |
| GACTACTCGGGTATCTAATCCTGTTTGCTCCCCACGCTTTCGCACCTGAG | 727 | 0.15360690998677332 | No Hit |
| GACTACAAGGGTATCTAATCCTGTTTGCTACCCACGCTTTCGAATCTCAG | 722 | 0.15255046631423708 | No Hit |
| GACTACTCGGGTATCTAATCCTGTTCGCTCCCCATGCTTTCGCTTCTCAG | 719 | 0.15191660011071528 | No Hit |
| GACTACCCGGGTATCTAATCCTGTTTGCTCCCCACGCTTTCGTGCCTCAG | 709 | 0.14980371276564275 | No Hit |
| GACTACTAGGGTATCTAATCCTGTTCGCTCCCCATGCTTTCGCTTCTCAG | 700 | 0.14790211415507748 | No Hit |
| GACTACAAGGGTATCTAATCCTGTTTGCTCCCCACGCTTTCGTGCCTCAG | 693 | 0.14642309301352668 | No Hit |
| GACTACACGGGTATCTAATCCTGTTTGCTCCCCACGCTTTCGTGCCTCAG | 683 | 0.14431020566845418 | No Hit |
| GACTACACGGGTATCTAATCCTGTTTGCTACCCACGCTTTCGAATCTCAG | 681 | 0.14388762819943965 | No Hit |
| GACTACCCGGGTATCTAATCCTGTTTGCTCCCCACGCTTTCGCACCTGAG | 667 | 0.1409295859163381 | No Hit |
| GACTACCGGGGTATCTAATCCTGTTCGCTCCCCATGCTTTCGCTTCTCAG | 665 | 0.1405070084473236 | No Hit |
| GACTACCAGGGTATCTAATCCTGTTCGCTCCCCATGCTTTCGCTTCTCAG | 664 | 0.14029571971281635 | No Hit |
| GACTACCAGGGTATCTAATCCTGTTTGCTCCCCACGCTTTCGCACCTGAG | 663 | 0.14008443097830908 | No Hit |
| GACTACAAGGGTATCTAATCCTGTTTGCTCCCCACGCTTTCGCACCTGAG | 658 | 0.13902798730577282 | No Hit |
| GACTACACGGGTATCTAATCCTGTTTGCTCCCCACGCTTTCGCACCTGAG | 651 | 0.13754896616422205 | No Hit |
| GACTACTGGGGTATCTAATCCTGTTCGCTCCCCACACTTTCGCTCCTCAG | 648 | 0.1369150999607003 | No Hit |
| GACTACAAGGGTATCTAATCCTGTTCGCTCCCCATGCTTTCGCTTCTCAG | 641 | 0.1354360788191495 | No Hit |
| GACTACTAGGGTATCTAATCCTGTTTGCTCCCCATGCTTTCGCACCTCAG | 615 | 0.12994257172196094 | No Hit |
| GACTACCGGGGTATCTAATCCTGTTTGCTCCCCATGCTTTCGCACCTCAG | 610 | 0.12888612804942468 | No Hit |
| GACTACACGGGTATCTAATCCTGTTCGCTCCCCATGCTTTCGCTTCTCAG | 608 | 0.12846355058041017 | No Hit |
| GACTACAGGGGTATCTAATCCTGTTTGCTCCCCATGCTTTCGCACCTCAG | 607 | 0.12825226184590288 | No Hit |
| GACTACCCGGGTATCTAATCCTGTTCGCTCCCCATGCTTTCGCTTCTCAG | 594 | 0.1255055082973086 | No Hit |
| GACTACCAGGGTATCTAATCCTGTTTGCTCCCCATGCTTTCGCACCTCAG | 582 | 0.12297004348322156 | No Hit |
| GACTACTGGGGTATCTAATCCTGTTCGCTACCCATGCTTTCGAGCCTCAG | 568 | 0.12001200120012002 | No Hit |
| GACTACTGGGGTATCTAATCCTGTTTGCTACCCACACTTTCGAGCCTCAG | 560 | 0.11832169132406198 | No Hit |
| GACTACTCGGGTATCTAATCCTGTTCGCTCCCCACACTTTCGCTCCTCAG | 553 | 0.11684267018251121 | No Hit |
| GACTACTCGGGTATCTAATCCTGTTTGCTCCCCATGCTTTCGCACCTCAG | 551 | 0.11642009271349671 | No Hit |
| GACTACAAGGGTATCTAATCCTGTTCGCTCCCCACACTTTCGCTCCTCAG | 550 | 0.11620880397898944 | No Hit |
| GACTACTAGGGTATCTAATCCTGTTCGCTCCCCACACTTTCGCTCCTCAG | 547 | 0.11557493777546769 | No Hit |
| GACTACAGGGGTATCTAATCCTGTTCGCTCCCCACACTTTCGCTCCTCAG | 546 | 0.11536364904096044 | No Hit |
| GACTACTAGGGTATCTAATCCTGTTTGCTACCCACACTTTCGAGCCTCAG | 535 | 0.11303947296138064 | No Hit |
| GACTACCGGGGTATCTAATCCTGTTCGCTCCCCACACTTTCGCTCCTCAG | 534 | 0.11282818422687341 | No Hit |
| GACTACAAGGGTATCTAATCCTGTTTGCTCCCCATGCTTTCGCACCTCAG | 533 | 0.11261689549236613 | No Hit |
| GACTACCGGGGTATCTAATCCTGTTTGCTACCCACACTTTCGAGCCTCAG | 532 | 0.11240560675785888 | No Hit |
| GACTACACGGGTATCTAATCCTGTTCGCTCCCCACACTTTCGCTCCTCAG | 527 | 0.11134916308532261 | No Hit |
| GACTACACGGGTATCTAATCCTGTTTGCTCCCCATGCTTTCGCACCTCAG | 518 | 0.10944756447475734 | No Hit |
| GACTACAGGGGTATCTAATCCTGTTTGCTACCCACACTTTCGAGCCTCAG | 516 | 0.10902498700574281 | No Hit |
| GACTACTGGGGTATCTAATCCTGTTCGCTCCCCACGCTTTCGTGCCTCAG | 514 | 0.10860240953672831 | No Hit |
| GACTACCAGGGTATCTAATCCTGTTCGCTCCCCACACTTTCGCTCCTCAG | 512 | 0.10817983206771382 | No Hit |
| GACTACTGGGGTATCTAAGCCTGTTCGCTCCCCACGCTTTCGCTCCTCAG | 501 | 0.10585565598813403 | No Hit |
| GACTACTAGGGTATCTAATCCTGTTCGCTACCCATGCTTTCGAGCCTCAG | 493 | 0.104165346112076 | No Hit |
| GACTACAAGGGTATCTAATCCTGTTTGCTACCCACACTTTCGAGCCTCAG | 493 | 0.104165346112076 | No Hit |
| GACTACAGGGGTATCTAATCCTGTTCGCTACCCATGCTTTCGAGCCTCAG | 489 | 0.10332019117404699 | No Hit |
| GACTACCCGGGTATCTAATCCTGTTCGCTCCCCACACTTTCGCTCCTCAG | 481 | 0.10162988129798894 | No Hit |
| GACTACTCGGGTATCTAATCCTGTTTGCTACCCACACTTTCGAGCCTCAG | 478 | 0.10099601509446719 | No Hit |

## Adapter Content

## Kmer Content

| Sequence | Count | PValue | Obs/Exp Max | Max Obs/Exp Position |
| --- | --- | --- | --- | --- |
| GTTAGGG | 25 | 0.0 | 4754.021 | 295 |
| GTTAGAT | 5 | 3.617963E-4 | 4754.0205 | 295 |
| GTGAGCG | 5 | 3.617963E-4 | 4754.0205 | 295 |
| GATTGAG | 5 | 3.617963E-4 | 4754.0205 | 295 |
| GTTGGCG | 20 | 0.0 | 4754.0205 | 295 |
| CTTAGAG | 5 | 3.617963E-4 | 4754.0205 | 295 |
| GTTAGCG | 440 | 0.0 | 4591.9517 | 295 |
| TTAGACG | 45 | 0.0 | 4225.796 | 295 |
| TTAGCCG | 1415 | 0.0 | 4132.47 | 295 |
| TTAGGCG | 85 | 0.0 | 3635.4275 | 295 |
| GTTAGAG | 20 | 3.3833203E-10 | 3565.5154 | 295 |
| GTTAGAA | 20 | 3.3833203E-10 | 3565.5154 | 295 |
| GATAGCG | 20 | 3.3833203E-10 | 3565.5154 | 295 |
| GTTTGCG | 45 | 0.0 | 3169.347 | 295 |
| GTTAGCA | 55 | 0.0 | 3025.286 | 295 |
| TTTGCCG | 90 | 0.0 | 2905.2349 | 295 |
| ATAGGCG | 10 | 0.001446983 | 2377.0103 | 295 |
| ATTACGT | 10 | 0.001446983 | 2377.0103 | 295 |
| GTTAGTG | 10 | 0.001446983 | 2377.0103 | 295 |
| GGTAGCG | 25 | 3.1580894E-6 | 1901.6083 | 295 |

Produced by FastQC (version 0.11.7)
