# Supplementary material for: Assessment of airborne bacteria from a public health institution in Mexico City
Source: PLOS Glob Public Health. 2024 Nov 7;4(11):e0003672. doi: 10.1371/journal.pgph.0003672 (PMC11542838; doi:10.1371/journal.pgph.0003672)
Supplement: S1 Text — (ZIP) [file pgph.0003672.s001.zip › Hospital_16S_QC/21022023_CP1D3_16S_S36_L001_R1_001_fastqc.html]

21022023\_CP1D3\_16S\_S36\_L001\_R1\_001.fastq.gz FastQC Report 

FastQC Report

Wed 15 Mar 2023  
21022023\_CP1D3\_16S\_S36\_L001\_R1\_001.fastq.gz

## Summary

- Basic Statistics
- Per base sequence quality
- Per tile sequence quality
- Per sequence quality scores
- Per base sequence content
- Per sequence GC content
- Per base N content
- Sequence Length Distribution
- Sequence Duplication Levels
- Overrepresented sequences
- Adapter Content
- Kmer Content

## Basic Statistics

| Measure | Value |
| --- | --- |
| Filename | 21022023\_CP1D3\_16S\_S36\_L001\_R1\_001.fastq.gz |
| File type | Conventional base calls |
| Encoding | Sanger / Illumina 1.9 |
| Total Sequences | 824902 |
| Sequences flagged as poor quality | 0 |
| Sequence length | 51-301 |
| %GC | 55 |

## Per base sequence quality

## Per tile sequence quality

## Per sequence quality scores

## Per base sequence content

## Per sequence GC content

## Per base N content

## Sequence Length Distribution

## Sequence Duplication Levels

## Overrepresented sequences

| Sequence | Count | Percentage | Possible Source |
| --- | --- | --- | --- |
| CCTACGGGAGGCTGCAGTGGGGAATATTGGACAATGGGCGAAAGCCTGAT | 12740 | 1.5444258833170486 | No Hit |
| CCTACGGGTGGCTGCAGTGGGGAATATTGGACAATGGGCGAAAGCCTGAT | 12659 | 1.5346065350793185 | No Hit |
| CCTACGGGGGGCTGCAGTGGGGAATATTGGACAATGGGCGAAAGCCTGAT | 12062 | 1.4622343017716044 | No Hit |
| CCTACGGGGGGCAGCAGTGGGGAATATTGGACAATGGGCGAAAGCCTGAT | 11969 | 1.4509602352764328 | No Hit |
| CCTACGGGAGGCTGCAGTGGGGAATCTTAGACAATGGGGGCAACCCTGAT | 10548 | 1.2786973482910697 | No Hit |
| CCTACGGGAGGCTGCAGTGGGGAATATTGGACAATGGGCGCAAGCCTGAT | 10533 | 1.276878950469268 | No Hit |
| CCTACGGGAGGCAGCAGTGGGGAATATTGGACAATGGGCGAAAGCCTGAT | 10528 | 1.2762728178620004 | No Hit |
| CCTACGGGTGGCTGCAGTGGGGAATATTGGACAATGGGCGCAAGCCTGAT | 10448 | 1.266574696145724 | No Hit |
| CCTACGGGAGGCTGCAGTGGGGAATATTGCACAATGGGCGCAAGCCTGAT | 10439 | 1.265483657452643 | No Hit |
| CCTACGGGTGGCTGCAGTGGGGAATCTTAGACAATGGGGGCAACCCTGAT | 10111 | 1.2257213584159088 | No Hit |
| CCTACGGGGGGCTGCAGTGGGGAATCTTAGACAATGGGGGCAACCCTGAT | 9968 | 1.2083859658480645 | No Hit |
| CCTACGGGTGGCTGCAGTGGGGAATATTGCACAATGGGCGCAAGCCTGAT | 9962 | 1.2076586067193438 | No Hit |
| CCTACGGGGGGCTGCAGTGGGGAATATTGGACAATGGGCGCAAGCCTGAT | 9941 | 1.205112849768821 | No Hit |
| CCTACGGGGGGCAGCAGTGGGGAATATTGCACAATGGGCGCAAGCCTGAT | 9879 | 1.1975968054387065 | No Hit |
| CCTACGGGGGGCAGCAGTGGGGAATATTGGACAATGGGCGCAAGCCTGAT | 9879 | 1.1975968054387065 | No Hit |
| CCTACGGGGGGCAGCAGTGGGGAATCTTAGACAATGGGGGCAACCCTGAT | 9812 | 1.1894746285013251 | No Hit |
| CCTACGGGGGGCTGCAGTGGGGAATATTGCACAATGGGCGCAAGCCTGAT | 9753 | 1.182322263735571 | No Hit |
| CCTACGGGTGGCAGCAGTGGGGAATATTGGACAATGGGCGAAAGCCTGAT | 9562 | 1.1591679981379606 | No Hit |
| CCTACGGGCGGCTGCAGTGGGGAATATTGGACAATGGGCGAAAGCCTGAT | 8908 | 1.0798858531073994 | No Hit |
| CCTACGGGAGGCAGCAGTGGGGAATATTGGACAATGGGCGCAAGCCTGAT | 8742 | 1.0597622505461255 | No Hit |
| CCTACGGGAGGCTGCAGTGGGGAATATTGCACAATGGGCGAAAGCCTGAT | 8628 | 1.0459424271004314 | No Hit |
| CCTACGGGAGGCAGCAGTGGGGAATATTGCACAATGGGCGCAAGCCTGAT | 8556 | 1.0372141175557823 | No Hit |
| CCTACGGGAGGCAGCAGTGGGGAATCTTAGACAATGGGGGCAACCCTGAT | 8244 | 0.9993914428623036 | No Hit |
| CCTACGGGTGGCTGCAGTGGGGAATATTGCACAATGGGCGAAAGCCTGAT | 8203 | 0.9944211554827119 | No Hit |
| CCTACGGGGGGCTGCAGTGGGGAATATTGCACAATGGGCGAAAGCCTGAT | 7894 | 0.9569621603535935 | No Hit |
| CCTACGGGGGGCAGCAGTGGGGAATATTGCACAATGGGCGAAAGCCTGAT | 7823 | 0.948355077330398 | No Hit |
| CCTACGGGTGGCAGCAGTGGGGAATATTGGACAATGGGCGCAAGCCTGAT | 7697 | 0.9330805356272623 | No Hit |
| CCTACGGGCGGCAGCAGTGGGGAATATTGGACAATGGGCGAAAGCCTGAT | 7671 | 0.9299286460694726 | No Hit |
| CCTACGGGTGGCAGCAGTGGGGAATCTTAGACAATGGGGGCAACCCTGAT | 7530 | 0.912835706544535 | No Hit |
| CCTACGGGCGGCTGCAGTGGGGAATCTTAGACAATGGGGGCAACCCTGAT | 7461 | 0.9044710765642463 | No Hit |
| CCTACGGGTGGCAGCAGTGGGGAATATTGCACAATGGGCGCAAGCCTGAT | 7292 | 0.8839837944386121 | No Hit |
| CCTACGGGCGGCTGCAGTGGGGAATATTGGACAATGGGCGCAAGCCTGAT | 7145 | 0.8661634957849539 | No Hit |
| CCTACGGGCGGCTGCAGTGGGGAATATTGCACAATGGGCGCAAGCCTGAT | 7097 | 0.8603446227551879 | No Hit |
| CCTACGGGAGGCAGCAGTGGGGAATATTGCACAATGGGCGAAAGCCTGAT | 6949 | 0.8424030975800763 | No Hit |
| CCTACGGGAGGCTGCAGTGGGGAATCTTAGACAATGGGGGAAACCCTGAT | 6248 | 0.7574233060412024 | No Hit |
| CCTACGGGCGGCAGCAGTGGGGAATATTGGACAATGGGCGCAAGCCTGAT | 6247 | 0.757302079519749 | No Hit |
| CCTACGGGCGGCAGCAGTGGGGAATCTTAGACAATGGGGGCAACCCTGAT | 6190 | 0.750392167796902 | No Hit |
| CCTACGGGTGGCAGCAGTGGGGAATATTGCACAATGGGCGAAAGCCTGAT | 6177 | 0.748816223018007 | No Hit |
| CCTACGGGCGGCAGCAGTGGGGAATATTGCACAATGGGCGCAAGCCTGAT | 5946 | 0.7208128965622583 | No Hit |
| CCTACGGGCGGCTGCAGTGGGGAATATTGCACAATGGGCGAAAGCCTGAT | 5878 | 0.7125694931034232 | No Hit |
| CCTACGGGTGGCTGCAGTGGGGAATCTTAGACAATGGGGGAAACCCTGAT | 5858 | 0.7101449626743541 | No Hit |
| CCTACGGGGGGCTGCAGTGGGGAATCTTAGACAATGGGGGAAACCCTGAT | 5784 | 0.7011742000867982 | No Hit |
| CCTACGGGGGGCAGCAGTGGGGAATCTTAGACAATGGGGGAAACCCTGAT | 5529 | 0.6702614371161666 | No Hit |
| CCTACGGGAGGCTGCAGTGGGGAATATTGCACAATGGGCGGAAGCCTGAT | 5380 | 0.6521986854196014 | No Hit |
| CCTACGGGGGGCAGCAGTAGGGAATCTTCCGCAATGGGCGAAAGCCTGAC | 5223 | 0.6331661215514085 | No Hit |
| CCTACGGGTGGCTGCAGTGGGGAATATTGCACAATGGGCGGAAGCCTGAT | 5185 | 0.6285595137361771 | No Hit |
| CCTACGGGGGGCTGCAGTGGGGAATATTGCACAATGGGCGGAAGCCTGAT | 5033 | 0.6101330824752516 | No Hit |
| CCTACGGGCGGCAGCAGTGGGGAATATTGCACAATGGGCGAAAGCCTGAT | 5031 | 0.6098906294323447 | No Hit |
| CCTACGGGAGGCAGCAGTGGGGAATCTTAGACAATGGGGGAAACCCTGAT | 4901 | 0.5941311816433952 | No Hit |
| CCTACGGGGGGCAGCAGTGGGGAATATTGCACAATGGGCGGAAGCCTGAT | 4900 | 0.5940099551219418 | No Hit |
| CCTACGGGAGGCAGCAGTAGGGAATCTTCCGCAATGGGCGAAAGCCTGAC | 4543 | 0.5507320869630574 | No Hit |
| CCTACGGGTGGCAGCAGTGGGGAATCTTAGACAATGGGGGAAACCCTGAT | 4352 | 0.527577821365447 | No Hit |
| CCTACGGGAGGCAGCAGTGGGGAATATTGCACAATGGGCGGAAGCCTGAT | 4295 | 0.5206679096426 | No Hit |
| CCTACGGGTGGCAGCAGTAGGGAATCTTCCGCAATGGGCGAAAGCCTGAC | 4277 | 0.5184858322564377 | No Hit |
| CCTACGGGCGGCTGCAGTGGGGAATCTTAGACAATGGGGGAAACCCTGAT | 4184 | 0.5072117657612661 | No Hit |
| CCTACGGGAGGCTGCAGTGGGGAATTTTGGACAATGGGCGCAAGCCTGAT | 4070 | 0.49339194231557204 | No Hit |
| CCTACGGGTGGCTGCAGTGGGGAATTTTGGACAATGGGCGCAAGCCTGAT | 3977 | 0.48211787582040044 | No Hit |
| CCTACGGGAGGCTGCAGTGGGGAATTTTCCGCAATGGGCGAAAGCCTGAC | 3932 | 0.4766626823549949 | No Hit |
| CCTACGGGGGGCTGCAGTGGGGAATTTTGGACAATGGGCGCAAGCCTGAT | 3883 | 0.47072258280377544 | No Hit |
| CCTACGGGTGGCTGCAGTGGGGAATTTTCCGCAATGGGCGAAAGCCTGAC | 3867 | 0.4687829584605201 | No Hit |
| CCTACGGGGGGCAGCAGTGGGGAATTTTGGACAATGGGCGCAAGCCTGAT | 3818 | 0.46284285890930077 | No Hit |
| CCTACGGGTGGCAGCAGTGGGGAATATTGCACAATGGGCGGAAGCCTGAT | 3804 | 0.46114568760895236 | No Hit |
| CCTACGGGAGGCTGCAGTAGGGAATCTTCCGCAATGGGCGAAAGCCTGAC | 3744 | 0.4538720963217449 | No Hit |
| CCTACGGGCGGCTGCAGTGGGGAATATTGCACAATGGGCGGAAGCCTGAT | 3636 | 0.4407796320047715 | No Hit |
| CCTACGGGCGGCAGCAGTGGGGAATCTTAGACAATGGGGGAAACCCTGAT | 3616 | 0.43835510157570234 | No Hit |
| CCTACGGGGGGCTGCAGTGGGGAATTTTCCGCAATGGGCGAAAGCCTGAC | 3533 | 0.42829330029506535 | No Hit |
| CCTACGGGGGGCAGCAGTGGGGAATTTTCCGCAATGGGCGAAAGCCTGAC | 3429 | 0.41568574206390574 | No Hit |
| CCTACGGGGGGCTGCAGTAGGGAATCTTCCGCAATGGGCGAAAGCCTGAC | 3410 | 0.41338243815629006 | No Hit |
| CCTACGGGTGGCTGCAGTAGGGAATCTTCCGCAATGGGCGAAAGCCTGAC | 3404 | 0.41265507902756937 | No Hit |
| CCTACGGGAGGCAGCAGTGGGGAATTTTGGACAATGGGCGCAAGCCTGAT | 3299 | 0.39992629427495635 | No Hit |
| CCTACGGGCGGCAGCAGTAGGGAATCTTCCGCAATGGGCGAAAGCCTGAC | 3223 | 0.3907130786444935 | No Hit |
| CCTACGGGAGGCAGCAGTGGGGAATTTTCCGCAATGGGCGAAAGCCTGAC | 3101 | 0.37592344302717173 | No Hit |
| CCTACGGGCGGCAGCAGTGGGGAATATTGCACAATGGGCGGAAGCCTGAT | 3001 | 0.363800790881826 | No Hit |
| CCTACGGGAGGCTGCAGTGGGGAATATTGCGCAATGGGCGGAAGCCTGAC | 2939 | 0.3562847465517116 | No Hit |
| CCTACGGGTGGCAGCAGTGGGGAATTTTGGACAATGGGCGCAAGCCTGAT | 2918 | 0.353738989601189 | No Hit |
| CCTACGGGGGGCAGCAGTAGGGAATCTTCCGCAATGGACGAAAGTCTGAC | 2890 | 0.35034464700049217 | No Hit |
| CCTACGGGCGGCTGCAGTGGGGAATTTTGGACAATGGGCGCAAGCCTGAT | 2841 | 0.34440454744927274 | No Hit |
| CCTACGGGTGGCAGCAGTGGGGAATTTTCCGCAATGGGCGAAAGCCTGAC | 2833 | 0.3434347352776451 | No Hit |
| CCTACGGGGGGCAGCAGTGGGGAATATTGCGCAATGGGCGGAAGCCTGAC | 2808 | 0.34040407224130864 | No Hit |
| CCTACGGGGGGCTGCAGTGGGGAATATTGCGCAATGGGCGGAAGCCTGAC | 2794 | 0.3387069009409603 | No Hit |
| CCTACGGGTGGCTGCAGTGGGGAATATTGCGCAATGGGCGGAAGCCTGAC | 2788 | 0.33797954181223955 | No Hit |
| CCTACGGGTGGCTGCAGTGGGGAATATTGCGCAATGGGCGAAAGCCTGAC | 2746 | 0.3328880279111943 | No Hit |
| CCTACGGGCGGCTGCAGTGGGGAATTTTCCGCAATGGGCGAAAGCCTGAC | 2744 | 0.3326455748682874 | No Hit |
| CCTACGGGGGGCAGCAGTGGGGAATATTGCGCAATGGGCGAAAGCCTGAC | 2742 | 0.33240312182538045 | No Hit |
| CCTACGGGGGGCTGCAGTGGGGAATATTGCGCAATGGGCGAAAGCCTGAC | 2714 | 0.32900877922468363 | No Hit |
| CCTACGGGAGGCTGCAGTGGGGAATATTGCGCAATGGGCGAAAGCCTGAC | 2603 | 0.3155526353433499 | No Hit |
| CCTACGGGCGGCAGCAGTGGGGAATTTTGGACAATGGGCGCAAGCCTGAT | 2489 | 0.3017328118976557 | No Hit |
| CCTACGGGAGGCAGCAGTAGGGAATCTTCCGCAATGGACGAAAGTCTGAC | 2460 | 0.29821724277550543 | No Hit |
| CCTACGGGAGGCAGCAGTGGGGAATATTGCGCAATGGGCGGAAGCCTGAC | 2442 | 0.2960351653893432 | No Hit |
| CCTACGGGCGGCTGCAGTAGGGAATCTTCCGCAATGGGCGAAAGCCTGAC | 2431 | 0.2947016736533552 | No Hit |
| CCTACGGGTGGCAGCAGTAGGGAATCTTCCGCAATGGACGAAAGTCTGAC | 2360 | 0.2860945906301597 | No Hit |
| CCTACGGGAGGCAGCAGTGGGGAATATTGCGCAATGGGCGAAAGCCTGAC | 2359 | 0.2859733641087063 | No Hit |
| CCTACGGGCGGCAGCAGTGGGGAATTTTCCGCAATGGGCGAAAGCCTGAC | 2306 | 0.279548358471673 | No Hit |
| CCTACGGGTGGCAGCAGTGGGGAATATTGCGCAATGGGCGGAAGCCTGAC | 2191 | 0.2656073085045254 | No Hit |
| CCTACGGGCGGCTGCAGTGGGGAATATTGCGCAATGGGCGGAAGCCTGAC | 2093 | 0.25372710940208654 | No Hit |
| CCTACGGGTGGCAGCAGTGGGGAATATTGCGCAATGGGCGAAAGCCTGAC | 2076 | 0.2516662585373778 | No Hit |
| CCTACGGGAGGCTGCAGTAGGGAATCTTCCGCAATGGACGAAAGTCTGAC | 2038 | 0.2470596507221464 | No Hit |
| CCTACGGGTGGCTGCAGTAGGGAATCTTCCGCAATGGACGAAAGTCTGAC | 1983 | 0.24039219204220622 | No Hit |
| CCTACGGGGGGCTGCAGTAGGGAATCTTCCGCAATGGACGAAAGTCTGAC | 1940 | 0.23517945161970755 | No Hit |
| CCTACGGGCGGCTGCAGTGGGGAATATTGCGCAATGGGCGAAAGCCTGAC | 1916 | 0.23227001510482456 | No Hit |
| CCTACGGGAGGCTGCAGTGGGGAATATTGGACAATGGGCGGAAGCCTGAT | 1871 | 0.22681482163941896 | No Hit |
| CCTACGGGCGGCAGCAGTGGGGAATATTGCGCAATGGGCGGAAGCCTGAC | 1825 | 0.22123840165255995 | No Hit |
| CCTACGGGCGGCAGCAGTAGGGAATCTTCCGCAATGGACGAAAGTCTGAC | 1800 | 0.2182077386162235 | No Hit |
| CCTACGGGTGGCTGCAGTGGGGAATATTGGACAATGGGGGCAACCCTGAT | 1785 | 0.21638934079442163 | No Hit |
| CCTACGGGAGGCTGCAGTGGGGAATATTGGACAATGGGGGCAACCCTGAT | 1757 | 0.21299499819372483 | No Hit |
| CCTACGGGCGGCAGCAGTGGGGAATATTGCGCAATGGGCGAAAGCCTGAC | 1747 | 0.21178273297919026 | No Hit |
| CCTACGGGGGGCAGCAGTGGGGAATATTGGACAATGGGCGGAAGCCTGAT | 1735 | 0.21032801472174878 | No Hit |
| CCTACGGGTGGCTGCAGTGGGGAATATTGGACAATGGGCGGAAGCCTGAT | 1714 | 0.20778225777122616 | No Hit |
| CCTACGGGGGGCAGCAGTGGGGAATATTGGACAATGGGGGCAACCCTGAT | 1706 | 0.2068124455995985 | No Hit |
| CCTACGGGTGGCTGCAGTGGGGAATCTTAGACAATGGGCGCAAGCCTGAT | 1703 | 0.20644876603523812 | No Hit |
| CCTACGGGGGGCTGCAGTGGGGAATATTGGACAATGGGCGGAAGCCTGAT | 1687 | 0.20450914169198278 | No Hit |
| CCTACGGGGGGCTGCAGTGGGGAATATTGGACAATGGGGGCAACCCTGAT | 1683 | 0.204024235606169 | No Hit |
| CCTACGGGGGGCTGCAGTGGGGAATCTTAGACAATGGGCGCAAGCCTGAT | 1677 | 0.20329687647744824 | No Hit |
| CCTACGGGAGGCTGCAGTGGGGAATCTTAGACAATGGGCGCAAGCCTGAT | 1659 | 0.201114799091286 | No Hit |
| CCTACGGGGGGCAGCAGTGGGGAATCTTAGACAATGGGCGCAAGCCTGAT | 1616 | 0.19590205866878732 | No Hit |
| CCTACGGGAGGCTGCAGTGGGGAATATTGCACAATGGGGGAAACCCTGAT | 1580 | 0.19153790389646286 | No Hit |
| CCTACGGGGGGCTGCAGTGGGGAATATTGCACAATGGGGGAAACCCTGAT | 1563 | 0.18947705303175408 | No Hit |
| CCTACGGGTGGCTGCAGTGGGGAATATTGCACAATGGGGGAAACCCTGAT | 1541 | 0.186810069559778 | No Hit |
| CCTACGGGAGGCAGCAGTGGGGAATCTTAGACAATGGGCGCAAGCCTGAT | 1490 | 0.18062751696565166 | No Hit |
| CCTACGGGAGGCAGCAGTGGGGAATATTGGACAATGGGCGGAAGCCTGAT | 1465 | 0.17759685392931523 | No Hit |
| CCTACGGGGGGCAGCAGTGGGGAATATTGCACAATGGGGGAAACCCTGAT | 1444 | 0.17505109697879262 | No Hit |
| CCTACGGGAGGCAGCAGTGGGGAATATTGGACAATGGGGGCAACCCTGAT | 1434 | 0.17383883176425807 | No Hit |
| CCTACGGGTGGCAGCAGTGGGGAATATTGGACAATGGGCGGAAGCCTGAT | 1377 | 0.16692892004141097 | No Hit |
| CCTACGGGAGGCAGCAGTGGGGAATATTGCACAATGGGGGAAACCCTGAT | 1330 | 0.16123127353309846 | No Hit |
| CCTACGGGCGGCTGCAGTAGGGAATCTTCCGCAATGGACGAAAGTCTGAC | 1329 | 0.16111004701164502 | No Hit |
| CCTACGGGTGGCAGCAGTGGGGAATCTTAGACAATGGGCGCAAGCCTGAT | 1328 | 0.16098882049019156 | No Hit |
| CCTACGGGGGGCAGCAGTGGGGAATTTTGGACAATGGGCGAAAGCCTGAT | 1295 | 0.15698834528222746 | No Hit |
| CCTACGGGGGGCAGCAGTAGGGAATCTTCCGCAATGGACGCAAGTCTGAC | 1281 | 0.15529117398187905 | No Hit |
| CCTACGGGCGGCTGCAGTGGGGAATCTTAGACAATGGGCGCAAGCCTGAT | 1271 | 0.15407890876734448 | No Hit |
| CCTACGGGTGGCAGCAGTGGGGAATATTGGACAATGGGGGCAACCCTGAT | 1261 | 0.1528666435528099 | No Hit |
| CCTACGGGCGGCTGCAGTGGGGAATATTGGACAATGGGCGGAAGCCTGAT | 1253 | 0.15189683138118226 | No Hit |
| CCTACGGGCGGCTGCAGTGGGGAATATTGGACAATGGGGGCAACCCTGAT | 1221 | 0.1480175826946716 | No Hit |
| CCTACGGGAGGCTGCAGTGGGGAATTTTGGACAATGGGCGAAAGCCTGAT | 1181 | 0.1431685218365333 | No Hit |
| CCTACGGGTGGCTGCAGTGGGGAATTTTGGACAATGGGCGAAAGCCTGAT | 1177 | 0.14268361575071947 | No Hit |
| CCTACGGGGGGCTGCAGTGGGGAATTTTGGACAATGGGCGAAAGCCTGAT | 1164 | 0.14110767097182453 | No Hit |
| CCTACGGGTGGCTGCAGTGGGGAATTTTGGACAATGGGGGCAACCCTGAT | 1149 | 0.13928927315002268 | No Hit |
| CCTACGGGAGGCAGCAGTGGGGAATTTTGGACAATGGGCGAAAGCCTGAT | 1122 | 0.13601615707077933 | No Hit |
| CCTACGGGCGGCAGCAGTGGGGAATCTTAGACAATGGGCGCAAGCCTGAT | 1113 | 0.1349251183776982 | No Hit |
| CCTACGGGTGGCAGCAGTGGGGAATATTGCACAATGGGGGAAACCCTGAT | 1113 | 0.1349251183776982 | No Hit |
| CCTACGGGAGGCTGCAGTGGGGAATTTTGGACAATGGGGGCAACCCTGAT | 1094 | 0.1326218144700825 | No Hit |
| CCTACGGGAGGCAGCAGTAGGGAATCTTCCGCAATGGACGCAAGTCTGAC | 1094 | 0.1326218144700825 | No Hit |
| CCTACGGGCGGCAGCAGTGGGGAATATTGGACAATGGGGGCAACCCTGAT | 1074 | 0.13019728404101336 | No Hit |
| CCTACGGGCGGCTGCAGTGGGGAATATTGCACAATGGGGGAAACCCTGAT | 1071 | 0.129833604476653 | No Hit |
| CCTACGGGGGGCTGCAGTGGGGAATTTTGGACAATGGGGGCAACCCTGAT | 1068 | 0.12946992491229262 | No Hit |
| CCTACGGGTGGCAGCAGTAGGGAATCTTCCGCAATGGACGCAAGTCTGAC | 1063 | 0.12886379230502532 | No Hit |
| CCTACGGGCGGCAGCAGTGGGGAATATTGGACAATGGGCGGAAGCCTGAT | 1054 | 0.1277727536119442 | No Hit |
| CCTACGGGTGGCAGCAGTGGGGAATTTTGGACAATGGGCGAAAGCCTGAT | 1051 | 0.12740907404758384 | No Hit |
| CCTACGGGGGGCAGCAGTGGGGAATATTGGACAATGGGGGGAACCCTGAT | 1045 | 0.12668171491886307 | No Hit |
| CCTACGGGGGGCAGCAGTGGGGAATTTTGGACAATGGGGGCAACCCTGAT | 1037 | 0.12571190274723543 | No Hit |
| CCTACGGGGGGCAGCAGTAGGGAATCTTCCACAATGGACGAAAGTCTGAT | 1031 | 0.12498454361851469 | No Hit |
| CCTACGGGAGGCAGCAGTAGGGAATCTTCCACAATGGACGAAAGTCTGAT | 1018 | 0.12340859883961973 | No Hit |
| CCTACGGGTGGCTGCAGTGGGGAATATTGGACAATGGGGGGAACCCTGAT | 1012 | 0.12268123971089899 | No Hit |
| CCTACGGGAGGCTGCAGTGGGGAATATTGGACAATGGGGGGAACCCTGAT | 1007 | 0.1220751071036317 | No Hit |
| CCTACGGGGGGCTGCAGTGGGGAATATTGGACAATGGGGGGAACCCTGAT | 999 | 0.12110529493200403 | No Hit |
| CCTACGGGCGGCAGCAGTGGGGAATATTGCACAATGGGGGAAACCCTGAT | 929 | 0.11261943843026202 | No Hit |
| CCTACGGGAGGCTGCAGTGGGGAATCTTGCGCAATGGGCGAAAGCCTGAC | 923 | 0.11189207930154127 | No Hit |
| CCTACGGGTGGCAGCAGTAGGGAATCTTCCACAATGGACGAAAGTCTGAT | 914 | 0.11080104060846015 | No Hit |
| CCTACGGGAGGCTGCAGTAGGGAATCTTCCGCAATGGACGCAAGTCTGAC | 910 | 0.11031613452264631 | No Hit |
| CCTACGGGCGGCTGCAGTGGGGAATTTTGGACAATGGGCGAAAGCCTGAT | 909 | 0.11019490800119287 | No Hit |
| CCTACGGGGGGCAGCAGTGGGGAATCTTGCGCAATGGGCGAAAGCCTGAC | 902 | 0.10934632235101867 | No Hit |
| CCTACGGGTGGCTGCAGTGGGGAATCTTGCGCAATGGGCGAAAGCCTGAC | 892 | 0.1081340571364841 | No Hit |
| CCTACGGGAGGCAGCAGTGGGGAATATTGGACAATGGGGGGAACCCTGAT | 880 | 0.1066793388790426 | No Hit |
| CCTACGGGGGGCTGCAGTGGGGAATCTTGCGCAATGGGCGAAAGCCTGAC | 869 | 0.10534584714305456 | No Hit |
| CCTACGGGAGGCAGCAGTGGGGAATCTTGCGCAATGGGCGAAAGCCTGAC | 868 | 0.1052246206216011 | No Hit |
| CCTACGGGTGGCTGCAGTAGGGAATCTTCCGCAATGGACGCAAGTCTGAC | 863 | 0.10461848801433384 | No Hit |
| CCTACGGGAGGCAGCAGTGGGGAATTTTGGACAATGGGGGCAACCCTGAT | 849 | 0.10292131671398543 | No Hit |
| CCTACGGGAGGCTGCAGTAGGGAATCTTCCACAATGGACGAAAGTCTGAT | 834 | 0.10110291889218355 | No Hit |
| CCTACGGGCGGCAGCAGTGGGGAATTTTGGACAATGGGCGAAAGCCTGAT | 832 | 0.10086046584927663 | No Hit |
| CCTACGGGCGGCAGCAGTAGGGAATCTTCCGCAATGGACGCAAGTCTGAC | 828 | 0.10037555976346281 | No Hit |
| CCTACGGGAGGCTGCAGTGGGGAATCTTGGACAATGGGGGCAACCCTGAT | 826 | 0.10013310672055589 | No Hit |
| CCTACGGGGGGCTGCAGTAGGGAATCTTCCGCAATGGACGCAAGTCTGAC | 825 | 0.10001188019910243 | No Hit |

## Adapter Content

## Kmer Content

| Sequence | Count | PValue | Obs/Exp Max | Max Obs/Exp Position |
| --- | --- | --- | --- | --- |
| CAAAGAG | 25 | 3.3469405E-10 | 318.59015 | 295 |
| ATGTGAT | 615 | 0.0 | 315.99997 | 295 |
| GAGAGAG | 12745 | 0.0 | 315.46548 | 295 |
| AGTGTTG | 2030 | 0.0 | 309.95837 | 295 |
| ATCCAAA | 3210 | 0.0 | 305.6877 | 295 |
| GGGAGAG | 3090 | 0.0 | 301.06253 | 295 |
| ATTGGAT | 1550 | 0.0 | 297.0082 | 295 |
| TAGTTAT | 20 | 6.290793E-8 | 293.98813 | 3 |
| TAGTAAT | 10 | 8.5521484E-4 | 293.98813 | 3 |
| CCTACGG | 81060 | 0.0 | 292.73648 | 1 |
| GGGAGGC | 22570 | 0.0 | 292.22968 | 6 |
| CTACGGG | 82120 | 0.0 | 292.12653 | 2 |
| TGGCTGC | 11795 | 0.0 | 291.99414 | 9 |
| GTGGCTG | 11825 | 0.0 | 291.3777 | 8 |
| GGGTGGC | 20630 | 0.0 | 291.28052 | 6 |
| CGGGTGG | 20655 | 0.0 | 290.92798 | 5 |
| GGGGCAG | 11825 | 0.0 | 290.5075 | 8 |
| GGGCGGC | 15915 | 0.0 | 290.29364 | 6 |
| GGGCAGC | 11840 | 0.0 | 290.2636 | 9 |
| AGGCTGC | 12465 | 0.0 | 290.0966 | 9 |

Produced by FastQC (version 0.11.7)
