# Supplementary material for: Assessment of airborne bacteria from a public health institution in Mexico City
Source: PLOS Glob Public Health. 2024 Nov 7;4(11):e0003672. doi: 10.1371/journal.pgph.0003672 (PMC11542838; doi:10.1371/journal.pgph.0003672)
Supplement: S1 Text — (ZIP) [file pgph.0003672.s001.zip › Hospital_16S_QC/21022023_CP1D3_16S_S36_L001_R2_001_fastqc.html]

21022023\_CP1D3\_16S\_S36\_L001\_R2\_001.fastq.gz FastQC Report 

FastQC Report

Wed 15 Mar 2023  
21022023\_CP1D3\_16S\_S36\_L001\_R2\_001.fastq.gz

## Summary

- Basic Statistics
- Per base sequence quality
- Per tile sequence quality
- Per sequence quality scores
- Per base sequence content
- Per sequence GC content
- Per base N content
- Sequence Length Distribution
- Sequence Duplication Levels
- Overrepresented sequences
- Adapter Content
- Kmer Content

## Basic Statistics

| Measure | Value |
| --- | --- |
| Filename | 21022023\_CP1D3\_16S\_S36\_L001\_R2\_001.fastq.gz |
| File type | Conventional base calls |
| Encoding | Sanger / Illumina 1.9 |
| Total Sequences | 824902 |
| Sequences flagged as poor quality | 0 |
| Sequence length | 51-301 |
| %GC | 55 |

## Per base sequence quality

## Per tile sequence quality

## Per sequence quality scores

## Per base sequence content

## Per sequence GC content

## Per base N content

## Sequence Length Distribution

## Sequence Duplication Levels

## Overrepresented sequences

| Sequence | Count | Percentage | Possible Source |
| --- | --- | --- | --- |
| GACTACTGGGGTATCTAATCCTGTTTGCTCCCCACGCTTTCGCACCTCAG | 22338 | 2.7079580362273337 | No Hit |
| GACTACAGGGGTATCTAATCCTGTTTGCTCCCCACGCTTTCGCACCTCAG | 19199 | 2.3274279853849307 | No Hit |
| GACTACTAGGGTATCTAATCCTGTTTGCTCCCCACGCTTTCGCACCTCAG | 18690 | 2.2657236859651206 | No Hit |
| GACTACCGGGGTATCTAATCCTGTTTGCTCCCCACGCTTTCGCACCTCAG | 18597 | 2.254449619469949 | No Hit |
| GACTACTCGGGTATCTAATCCTGTTTGCTCCCCACGCTTTCGCACCTCAG | 17720 | 2.148133960155267 | No Hit |
| GACTACCAGGGTATCTAATCCTGTTTGCTCCCCACGCTTTCGCACCTCAG | 16879 | 2.046182455612909 | No Hit |
| GACTACAAGGGTATCTAATCCTGTTTGCTCCCCACGCTTTCGCACCTCAG | 16871 | 2.0452126434412814 | No Hit |
| GACTACCCGGGTATCTAATCCTGTTTGCTCCCCACGCTTTCGCACCTCAG | 15120 | 1.8329450043762776 | No Hit |
| GACTACACGGGTATCTAATCCTGTTTGCTCCCCACGCTTTCGCACCTCAG | 14779 | 1.7916067605606485 | No Hit |
| GACTACTGGGGTATCTAATCCTGTTCGCTCCCCATGCTTTCGCTCCTCAG | 13311 | 1.6136462270669727 | No Hit |
| GACTACAGGGGTATCTAATCCTGTTCGCTCCCCATGCTTTCGCTCCTCAG | 11462 | 1.3894983888995298 | No Hit |
| GACTACTAGGGTATCTAATCCTGTTCGCTCCCCATGCTTTCGCTCCTCAG | 11155 | 1.3522818468133184 | No Hit |
| GACTACCGGGGTATCTAATCCTGTTCGCTCCCCATGCTTTCGCTCCTCAG | 11001 | 1.333612962509486 | No Hit |
| GACTACTCGGGTATCTAATCCTGTTCGCTCCCCATGCTTTCGCTCCTCAG | 10795 | 1.3086402990900736 | No Hit |
| GACTACCAGGGTATCTAATCCTGTTCGCTCCCCATGCTTTCGCTCCTCAG | 10150 | 1.2304491927525938 | No Hit |
| GACTACAAGGGTATCTAATCCTGTTCGCTCCCCATGCTTTCGCTCCTCAG | 9753 | 1.182322263735571 | No Hit |
| GACTACCCGGGTATCTAATCCTGTTCGCTCCCCATGCTTTCGCTCCTCAG | 9102 | 1.1034037982693703 | No Hit |
| GACTACACGGGTATCTAATCCTGTTCGCTCCCCATGCTTTCGCTCCTCAG | 8917 | 1.0809768918004805 | No Hit |
| GACTACTGGGGTATCTAATCCTGTTTGCTCCCCACGCTTTCGCGCCTCAG | 7765 | 0.9413239390860976 | No Hit |
| GACTACTGGGGTATCTAATCCTGTTCGCTCCCCACGCTTTCGCTCCTCAG | 7268 | 0.8810743579237291 | No Hit |
| GACTACTAGGGTATCTAATCCTGTTTGCTCCCCACGCTTTCGCGCCTCAG | 6580 | 0.7976705111637503 | No Hit |
| GACTACAGGGGTATCTAATCCTGTTTGCTCCCCACGCTTTCGCGCCTCAG | 6554 | 0.7945186216059604 | No Hit |
| GACTACAGGGGTATCTAATCCTGTTCGCTCCCCACGCTTTCGCTCCTCAG | 6300 | 0.7637270851567823 | No Hit |
| GACTACCGGGGTATCTAATCCTGTTTGCTCCCCACGCTTTCGCGCCTCAG | 6293 | 0.762878499506608 | No Hit |
| GACTACCGGGGTATCTAATCCTGTTCGCTCCCCACGCTTTCGCTCCTCAG | 6125 | 0.7425124439024272 | No Hit |
| GACTACTAGGGTATCTAATCCTGTTCGCTCCCCACGCTTTCGCTCCTCAG | 6084 | 0.7375421565228355 | No Hit |
| GACTACTCGGGTATCTAATCCTGTTTGCTCCCCACGCTTTCGCGCCTCAG | 6082 | 0.7372997034799285 | No Hit |
| GACTACTGGGGTATCTAATCCTGTTTGCTCCCCACGCTTTCGTGCATGAG | 5907 | 0.7160850622255734 | No Hit |
| GACTACTCGGGTATCTAATCCTGTTCGCTCCCCACGCTTTCGCTCCTCAG | 5838 | 0.7077204322452849 | No Hit |
| GACTACCAGGGTATCTAATCCTGTTTGCTCCCCACGCTTTCGCGCCTCAG | 5837 | 0.7075992057238314 | No Hit |
| GACTACAAGGGTATCTAATCCTGTTTGCTCCCCACGCTTTCGCGCCTCAG | 5808 | 0.7040836366016812 | No Hit |
| GACTACCAGGGTATCTAATCCTGTTCGCTCCCCACGCTTTCGCTCCTCAG | 5389 | 0.6532897241126825 | No Hit |
| GACTACAAGGGTATCTAATCCTGTTCGCTCCCCACGCTTTCGCTCCTCAG | 5228 | 0.6337722541586759 | No Hit |
| GACTACCCGGGTATCTAATCCTGTTTGCTCCCCACGCTTTCGCGCCTCAG | 5201 | 0.6304991380794325 | No Hit |
| GACTACACGGGTATCTAATCCTGTTTGCTCCCCACGCTTTCGCGCCTCAG | 5191 | 0.6292868728648979 | No Hit |
| GACTACTAGGGTATCTAATCCTGTTTGCTCCCCACGCTTTCGTGCATGAG | 5011 | 0.6074660990032755 | No Hit |
| GACTACCCGGGTATCTAATCCTGTTCGCTCCCCACGCTTTCGCTCCTCAG | 4993 | 0.6052840216171134 | No Hit |
| GACTACTGGGGTATCTAATCCTGTTTGCTCCCCACACTTTCGCACCTCAG | 4978 | 0.6034656237953114 | No Hit |
| GACTACAGGGGTATCTAATCCTGTTTGCTCCCCACGCTTTCGTGCATGAG | 4941 | 0.5989802425015335 | No Hit |
| GACTACTCGGGTATCTAATCCTGTTTGCTCCCCACGCTTTCGTGCATGAG | 4761 | 0.5771594686399112 | No Hit |
| GACTACCGGGGTATCTAATCCTGTTTGCTCCCCACGCTTTCGTGCATGAG | 4663 | 0.5652792695374723 | No Hit |
| GACTACACGGGTATCTAATCCTGTTCGCTCCCCACGCTTTCGCTCCTCAG | 4657 | 0.5645519104087516 | No Hit |
| GACTACAAGGGTATCTAATCCTGTTTGCTCCCCACGCTTTCGTGCATGAG | 4485 | 0.5437009487187568 | No Hit |
| GACTACCAGGGTATCTAATCCTGTTTGCTCCCCACGCTTTCGTGCATGAG | 4407 | 0.5342452800453872 | No Hit |
| GACTACAGGGGTATCTAATCCTGTTTGCTCCCCACACTTTCGCACCTCAG | 4305 | 0.5218801748571346 | No Hit |
| GACTACTAGGGTATCTAATCCTGTTTGCTCCCCACACTTTCGCACCTCAG | 4304 | 0.5217589483356811 | No Hit |
| GACTACTGGGGTATCTAATCCTGTTTGATCCCCACGCTTTCGCACATCAG | 4223 | 0.511939600097951 | No Hit |
| GACTACCGGGGTATCTAATCCTGTTTGCTCCCCACACTTTCGCACCTCAG | 4164 | 0.504787235332197 | No Hit |
| GACTACCCGGGTATCTAATCCTGTTTGCTCCCCACGCTTTCGTGCATGAG | 4121 | 0.4995744949096984 | No Hit |
| GACTACTCGGGTATCTAATCCTGTTTGCTCCCCACACTTTCGCACCTCAG | 4048 | 0.49072495884359596 | No Hit |
| GACTACACGGGTATCTAATCCTGTTTGCTCCCCACGCTTTCGTGCATGAG | 3842 | 0.4657522954241837 | No Hit |
| GACTACCAGGGTATCTAATCCTGTTTGCTCCCCACACTTTCGCACCTCAG | 3814 | 0.4623579528234869 | No Hit |
| GACTACAAGGGTATCTAATCCTGTTTGCTCCCCACACTTTCGCACCTCAG | 3758 | 0.45556926762209327 | No Hit |
| GACTACAGGGGTATCTAATCCTGTTTGATCCCCACGCTTTCGCACATCAG | 3656 | 0.4432041624338406 | No Hit |
| GACTACTAGGGTATCTAATCCTGTTTGATCCCCACGCTTTCGCACATCAG | 3650 | 0.4424768033051199 | No Hit |
| GACTACCGGGGTATCTAATCCTGTTTGATCCCCACGCTTTCGCACATCAG | 3590 | 0.4352032120179124 | No Hit |
| GACTACTGGGGTATCTAATCCTGTTTGCTCCCCATGCTTTCGTACCTCAG | 3586 | 0.4347183059320986 | No Hit |
| GACTACTCGGGTATCTAATCCTGTTTGATCCCCACGCTTTCGCACATCAG | 3521 | 0.42683858203762387 | No Hit |
| GACTACCCGGGTATCTAATCCTGTTTGCTCCCCACACTTTCGCACCTCAG | 3520 | 0.4267173555161704 | No Hit |
| GACTACTGGGGTATCTAATCCTGTTTGCTCCCCACGCTTTCGTGCCTCAG | 3423 | 0.41495838293518506 | No Hit |
| GACTACACGGGTATCTAATCCTGTTTGCTCCCCACACTTTCGCACCTCAG | 3402 | 0.4124126259846624 | No Hit |
| GACTACAAGGGTATCTAATCCTGTTTGATCCCCACGCTTTCGCACATCAG | 3278 | 0.3973805373244337 | No Hit |
| GACTACAGGGGTATCTAATCCTGTTTGCTCCCCATGCTTTCGTACCTCAG | 3229 | 0.3914404377732143 | No Hit |
| GACTACCAGGGTATCTAATCCTGTTTGATCCCCACGCTTTCGCACATCAG | 3202 | 0.38816732169397095 | No Hit |
| GACTACCGGGGTATCTAATCCTGTTTGCTCCCCATGCTTTCGTACCTCAG | 3076 | 0.37289277999083525 | No Hit |
| GACTACAGGGGTATCTAATCCTGTTTGCTCCCCACGCTTTCGTGCCTCAG | 3073 | 0.3725291004264749 | No Hit |
| GACTACTAGGGTATCTAATCCTGTTTGCTCCCCATGCTTTCGTACCTCAG | 3019 | 0.3659828682679882 | No Hit |
| GACTACTAGGGTATCTAATCCTGTTTGCTCCCCACGCTTTCGTGCCTCAG | 2897 | 0.3511932326506664 | No Hit |
| GACTACACGGGTATCTAATCCTGTTTGATCCCCACGCTTTCGCACATCAG | 2861 | 0.3468290778783419 | No Hit |
| GACTACCCGGGTATCTAATCCTGTTTGATCCCCACGCTTTCGCACATCAG | 2860 | 0.3467078513568885 | No Hit |
| GACTACTCGGGTATCTAATCCTGTTTGCTCCCCATGCTTTCGTACCTCAG | 2852 | 0.3457380391852608 | No Hit |
| GACTACTGGGGTATCTAATCCCATTTGCTCCCCTAGCTTTCGTCTCTCAG | 2814 | 0.3411314313700294 | No Hit |
| GACTACCAGGGTATCTAATCCTGTTTGCTCCCCATGCTTTCGTACCTCAG | 2743 | 0.3325243483468339 | No Hit |
| GACTACAAGGGTATCTAATCCTGTTTGCTCCCCATGCTTTCGTACCTCAG | 2738 | 0.33191821573956665 | No Hit |
| GACTACCGGGGTATCTAATCCTGTTTGCTCCCCACGCTTTCGTGCCTCAG | 2734 | 0.3314333096537528 | No Hit |
| GACTACTCGGGTATCTAATCCTGTTTGCTCCCCACGCTTTCGTGCCTCAG | 2669 | 0.32355358575927806 | No Hit |
| GACTACAAGGGTATCTAATCCTGTTTGCTCCCCACGCTTTCGTGCCTCAG | 2540 | 0.30791536449178203 | No Hit |
| GACTACCCGGGTATCTAATCCTGTTTGCTCCCCATGCTTTCGTACCTCAG | 2530 | 0.3067030992772475 | No Hit |
| GACTACTGGGGTATCTAATCCTGTTCGCTACCCATGCTTTCGCTCCTCAG | 2503 | 0.3034299831980041 | No Hit |
| GACTACTGGGGTATCTAATCCTGTTTGCTCCCCACGCTGTCGCGCCTCAG | 2477 | 0.30027809364021424 | No Hit |
| GACTACCAGGGTATCTAATCCTGTTTGCTCCCCACGCTTTCGTGCCTCAG | 2475 | 0.3000356405973073 | No Hit |
| GACTACAGGGGTATCTAATCCCATTTGCTCCCCTAGCTTTCGTCTCTCAG | 2439 | 0.29567148582498287 | No Hit |
| GACTACACGGGTATCTAATCCTGTTTGCTCCCCATGCTTTCGTACCTCAG | 2432 | 0.29482290017480867 | No Hit |
| GACTACTAGGGTATCTAATCCCATTTGCTCCCCTAGCTTTCGTCTCTCAG | 2331 | 0.2825790215080094 | No Hit |
| GACTACACGGGTATCTAATCCTGTTTGCTCCCCACGCTTTCGTGCCTCAG | 2310 | 0.28003326455748684 | No Hit |
| GACTACCCGGGTATCTAATCCTGTTTGCTCCCCACGCTTTCGTGCCTCAG | 2290 | 0.2776087341284177 | No Hit |
| GACTACCGGGGTATCTAATCCCATTTGCTCCCCTAGCTTTCGTCTCTCAG | 2282 | 0.27663892195679 | No Hit |
| GACTACAGGGGTATCTAATCCTGTTCGCTACCCATGCTTTCGCTCCTCAG | 2251 | 0.27288089979173286 | No Hit |
| GACTACCAGGGTATCTAATCCCATTTGCTCCCCTAGCTTTCGTCTCTCAG | 2137 | 0.25906107634603864 | No Hit |
| GACTACTAGGGTATCTAATCCTGTTTGCTCCCCACGCTGTCGCGCCTCAG | 2126 | 0.25772758461005063 | No Hit |
| GACTACCGGGGTATCTAATCCTGTTTGCTCCCCACGCTGTCGCGCCTCAG | 2124 | 0.25748513156714375 | No Hit |
| GACTACAAGGGTATCTAATCCCATTTGCTCCCCTAGCTTTCGTCTCTCAG | 2104 | 0.25506060113807455 | No Hit |
| GACTACCGGGGTATCTAATCCTGTTCGCTACCCATGCTTTCGCTCCTCAG | 2083 | 0.252514844187552 | No Hit |
| GACTACTAGGGTATCTAATCCTGTTCGCTACCCATGCTTTCGCTCCTCAG | 2066 | 0.25045399332284324 | No Hit |
| GACTACTCGGGTATCTAATCCCATTTGCTCCCCTAGCTTTCGTCTCTCAG | 2042 | 0.24754455680796023 | No Hit |
| GACTACTCGGGTATCTAATCCTGTTCGCTACCCATGCTTTCGCTCCTCAG | 2041 | 0.2474233302865068 | No Hit |
| GACTACTGGGGTATCTAATCCTGTTCGCTCCCCATGCTTTCGCTTCTCAG | 2022 | 0.24512002637889105 | No Hit |
| GACTACAGGGGTATCTAATCCTGTTTGCTCCCCACGCTGTCGCGCCTCAG | 2015 | 0.24427144072871687 | No Hit |
| GACTACTCGGGTATCTAATCCTGTTTGCTCCCCACGCTGTCGCGCCTCAG | 1966 | 0.23833134117749746 | No Hit |
| GACTACAAGGGTATCTAATCCTGTTCGCTACCCATGCTTTCGCTCCTCAG | 1941 | 0.235300678141161 | No Hit |
| GACTACCCGGGTATCTAATCCCATTTGCTCCCCTAGCTTTCGTCTCTCAG | 1891 | 0.22923935206848814 | No Hit |
| GACTACTGGGGTATCTAATCCTGTTTGCTCCCCACGCTTTCGAGCCTCAG | 1886 | 0.22863321946122087 | No Hit |
| GACTACCAGGGTATCTAATCCTGTTCGCTACCCATGCTTTCGCTCCTCAG | 1866 | 0.2262086890321517 | No Hit |
| GACTACAAGGGTATCTAATCCTGTTTGCTCCCCACGCTGTCGCGCCTCAG | 1808 | 0.21917755078785117 | No Hit |
| GACTACCAGGGTATCTAATCCTGTTTGCTCCCCACGCTGTCGCGCCTCAG | 1807 | 0.2190563242663977 | No Hit |
| GACTACCCGGGTATCTAATCCTGTTCGCTACCCATGCTTTCGCTCCTCAG | 1797 | 0.21784405905186313 | No Hit |
| GACTACAGGGGTATCTAATCCTGTTCGCTCCCCATGCTTTCGCTTCTCAG | 1796 | 0.2177228325304097 | No Hit |
| GACTACTGGGGTATCTAATCCTGTTCGCTCCCCACACTTTCGCTCCTCAG | 1788 | 0.21675302035878202 | No Hit |
| GACTACACGGGTATCTAATCCCATTTGCTCCCCTAGCTTTCGTCTCTCAG | 1757 | 0.21299499819372483 | No Hit |
| GACTACCGGGGTATCTAATCCTGTTCGCTCCCCATGCTTTCGCTTCTCAG | 1701 | 0.2062063129923312 | No Hit |
| GACTACAGGGGTATCTAATCCTGTTTGCTCCCCACGCTTTCGAGCCTCAG | 1697 | 0.20572140690651736 | No Hit |
| GACTACTAGGGTATCTAATCCTGTTCGCTCCCCATGCTTTCGCTTCTCAG | 1692 | 0.20511527429925008 | No Hit |
| GACTACTAGGGTATCTAATCCTGTTTGCTCCCCACGCTTTCGAGCCTCAG | 1650 | 0.20002376039820485 | No Hit |
| GACTACACGGGTATCTAATCCTGTTCGCTACCCATGCTTTCGCTCCTCAG | 1636 | 0.1983265890978565 | No Hit |
| GACTACACGGGTATCTAATCCTGTTTGCTCCCCACGCTGTCGCGCCTCAG | 1619 | 0.1962657382331477 | No Hit |
| GACTACCCGGGTATCTAATCCTGTTTGCTCCCCACGCTGTCGCGCCTCAG | 1615 | 0.19578083214733386 | No Hit |
| GACTACCGGGGTATCTAATCCTGTTTGCTCCCCACGCTTTCGAGCCTCAG | 1578 | 0.19129545085355593 | No Hit |
| GACTACTCGGGTATCTAATCCTGTTCGCTCCCCATGCTTTCGCTTCTCAG | 1555 | 0.1885072408601264 | No Hit |
| GACTACAAGGGTATCTAATCCTGTTCGCTCCCCATGCTTTCGCTTCTCAG | 1519 | 0.18414308608780194 | No Hit |
| GACTACAGGGGTATCTAATCCTGTTCGCTCCCCACACTTTCGCTCCTCAG | 1513 | 0.1834157269590812 | No Hit |
| GACTACTGGGGTATCTAATCCTGTTTGCTCCCCACGCTTTCGCACCTGAG | 1504 | 0.18232468826600007 | No Hit |
| GACTACCAGGGTATCTAATCCTGTTCGCTCCCCATGCTTTCGCTTCTCAG | 1482 | 0.17965770479402401 | No Hit |
| GACTACTAGGGTATCTAATCCTGTTCGCTCCCCACACTTTCGCTCCTCAG | 1481 | 0.17953647827257058 | No Hit |
| GACTACCGGGGTATCTAATCCTGTTCGCTCCCCACACTTTCGCTCCTCAG | 1468 | 0.1779605334936756 | No Hit |
| GACTACCAGGGTATCTAATCCTGTTTGCTCCCCACGCTTTCGAGCCTCAG | 1429 | 0.17323269915699077 | No Hit |
| GACTACAGGGGTATCTAATCCTGTTTGCTCCCCACGCTTTCGCACCTGAG | 1414 | 0.1714143013351889 | No Hit |
| GACTACCCGGGTATCTAATCCTGTTCGCTCCCCATGCTTTCGCTTCTCAG | 1411 | 0.17105062177082853 | No Hit |
| GACTACAAGGGTATCTAATCCTGTTTGCTCCCCACGCTTTCGAGCCTCAG | 1406 | 0.17044448916356125 | No Hit |
| GACTACTCGGGTATCTAATCCTGTTTGCTCCCCACGCTTTCGAGCCTCAG | 1382 | 0.16753505264867827 | No Hit |
| GACTACACGGGTATCTAATCCTGTTCGCTCCCCATGCTTTCGCTTCTCAG | 1365 | 0.1654742017839695 | No Hit |
| GACTACAAGGGTATCTAATCCTGTTCGCTCCCCACACTTTCGCTCCTCAG | 1355 | 0.1642619365694349 | No Hit |
| GACTACCAGGGTATCTAATCCTGTTCGCTCCCCACACTTTCGCTCCTCAG | 1350 | 0.16365580396216764 | No Hit |
| GACTACTCGGGTATCTAATCCTGTTCGCTCCCCACACTTTCGCTCCTCAG | 1350 | 0.16365580396216764 | No Hit |
| GACTACTAGGGTATCTAATCCTGTTTGCTCCCCACGCTTTCGCACCTGAG | 1302 | 0.15783693093240167 | No Hit |
| GACTACCCGGGTATCTAATCCTGTTTGCTCCCCACGCTTTCGAGCCTCAG | 1296 | 0.15710957180368093 | No Hit |
| GACTACTCGGGTATCTAATCCTGTTTGCTCCCCACGCTTTCGCACCTGAG | 1291 | 0.15650343919641363 | No Hit |
| GACTACCGGGGTATCTAATCCTGTTTGCTCCCCACGCTTTCGCACCTGAG | 1265 | 0.15335154963862374 | No Hit |
| GACTACACGGGTATCTAATCCTGTTTGCTCCCCACGCTTTCGAGCCTCAG | 1253 | 0.15189683138118226 | No Hit |
| GACTACCCGGGTATCTAATCCTGTTCGCTCCCCACACTTTCGCTCCTCAG | 1218 | 0.14765390313031124 | No Hit |
| GACTACACGGGTATCTAATCCTGTTCGCTCCCCACACTTTCGCTCCTCAG | 1207 | 0.1463204113943232 | No Hit |
| GACTACCAGGGTATCTAATCCTGTTTGCTCCCCACGCTTTCGCACCTGAG | 1181 | 0.1431685218365333 | No Hit |
| GACTACAAGGGTATCTAATCCTGTTTGCTCCCCACGCTTTCGCACCTGAG | 1171 | 0.14195625662199873 | No Hit |
| GACTACCCGGGTATCTAATCCTGTTTGCTCCCCACGCTTTCGCACCTGAG | 1039 | 0.12595435579014236 | No Hit |
| GACTACTGGGGTATCTAATCCTGTTTGCTCCCCATGCTTTCGCACCTCAG | 1038 | 0.1258331292686889 | No Hit |
| GACTACACGGGTATCTAATCCTGTTTGCTCCCCACGCTTTCGCACCTGAG | 995 | 0.12062038884619022 | No Hit |
| GACTACTGGGGTATCTAATCCTGTTCGCTCCCCACGCTTTCGTGCCTCAG | 965 | 0.11698359320258649 | No Hit |
| GACTACCGGGGTATCTAATCCTGTTTGCTCCCCATGCTTTCGCACCTCAG | 884 | 0.10716424496485644 | No Hit |
| GACTACTAGGGTATCTAATCCTGTTCGCTCCCCACGCTTTCGTGCCTCAG | 858 | 0.10401235540706655 | No Hit |
| GACTACTAGGGTATCTAATCCTGTTTGCTCCCCATGCTTTCGCACCTCAG | 852 | 0.1032849962783458 | No Hit |
| GACTACTGGGGTATCTAATCCTGTTTGATCCCCACGCTTTCGTGCCTCAG | 841 | 0.10195150454235775 | No Hit |
| GACTACAGGGGTATCTAATCCTGTTTGCTCCCCATGCTTTCGCACCTCAG | 839 | 0.10170905149945084 | No Hit |
| GACTACAGGGGTATCTAATCCTGTTCGCTCCCCACGCTTTCGTGCCTCAG | 837 | 0.10146659845654393 | No Hit |
| GACTACCGGGGTATCTAATCCTGTTCGCTCCCCACGCTTTCGTGCCTCAG | 834 | 0.10110291889218355 | No Hit |
| GACTACTGGGGTATCTAATCCGGTTCGCTCCCCACACTTTCGCGCCTCAG | 827 | 0.10025433324200934 | No Hit |

## Adapter Content

## Kmer Content

| Sequence | Count | PValue | Obs/Exp Max | Max Obs/Exp Position |
| --- | --- | --- | --- | --- |
| GTTAGAA | 5 | 2.1176899E-4 | 6215.2983 | 295 |
| GTGAGGG | 5 | 2.1176899E-4 | 6215.2983 | 295 |
| GTTGGAA | 5 | 2.1176899E-4 | 6215.2983 | 295 |
| GATAGCG | 20 | 0.0 | 6215.2983 | 295 |
| GTTAGCG | 690 | 0.0 | 5900.03 | 295 |
| GTTAGGG | 60 | 0.0 | 5697.3574 | 295 |
| TTAGCCG | 1825 | 0.0 | 5193.606 | 295 |
| GTTGGCG | 145 | 0.0 | 4715.054 | 295 |
| TTAGACG | 55 | 0.0 | 4520.2173 | 295 |
| TTAGGCG | 75 | 0.0 | 4143.532 | 295 |
| GTTAGCA | 95 | 0.0 | 3598.331 | 295 |
| TTTGCCG | 130 | 0.0 | 3346.6992 | 295 |
| GGGCTCA | 10 | 8.469853E-4 | 3107.6492 | 295 |
| GTTAGGA | 10 | 8.469853E-4 | 3107.6492 | 295 |
| GTAGGCG | 20 | 7.2473995E-7 | 3107.6492 | 295 |
| ATCTTGT | 10 | 8.469853E-4 | 3107.6492 | 295 |
| ATCCATA | 10 | 8.469853E-4 | 3107.6492 | 295 |
| GTAAGAG | 10 | 8.469853E-4 | 3107.6492 | 295 |
| GGTAGGG | 10 | 8.469853E-4 | 3107.6492 | 295 |
| GTTTGCG | 65 | 0.0 | 2868.5994 | 295 |

Produced by FastQC (version 0.11.7)
