# Supplementary material for: Assessment of airborne bacteria from a public health institution in Mexico City
Source: PLOS Glob Public Health. 2024 Nov 7;4(11):e0003672. doi: 10.1371/journal.pgph.0003672 (PMC11542838; doi:10.1371/journal.pgph.0003672)
Supplement: S1 Text — (ZIP) [file pgph.0003672.s001.zip › Hospital_16S_QC/21022023_CP2D1_16S_S37_L001_R1_001_fastqc.html]

21022023\_CP2D1\_16S\_S37\_L001\_R1\_001.fastq.gz FastQC Report 

FastQC Report

Wed 15 Mar 2023  
21022023\_CP2D1\_16S\_S37\_L001\_R1\_001.fastq.gz

## Summary

- Basic Statistics
- Per base sequence quality
- Per tile sequence quality
- Per sequence quality scores
- Per base sequence content
- Per sequence GC content
- Per base N content
- Sequence Length Distribution
- Sequence Duplication Levels
- Overrepresented sequences
- Adapter Content
- Kmer Content

## Basic Statistics

| Measure | Value |
| --- | --- |
| Filename | 21022023\_CP2D1\_16S\_S37\_L001\_R1\_001.fastq.gz |
| File type | Conventional base calls |
| Encoding | Sanger / Illumina 1.9 |
| Total Sequences | 142277 |
| Sequences flagged as poor quality | 0 |
| Sequence length | 40-301 |
| %GC | 55 |

## Per base sequence quality

## Per tile sequence quality

## Per sequence quality scores

## Per base sequence content

## Per sequence GC content

## Per base N content

## Sequence Length Distribution

## Sequence Duplication Levels

## Overrepresented sequences

| Sequence | Count | Percentage | Possible Source |
| --- | --- | --- | --- |
| CCTACGGGTGGCTGCAGTGGGGAATATTGCACAATGGGCGCAAGCCTGAT | 3167 | 2.225939540473864 | No Hit |
| CCTACGGGAGGCTGCAGTGGGGAATATTGCACAATGGGCGCAAGCCTGAT | 3063 | 2.152842694181069 | No Hit |
| CCTACGGGGGGCTGCAGTGGGGAATATTGCACAATGGGCGCAAGCCTGAT | 2807 | 1.9729119956141892 | No Hit |
| CCTACGGGTGGCTGCAGTGGGGAATATTGCACAATGGGCGAAAGCCTGAT | 2783 | 1.9560434926235442 | No Hit |
| CCTACGGGAGGCTGCAGTGGGGAATATTGCACAATGGGCGAAAGCCTGAT | 2461 | 1.7297244108323901 | No Hit |
| CCTACGGGCGGCTGCAGTGGGGAATATTGCACAATGGGCGCAAGCCTGAT | 2330 | 1.63765049867512 | No Hit |
| CCTACGGGGGGCTGCAGTGGGGAATATTGCACAATGGGCGAAAGCCTGAT | 2239 | 1.573690758168924 | No Hit |
| CCTACGGGTGGCTGCAGTGGGGAATATTGGACAATGGGCGAAAGCCTGAT | 2160 | 1.518165269158051 | No Hit |
| CCTACGGGTGGCTGCAGTGGGGAATATTGGACAATGGGCGCAAGCCTGAT | 1963 | 1.3797029737765063 | No Hit |
| CCTACGGGAGGCTGCAGTGGGGAATATTGGACAATGGGCGAAAGCCTGAT | 1954 | 1.3733772851550146 | No Hit |
| CCTACGGGGGGCTGCAGTGGGGAATATTGGACAATGGGCGAAAGCCTGAT | 1924 | 1.3522916564167082 | No Hit |
| CCTACGGGCGGCTGCAGTGGGGAATATTGCACAATGGGCGAAAGCCTGAT | 1885 | 1.3248803390569102 | No Hit |
| CCTACGGGAGGCAGCAGTGGGGAATATTGCACAATGGGCGCAAGCCTGAT | 1863 | 1.3094175446488188 | No Hit |
| CCTACGGGGGGCAGCAGTGGGGAATATTGCACAATGGGCGCAAGCCTGAT | 1860 | 1.3073089817749883 | No Hit |
| CCTACGGGAGGCTGCAGTGGGGAATATTGGACAATGGGCGCAAGCCTGAT | 1814 | 1.274977684376252 | No Hit |
| CCTACGGGTGGCAGCAGTGGGGAATATTGCACAATGGGCGCAAGCCTGAT | 1745 | 1.2264807382781475 | No Hit |
| CCTACGGGGGGCTGCAGTGGGGAATATTGGACAATGGGCGCAAGCCTGAT | 1734 | 1.218749341074102 | No Hit |
| CCTACGGGTGGCTGCAGTGGGGAATCTTAGACAATGGGGGCAACCCTGAT | 1639 | 1.1519781834027987 | No Hit |
| CCTACGGGAGGCTGCAGTGGGGAATCTTAGACAATGGGGGCAACCCTGAT | 1585 | 1.1140240516738473 | No Hit |
| CCTACGGGGGGCTGCAGTGGGGAATCTTAGACAATGGGGGCAACCCTGAT | 1577 | 1.1084012173436326 | No Hit |
| CCTACGGGCGGCTGCAGTGGGGAATATTGGACAATGGGCGAAAGCCTGAT | 1557 | 1.0943441315180948 | No Hit |
| CCTACGGGTGGCTGCAGTGGGGAATATTGCACAATGGGCGGAAGCCTGAT | 1547 | 1.0873155886053263 | No Hit |
| CCTACGGGGGGCAGCAGTGGGGAATATTGCACAATGGGCGAAAGCCTGAT | 1528 | 1.0739613570710655 | No Hit |
| CCTACGGGAGGCAGCAGTGGGGAATATTGCACAATGGGCGAAAGCCTGAT | 1518 | 1.0669328141582968 | No Hit |
| CCTACGGGCGGCTGCAGTGGGGAATATTGGACAATGGGCGCAAGCCTGAT | 1464 | 1.0289786824293454 | No Hit |
| CCTACGGGGGGCTGCAGTGGGGAATATTGCACAATGGGCGGAAGCCTGAT | 1442 | 1.0135158880212543 | No Hit |
| CCTACGGGAGGCAGCAGTGGGGAATATTGGACAATGGGCGAAAGCCTGAT | 1398 | 0.9825902992050719 | No Hit |
| CCTACGGGCGGCAGCAGTGGGGAATATTGCACAATGGGCGCAAGCCTGAT | 1379 | 0.9692360676708112 | No Hit |
| CCTACGGGAGGCTGCAGTGGGGAATATTGCACAATGGGCGGAAGCCTGAT | 1361 | 0.9565846904278275 | No Hit |
| CCTACGGGGGGCAGCAGTGGGGAATATTGGACAATGGGCGAAAGCCTGAT | 1348 | 0.947447584641228 | No Hit |
| CCTACGGGCGGCTGCAGTGGGGAATCTTAGACAATGGGGGCAACCCTGAT | 1333 | 0.936904770272075 | No Hit |
| CCTACGGGTGGCAGCAGTGGGGAATATTGCACAATGGGCGAAAGCCTGAT | 1294 | 0.9094934529122767 | No Hit |
| CCTACGGGCGGCTGCAGTGGGGAATATTGCACAATGGGCGGAAGCCTGAT | 1223 | 0.8595907982316187 | No Hit |
| CCTACGGGTGGCAGCAGTGGGGAATATTGGACAATGGGCGAAAGCCTGAT | 1202 | 0.8448308581148043 | No Hit |
| CCTACGGGGGGCAGCAGTGGGGAATCTTAGACAATGGGGGCAACCCTGAT | 1192 | 0.8378023152020355 | No Hit |
| CCTACGGGAGGCAGCAGTGGGGAATATTGGACAATGGGCGCAAGCCTGAT | 1156 | 0.8124995607160679 | No Hit |
| CCTACGGGGGGCAGCAGTGGGGAATATTGGACAATGGGCGCAAGCCTGAT | 1147 | 0.806173872094576 | No Hit |
| CCTACGGGCGGCAGCAGTGGGGAATATTGCACAATGGGCGAAAGCCTGAT | 1092 | 0.767516886074348 | No Hit |
| CCTACGGGTGGCAGCAGTGGGGAATATTGGACAATGGGCGCAAGCCTGAT | 1065 | 0.7485398202098723 | No Hit |
| CCTACGGGGGGCAGCAGTGGGGAATATTGCACAATGGGCGGAAGCCTGAT | 1020 | 0.7169113771024128 | No Hit |
| CCTACGGGCGGCAGCAGTGGGGAATATTGGACAATGGGCGAAAGCCTGAT | 972 | 0.6831743711211229 | No Hit |
| CCTACGGGAGGCAGCAGTGGGGAATCTTAGACAATGGGGGCAACCCTGAT | 948 | 0.6663058681304779 | No Hit |
| CCTACGGGTGGCAGCAGTGGGGAATCTTAGACAATGGGGGCAACCCTGAT | 917 | 0.6445173851008947 | No Hit |
| CCTACGGGAGGCAGCAGTGGGGAATATTGCACAATGGGCGGAAGCCTGAT | 907 | 0.637488842188126 | No Hit |
| CCTACGGGCGGCAGCAGTGGGGAATCTTAGACAATGGGGGCAACCCTGAT | 868 | 0.6100775248283279 | No Hit |
| CCTACGGGTGGCAGCAGTGGGGAATATTGCACAATGGGCGGAAGCCTGAT | 836 | 0.5875861875074678 | No Hit |
| CCTACGGGCGGCAGCAGTGGGGAATATTGGACAATGGGCGCAAGCCTGAT | 813 | 0.5714205388080997 | No Hit |
| CCTACGGGGGGCAGCAGTAGGGAATCTTCCGCAATGGGCGAAAGCCTGAC | 800 | 0.5622834330215003 | No Hit |
| CCTACGGGAGGCTGCAGTAGGGAATCTTCCGCAATGGGCGAAAGCCTGAC | 795 | 0.5587691615651159 | No Hit |
| CCTACGGGCGGCAGCAGTGGGGAATATTGCACAATGGGCGGAAGCCTGAT | 779 | 0.5475234929046859 | No Hit |
| CCTACGGGTGGCTGCAGTAGGGAATCTTCCGCAATGGGCGAAAGCCTGAC | 759 | 0.5334664070791484 | No Hit |
| CCTACGGGAGGCAGCAGTAGGGAATCTTCCGCAATGGGCGAAAGCCTGAC | 755 | 0.5306549899140409 | No Hit |
| CCTACGGGGGGCTGCAGTAGGGAATCTTCCGCAATGGGCGAAAGCCTGAC | 751 | 0.5278435727489335 | No Hit |
| CCTACGGGTGGCAGCAGTAGGGAATCTTCCGCAATGGGCGAAAGCCTGAC | 693 | 0.48707802385487464 | No Hit |
| CCTACGGGTGGCTGCAGTGGGGAATATTGGACAATGGGGGCAACCCTGAT | 663 | 0.4659923951165684 | No Hit |
| CCTACGGGAGGCTGCAGTGGGGAATATTGGACAATGGGGGCAACCCTGAT | 655 | 0.4603695607863533 | No Hit |
| CCTACGGGAGGCTGCAGTGGGGAATATTGCACAATGGGGGAAACCCTGAT | 644 | 0.4526381635823078 | No Hit |
| CCTACGGGTGGCTGCAGTGGGGAATTTTGGACAATGGGCGCAAGCCTGAT | 641 | 0.4505296007084771 | No Hit |
| CCTACGGGTGGCTGCAGTGGGGAATATTGCACAATGGGGGAAACCCTGAT | 638 | 0.4484210378346465 | No Hit |
| CCTACGGGAGGCTGCAGTGGGGAATTTTGGACAATGGGCGCAAGCCTGAT | 630 | 0.4427982035044315 | No Hit |
| CCTACGGGGGGCTGCAGTGGGGAATATTGCACAATGGGGGAAACCCTGAT | 624 | 0.43858107775677024 | No Hit |
| CCTACGGGCGGCTGCAGTAGGGAATCTTCCGCAATGGGCGAAAGCCTGAC | 583 | 0.40976405181441833 | No Hit |
| CCTACGGGCGGCTGCAGTGGGGAATTTTGGACAATGGGCGCAAGCCTGAT | 573 | 0.4027355089016496 | No Hit |
| CCTACGGGGGGCTGCAGTGGGGAATTTTGGACAATGGGCGCAAGCCTGAT | 568 | 0.3992212374452652 | No Hit |
| CCTACGGGCGGCAGCAGTAGGGAATCTTCCGCAATGGGCGAAAGCCTGAC | 552 | 0.3879755687848352 | No Hit |
| CCTACGGGGGGCTGCAGTGGGGAATATTGGACAATGGGGGCAACCCTGAT | 537 | 0.3774327544156821 | No Hit |
| CCTACGGGCGGCTGCAGTGGGGAATATTGCACAATGGGGGAAACCCTGAT | 494 | 0.34721001989077643 | No Hit |
| CCTACGGGCGGCTGCAGTGGGGAATATTGGACAATGGGGGCAACCCTGAT | 477 | 0.33526149693906954 | No Hit |
| CCTACGGGGGGCTGCAGTAGGGAATCTTCCGCAATGGACGAAAGTCTGAC | 448 | 0.3148787224920402 | No Hit |
| CCTACGGGGGGCAGCAGTAGGGAATCTTCCGCAATGGACGAAAGTCTGAC | 448 | 0.3148787224920402 | No Hit |
| CCTACGGGAGGCAGCAGTGGGGAATTTTGGACAATGGGCGCAAGCCTGAT | 441 | 0.30995874245310207 | No Hit |
| CCTACGGGTGGCTGCAGTAGGGAATCTTCCGCAATGGACGAAAGTCTGAC | 436 | 0.3064444709967177 | No Hit |
| CCTACGGGAGGCAGCAGTGGGGAATATTGCACAATGGGGGAAACCCTGAT | 417 | 0.29309023946245705 | No Hit |
| CCTACGGGAGGCTGCAGTAGGGAATCTTCCGCAATGGACGAAAGTCTGAC | 414 | 0.2909816765886264 | No Hit |
| CCTACGGGTGGCTGCAGTGGGGAATATTGCGCAATGGGCGGAAGCCTGAC | 410 | 0.2881702594235189 | No Hit |
| CCTACGGGGGGCAGCAGTGGGGAATTTTGGACAATGGGCGCAAGCCTGAT | 403 | 0.2832502793845808 | No Hit |
| CCTACGGGTGGCAGCAGTGGGGAATTTTGGACAATGGGCGCAAGCCTGAT | 399 | 0.2804388622194733 | No Hit |
| CCTACGGGGGGCAGCAGTGGGGAATATTGGACAATGGGGGCAACCCTGAT | 399 | 0.2804388622194733 | No Hit |
| CCTACGGGGGGCTGCAGTGGGGAATATTGCGCAATGGGCGGAAGCCTGAC | 391 | 0.27481602788925824 | No Hit |
| CCTACGGGAGGCAGCAGTGGGGAATATTGGACAATGGGGGCAACCCTGAT | 391 | 0.27481602788925824 | No Hit |
| CCTACGGGTGGCAGCAGTGGGGAATATTGGACAATGGGGGCAACCCTGAT | 379 | 0.2663817763939358 | No Hit |
| CCTACGGGCGGCTGCAGTAGGGAATCTTCCGCAATGGACGAAAGTCTGAC | 377 | 0.26497606781138205 | No Hit |
| CCTACGGGTGGCAGCAGTAGGGAATCTTCCGCAATGGACGAAAGTCTGAC | 372 | 0.26146179635499767 | No Hit |
| CCTACGGGAGGCAGCAGTAGGGAATCTTCCGCAATGGACGAAAGTCTGAC | 368 | 0.25865037918989014 | No Hit |
| CCTACGGGGGGCAGCAGTGGGGAATATTGCACAATGGGGGAAACCCTGAT | 351 | 0.24670185623818325 | No Hit |
| CCTACGGGAGGCTGCAGTGGGGAATATTGCGCAATGGGCGAAAGCCTGAC | 338 | 0.23756475045158387 | No Hit |
| CCTACGGGAGGCTGCAGTGGGGAATATTGCGCAATGGGCGGAAGCCTGAC | 327 | 0.22983335324753823 | No Hit |
| CCTACGGGCGGCAGCAGTGGGGAATATTGGACAATGGGGGCAACCCTGAT | 321 | 0.22561622749987703 | No Hit |
| CCTACGGGCGGCTGCAGTGGGGAATATTGCGCAATGGGCGGAAGCCTGAC | 320 | 0.2249133732086001 | No Hit |
| CCTACGGGTGGCTGCAGTGGGGAATATTGCGCAATGGGCGAAAGCCTGAC | 319 | 0.22421051891732324 | No Hit |
| CCTACGGGTGGCTGCAGTGGGGAATCTTGGACAATGGGGGCAACCCTGAT | 317 | 0.22280481033476948 | No Hit |
| CCTACGGGTGGCAGCAGTGGGGAATATTGCACAATGGGGGAAACCCTGAT | 310 | 0.21788483029583136 | No Hit |
| CCTACGGGCGGCAGCAGTAGGGAATCTTCCGCAATGGACGAAAGTCTGAC | 302 | 0.21226199596561637 | No Hit |
| CTTGGTCATTTAGAGGAAGTAAAAGTCGTAACAAGGTTTCCGTAGGTGAA | 300 | 0.21085628738306264 | No Hit |
| CCTACGGGGGGCTGCAGTGGGGAATATTGCGCAATGGGCGAAAGCCTGAC | 296 | 0.2080448702179551 | No Hit |
| CCTACGGGCGGCAGCAGTGGGGAATTTTGGACAATGGGCGCAAGCCTGAT | 295 | 0.20734201592667822 | No Hit |
| CCTACGGGCGGCTGCAGTGGGGAATATTGCGCAATGGGCGAAAGCCTGAC | 265 | 0.18625638718837198 | No Hit |
| CCTACGGGCGGCAGCAGTGGGGAATATTGCACAATGGGGGAAACCCTGAT | 260 | 0.1827421157319876 | No Hit |
| CCTACGGGAGGCTGCAGTGGGGAATATTGGACAATGGGGGGAACCCTGAT | 257 | 0.180633552858157 | No Hit |
| CCTACGGGAGGCTGCAGTGGGGAATCTTGGACAATGGGGGCAACCCTGAT | 256 | 0.1799306985668801 | No Hit |
| CCTACGGGGGGCTGCAGTGGGGAATCTTGGACAATGGGGGCAACCCTGAT | 252 | 0.1771192814017726 | No Hit |
| CCTACGGGGGGCTGCAGTGGGGAATATTGGACAATGGGGGGAACCCTGAT | 246 | 0.17290215565411135 | No Hit |
| CCTACGGGGGGCAGCAGTGGGGAATATTGCGCAATGGGCGGAAGCCTGAC | 243 | 0.17079359278028072 | No Hit |
| CCTACGGGTGGCTGCAGTGGGGAATATTGGACAATGGGCGGAAGCCTGAT | 242 | 0.17009073848900386 | No Hit |
| CCTACGGGGGGCTGCAGTGGGGAATTTTCCGCAATGGGCGAAAGCCTGAC | 240 | 0.1686850299064501 | No Hit |
| CCTACGGGAGGCAGCAGTGGGGAATATTGCGCAATGGGCGGAAGCCTGAC | 236 | 0.1658736127413426 | No Hit |
| CCTACGGGAGGCTGCAGTAGGGAATCTTCCACAATGGACGAAAGTCTGAT | 233 | 0.16376504986751197 | No Hit |
| CCTACGGGTGGCTGCAGTGGGGAATATTGGACAATGGGGGGAACCCTGAT | 227 | 0.1595479241198507 | No Hit |
| CCTACGGGAGGCTGCAGTGGGGAATTTTCCGCAATGGGCGAAAGCCTGAC | 222 | 0.15603365266346633 | No Hit |
| CCTACGGGCGGCTGCAGTGGGGAATCTTGGACAATGGGGGCAACCCTGAT | 220 | 0.15462794408091257 | No Hit |
| CCTACGGGGGGCTGCAGTAGGGAATCTTCCACAATGGACGAAAGTCTGAT | 219 | 0.1539250897896357 | No Hit |
| CCTACGGGTGGCTGCAGTGGGGAATCTTGCGCAATGGGCGAAAGCCTGAC | 218 | 0.15322223549835884 | No Hit |
| CCTACGGGTGGCTGCAGTAGGGAATCTTCCACAATGGACGAAAGTCTGAT | 217 | 0.15251938120708197 | No Hit |
| CCTACGGGAGGCTGCAGTAGGGAATCTTCCGCAATGGACGCAAGTCTGAC | 216 | 0.15181652691580508 | No Hit |
| CCTACGGGAGGCAGCAGTAGGGAATCTTCCACAATGGACGAAAGTCTGAT | 213 | 0.14970796404197445 | No Hit |
| CCTACGGGTGGCTGCAGTGGGGAATTTTCCGCAATGGGCGAAAGCCTGAC | 212 | 0.14900510975069758 | No Hit |
| CCTACGGGTGGCAGCAGTGGGGAATATTGCGCAATGGGCGGAAGCCTGAC | 211 | 0.1483022554594207 | No Hit |
| CCTACGGGCGGCTGCAGTGGGGAATATTGGACAATGGGGGGAACCCTGAT | 207 | 0.1454908382943132 | No Hit |
| CCTACGGGGGGCTGCAGTGGGGAATCTTGCGCAATGGGCGAAAGCCTGAC | 203 | 0.1426794211292057 | No Hit |
| CCTACGGGTGGCTGCAGTAGGGAATCTTCCGCAATGGACGCAAGTCTGAC | 202 | 0.14197656683792884 | No Hit |
| CCTACGGGTGGCAGCAGTAGGGAATCTTCCACAATGGACGAAAGTCTGAT | 202 | 0.14197656683792884 | No Hit |
| CCTACGGGGGGCAGCAGTGGGGAATATTGCGCAATGGGCGAAAGCCTGAC | 201 | 0.14127371254665197 | No Hit |
| CCTACGGGCGGCAGCAGTGGGGAATATTGCGCAATGGGCGGAAGCCTGAC | 195 | 0.1370565867989907 | No Hit |
| CCTACGGGGGGCAGCAGTAGGGAATCTTCCACAATGGACGAAAGTCTGAT | 194 | 0.13635373250771382 | No Hit |
| CCTACGGGCGGCAGCAGTGGGGAATATTGCGCAATGGGCGAAAGCCTGAC | 193 | 0.13565087821643695 | No Hit |
| CCTACGGGAGGCAGCAGTGGGGAATATTGCGCAATGGGCGAAAGCCTGAC | 190 | 0.13354231534260633 | No Hit |
| CCTACGGGAGGCTGCAGTGGGGAATCTTGCGCAATGGGCGAAAGCCTGAC | 188 | 0.13213660676005257 | No Hit |
| CCTACGGGAGGCAGCAGTAGGGAATCTTCCGCAATGGACGCAAGTCTGAC | 185 | 0.13002804388622197 | No Hit |
| CCTACGGGCGGCTGCAGTGGGGAATCTTGCGCAATGGGCGAAAGCCTGAC | 183 | 0.12862233530366818 | No Hit |
| CCTACGGGTGGCTGCAGTAGGGAATCTTCGGCAATGGGGGCAACCCTGAC | 178 | 0.12510806384728382 | No Hit |
| CCTACGGGGGGCAGCAGTGGGGAATCTTGGACAATGGGGGCAACCCTGAT | 176 | 0.12370235526473006 | No Hit |
| CCTACGGGTGGCAGCAGTGGGGAATATTGCGCAATGGGCGAAAGCCTGAC | 176 | 0.12370235526473006 | No Hit |
| CCTACGGGAGGCTGCAGTGGGGAATATTGGACAATGGGCGGAAGCCTGAT | 175 | 0.12299950097345319 | No Hit |
| CCTACGGGGGGCAGCAGTAGGGAATCTTCCGCAATGGACGCAAGTCTGAC | 172 | 0.12089093809962256 | No Hit |
| CCTACGGGGGGCTGCAGTAGGGAATCTTCCGCAATGGACGCAAGTCTGAC | 171 | 0.1201880838083457 | No Hit |
| CCTACGGGCGGCTGCAGTGGGGAATATTGGACAATGGGCGGAAGCCTGAT | 168 | 0.11807952093451507 | No Hit |
| CCTACGGGAGGCTGCAGTAGGGAATCTTCGGCAATGGGGGCAACCCTGAC | 165 | 0.11597095806068444 | No Hit |
| CCTACGGGGGGCTGCAGTGGGGAATATTGGACAATGGGCGGAAGCCTGAT | 165 | 0.11597095806068444 | No Hit |
| CCTACGGGTGGCAGCAGTAGGGAATCTTCCGCAATGGACGCAAGTCTGAC | 162 | 0.1138623951868538 | No Hit |
| CCTACGGGCGGCTGCAGTAGGGAATCTTCCACAATGGACGAAAGTCTGAT | 160 | 0.11245668660430005 | No Hit |
| CCTACGGGCGGCTGCAGTGGGGAATTTTCCGCAATGGGCGAAAGCCTGAC | 160 | 0.11245668660430005 | No Hit |
| CCTACGGGCGGCAGCAGTAGGGAATCTTCCACAATGGACGAAAGTCTGAT | 156 | 0.10964526943919256 | No Hit |
| CCTACGGGAGGCAGCAGTGGGGAATATTGGACAATGGGGGGAACCCTGAT | 155 | 0.10894241514791568 | No Hit |
| CCTACGGGAGGCAGCAGTGGGGAATCTTGGACAATGGGGGCAACCCTGAT | 150 | 0.10542814369153132 | No Hit |
| CCTACGGGTGGCTGCAGTGGGGAATATTGGACAATGGGGGAAACCCTGAT | 148 | 0.10402243510897755 | No Hit |
| CCTACGGGGGGCAGCAGTAGGGAATCTTCGGCAATGGGGGCAACCCTGAC | 145 | 0.10191387223514693 | No Hit |
| CCTACGGGGGGCTGCAGTAGGGAATCTTCGGCAATGGGGGCAACCCTGAC | 145 | 0.10191387223514693 | No Hit |
| CCTACGGGTGGCAGCAGTGGGGAATATTGGACAATGGGGGGAACCCTGAT | 143 | 0.10050816365259319 | No Hit |

## Adapter Content

## Kmer Content

| Sequence | Count | PValue | Obs/Exp Max | Max Obs/Exp Position |
| --- | --- | --- | --- | --- |
| AAGTAGA | 20 | 4.2771717E-8 | 317.42258 | 295 |
| ATTCATA | 10 | 6.7904865E-4 | 317.42258 | 295 |
| ATTGGTG | 10 | 6.7904865E-4 | 317.42258 | 295 |
| ATTTCAT | 40 | 0.0 | 317.42258 | 295 |
| ATTTCAG | 20 | 4.2771717E-8 | 317.42258 | 295 |
| ATGTGAT | 240 | 0.0 | 317.42255 | 295 |
| AGTGGAT | 25 | 3.4015102E-10 | 317.42255 | 295 |
| GCTTGTG | 50 | 0.0 | 317.42255 | 295 |
| TATGATA | 15 | 5.393551E-6 | 317.42255 | 295 |
| GAGAGAG | 1285 | 0.0 | 316.18744 | 295 |
| AGTGTTG | 255 | 0.0 | 311.1986 | 295 |
| TGGAGAG | 150 | 0.0 | 306.8418 | 295 |
| GGGAGAG | 485 | 0.0 | 297.78815 | 295 |
| AGGTATG | 75 | 0.0 | 296.26105 | 295 |
| ATCCAAA | 375 | 0.0 | 296.26105 | 295 |
| TATTTAG | 25 | 5.4023985E-10 | 293.88086 | 7 |
| GGGAGAC | 10 | 8.554069E-4 | 293.88086 | 6 |
| CTTGGTC | 50 | 0.0 | 293.88086 | 1 |
| TAGTTAT | 10 | 8.554069E-4 | 293.88086 | 3 |
| CTCGGTC | 10 | 8.554069E-4 | 293.88086 | 1 |

Produced by FastQC (version 0.11.7)
