# Supplementary material for: Assessment of airborne bacteria from a public health institution in Mexico City
Source: PLOS Glob Public Health. 2024 Nov 7;4(11):e0003672. doi: 10.1371/journal.pgph.0003672 (PMC11542838; doi:10.1371/journal.pgph.0003672)
Supplement: S1 Text — (ZIP) [file pgph.0003672.s001.zip › Hospital_16S_QC/21022023_CP2D1_16S_S37_L001_R2_001_fastqc.html]

21022023\_CP2D1\_16S\_S37\_L001\_R2\_001.fastq.gz FastQC Report 

FastQC Report

Wed 15 Mar 2023  
21022023\_CP2D1\_16S\_S37\_L001\_R2\_001.fastq.gz

## Summary

- Basic Statistics
- Per base sequence quality
- Per tile sequence quality
- Per sequence quality scores
- Per base sequence content
- Per sequence GC content
- Per base N content
- Sequence Length Distribution
- Sequence Duplication Levels
- Overrepresented sequences
- Adapter Content
- Kmer Content

## Basic Statistics

| Measure | Value |
| --- | --- |
| Filename | 21022023\_CP2D1\_16S\_S37\_L001\_R2\_001.fastq.gz |
| File type | Conventional base calls |
| Encoding | Sanger / Illumina 1.9 |
| Total Sequences | 142277 |
| Sequences flagged as poor quality | 0 |
| Sequence length | 80-301 |
| %GC | 54 |

## Per base sequence quality

## Per tile sequence quality

## Per sequence quality scores

## Per base sequence content

## Per sequence GC content

## Per base N content

## Sequence Length Distribution

## Sequence Duplication Levels

## Overrepresented sequences

| Sequence | Count | Percentage | Possible Source |
| --- | --- | --- | --- |
| GACTACTGGGGTATCTAATCCTGTTCGCTCCCCATGCTTTCGCTCCTCAG | 3658 | 2.5710409974908104 | No Hit |
| GACTACAGGGGTATCTAATCCTGTTCGCTCCCCATGCTTTCGCTCCTCAG | 3205 | 2.2526480035423857 | No Hit |
| GACTACTAGGGTATCTAATCCTGTTCGCTCCCCATGCTTTCGCTCCTCAG | 3062 | 2.1521398398897924 | No Hit |
| GACTACCGGGGTATCTAATCCTGTTCGCTCCCCATGCTTTCGCTCCTCAG | 3051 | 2.144408442685747 | No Hit |
| GACTACTGGGGTATCTAATCCTGTTTGCTCCCCACGCTTTCGCACCTCAG | 2945 | 2.069905887810398 | No Hit |
| GACTACTCGGGTATCTAATCCTGTTCGCTCCCCATGCTTTCGCTCCTCAG | 2825 | 1.9855633728571729 | No Hit |
| GACTACCAGGGTATCTAATCCTGTTCGCTCCCCATGCTTTCGCTCCTCAG | 2787 | 1.9588549097886518 | No Hit |
| GACTACAAGGGTATCTAATCCTGTTCGCTCCCCATGCTTTCGCTCCTCAG | 2763 | 1.9419864067980068 | No Hit |
| GACTACAGGGGTATCTAATCCTGTTTGCTCCCCACGCTTTCGCACCTCAG | 2586 | 1.817581197242 | No Hit |
| GACTACTAGGGTATCTAATCCTGTTTGCTCCCCACGCTTTCGCACCTCAG | 2523 | 1.7733013768915566 | No Hit |
| GACTACCCGGGTATCTAATCCTGTTCGCTCCCCATGCTTTCGCTCCTCAG | 2454 | 1.7248044307934522 | No Hit |
| GACTACCGGGGTATCTAATCCTGTTTGCTCCCCACGCTTTCGCACCTCAG | 2418 | 1.6995016763074848 | No Hit |
| GACTACTCGGGTATCTAATCCTGTTTGCTCCCCACGCTTTCGCACCTCAG | 2416 | 1.698095967724931 | No Hit |
| GACTACACGGGTATCTAATCCTGTTCGCTCCCCATGCTTTCGCTCCTCAG | 2389 | 1.6791189018604553 | No Hit |
| GACTACAAGGGTATCTAATCCTGTTTGCTCCCCACGCTTTCGCACCTCAG | 2258 | 1.5870449897031846 | No Hit |
| GACTACCAGGGTATCTAATCCTGTTTGCTCCCCACGCTTTCGCACCTCAG | 2254 | 1.5842335725380772 | No Hit |
| GACTACCCGGGTATCTAATCCTGTTTGCTCCCCACGCTTTCGCACCTCAG | 2086 | 1.466154051603562 | No Hit |
| GACTACACGGGTATCTAATCCTGTTTGCTCCCCACGCTTTCGCACCTCAG | 2037 | 1.4317141913309952 | No Hit |
| GACTACTGGGGTATCTAATCCTGTTTGCTCCCCATGCTTTCGTACCTCAG | 1154 | 0.8110938521335141 | No Hit |
| GACTACTGGGGTATCTAATCCTGTTTGCTCCCCACGCTTTCGCGCCTCAG | 1057 | 0.7429169858796573 | No Hit |
| GACTACTAGGGTATCTAATCCTGTTTGCTCCCCATGCTTTCGTACCTCAG | 990 | 0.6958257483641066 | No Hit |
| GACTACTCGGGTATCTAATCCTGTTTGCTCCCCATGCTTTCGTACCTCAG | 972 | 0.6831743711211229 | No Hit |
| GACTACAGGGGTATCTAATCCTGTTTGCTCCCCATGCTTTCGTACCTCAG | 969 | 0.6810658082472922 | No Hit |
| GACTACCGGGGTATCTAATCCTGTTTGCTCCCCATGCTTTCGTACCTCAG | 963 | 0.6768486824996309 | No Hit |
| GACTACAGGGGTATCTAATCCTGTTTGCTCCCCACGCTTTCGCGCCTCAG | 925 | 0.6501402194311098 | No Hit |
| GACTACTAGGGTATCTAATCCTGTTTGCTCCCCACGCTTTCGCGCCTCAG | 912 | 0.6410031136445103 | No Hit |
| GACTACTGGGGTATCTAATCCTGTTCGCTCCCCACGCTTTCGCTCCTCAG | 863 | 0.6065632533719435 | No Hit |
| GACTACCGGGGTATCTAATCCTGTTTGCTCCCCACGCTTTCGCGCCTCAG | 838 | 0.5889918960900216 | No Hit |
| GACTACCAGGGTATCTAATCCTGTTTGCTCCCCATGCTTTCGTACCTCAG | 821 | 0.5770433731383147 | No Hit |
| GACTACTGGGGTATCTAATCCTGTTTGATCCCCACGCTTTCGCACATCAG | 820 | 0.5763405188470379 | No Hit |
| GACTACTCGGGTATCTAATCCTGTTTGCTCCCCACGCTTTCGCGCCTCAG | 792 | 0.5566605986912854 | No Hit |
| GACTACCAGGGTATCTAATCCTGTTTGCTCCCCACGCTTTCGCGCCTCAG | 791 | 0.5559577444000084 | No Hit |
| GACTACAAGGGTATCTAATCCTGTTTGCTCCCCATGCTTTCGTACCTCAG | 770 | 0.5411978042831941 | No Hit |
| GACTACAGGGGTATCTAATCCTGTTTGATCCCCACGCTTTCGCACATCAG | 769 | 0.5404949499919172 | No Hit |
| GACTACACGGGTATCTAATCCTGTTTGCTCCCCATGCTTTCGTACCTCAG | 757 | 0.5320606984965947 | No Hit |
| GACTACAAGGGTATCTAATCCTGTTTGCTCCCCACGCTTTCGCGCCTCAG | 749 | 0.5264378641663797 | No Hit |
| GACTACTAGGGTATCTAATCCTGTTTGATCCCCACGCTTTCGCACATCAG | 734 | 0.5158950497972266 | No Hit |
| GACTACAGGGGTATCTAATCCTGTTCGCTCCCCACGCTTTCGCTCCTCAG | 732 | 0.5144893412146727 | No Hit |
| GACTACCGGGGTATCTAATCCTGTTCGCTCCCCACGCTTTCGCTCCTCAG | 731 | 0.5137864869233959 | No Hit |
| GACTACTCGGGTATCTAATCCTGTTCGCTCCCCACGCTTTCGCTCCTCAG | 726 | 0.5102722154670116 | No Hit |
| GACTACACGGGTATCTAATCCTGTTTGCTCCCCACGCTTTCGCGCCTCAG | 718 | 0.5046493811367965 | No Hit |
| GACTACCGGGGTATCTAATCCTGTTTGATCCCCACGCTTTCGCACATCAG | 706 | 0.4962151296414741 | No Hit |
| GACTACCCGGGTATCTAATCCTGTTTGCTCCCCACGCTTTCGCGCCTCAG | 705 | 0.4955122753501972 | No Hit |
| GACTACTAGGGTATCTAATCCTGTTCGCTCCCCACGCTTTCGCTCCTCAG | 700 | 0.49199800389381276 | No Hit |
| GACTACTCGGGTATCTAATCCTGTTTGATCCCCACGCTTTCGCACATCAG | 689 | 0.4842666066897671 | No Hit |
| GACTACTGGGGTATCTAATCCTGTTTGCTCCCCACGCTTTCGTGCATGAG | 687 | 0.4828608981072134 | No Hit |
| GACTACCCGGGTATCTAATCCTGTTCGCTCCCCACGCTTTCGCTCCTCAG | 684 | 0.4807523352333828 | No Hit |
| GACTACAAGGGTATCTAATCCTGTTTGATCCCCACGCTTTCGCACATCAG | 683 | 0.48004948094210587 | No Hit |
| GACTACCCGGGTATCTAATCCTGTTTGCTCCCCATGCTTTCGTACCTCAG | 679 | 0.4772380637769984 | No Hit |
| GACTACAAGGGTATCTAATCCTGTTCGCTCCCCACGCTTTCGCTCCTCAG | 649 | 0.4561524350386922 | No Hit |
| GACTACCAGGGTATCTAATCCTGTTTGATCCCCACGCTTTCGCACATCAG | 629 | 0.44209534921315463 | No Hit |
| GACTACCAGGGTATCTAATCCTGTTCGCTCCCCACGCTTTCGCTCCTCAG | 608 | 0.42733540909634027 | No Hit |
| GACTACACGGGTATCTAATCCTGTTTGATCCCCACGCTTTCGCACATCAG | 596 | 0.41890115760101776 | No Hit |
| GACTACTGGGGTATCTAATCCTGTTTGCTCCCCACGCTTTCGAGCCTCAG | 592 | 0.4160897404359102 | No Hit |
| GACTACAGGGGTATCTAATCCTGTTTGCTCCCCACGCTTTCGTGCATGAG | 592 | 0.4160897404359102 | No Hit |
| GACTACCCGGGTATCTAATCCTGTTTGATCCCCACGCTTTCGCACATCAG | 590 | 0.41468403185335645 | No Hit |
| GACTACACGGGTATCTAATCCTGTTCGCTCCCCACGCTTTCGCTCCTCAG | 555 | 0.39008413165866584 | No Hit |
| GACTACTCGGGTATCTAATCCTGTTTGCTCCCCACGCTTTCGTGCATGAG | 549 | 0.3858670059110046 | No Hit |
| GACTACCGGGGTATCTAATCCTGTTTGCTCCCCACGCTTTCGTGCATGAG | 548 | 0.3851641516197277 | No Hit |
| GACTACTAGGGTATCTAATCCTGTTTGCTCCCCACGCTTTCGTGCATGAG | 542 | 0.38094702587206647 | No Hit |
| GACTACTGGGGTATCTAATCCTGTTTGCTCCCCACGCTTTCGTGCCTCAG | 541 | 0.3802441715807896 | No Hit |
| GACTACAAGGGTATCTAATCCTGTTTGCTCCCCACGCTTTCGTGCATGAG | 533 | 0.37462133725057456 | No Hit |
| GACTACCAGGGTATCTAATCCTGTTTGCTCCCCACGCTTTCGTGCATGAG | 510 | 0.3584556885512064 | No Hit |
| GACTACTGGGGTATCTAATCCTGTTCGCTACCCATGCTTTCGCTCCTCAG | 503 | 0.3535357085122683 | No Hit |
| GACTACTGGGGTATCTAATCCTGTTTGCTCCCCACGCTTTCGCACCTGAG | 501 | 0.35212999992971455 | No Hit |
| GACTACAGGGGTATCTAATCCTGTTTGCTCCCCACGCTTTCGAGCCTCAG | 494 | 0.34721001989077643 | No Hit |
| GACTACCGGGGTATCTAATCCTGTTTGCTCCCCACGCTTTCGAGCCTCAG | 488 | 0.3429928941431152 | No Hit |
| GACTACCGGGGTATCTAATCCTGTTCGCTACCCATGCTTTCGCTCCTCAG | 482 | 0.3387757683954539 | No Hit |
| GACTACACGGGTATCTAATCCTGTTTGCTCCCCACGCTTTCGTGCATGAG | 480 | 0.3373700598129002 | No Hit |
| GACTACTAGGGTATCTAATCCTGTTTGCTCCCCACGCTTTCGAGCCTCAG | 472 | 0.3317472254826852 | No Hit |
| GACTACTAGGGTATCTAATCCTGTTCGCTACCCATGCTTTCGCTCCTCAG | 470 | 0.3303415169001314 | No Hit |
| GACTACAGGGGTATCTAATCCTGTTTGCTCCCCACGCTTTCGCACCTGAG | 464 | 0.32612439115247016 | No Hit |
| GACTACCCGGGTATCTAATCCTGTTTGCTCCCCACGCTTTCGTGCATGAG | 461 | 0.32401582827863956 | No Hit |
| GACTACAGGGGTATCTAATCCTGTTTGCTCCCCACGCTTTCGTGCCTCAG | 460 | 0.3233129739873627 | No Hit |
| GACTACAGGGGTATCTAATCCTGTTCGCTACCCATGCTTTCGCTCCTCAG | 456 | 0.3205015568222552 | No Hit |
| GACTACTGGGGTATCTAATCCTGTTCGCTCCCCATGCTTTCGCTTCTCAG | 453 | 0.3183929939484245 | No Hit |
| GACTACTCGGGTATCTAATCCTGTTTGCTCCCCACGCTTTCGAGCCTCAG | 446 | 0.3134730139094864 | No Hit |
| GACTACTCGGGTATCTAATCCTGTTCGCTACCCATGCTTTCGCTCCTCAG | 446 | 0.3134730139094864 | No Hit |
| GACTACCAGGGTATCTAATCCTGTTTGCTCCCCACGCTTTCGAGCCTCAG | 438 | 0.3078501795792714 | No Hit |
| GACTACAAGGGTATCTAATCCTGTTCGCTACCCATGCTTTCGCTCCTCAG | 430 | 0.3022273452490564 | No Hit |
| GACTACCGGGGTATCTAATCCTGTTTGCTCCCCACGCTTTCGCACCTGAG | 428 | 0.3008216366665027 | No Hit |
| GACTACTCGGGTATCTAATCCTGTTTGCTCCCCACGCTTTCGCACCTGAG | 424 | 0.29801021950139517 | No Hit |
| GACTACTCGGGTATCTAATCCTGTTTGCTCCCCACGCTTTCGTGCCTCAG | 423 | 0.2973073652101183 | No Hit |
| GACTACCGGGGTATCTAATCCTGTTTGCTCCCCACGCTTTCGTGCCTCAG | 416 | 0.2923873851711802 | No Hit |
| GACTACAAGGGTATCTAATCCTGTTTGCTCCCCACGCTTTCGAGCCTCAG | 410 | 0.2881702594235189 | No Hit |
| GACTACAAGGGTATCTAATCCTGTTTGCTCCCCACGCTTTCGTGCCTCAG | 407 | 0.28606169654968827 | No Hit |
| GACTACAAGGGTATCTAATCCTGTTTGCTCCCCACGCTTTCGCACCTGAG | 406 | 0.2853588422584114 | No Hit |
| GACTACTAGGGTATCTAATCCTGTTTGCTCCCCACGCTTTCGTGCCTCAG | 405 | 0.28465598796713454 | No Hit |
| GACTACCAGGGTATCTAATCCTGTTCGCTACCCATGCTTTCGCTCCTCAG | 405 | 0.28465598796713454 | No Hit |
| GACTACCAGGGTATCTAATCCTGTTTGCTCCCCACGCTTTCGCACCTGAG | 395 | 0.2776274450543658 | No Hit |
| GACTACACGGGTATCTAATCCTGTTTGCTCCCCACGCTTTCGCACCTGAG | 392 | 0.27551888218053516 | No Hit |
| GACTACTAGGGTATCTAATCCTGTTTGCTCCCCACGCTTTCGCACCTGAG | 391 | 0.27481602788925824 | No Hit |
| GACTACCGGGGTATCTAATCCTGTTCGCTCCCCATGCTTTCGCTTCTCAG | 390 | 0.2741131735979814 | No Hit |
| GACTACCCGGGTATCTAATCCTGTTCGCTACCCATGCTTTCGCTCCTCAG | 381 | 0.2677874849764895 | No Hit |
| GACTACACGGGTATCTAATCCTGTTTGCTCCCCACGCTTTCGAGCCTCAG | 380 | 0.26708463068521265 | No Hit |
| GACTACCCGGGTATCTAATCCTGTTTGCTCCCCACGCTTTCGCACCTGAG | 371 | 0.2607589420637208 | No Hit |
| GACTACAGGGGTATCTAATCCTGTTCGCTCCCCATGCTTTCGCTTCTCAG | 369 | 0.259353233481167 | No Hit |
| GACTACCCGGGTATCTAATCCTGTTTGCTCCCCACGCTTTCGAGCCTCAG | 364 | 0.2558389620247826 | No Hit |
| GACTACCAGGGTATCTAATCCTGTTTGCTCCCCACGCTTTCGTGCCTCAG | 362 | 0.2544332534422289 | No Hit |
| GACTACTCGGGTATCTAATCCTGTTCGCTCCCCATGCTTTCGCTTCTCAG | 357 | 0.2509189819858445 | No Hit |
| GACTACCCGGGTATCTAATCCTGTTTGCTCCCCACGCTTTCGTGCCTCAG | 356 | 0.25021612769456764 | No Hit |
| GACTACTAGGGTATCTAATCCTGTTCGCTCCCCATGCTTTCGCTTCTCAG | 355 | 0.24951327340329074 | No Hit |
| GACTACTGGGGTATCTAATCCTGTTTGCTCCCCACGCTGTCGCGCCTCAG | 353 | 0.24810756482073704 | No Hit |
| GACTACCAGGGTATCTAATCCTGTTCGCTCCCCATGCTTTCGCTTCTCAG | 350 | 0.24599900194690638 | No Hit |
| GACTACACGGGTATCTAATCCTGTTCGCTACCCATGCTTTCGCTCCTCAG | 346 | 0.2431875847817989 | No Hit |
| GACTACACGGGTATCTAATCCTGTTTGCTCCCCACGCTTTCGTGCCTCAG | 326 | 0.2291304989562614 | No Hit |
| GACTACTGGGGTATCTAATCCTGTTTGCTACCCACGCTTTCGAATCTCAG | 313 | 0.219993393169662 | No Hit |
| GACTACTAGGGTATCTAATCCTGTTTGCTCCCCACGCTGTCGCGCCTCAG | 311 | 0.21858768458710826 | No Hit |
| GACTACTCGGGTATCTAATCCTGTTTGCTCCCCACGCTGTCGCGCCTCAG | 305 | 0.214370558839447 | No Hit |
| GACTACAGGGGTATCTAATCCTGTTTGCTCCCCACGCTGTCGCGCCTCAG | 304 | 0.21366770454817013 | No Hit |
| GACTACCGGGGTATCTAATCCTGTTTGCTCCCCACGCTGTCGCGCCTCAG | 300 | 0.21085628738306264 | No Hit |
| GACTACCCGGGTATCTAATCCTGTTCGCTCCCCATGCTTTCGCTTCTCAG | 296 | 0.2080448702179551 | No Hit |
| GACTACAAGGGTATCTAATCCTGTTCGCTCCCCATGCTTTCGCTTCTCAG | 292 | 0.2052334530528476 | No Hit |
| GACTACACGGGTATCTAATCCTGTTCGCTCCCCATGCTTTCGCTTCTCAG | 288 | 0.2024220358877401 | No Hit |
| GACTACTAGGGTATCTAATCCTGTTTGCTACCCACGCTTTCGAATCTCAG | 284 | 0.1996106187226326 | No Hit |
| GACTACAAGGGTATCTAATCCTGTTTGCTCCCCACGCTGTCGCGCCTCAG | 280 | 0.19679920155752512 | No Hit |
| GACTACACGGGTATCTAATCCTGTTTGCTCCCCACGCTGTCGCGCCTCAG | 276 | 0.1939877843924176 | No Hit |
| GACTACAGGGGTATCTAATCCTGTTTGCTACCCACGCTTTCGAATCTCAG | 265 | 0.18625638718837198 | No Hit |
| GACTACCGGGGTATCTAATCCTGTTTGCTACCCACGCTTTCGAATCTCAG | 258 | 0.18133640714943386 | No Hit |
| GACTACCAGGGTATCTAATCCTGTTTGCTCCCCACGCTGTCGCGCCTCAG | 255 | 0.1792278442756032 | No Hit |
| GACTACTCGGGTATCTAATCCTGTTTGCTACCCACGCTTTCGAATCTCAG | 252 | 0.1771192814017726 | No Hit |
| GACTACAAGGGTATCTAATCCTGTTTGCTACCCACGCTTTCGAATCTCAG | 251 | 0.1764164271104957 | No Hit |
| GACTACCAGGGTATCTAATCCTGTTTGCTACCCACGCTTTCGAATCTCAG | 248 | 0.17430786423666508 | No Hit |
| GACTACCCGGGTATCTAATCCTGTTTGCTCCCCACGCTGTCGCGCCTCAG | 244 | 0.1714964470715576 | No Hit |
| GACTACCCGGGTATCTAATCCTGTTTGCTACCCACGCTTTCGAATCTCAG | 239 | 0.1679821756151732 | No Hit |
| GACTACACGGGTATCTAATCCTGTTTGCTACCCACGCTTTCGAATCTCAG | 225 | 0.15814221553729696 | No Hit |
| GACTACTGGGGTATCTAATCCTGTTCGCTCCCCACACTTTCGCTCCTCAG | 219 | 0.1539250897896357 | No Hit |
| GACTACTGGGGTATCTAATCCTGTTTGCTCCCCATGCTTTCGCACCTCAG | 208 | 0.1461936925855901 | No Hit |
| GACTACAGGGGTATCTAATCCTGTTCGCTCCCCACACTTTCGCTCCTCAG | 206 | 0.14478798400303633 | No Hit |
| GACTACAGGGGTATCTAATCCGGTTCGCTCCCCACACTTTCGCGCCTCAG | 188 | 0.13213660676005257 | No Hit |
| GACTACTCGGGTATCTAATCCTGTTTGCTCCCCATGCTTTCGCACCTCAG | 187 | 0.1314337524687757 | No Hit |
| GACTACTGGGGTATCTAATCCGGTTCGCTCCCCACACTTTCGCGCCTCAG | 186 | 0.13073089817749883 | No Hit |
| GACTACCGGGGTATCTAATCCTGTTCGCTCCCCACACTTTCGCTCCTCAG | 174 | 0.12229664668217631 | No Hit |
| GACTACTAGGGTATCTAATCCTGTTTGCTCCCCATGCTTTCGCACCTCAG | 173 | 0.12159379239089944 | No Hit |
| GACTACTAGGGTATCTAATCCGGTTCGCTCCCCACACTTTCGCGCCTCAG | 173 | 0.12159379239089944 | No Hit |
| GACTACCGGGGTATCTAATCCGGTTCGCTCCCCACACTTTCGCGCCTCAG | 172 | 0.12089093809962256 | No Hit |
| GACTACCGGGGTATCTAATCCTGTTTGCTCCCCATGCTTTCGCACCTCAG | 170 | 0.11948522951706882 | No Hit |
| GACTACCCGGGTATCTAATCCTGTTCGCTCCCCACACTTTCGCTCCTCAG | 164 | 0.11526810376940758 | No Hit |
| GACTACCAGGGTATCTAATCCGGTTCGCTCCCCACACTTTCGCGCCTCAG | 163 | 0.1145652494781307 | No Hit |
| GACTACTGGGGTATCTAATCCTGTTCGCTACCCATGCTTTCGCTTCTCAG | 163 | 0.1145652494781307 | No Hit |
| GACTACAGGGGTATCTAATCCTGTTTGCTCCCCATGCTTTCGCACCTCAG | 163 | 0.1145652494781307 | No Hit |
| GACTACTAGGGTATCTAATCCTGTTCGCTCCCCACACTTTCGCTCCTCAG | 162 | 0.1138623951868538 | No Hit |
| GACTACTCGGGTATCTAATCCTGTTCGCTCCCCACACTTTCGCTCCTCAG | 161 | 0.11315954089557695 | No Hit |
| GACTACAAGGGTATCTAATCCTGTTCGCTCCCCACACTTTCGCTCCTCAG | 161 | 0.11315954089557695 | No Hit |
| GACTACACGGGTATCTAATCCGGTTCGCTCCCCACACTTTCGCGCCTCAG | 161 | 0.11315954089557695 | No Hit |
| GACTACACGGGTATCTAATCCTGTTTGCTCCCCATGCTTTCGCACCTCAG | 158 | 0.11105097802174631 | No Hit |
| GACTACAAGGGTATCTAATCCGGTTCGCTCCCCACACTTTCGCGCCTCAG | 158 | 0.11105097802174631 | No Hit |
| GACTACACGGGTATCTAATCCTGTTCGCTCCCCACACTTTCGCTCCTCAG | 157 | 0.11034812373046944 | No Hit |
| GACTACCAGGGTATCTAATCCTGTTCGCTACCCATGCTTTCGCTTCTCAG | 156 | 0.10964526943919256 | No Hit |
| GACTACCAGGGTATCTAATCCTGTTCGCTCCCCACACTTTCGCTCCTCAG | 156 | 0.10964526943919256 | No Hit |
| GACTACAGGGGTATCTAATCCTGTTCGCTACCCATGCTTTCGCTTCTCAG | 153 | 0.10753670656536193 | No Hit |
| GACTACAAGGGTATCTAATCCTGTTTGCTCCCCATGCTTTCGCACCTCAG | 153 | 0.10753670656536193 | No Hit |
| GACTACTGGGGTATCTAATCCTGTTCGCTCCCCACGCTTTCGTGCCTCAG | 151 | 0.10613099798280819 | No Hit |
| GACTACTGGGGTATCTAATCCTGTTCGCTCCCCACGCTTTCGCTTCTCAG | 144 | 0.10121101794387005 | No Hit |
| GACTACCCGGGTATCTAATCCGGTTCGCTCCCCACACTTTCGCGCCTCAG | 144 | 0.10121101794387005 | No Hit |
| GACTACTGGGGTATCTAATCCTGTTCGCTCCCCACGCTTTCGCGCCTCAG | 143 | 0.10050816365259319 | No Hit |

## Adapter Content

## Kmer Content

| Sequence | Count | PValue | Obs/Exp Max | Max Obs/Exp Position |
| --- | --- | --- | --- | --- |
| GTTTGCG | 5 | 5.0522556E-4 | 4016.707 | 295 |
| ATTAGCA | 5 | 5.0522556E-4 | 4016.707 | 295 |
| TTAGACG | 10 | 3.3212382E-7 | 4016.707 | 295 |
| GTTAGGG | 5 | 5.0522556E-4 | 4016.707 | 295 |
| GTTAGCA | 20 | 0.0 | 4016.707 | 295 |
| TTATACG | 5 | 5.0522556E-4 | 4016.707 | 295 |
| ATTTTGG | 5 | 5.0522556E-4 | 4016.707 | 295 |
| ACTCGTT | 5 | 5.0522556E-4 | 4016.707 | 295 |
| ATGACGG | 5 | 5.0522556E-4 | 4016.707 | 295 |
| GGTGCGG | 5 | 5.0522556E-4 | 4016.707 | 295 |
| TTTGCCG | 20 | 0.0 | 4016.707 | 295 |
| GATAGCG | 5 | 5.0522556E-4 | 4016.707 | 295 |
| GGTAGCG | 5 | 5.0522556E-4 | 4016.707 | 295 |
| GTTAGCG | 145 | 0.0 | 3739.6924 | 295 |
| TTAGCCG | 540 | 0.0 | 3681.9812 | 295 |
| TTAGGCG | 35 | 0.0 | 3442.8916 | 295 |
| TTAGCGG | 15 | 1.1207103E-6 | 2677.8044 | 295 |
| TTTAGCG | 20 | 2.6560083E-6 | 2008.3535 | 295 |
| CTTCCTT | 10 | 0.0020205702 | 2008.3535 | 295 |
| CGGCCGG | 10 | 0.0020205702 | 2008.3535 | 295 |

Produced by FastQC (version 0.11.7)
