# Supplementary material for: Assessment of airborne bacteria from a public health institution in Mexico City
Source: PLOS Glob Public Health. 2024 Nov 7;4(11):e0003672. doi: 10.1371/journal.pgph.0003672 (PMC11542838; doi:10.1371/journal.pgph.0003672)
Supplement: S1 Text — (ZIP) [file pgph.0003672.s001.zip › Hospital_16S_QC/21022023_CP2D2_16S_S38_L001_R1_001_fastqc.html]

21022023\_CP2D2\_16S\_S38\_L001\_R1\_001.fastq.gz FastQC Report 

FastQC Report

Wed 15 Mar 2023  
21022023\_CP2D2\_16S\_S38\_L001\_R1\_001.fastq.gz

## Summary

- Basic Statistics
- Per base sequence quality
- Per tile sequence quality
- Per sequence quality scores
- Per base sequence content
- Per sequence GC content
- Per base N content
- Sequence Length Distribution
- Sequence Duplication Levels
- Overrepresented sequences
- Adapter Content
- Kmer Content

## Basic Statistics

| Measure | Value |
| --- | --- |
| Filename | 21022023\_CP2D2\_16S\_S38\_L001\_R1\_001.fastq.gz |
| File type | Conventional base calls |
| Encoding | Sanger / Illumina 1.9 |
| Total Sequences | 653052 |
| Sequences flagged as poor quality | 0 |
| Sequence length | 40-301 |
| %GC | 55 |

## Per base sequence quality

## Per tile sequence quality

## Per sequence quality scores

## Per base sequence content

## Per sequence GC content

## Per base N content

## Sequence Length Distribution

## Sequence Duplication Levels

## Overrepresented sequences

| Sequence | Count | Percentage | Possible Source |
| --- | --- | --- | --- |
| CCTACGGGTGGCTGCAGTGGGGAATATTGCACAATGGGCGCAAGCCTGAT | 13578 | 2.079160618143731 | No Hit |
| CCTACGGGAGGCTGCAGTGGGGAATATTGCACAATGGGCGCAAGCCTGAT | 13135 | 2.0113252849696504 | No Hit |
| CCTACGGGGGGCTGCAGTGGGGAATATTGCACAATGGGCGCAAGCCTGAT | 12584 | 1.9269522182000822 | No Hit |
| CCTACGGGTGGCTGCAGTGGGGAATATTGCACAATGGGCGAAAGCCTGAT | 10893 | 1.6680141857003732 | No Hit |
| CCTACGGGAGGCTGCAGTGGGGAATATTGCACAATGGGCGAAAGCCTGAT | 10508 | 1.6090602279757202 | No Hit |
| CCTACGGGTGGCTGCAGTGGGGAATATTGGACAATGGGCGAAAGCCTGAT | 10291 | 1.575831633621825 | No Hit |
| CCTACGGGGGGCTGCAGTGGGGAATATTGCACAATGGGCGAAAGCCTGAT | 9850 | 1.5083025547735862 | No Hit |
| CCTACGGGCGGCTGCAGTGGGGAATATTGCACAATGGGCGCAAGCCTGAT | 9788 | 1.4988086706724733 | No Hit |
| CCTACGGGGGGCAGCAGTGGGGAATATTGCACAATGGGCGCAAGCCTGAT | 9684 | 1.482883445728671 | No Hit |
| CCTACGGGAGGCTGCAGTGGGGAATATTGGACAATGGGCGAAAGCCTGAT | 9518 | 1.4574643366837556 | No Hit |
| CCTACGGGGGGCAGCAGTAGGGAATCTTCCGCAATGGGCGAAAGCCTGAC | 9218 | 1.4115261878074028 | No Hit |
| CCTACGGGGGGCTGCAGTGGGGAATATTGGACAATGGGCGAAAGCCTGAT | 9050 | 1.3858008244366453 | No Hit |
| CCTACGGGAGGCAGCAGTGGGGAATATTGCACAATGGGCGCAAGCCTGAT | 8896 | 1.362219241346784 | No Hit |
| CCTACGGGTGGCTGCAGTGGGGAATATTGGACAATGGGCGCAAGCCTGAT | 8847 | 1.3547160103636462 | No Hit |
| CCTACGGGAGGCTGCAGTAGGGAATCTTCCGCAATGGGCGAAAGCCTGAC | 8720 | 1.335268860672657 | No Hit |
| CCTACGGGTGGCTGCAGTGGGGAATCTTAGACAATGGGGGCAACCCTGAT | 8667 | 1.3271531210378347 | No Hit |
| CCTACGGGTGGCTGCAGTAGGGAATCTTCCGCAATGGGCGAAAGCCTGAC | 8646 | 1.32393745061649 | No Hit |
| CCTACGGGAGGCAGCAGTAGGGAATCTTCCGCAATGGGCGAAAGCCTGAC | 8595 | 1.31612796530751 | No Hit |
| CCTACGGGAGGCTGCAGTGGGGAATATTGGACAATGGGCGCAAGCCTGAT | 8492 | 1.3003558675266287 | No Hit |
| CCTACGGGGGGCTGCAGTAGGGAATCTTCCGCAATGGGCGAAAGCCTGAC | 8440 | 1.2923932550547277 | No Hit |
| CCTACGGGAGGCTGCAGTGGGGAATCTTAGACAATGGGGGCAACCCTGAT | 8278 | 1.2675866546614971 | No Hit |
| CCTACGGGTGGCAGCAGTGGGGAATATTGCACAATGGGCGCAAGCCTGAT | 8162 | 1.2498239037626406 | No Hit |
| CCTACGGGCGGCTGCAGTGGGGAATATTGCACAATGGGCGAAAGCCTGAT | 8139 | 1.2463019790154537 | No Hit |
| CCTACGGGGGGCTGCAGTGGGGAATATTGGACAATGGGCGCAAGCCTGAT | 8027 | 1.2291517367682818 | No Hit |
| CCTACGGGTGGCAGCAGTAGGGAATCTTCCGCAATGGGCGAAAGCCTGAC | 8024 | 1.2286923552795184 | No Hit |
| CCTACGGGGGGCTGCAGTGGGGAATCTTAGACAATGGGGGCAACCCTGAT | 7979 | 1.2218016329480654 | No Hit |
| CCTACGGGCGGCTGCAGTGGGGAATATTGGACAATGGGCGAAAGCCTGAT | 7592 | 1.1625414208975702 | No Hit |
| CCTACGGGGGGCAGCAGTGGGGAATATTGCACAATGGGCGAAAGCCTGAT | 7450 | 1.140797363762763 | No Hit |
| CCTACGGGGGGCAGCAGTGGGGAATATTGGACAATGGGCGAAAGCCTGAT | 6926 | 1.0605587303920667 | No Hit |
| CCTACGGGAGGCAGCAGTGGGGAATATTGCACAATGGGCGAAAGCCTGAT | 6895 | 1.0558117883415103 | No Hit |
| CCTACGGGCGGCTGCAGTGGGGAATATTGGACAATGGGCGCAAGCCTGAT | 6723 | 1.029473916319068 | No Hit |
| CCTACGGGCGGCTGCAGTGGGGAATCTTAGACAATGGGGGCAACCCTGAT | 6489 | 0.9936421601955128 | No Hit |
| CCTACGGGCGGCTGCAGTAGGGAATCTTCCGCAATGGGCGAAAGCCTGAC | 6470 | 0.9907327441000104 | No Hit |
| CCTACGGGTGGCAGCAGTGGGGAATATTGCACAATGGGCGAAAGCCTGAT | 6420 | 0.9830763859539515 | No Hit |
| CCTACGGGCGGCAGCAGTGGGGAATATTGCACAATGGGCGCAAGCCTGAT | 6373 | 0.9758794092966563 | No Hit |
| CCTACGGGAGGCAGCAGTGGGGAATATTGGACAATGGGCGAAAGCCTGAT | 6347 | 0.9718981030607057 | No Hit |
| CCTACGGGCGGCAGCAGTAGGGAATCTTCCGCAATGGGCGAAAGCCTGAC | 6265 | 0.9593416757011693 | No Hit |
| CCTACGGGGGGCAGCAGTGGGGAATATTGGACAATGGGCGCAAGCCTGAT | 6015 | 0.9210598849708752 | No Hit |
| CCTACGGGGGGCAGCAGTGGGGAATCTTAGACAATGGGGGCAACCCTGAT | 5964 | 0.9132503996618953 | No Hit |
| CCTACGGGTGGCAGCAGTGGGGAATATTGGACAATGGGCGAAAGCCTGAT | 5752 | 0.8807874411226059 | No Hit |
| CCTACGGGAGGCAGCAGTGGGGAATATTGGACAATGGGCGCAAGCCTGAT | 5699 | 0.8726717014877835 | No Hit |
| CCTACGGGAGGCAGCAGTGGGGAATCTTAGACAATGGGGGCAACCCTGAT | 5638 | 0.8633309445495918 | No Hit |
| CCTACGGGCGGCAGCAGTGGGGAATATTGCACAATGGGCGAAAGCCTGAT | 5025 | 0.7694639936789107 | No Hit |
| CCTACGGGTGGCAGCAGTGGGGAATATTGGACAATGGGCGCAAGCCTGAT | 5010 | 0.767167086235093 | No Hit |
| CCTACGGGTGGCAGCAGTGGGGAATCTTAGACAATGGGGGCAACCCTGAT | 4902 | 0.7506293526396061 | No Hit |
| CCTACGGGTGGCTGCAGTGGGGAATATTGCACAATGGGCGGAAGCCTGAT | 4672 | 0.7154101051677355 | No Hit |
| CCTACGGGCGGCAGCAGTGGGGAATATTGGACAATGGGCGAAAGCCTGAT | 4627 | 0.7085193828362826 | No Hit |
| CCTACGGGAGGCTGCAGTGGGGAATATTGCACAATGGGCGGAAGCCTGAT | 4436 | 0.6792720947183379 | No Hit |
| CCTACGGGCGGCAGCAGTGGGGAATATTGGACAATGGGCGCAAGCCTGAT | 4301 | 0.6585999277239791 | No Hit |
| CCTACGGGCGGCAGCAGTGGGGAATCTTAGACAATGGGGGCAACCCTGAT | 4178 | 0.6397652866846744 | No Hit |
| CCTACGGGGGGCTGCAGTGGGGAATATTGCACAATGGGCGGAAGCCTGAT | 4170 | 0.638540269381305 | No Hit |
| CCTACGGGCGGCTGCAGTGGGGAATATTGCACAATGGGCGGAAGCCTGAT | 3456 | 0.5292074750555852 | No Hit |
| CCTACGGGGGGCAGCAGTGGGGAATATTGCACAATGGGCGGAAGCCTGAT | 3252 | 0.49796953381966524 | No Hit |
| CCTACGGGAGGCAGCAGTGGGGAATATTGCACAATGGGCGGAAGCCTGAT | 2838 | 0.43457488837029823 | No Hit |
| CCTACGGGTGGCTGCAGTGGGGAATTTTCCGCAATGGGCGAAAGCCTGAC | 2717 | 0.4160465016568359 | No Hit |
| CCTACGGGTGGCAGCAGTGGGGAATATTGCACAATGGGCGGAAGCCTGAT | 2624 | 0.4018056755051665 | No Hit |
| CCTACGGGAGGCTGCAGTGGGGAATTTTCCGCAATGGGCGAAAGCCTGAC | 2586 | 0.3959868433141618 | No Hit |
| CCTACGGGAGGCTGCAGTGGGGAATTTTGGACAATGGGCGCAAGCCTGAT | 2500 | 0.38281790730294063 | No Hit |
| CCTACGGGTGGCTGCAGTGGGGAATTTTGGACAATGGGCGCAAGCCTGAT | 2492 | 0.38159288999957125 | No Hit |
| CCTACGGGGGGCTGCAGTGGGGAATTTTCCGCAATGGGCGAAAGCCTGAC | 2375 | 0.3636770119377936 | No Hit |
| CCTACGGGTGGCTGCAGTGGGGAATATTGGACAATGGGGGGAACCCTGAT | 2254 | 0.34514862522433126 | No Hit |
| CCTACGGGGGGCTGCAGTGGGGAATTTTGGACAATGGGCGCAAGCCTGAT | 2230 | 0.34147357331422307 | No Hit |
| CCTACGGGCGGCAGCAGTGGGGAATATTGCACAATGGGCGGAAGCCTGAT | 2186 | 0.3347359781456913 | No Hit |
| CCTACGGGGGGCAGCAGTAGGGAATCTTCCGCAATGGACGAAAGTCTGAC | 2159 | 0.3306015447468196 | No Hit |
| CCTACGGGAGGCTGCAGTGGGGAATATTGGACAATGGGGGGAACCCTGAT | 2139 | 0.32753900148839604 | No Hit |
| CCTACGGGAGGCAGCAGTAGGGAATCTTCCGCAATGGACGAAAGTCTGAC | 2121 | 0.32478271255581487 | No Hit |
| CCTACGGGTGGCTGCAGTAGGGAATCTTCCGCAATGGACGAAAGTCTGAC | 2102 | 0.3218732964603125 | No Hit |
| CCTACGGGAGGCTGCAGTAGGGAATCTTCCGCAATGGACGAAAGTCTGAC | 2052 | 0.3142169383142537 | No Hit |
| CCTACGGGTGGCTGCAGTGGGGAATATTGCACAATGGGGGAAACCCTGAT | 2028 | 0.3105418864041455 | No Hit |
| CCTACGGGGGGCTGCAGTGGGGAATATTGGACAATGGGGGGAACCCTGAT | 1972 | 0.3019667652805596 | No Hit |
| CCTACGGGCGGCTGCAGTGGGGAATTTTCCGCAATGGGCGAAAGCCTGAC | 1951 | 0.2987510948592149 | No Hit |
| CCTACGGGGGGCTGCAGTAGGGAATCTTCCGCAATGGACGAAAGTCTGAC | 1933 | 0.2959948059266337 | No Hit |
| CCTACGGGTGGCAGCAGTAGGGAATCTTCCGCAATGGACGAAAGTCTGAC | 1894 | 0.2900228465727078 | No Hit |
| CCTACGGGGGGCAGCAGTGGGGAATTTTCCGCAATGGGCGAAAGCCTGAC | 1834 | 0.2808352167974373 | No Hit |
| CCTACGGGCGGCTGCAGTGGGGAATTTTGGACAATGGGCGCAAGCCTGAT | 1803 | 0.2760882747468808 | No Hit |
| CCTACGGGGGGCTGCAGTGGGGAATATTGCACAATGGGGGAAACCCTGAT | 1794 | 0.2747101302805902 | No Hit |
| CCTACGGGAGGCTGCAGTGGGGAATATTGCACAATGGGGGAAACCCTGAT | 1770 | 0.27103507837048196 | No Hit |
| CCTACGGGTGGCTGCAGTGGGGAATCTTGGACAATGGGGGCAACCCTGAT | 1710 | 0.2618474485952114 | No Hit |
| CCTACGGGCGGCTGCAGTGGGGAATATTGGACAATGGGGGGAACCCTGAT | 1674 | 0.25633487073004907 | No Hit |
| CCTACGGGAGGCAGCAGTGGGGAATTTTCCGCAATGGGCGAAAGCCTGAC | 1650 | 0.2526598188199408 | No Hit |
| CCTACGGGGGGCAGCAGTGGGGAATTTTGGACAATGGGCGCAAGCCTGAT | 1649 | 0.25250669165701967 | No Hit |
| CCTACGGGAGGCAGCAGTGGGGAATTTTGGACAATGGGCGCAAGCCTGAT | 1629 | 0.2494441483985961 | No Hit |
| CCTACGGGAGGCTGCAGTGGGGAATCTTGGACAATGGGGGCAACCCTGAT | 1608 | 0.24622847797725142 | No Hit |
| CCTACGGGTGGCTGCAGTGGGGAATATTGGACAATGGGGGCAACCCTGAT | 1581 | 0.24209404457837966 | No Hit |
| CCTACGGGCGGCTGCAGTAGGGAATCTTCCGCAATGGACGAAAGTCTGAC | 1524 | 0.23336579629187262 | No Hit |
| CCTACGGGCGGCAGCAGTAGGGAATCTTCCGCAATGGACGAAAGTCTGAC | 1506 | 0.23060950735929145 | No Hit |
| CCTACGGGAGGCAGCAGTGGGGAATATTGGACAATGGGGGGAACCCTGAT | 1505 | 0.23045638019637027 | No Hit |
| CCTACGGGGGGCAGCAGTGGGGAATATTGGACAATGGGGGGAACCCTGAT | 1486 | 0.22754696410086794 | No Hit |
| CCTACGGGGGGCTGCAGTGGGGAATCTTGGACAATGGGGGCAACCCTGAT | 1466 | 0.2244844208424444 | No Hit |
| CCTACGGGTGGCAGCAGTGGGGAATTTTCCGCAATGGGCGAAAGCCTGAC | 1461 | 0.22371878502783854 | No Hit |
| CCTACGGGCGGCTGCAGTGGGGAATATTGCACAATGGGGGAAACCCTGAT | 1444 | 0.22111562325817852 | No Hit |
| CCTACGGGAGGCTGCAGTGGGGAATATTGGACAATGGGGGCAACCCTGAT | 1431 | 0.2191249701402032 | No Hit |
| CCTACGGGTGGCTGCAGTGGGGAATATTGCGCAATGGGCGGAAGCCTGAC | 1416 | 0.21682806269638558 | No Hit |
| CCTACGGGGGGCTGCAGTGGGGAATATTGGACAATGGGGGCAACCCTGAT | 1409 | 0.21575617255593735 | No Hit |
| CCTACGGGTGGCTGCAGTGGGGAATATTGCGCAATGGGCGAAAGCCTGAC | 1389 | 0.21269362929751384 | No Hit |
| CCTACGGGTGGCAGCAGTGGGGAATTTTGGACAATGGGCGCAAGCCTGAT | 1386 | 0.21223424780875028 | No Hit |
| CCTACGGGCGGCTGCAGTGGGGAATCTTGGACAATGGGGGCAACCCTGAT | 1338 | 0.20488414398853383 | No Hit |
| CCTACGGGCGGCAGCAGTGGGGAATTTTCCGCAATGGGCGAAAGCCTGAC | 1323 | 0.2025872365447162 | No Hit |
| CCTACGGGAGGCTGCAGTGGGGAATATTGCGCAATGGGCGAAAGCCTGAC | 1296 | 0.19845280314584446 | No Hit |
| CCTACGGGGGGCAGCAGTGGGGAATATTGCACAATGGGGGAAACCCTGAT | 1283 | 0.19646215002786915 | No Hit |
| CCTACGGGAGGCTGCAGTGGGGAATATTGCGCAATGGGCGGAAGCCTGAC | 1257 | 0.19248084379191857 | No Hit |
| CCTACGGGGGGCAGCAGTGGGGAATCTTGGACAATGGGGGCAACCCTGAT | 1235 | 0.18911204620765268 | No Hit |
| CCTACGGGGGGCTGCAGTGGGGAATATTGCGCAATGGGCGGAAGCCTGAC | 1226 | 0.1877339017413621 | No Hit |
| CCTACGGGTGGCAGCAGTGGGGAATATTGGACAATGGGGGGAACCCTGAT | 1222 | 0.1871213930896774 | No Hit |
| CCTACGGGAGGCAGCAGTGGGGAATATTGCACAATGGGGGAAACCCTGAT | 1213 | 0.18574324862338681 | No Hit |
| CCTACGGGGGGCTGCAGTGGGGAATATTGCGCAATGGGCGAAAGCCTGAC | 1208 | 0.18497761280878092 | No Hit |
| CCTACGGGCGGCAGCAGTGGGGAATTTTGGACAATGGGCGCAAGCCTGAT | 1141 | 0.17471809289306212 | No Hit |
| CCTACGGGCGGCAGCAGTGGGGAATATTGGACAATGGGGGGAACCCTGAT | 1105 | 0.16920551502789977 | No Hit |
| CCTACGGGGGGCAGCAGTAGGGAATCTTCCGCAATGGACGCAAGTCTGAC | 1105 | 0.16920551502789977 | No Hit |
| CCTACGGGAGGCAGCAGTGGGGAATCTTGGACAATGGGGGCAACCCTGAT | 1096 | 0.16782737056160918 | No Hit |
| CCTACGGGAGGCTGCAGTAGGGAATCTTCCGCAATGGACGCAAGTCTGAC | 1089 | 0.16675548042116095 | No Hit |
| CCTACGGGTGGCTGCAGTAGGGAATCTTCCGCAATGGACGCAAGTCTGAC | 1077 | 0.16491795446610683 | No Hit |
| CCTACGGGAGGCAGCAGTAGGGAATCTTCCGCAATGGACGCAAGTCTGAC | 1072 | 0.16415231865150096 | No Hit |
| CCTACGGGCGGCTGCAGTGGGGAATATTGGACAATGGGGGCAACCCTGAT | 1064 | 0.16292730134813152 | No Hit |
| CCTACGGGTGGCAGCAGTGGGGAATATTGCACAATGGGGGAAACCCTGAT | 1044 | 0.15986475808970801 | No Hit |
| CCTACGGGTGGCAGCAGTAGGGAATCTTCCGCAATGGACGCAAGTCTGAC | 1041 | 0.1594053766009445 | No Hit |
| CCTACGGGCGGCTGCAGTGGGGAATATTGCGCAATGGGCGGAAGCCTGAC | 1039 | 0.15909912227510214 | No Hit |
| CCTACGGGGGGCTGCAGTAGGGAATCTTCCGCAATGGACGCAAGTCTGAC | 1030 | 0.15772097780881153 | No Hit |
| CCTACGGGGGGCAGCAGTGGGGAATATTGGACAATGGGGGCAACCCTGAT | 1020 | 0.1561897061795998 | No Hit |
| CCTACGGGTGGCTGCAGTGGGGAATATTGGACAATGGGCGGAAGCCTGAT | 1019 | 0.1560365790166786 | No Hit |
| CCTACGGGCGGCTGCAGTGGGGAATATTGCGCAATGGGCGAAAGCCTGAC | 995 | 0.1523615271065704 | No Hit |
| CCTACGGGAGGCAGCAGTGGGGAATATTGGACAATGGGGGCAACCCTGAT | 990 | 0.1515958912919645 | No Hit |
| CCTACGGGTGGCAGCAGTGGGGAATCTTGGACAATGGGGGCAACCCTGAT | 956 | 0.1463895677526445 | No Hit |
| CCTACGGGAGGCTGCAGTGGGGAATATTGGACAATGGGCGGAAGCCTGAT | 953 | 0.145930186263881 | No Hit |
| CCTACGGGGGGCAGCAGTGGGGAATATTGCGCAATGGGCGGAAGCCTGAC | 951 | 0.14562393193803863 | No Hit |
| CCTACGGGAGGCAGCAGTGGGGAATATTGCGCAATGGGCGAAAGCCTGAC | 907 | 0.13888633676950687 | No Hit |
| CCTACGGGCGGCAGCAGTGGGGAATATTGCACAATGGGGGAAACCCTGAT | 904 | 0.13842695528074334 | No Hit |
| CCTACGGGGGGCAGCAGTGGGGAATATTGCGCAATGGGCGAAAGCCTGAC | 903 | 0.13827382811782216 | No Hit |
| CCTACGGGGGGCTGCAGTGGGGAATATTGGACAATGGGCGGAAGCCTGAT | 887 | 0.13582379351108334 | No Hit |
| CCTACGGGCGGCTGCAGTAGGGAATCTTCCGCAATGGACGCAAGTCTGAC | 834 | 0.127708053876261 | No Hit |
| CCTACGGGTGGCAGCAGTGGGGAATATTGCGCAATGGGCGAAAGCCTGAC | 833 | 0.12755492671333982 | No Hit |
| CCTACGGGTGGCTGCAGTGGGGAATCTTGCGCAATGGGCGAAAGCCTGAC | 832 | 0.12740179955041867 | No Hit |
| CCTACGGGTGGCAGCAGTGGGGAATATTGGACAATGGGGGCAACCCTGAT | 820 | 0.12556427359536454 | No Hit |
| CCTACGGGAGGCAGCAGTGGGGAATATTGCGCAATGGGCGGAAGCCTGAC | 814 | 0.12464551061783748 | No Hit |
| CCTACGGGAGGCTGCAGTGGGGAATCTTGCGCAATGGGCGAAAGCCTGAC | 790 | 0.12097045870772924 | No Hit |
| CCTACGGGCGGCAGCAGTGGGGAATCTTGGACAATGGGGGCAACCCTGAT | 780 | 0.11943918707851749 | No Hit |
| CCTACGGGCGGCAGCAGTAGGGAATCTTCCGCAATGGACGCAAGTCTGAC | 780 | 0.11943918707851749 | No Hit |
| CCTACGGGTGGCAGCAGTGGGGAATATTGCGCAATGGGCGGAAGCCTGAC | 768 | 0.11760166112346336 | No Hit |
| CCTACGGGCGGCTGCAGTGGGGAATATTGGACAATGGGCGGAAGCCTGAT | 760 | 0.11637664382009397 | No Hit |
| CCTACGGGAGGCAGCAGTAGGGAATCTTCCACAATGGACGAAAGTCTGAT | 748 | 0.11453911786503984 | No Hit |
| CCTACGGGGGGCAGCAGTAGGGAATCTTCCACAATGGACGAAAGTCTGAT | 743 | 0.11377348205043397 | No Hit |
| CCTACGGGGGGCTGCAGTGGGGAATCTTGCGCAATGGGCGAAAGCCTGAC | 710 | 0.10872028567403515 | No Hit |
| CCTACGGGTGGCTGCAGTAGGGAATCTTCCACAATGGACGAAAGTCTGAT | 710 | 0.10872028567403515 | No Hit |
| CCTACGGGAGGCTGCAGTAGGGAATCTTCCACAATGGACGAAAGTCTGAT | 704 | 0.1078015226965081 | No Hit |
| CCTACGGGCGGCAGCAGTGGGGAATATTGGACAATGGGGGCAACCCTGAT | 698 | 0.10688275971898102 | No Hit |
| CCTACGGGTGGCAGCAGTAGGGAATCTTCCACAATGGACGAAAGTCTGAT | 679 | 0.10397334362347867 | No Hit |

## Adapter Content

## Kmer Content

| Sequence | Count | PValue | Obs/Exp Max | Max Obs/Exp Position |
| --- | --- | --- | --- | --- |
| ATAATAG | 10 | 6.886257E-4 | 316.03555 | 295 |
| GTAAGAT | 125 | 0.0 | 316.03555 | 295 |
| ATTGCGG | 10 | 6.886257E-4 | 316.03555 | 295 |
| ATTTCGA | 50 | 0.0 | 316.03555 | 295 |
| GAGAGAG | 6565 | 0.0 | 313.62857 | 295 |
| AGGTATG | 800 | 0.0 | 310.1099 | 295 |
| ATCCAAA | 2820 | 0.0 | 308.75104 | 295 |
| AGTGTTG | 885 | 0.0 | 307.108 | 295 |
| ATGTGAT | 495 | 0.0 | 306.4587 | 295 |
| ATCTGAT | 435 | 0.0 | 301.5052 | 295 |
| GGGAGAG | 2770 | 0.0 | 297.21033 | 295 |
| CCTACGG | 63980 | 0.0 | 293.02783 | 1 |
| GGGTGGC | 17265 | 0.0 | 292.56885 | 6 |
| CTACGGG | 64890 | 0.0 | 292.54382 | 2 |
| AGGCAGC | 7705 | 0.0 | 292.49002 | 9 |
| TGGCTGC | 10580 | 0.0 | 292.0711 | 9 |
| GGGAGGC | 17710 | 0.0 | 291.52612 | 6 |
| GGGCGGC | 12765 | 0.0 | 291.25244 | 6 |
| CGGGTGG | 17365 | 0.0 | 291.05338 | 5 |
| GCGGCAG | 5340 | 0.0 | 290.98813 | 8 |

Produced by FastQC (version 0.11.7)
