# Supplementary material for: Assessment of airborne bacteria from a public health institution in Mexico City
Source: PLOS Glob Public Health. 2024 Nov 7;4(11):e0003672. doi: 10.1371/journal.pgph.0003672 (PMC11542838; doi:10.1371/journal.pgph.0003672)
Supplement: S1 Text — (ZIP) [file pgph.0003672.s001.zip › Hospital_16S_QC/21022023_CP2D3_16S_S39_L001_R1_001_fastqc.html]

21022023\_CP2D3\_16S\_S39\_L001\_R1\_001.fastq.gz FastQC Report 

FastQC Report

Wed 15 Mar 2023  
21022023\_CP2D3\_16S\_S39\_L001\_R1\_001.fastq.gz

## Summary

- Basic Statistics
- Per base sequence quality
- Per tile sequence quality
- Per sequence quality scores
- Per base sequence content
- Per sequence GC content
- Per base N content
- Sequence Length Distribution
- Sequence Duplication Levels
- Overrepresented sequences
- Adapter Content
- Kmer Content

## Basic Statistics

| Measure | Value |
| --- | --- |
| Filename | 21022023\_CP2D3\_16S\_S39\_L001\_R1\_001.fastq.gz |
| File type | Conventional base calls |
| Encoding | Sanger / Illumina 1.9 |
| Total Sequences | 431674 |
| Sequences flagged as poor quality | 0 |
| Sequence length | 35-301 |
| %GC | 56 |

## Per base sequence quality

## Per tile sequence quality

## Per sequence quality scores

## Per base sequence content

## Per sequence GC content

## Per base N content

## Sequence Length Distribution

## Sequence Duplication Levels

## Overrepresented sequences

| Sequence | Count | Percentage | Possible Source |
| --- | --- | --- | --- |
| CCTACGGGTGGCTGCAGTGGGGAATATTGCACAATGGGCGCAAGCCTGAT | 8117 | 1.88035415614561 | No Hit |
| CCTACGGGTGGCTGCAGTGGGGAATATTGCACAATGGGCGAAAGCCTGAT | 7556 | 1.7503949739849978 | No Hit |
| CCTACGGGAGGCTGCAGTGGGGAATATTGCACAATGGGCGCAAGCCTGAT | 7458 | 1.7276926569587236 | No Hit |
| CCTACGGGGGGCTGCAGTGGGGAATATTGCACAATGGGCGCAAGCCTGAT | 6926 | 1.6044515073875192 | No Hit |
| CCTACGGGAGGCTGCAGTGGGGAATATTGCACAATGGGCGAAAGCCTGAT | 6907 | 1.6000500377599762 | No Hit |
| CCTACGGGGGGCTGCAGTGGGGAATATTGCACAATGGGCGAAAGCCTGAT | 6550 | 1.517348740021405 | No Hit |
| CCTACGGGCGGCTGCAGTGGGGAATATTGCACAATGGGCGCAAGCCTGAT | 5612 | 1.3000551341984923 | No Hit |
| CCTACGGGCGGCTGCAGTGGGGAATATTGCACAATGGGCGAAAGCCTGAT | 5583 | 1.2933371016090847 | No Hit |
| CCTACGGGGGGCAGCAGTGGGGAATATTGCACAATGGGCGCAAGCCTGAT | 5076 | 1.1758873594425423 | No Hit |
| CCTACGGGTGGCTGCAGTGGGGAATCTTAGACAATGGGGGCAACCCTGAT | 4855 | 1.1246913179853315 | No Hit |
| CCTACGGGAGGCAGCAGTGGGGAATATTGCACAATGGGCGCAAGCCTGAT | 4826 | 1.1179732853959239 | No Hit |
| CCTACGGGTGGCTGCAGTGGGGAATATTGGACAATGGGCGCAAGCCTGAT | 4808 | 1.1138034720645673 | No Hit |
| CCTACGGGGGGCAGCAGTGGGGAATATTGCACAATGGGCGAAAGCCTGAT | 4733 | 1.0964292498505817 | No Hit |
| CCTACGGGAGGCTGCAGTGGGGAATCTTAGACAATGGGGGCAACCCTGAT | 4597 | 1.0649239935692212 | No Hit |
| CCTACGGGTGGCTGCAGTGGGGAATATTGGACAATGGGCGAAAGCCTGAT | 4575 | 1.0598275550531187 | No Hit |
| CCTACGGGTGGCTGCAGTGGGGAATATTGCACAATGGGCGGAAGCCTGAT | 4462 | 1.033650393584047 | No Hit |
| CCTACGGGAGGCTGCAGTGGGGAATATTGGACAATGGGCGCAAGCCTGAT | 4457 | 1.0324921121031148 | No Hit |
| CCTACGGGTGGCAGCAGTGGGGAATATTGCACAATGGGCGCAAGCCTGAT | 4365 | 1.0111797328539591 | No Hit |
| CCTACGGGAGGCTGCAGTGGGGAATATTGGACAATGGGCGAAAGCCTGAT | 4339 | 1.005156669153111 | No Hit |
| CCTACGGGAGGCAGCAGTGGGGAATATTGCACAATGGGCGAAAGCCTGAT | 4298 | 0.9956587610094655 | No Hit |
| CCTACGGGGGGCTGCAGTGGGGAATCTTAGACAATGGGGGCAACCCTGAT | 4278 | 0.9910256350857359 | No Hit |
| CCTACGGGGGGCTGCAGTGGGGAATATTGGACAATGGGCGCAAGCCTGAT | 4237 | 0.9815277269420907 | No Hit |
| CCTACGGGAGGCTGCAGTGGGGAATATTGCACAATGGGCGGAAGCCTGAT | 4177 | 0.9676283491709021 | No Hit |
| CCTACGGGGGGCTGCAGTGGGGAATATTGGACAATGGGCGAAAGCCTGAT | 4066 | 0.9419145002942035 | No Hit |
| CCTACGGGGGGCTGCAGTGGGGAATATTGCACAATGGGCGGAAGCCTGAT | 3966 | 0.9187488706755561 | No Hit |
| CCTACGGGTGGCAGCAGTGGGGAATATTGCACAATGGGCGAAAGCCTGAT | 3897 | 0.9027645862386894 | No Hit |
| CCTACGGGGGGCAGCAGTAGGGAATCTTCCGCAATGGGCGAAAGCCTGAC | 3835 | 0.8884018958751281 | No Hit |
| CCTACGGGAGGCTGCAGTAGGGAATCTTCCGCAATGGGCGAAAGCCTGAC | 3823 | 0.8856220203208902 | No Hit |
| CCTACGGGTGGCTGCAGTAGGGAATCTTCCGCAATGGGCGAAAGCCTGAC | 3740 | 0.8663945477374129 | No Hit |
| CCTACGGGAGGCAGCAGTAGGGAATCTTCCGCAATGGGCGAAAGCCTGAC | 3639 | 0.8429972618225791 | No Hit |
| CCTACGGGGGGCTGCAGTAGGGAATCTTCCGCAATGGGCGAAAGCCTGAC | 3614 | 0.8372058544179172 | No Hit |
| CCTACGGGCGGCTGCAGTGGGGAATATTGGACAATGGGCGCAAGCCTGAT | 3556 | 0.8237697892391017 | No Hit |
| CCTACGGGCGGCAGCAGTGGGGAATATTGCACAATGGGCGCAAGCCTGAT | 3538 | 0.8195999759077452 | No Hit |
| CCTACGGGCGGCTGCAGTGGGGAATATTGGACAATGGGCGAAAGCCTGAT | 3481 | 0.8063955670251162 | No Hit |
| CCTACGGGTGGCAGCAGTAGGGAATCTTCCGCAATGGGCGAAAGCCTGAC | 3472 | 0.8043106603594379 | No Hit |
| CCTACGGGCGGCTGCAGTGGGGAATCTTAGACAATGGGGGCAACCCTGAT | 3431 | 0.7948127522157925 | No Hit |
| CCTACGGGCGGCAGCAGTGGGGAATATTGCACAATGGGCGAAAGCCTGAT | 3344 | 0.7746586544475692 | No Hit |
| CCTACGGGCGGCTGCAGTGGGGAATATTGCACAATGGGCGGAAGCCTGAT | 3295 | 0.763307495934432 | No Hit |
| CCTACGGGGGGCAGCAGTGGGGAATATTGGACAATGGGCGCAAGCCTGAT | 3099 | 0.7179028618818831 | No Hit |
| CCTACGGGGGGCAGCAGTGGGGAATATTGGACAATGGGCGAAAGCCTGAT | 3028 | 0.7014552648526434 | No Hit |
| CCTACGGGAGGCAGCAGTGGGGAATATTGGACAATGGGCGCAAGCCTGAT | 2898 | 0.6713399463484018 | No Hit |
| CCTACGGGGGGCAGCAGTGGGGAATATTGCACAATGGGCGGAAGCCTGAT | 2881 | 0.6674017893132317 | No Hit |
| CCTACGGGGGGCAGCAGTGGGGAATCTTAGACAATGGGGGCAACCCTGAT | 2856 | 0.6616103819085699 | No Hit |
| CCTACGGGAGGCAGCAGTGGGGAATATTGGACAATGGGCGAAAGCCTGAT | 2828 | 0.6551240056153487 | No Hit |
| CCTACGGGAGGCAGCAGTGGGGAATCTTAGACAATGGGGGCAACCCTGAT | 2800 | 0.6486376293221273 | No Hit |
| CCTACGGGAGGCAGCAGTGGGGAATATTGCACAATGGGCGGAAGCCTGAT | 2689 | 0.6229237804454287 | No Hit |
| CCTACGGGCGGCTGCAGTAGGGAATCTTCCGCAATGGGCGAAAGCCTGAC | 2618 | 0.606476183416189 | No Hit |
| CCTACGGGCGGCAGCAGTAGGGAATCTTCCGCAATGGGCGAAAGCCTGAC | 2616 | 0.6060128708238162 | No Hit |
| CCTACGGGTGGCAGCAGTGGGGAATATTGGACAATGGGCGAAAGCCTGAT | 2606 | 0.6036963078619514 | No Hit |
| CCTACGGGTGGCAGCAGTGGGGAATCTTAGACAATGGGGGCAACCCTGAT | 2533 | 0.5867853982403388 | No Hit |
| CCTACGGGTGGCAGCAGTGGGGAATATTGGACAATGGGCGCAAGCCTGAT | 2502 | 0.579604053058558 | No Hit |
| CCTACGGGTGGCTGCAGTAGGGAATCTTCCGCAATGGACGAAAGTCTGAC | 2499 | 0.5789090841699986 | No Hit |
| CCTACGGGGGGCAGCAGTAGGGAATCTTCCGCAATGGACGAAAGTCTGAC | 2444 | 0.5661679878797425 | No Hit |
| CCTACGGGAGGCAGCAGTAGGGAATCTTCCGCAATGGACGAAAGTCTGAC | 2401 | 0.5562067671437242 | No Hit |
| CCTACGGGAGGCTGCAGTAGGGAATCTTCCGCAATGGACGAAAGTCTGAC | 2383 | 0.5520369538123676 | No Hit |
| CCTACGGGTGGCAGCAGTGGGGAATATTGCACAATGGGCGGAAGCCTGAT | 2348 | 0.5439289834458411 | No Hit |
| CCTACGGGGGGCTGCAGTAGGGAATCTTCCGCAATGGACGAAAGTCTGAC | 2283 | 0.5288713241937203 | No Hit |
| CCTACGGGCGGCAGCAGTGGGGAATATTGGACAATGGGCGCAAGCCTGAT | 2187 | 0.5066323197598187 | No Hit |
| CCTACGGGTGGCAGCAGTAGGGAATCTTCCGCAATGGACGAAAGTCTGAC | 2161 | 0.5006092560589704 | No Hit |
| CCTACGGGCGGCAGCAGTGGGGAATCTTAGACAATGGGGGCAACCCTGAT | 2093 | 0.4848566279182902 | No Hit |
| CCTACGGGCGGCAGCAGTGGGGAATATTGGACAATGGGCGAAAGCCTGAT | 2068 | 0.4790652205136283 | No Hit |
| CCTACGGGCGGCAGCAGTGGGGAATATTGCACAATGGGCGGAAGCCTGAT | 1991 | 0.46122768570726985 | No Hit |
| CCTACGGGTGGCTGCAGTGGGGAATATTGCGCAATGGGCGGAAGCCTGAC | 1991 | 0.46122768570726985 | No Hit |
| CCTACGGGAGGCTGCAGTGGGGAATATTGCGCAATGGGCGGAAGCCTGAC | 1891 | 0.4380620560886225 | No Hit |
| CCTACGGGTGGCTGCAGTGGGGAATATTGCGCAATGGGCGAAAGCCTGAC | 1813 | 0.41999286498607746 | No Hit |
| CCTACGGGCGGCTGCAGTAGGGAATCTTCCGCAATGGACGAAAGTCTGAC | 1802 | 0.41744464572802625 | No Hit |
| CCTACGGGCGGCAGCAGTAGGGAATCTTCCGCAATGGACGAAAGTCTGAC | 1763 | 0.40841005017675375 | No Hit |
| CCTACGGGGGGCTGCAGTGGGGAATATTGCGCAATGGGCGGAAGCCTGAC | 1720 | 0.3984488294407354 | No Hit |
| CCTACGGGTGGCTGCAGTGGGGAATATTGGACAATGGGGGCAACCCTGAT | 1686 | 0.3905725153703952 | No Hit |
| CCTACGGGTGGCTGCAGTGGGGAATTTTCCGCAATGGGCGAAAGCCTGAC | 1686 | 0.3905725153703952 | No Hit |
| CCTACGGGTGGCTGCAGTGGGGAATATTGCACAATGGGGGAAACCCTGAT | 1672 | 0.3873293272237846 | No Hit |
| CCTACGGGTGGCTGCAGTGGGGAATTTTGGACAATGGGCGCAAGCCTGAT | 1657 | 0.3838544827809875 | No Hit |
| CCTACGGGAGGCTGCAGTGGGGAATATTGCGCAATGGGCGAAAGCCTGAC | 1586 | 0.36740688575174785 | No Hit |
| CCTACGGGAGGCTGCAGTGGGGAATTTTGGACAATGGGCGCAAGCCTGAT | 1586 | 0.36740688575174785 | No Hit |
| CCTACGGGCGGCTGCAGTGGGGAATATTGCGCAATGGGCGGAAGCCTGAC | 1585 | 0.36717522945556136 | No Hit |
| CCTACGGGGGGCTGCAGTGGGGAATATTGCGCAATGGGCGAAAGCCTGAC | 1530 | 0.3544341331653053 | No Hit |
| CCTACGGGGGGCTGCAGTGGGGAATTTTGGACAATGGGCGCAAGCCTGAT | 1516 | 0.35119094501869463 | No Hit |
| CCTACGGGAGGCTGCAGTGGGGAATATTGGACAATGGGGGCAACCCTGAT | 1514 | 0.3507276324263217 | No Hit |
| CCTACGGGAGGCTGCAGTGGGGAATATTGCACAATGGGGGAAACCCTGAT | 1507 | 0.3491060383530164 | No Hit |
| CCTACGGGAGGCTGCAGTGGGGAATTTTCCGCAATGGGCGAAAGCCTGAC | 1482 | 0.3433146309483545 | No Hit |
| CCTACGGGGGGCTGCAGTGGGGAATATTGCACAATGGGGGAAACCCTGAT | 1458 | 0.3377548798398792 | No Hit |
| CCTACGGGGGGCTGCAGTGGGGAATATTGGACAATGGGGGCAACCCTGAT | 1457 | 0.3375232235436927 | No Hit |
| CCTACGGGGGGCTGCAGTGGGGAATTTTCCGCAATGGGCGAAAGCCTGAC | 1388 | 0.32153893910682596 | No Hit |
| CCTACGGGAGGCAGCAGTGGGGAATATTGCGCAATGGGCGGAAGCCTGAC | 1273 | 0.2948984650453815 | No Hit |
| CCTACGGGGGGCAGCAGTGGGGAATATTGCGCAATGGGCGGAAGCCTGAC | 1273 | 0.2948984650453815 | No Hit |
| CCTACGGGCGGCTGCAGTGGGGAATATTGGACAATGGGGGCAACCCTGAT | 1262 | 0.2923502457873302 | No Hit |
| CCTACGGGCGGCTGCAGTGGGGAATTTTGGACAATGGGCGCAAGCCTGAT | 1254 | 0.29049699541783847 | No Hit |
| CCTACGGGCGGCTGCAGTGGGGAATATTGCGCAATGGGCGAAAGCCTGAC | 1242 | 0.28771711986360077 | No Hit |
| CCTACGGGTGGCTGCAGTGGGGAATCTTGCGCAATGGGCGAAAGCCTGAC | 1228 | 0.28447393171699015 | No Hit |
| CCTACGGGCGGCTGCAGTGGGGAATTTTCCGCAATGGGCGAAAGCCTGAC | 1215 | 0.28146239986656596 | No Hit |
| CCTACGGGCGGCTGCAGTGGGGAATATTGCACAATGGGGGAAACCCTGAT | 1201 | 0.27821921171995534 | No Hit |
| CCTACGGGGGGCAGCAGTGGGGAATATTGGACAATGGGGGCAACCCTGAT | 1130 | 0.2617716146907157 | No Hit |
| CCTACGGGGGGCAGCAGTGGGGAATTTTGGACAATGGGCGCAAGCCTGAT | 1129 | 0.2615399583945292 | No Hit |
| CCTACGGGAGGCTGCAGTGGGGAATCTTGCGCAATGGGCGAAAGCCTGAC | 1127 | 0.2610766458021563 | No Hit |
| CCTACGGGGGGCAGCAGTGGGGAATATTGCGCAATGGGCGAAAGCCTGAC | 1114 | 0.2580651139517321 | No Hit |
| CCTACGGGGGGCAGCAGTGGGGAATATTGCACAATGGGGGAAACCCTGAT | 1109 | 0.2569068324707997 | No Hit |
| CCTACGGGTGGCAGCAGTGGGGAATATTGCGCAATGGGCGGAAGCCTGAC | 1107 | 0.2564435198784268 | No Hit |
| CCTACGGGGGGCAGCAGTGGGGAATTTTCCGCAATGGGCGAAAGCCTGAC | 1051 | 0.24347076729198422 | No Hit |
| CCTACGGGGGGCTGCAGTGGGGAATCTTGCGCAATGGGCGAAAGCCTGAC | 1051 | 0.24347076729198422 | No Hit |
| CCTACGGGAGGCAGCAGTGGGGAATATTGGACAATGGGGGCAACCCTGAT | 1050 | 0.24323911099579776 | No Hit |
| CCTACGGGTGGCTGCAGTGGGGAATATTGGACAATGGGCGGAAGCCTGAT | 1035 | 0.23976426655300062 | No Hit |
| CCTACGGGAGGCAGCAGTGGGGAATATTGCACAATGGGGGAAACCCTGAT | 1031 | 0.23883764136825475 | No Hit |
| CCTACGGGAGGCAGCAGTGGGGAATTTTGGACAATGGGCGCAAGCCTGAT | 1015 | 0.23513114062927118 | No Hit |
| CCTACGGGGGGCTGCAGTGGGGAATATTGGACAATGGGCGGAAGCCTGAT | 1015 | 0.23513114062927118 | No Hit |
| CCTACGGGAGGCTGCAGTGGGGAATATTGGACAATGGGCGGAAGCCTGAT | 1004 | 0.23258292137121997 | No Hit |
| CCTACGGGAGGCAGCAGTGGGGAATATTGCGCAATGGGCGAAAGCCTGAC | 991 | 0.22957138952079575 | No Hit |
| CCTACGGGTGGCAGCAGTGGGGAATATTGCGCAATGGGCGAAAGCCTGAC | 982 | 0.22748648285511752 | No Hit |
| CCTACGGGAGGCAGCAGTGGGGAATTTTCCGCAATGGGCGAAAGCCTGAC | 968 | 0.22424329470850687 | No Hit |
| CCTACGGGCGGCAGCAGTGGGGAATATTGCGCAATGGGCGGAAGCCTGAC | 945 | 0.21891519989621797 | No Hit |
| CCTACGGGTGGCAGCAGTGGGGAATTTTGGACAATGGGCGCAAGCCTGAT | 927 | 0.21474538656486145 | No Hit |
| CCTACGGGTGGCAGCAGTGGGGAATATTGGACAATGGGGGCAACCCTGAT | 916 | 0.21219716730681024 | No Hit |
| CCTACGGGTGGCAGCAGTGGGGAATTTTCCGCAATGGGCGAAAGCCTGAC | 911 | 0.21103888582587788 | No Hit |
| CCTACGGGCGGCTGCAGTGGGGAATCTTGCGCAATGGGCGAAAGCCTGAC | 892 | 0.20663741619833484 | No Hit |
| CCTACGGGTGGCAGCAGTGGGGAATATTGCACAATGGGGGAAACCCTGAT | 871 | 0.2017726339784189 | No Hit |
| CCTACGGGAGGCAGCAGTGGGGAATCTTGCGCAATGGGCGAAAGCCTGAC | 865 | 0.20038269620130006 | No Hit |
| CCTACGGGAGGCAGCAGTAGGGAATCTTCCGCAATGGACGCAAGTCTGAC | 858 | 0.19876110212799472 | No Hit |
| CCTACGGGGGGCAGCAGTAGGGAATCTTCCGCAATGGACGCAAGTCTGAC | 853 | 0.19760282064706236 | No Hit |
| CCTACGGGCGGCTGCAGTGGGGAATATTGGACAATGGGCGGAAGCCTGAT | 844 | 0.1955179139813841 | No Hit |
| CCTACGGGTGGCTGCAGTAGGGAATCTTCCGCAATGGACGCAAGTCTGAC | 835 | 0.19343300731570584 | No Hit |
| CCTACGGGGGGCTGCAGTAGGGAATCTTCCGCAATGGACGCAAGTCTGAC | 832 | 0.1927380384271464 | No Hit |
| CCTACGGGAGGCTGCAGTAGGGAATCTTCCGCAATGGACGCAAGTCTGAC | 828 | 0.19181141324240053 | No Hit |
| CCTACGGGGGGCAGCAGTGGGGAATCTTGCGCAATGGGCGAAAGCCTGAC | 794 | 0.18393509917206038 | No Hit |
| CCTACGGGCGGCAGCAGTGGGGAATATTGCGCAATGGGCGAAAGCCTGAC | 756 | 0.1751321599169744 | No Hit |
| CCTACGGGGGGCAGCAGTGGGGAATATTGGACAATGGGCGGAAGCCTGAT | 739 | 0.17119400288180434 | No Hit |
| CCTACGGGTGGCAGCAGTGGGGAATCTTGCGCAATGGGCGAAAGCCTGAC | 734 | 0.17003572140087195 | No Hit |
| CCTACGGGCGGCAGCAGTGGGGAATATTGGACAATGGGGGCAACCCTGAT | 723 | 0.16748750214282074 | No Hit |
| CCTACGGGCGGCTGCAGTAGGGAATCTTCCGCAATGGACGCAAGTCTGAC | 719 | 0.16656087695807484 | No Hit |
| CCTACGGGCGGCAGCAGTGGGGAATATTGCACAATGGGGGAAACCCTGAT | 708 | 0.16401265770002363 | No Hit |
| CCTACGGGTGGCAGCAGTAGGGAATCTTCCGCAATGGACGCAAGTCTGAC | 707 | 0.16378100140383717 | No Hit |
| CCTACGGGTGGCTGCAGTGGGGAATCTTGGACAATGGGGGCAACCCTGAT | 697 | 0.16146443844197242 | No Hit |
| CCTACGGGTGGCTGCAGTAGGGAATCTTCCACAATGGACGAAAGTCTGAT | 691 | 0.16007450066485357 | No Hit |
| CCTACGGGAGGCTGCAGTAGGGAATCTTCCACAATGGACGAAAGTCTGAT | 682 | 0.1579895939991753 | No Hit |
| CCTACGGGCGGCAGCAGTGGGGAATTTTCCGCAATGGGCGAAAGCCTGAC | 679 | 0.15729462511061587 | No Hit |
| CCTACGGGCGGCAGCAGTGGGGAATTTTGGACAATGGGCGCAAGCCTGAT | 669 | 0.15497806214875115 | No Hit |
| CCTACGGGGGGCAGCAGTAGGGAATCTTCCACAATGGACGAAAGTCTGAT | 657 | 0.15219818659451345 | No Hit |
| CCTACGGGAGGCAGCAGTGGGGAATATTGGACAATGGGCGGAAGCCTGAT | 653 | 0.15127156140976755 | No Hit |
| CCTACGGGAGGCTGCAGTGGGGAATCTTGGACAATGGGGGCAACCCTGAT | 646 | 0.14964996733646224 | No Hit |
| CCTACGGGTGGCTGCAGTGGGGAATATTGGACAATGGGGGGAACCCTGAT | 625 | 0.14478518511654628 | No Hit |
| CCTACGGGTGGCTGCAGTGGGGAATCTTGCGCAATGCGCGAAAGCGTGAC | 613 | 0.14200530956230858 | No Hit |
| CCTACGGGTGGCAGCAGTAGGGAATCTTCCACAATGGACGAAAGTCTGAT | 607 | 0.14061537178518976 | No Hit |
| CCTACGGGGGGCTGCAGTGGGGAATCTTGGACAATGGGGGCAACCCTGAT | 605 | 0.1401520591928168 | No Hit |
| CCTACGGGTGGCAGCAGTGGGGAATATTGGACAATGGGCGGAAGCCTGAT | 602 | 0.13945709030425738 | No Hit |
| CCTACGGGAGGCTGCAGTGGGGAATATTGGACAATGGGGGGAACCCTGAT | 596 | 0.13806715252713853 | No Hit |
| CCTACGGGCGGCAGCAGTAGGGAATCTTCCGCAATGGACGCAAGTCTGAC | 590 | 0.1366772147500197 | No Hit |
| CCTACGGGGGGCTGCAGTAGGGAATCTTCCACAATGGACGAAAGTCTGAT | 586 | 0.1357505895652738 | No Hit |
| CCTACGGGGGGCTGCAGTGGGGAATATTGGACAATGGGGGGAACCCTGAT | 574 | 0.1329707140110361 | No Hit |
| CCTACGGGCGGCAGCAGTGGGGAATCTTGCGCAATGGGCGAAAGCCTGAC | 571 | 0.1322757451224767 | No Hit |
| CCTACGGGGGGCTGCAGTGGGGAATCTTGCGCAATGCGCGAAAGCGTGAC | 571 | 0.1322757451224767 | No Hit |
| CCTACGGGAGGCAGCAGTAGGGAATCTTCCACAATGGACGAAAGTCTGAT | 561 | 0.12995918216061195 | No Hit |
| CCTACGGGTGGCTGCAGTGGGGAATTTTGGACAATGGGGGCAACCCTGAT | 546 | 0.12648433771781484 | No Hit |
| CCTACGGGAGGCTGCAGTGGGGAATTTTGGACAATGGGGGCAACCCTGAT | 544 | 0.1260210251254419 | No Hit |
| CCTACGGGTGGCTGCAGTGGGGAATCTTAGACAATGGGCGCAAGCCTGAT | 540 | 0.125094399940696 | No Hit |
| CCTACGGGAGGCTGCAGTGGGGAATCTTAGACAATGGGCGCAAGCCTGAT | 536 | 0.12416777475595009 | No Hit |
| CCTACGGGCGGCTGCAGTAGGGAATCTTCCACAATGGACGAAAGTCTGAT | 535 | 0.12393611845976363 | No Hit |
| CCTACGGGAGGCTGCAGTGGGGAATCTTGCGCAATGCGCGAAAGCGTGAC | 525 | 0.12161955549789888 | No Hit |
| CCTACGGGCGGCTGCAGTGGGGAATCTTGGACAATGGGGGCAACCCTGAT | 499 | 0.11559649179705056 | No Hit |
| CCTACGGGCGGCTGCAGTGGGGAATATTGGACAATGGGGGGAACCCTGAT | 495 | 0.11466986661230465 | No Hit |
| CCTACGGGCGGCAGCAGTGGGGAATATTGGACAATGGGCGGAAGCCTGAT | 489 | 0.11327992883518581 | No Hit |
| CCTACGGGGGGCTGCAGTGGGGAATCTTAGACAATGGGCGCAAGCCTGAT | 489 | 0.11327992883518581 | No Hit |
| CCTACGGGGGGCTGCAGTGGGGAATCTTGGACAATGGGGGAAACCCTGAT | 460 | 0.10656189624577807 | No Hit |
| CCTACGGGTGGCTGCAGTGGGGAATTTTGGACAATGGGCGAAAGCCTGAT | 455 | 0.1054036147648457 | No Hit |
| CCTACGGGAGGCTGCAGTGGGGAATCTTGGACAATGGGGGAAACCCTGAT | 455 | 0.1054036147648457 | No Hit |
| CCTACGGGAGGCTGCAGTGGGGAATTTTGGACAATGGGCGAAAGCCTGAT | 451 | 0.1044769895800998 | No Hit |
| CCTACGGGTGGCTGCAGTGGGGAATCTTGGACAATGGGGGAAACCCTGAT | 443 | 0.102623739210608 | No Hit |
| CCTACGGGCGGCAGCAGTAGGGAATCTTCCACAATGGACGAAAGTCTGAT | 440 | 0.10192877032204857 | No Hit |
| CCTACGGGGGGCAGCAGTGGGGAATATTGGACAATGGGGGGAACCCTGAT | 440 | 0.10192877032204857 | No Hit |
| CCTACGGGGGGCAGCAGTGGGGAATCTTGGACAATGGGGGCAACCCTGAT | 437 | 0.10123380143348916 | No Hit |

## Adapter Content

## Kmer Content

| Sequence | Count | PValue | Obs/Exp Max | Max Obs/Exp Position |
| --- | --- | --- | --- | --- |
| ATGTGAT | 655 | 0.0 | 315.99252 | 295 |
| GAGACAT | 10 | 6.888197E-4 | 315.99252 | 295 |
| GCAATAG | 20 | 4.3855835E-8 | 315.99252 | 295 |
| GAGAGAG | 3860 | 0.0 | 313.12732 | 295 |
| AGGTATG | 685 | 0.0 | 304.45996 | 295 |
| ATCTGAT | 115 | 0.0 | 302.25372 | 295 |
| CGGAGAG | 215 | 0.0 | 301.2952 | 295 |
| AGTGTTG | 755 | 0.0 | 299.2512 | 295 |
| ATTTGTG | 345 | 0.0 | 297.67413 | 295 |
| CTCGGTC | 25 | 5.4205884E-10 | 293.9942 | 1 |
| CCTACGG | 42295 | 0.0 | 292.98633 | 1 |
| CTACGGG | 42790 | 0.0 | 292.41397 | 2 |
| CGGCAGC | 3270 | 0.0 | 292.19608 | 9 |
| GGGTGGC | 11335 | 0.0 | 292.04895 | 6 |
| GGGCAGC | 4910 | 0.0 | 291.59912 | 9 |
| TGGCTGC | 6925 | 0.0 | 291.2347 | 9 |
| GGGCGGC | 8465 | 0.0 | 290.34753 | 6 |
| CGGCTGC | 5205 | 0.0 | 290.04037 | 9 |
| GGGAGGC | 11885 | 0.0 | 290.03635 | 6 |
| GGGGCAG | 4940 | 0.0 | 289.53073 | 8 |

Produced by FastQC (version 0.11.7)
