# Supplementary material for: Assessment of airborne bacteria from a public health institution in Mexico City
Source: PLOS Glob Public Health. 2024 Nov 7;4(11):e0003672. doi: 10.1371/journal.pgph.0003672 (PMC11542838; doi:10.1371/journal.pgph.0003672)
Supplement: S1 Text — (ZIP) [file pgph.0003672.s001.zip › Hospital_16S_QC/21022023_CP2D3_16S_S39_L001_R2_001_fastqc.html]

21022023\_CP2D3\_16S\_S39\_L001\_R2\_001.fastq.gz FastQC Report 

FastQC Report

Wed 15 Mar 2023  
21022023\_CP2D3\_16S\_S39\_L001\_R2\_001.fastq.gz

## Summary

- Basic Statistics
- Per base sequence quality
- Per tile sequence quality
- Per sequence quality scores
- Per base sequence content
- Per sequence GC content
- Per base N content
- Sequence Length Distribution
- Sequence Duplication Levels
- Overrepresented sequences
- Adapter Content
- Kmer Content

## Basic Statistics

| Measure | Value |
| --- | --- |
| Filename | 21022023\_CP2D3\_16S\_S39\_L001\_R2\_001.fastq.gz |
| File type | Conventional base calls |
| Encoding | Sanger / Illumina 1.9 |
| Total Sequences | 431674 |
| Sequences flagged as poor quality | 0 |
| Sequence length | 35-301 |
| %GC | 55 |

## Per base sequence quality

## Per tile sequence quality

## Per sequence quality scores

## Per base sequence content

## Per sequence GC content

## Per base N content

## Sequence Length Distribution

## Sequence Duplication Levels

## Overrepresented sequences

| Sequence | Count | Percentage | Possible Source |
| --- | --- | --- | --- |
| GACTACTGGGGTATCTAATCCTGTTCGCTCCCCATGCTTTCGCTCCTCAG | 10668 | 2.4713093677173052 | No Hit |
| GACTACTAGGGTATCTAATCCTGTTCGCTCCCCATGCTTTCGCTCCTCAG | 9225 | 2.137029332320223 | No Hit |
| GACTACAGGGGTATCTAATCCTGTTCGCTCCCCATGCTTTCGCTCCTCAG | 9211 | 2.133786144173613 | No Hit |
| GACTACCGGGGTATCTAATCCTGTTCGCTCCCCATGCTTTCGCTCCTCAG | 9059 | 2.0985743871532683 | No Hit |
| GACTACTCGGGTATCTAATCCTGTTCGCTCCCCATGCTTTCGCTCCTCAG | 8621 | 1.9971089294235929 | No Hit |
| GACTACCAGGGTATCTAATCCTGTTCGCTCCCCATGCTTTCGCTCCTCAG | 8182 | 1.8954118153977306 | No Hit |
| GACTACAAGGGTATCTAATCCTGTTCGCTCCCCATGCTTTCGCTCCTCAG | 8179 | 1.8947168465091713 | No Hit |
| GACTACTGGGGTATCTAATCCTGTTTGCTCCCCACGCTTTCGCACCTCAG | 7753 | 1.7960312643337333 | No Hit |
| GACTACACGGGTATCTAATCCTGTTCGCTCCCCATGCTTTCGCTCCTCAG | 7294 | 1.6897010243841417 | No Hit |
| GACTACCCGGGTATCTAATCCTGTTCGCTCCCCATGCTTTCGCTCCTCAG | 7247 | 1.6788131784633775 | No Hit |
| GACTACAGGGGTATCTAATCCTGTTTGCTCCCCACGCTTTCGCACCTCAG | 6596 | 1.528004929645983 | No Hit |
| GACTACTAGGGTATCTAATCCTGTTTGCTCCCCACGCTTTCGCACCTCAG | 6548 | 1.516885427429032 | No Hit |
| GACTACCGGGGTATCTAATCCTGTTTGCTCCCCACGCTTTCGCACCTCAG | 6407 | 1.4842218896667392 | No Hit |
| GACTACTCGGGTATCTAATCCTGTTTGCTCCCCACGCTTTCGCACCTCAG | 6077 | 1.4077753119252028 | No Hit |
| GACTACAAGGGTATCTAATCCTGTTTGCTCCCCACGCTTTCGCACCTCAG | 5824 | 1.349166268990025 | No Hit |
| GACTACCAGGGTATCTAATCCTGTTTGCTCCCCACGCTTTCGCACCTCAG | 5823 | 1.3489346126938384 | No Hit |
| GACTACACGGGTATCTAATCCTGTTTGCTCCCCACGCTTTCGCACCTCAG | 5337 | 1.236349652747212 | No Hit |
| GACTACTGGGGTATCTAATCCTGTTCGCTCCCCACGCTTTCGCTCCTCAG | 5316 | 1.231484870527296 | No Hit |
| GACTACCCGGGTATCTAATCCTGTTTGCTCCCCACGCTTTCGCACCTCAG | 5308 | 1.2296316201578041 | No Hit |
| GACTACTGGGGTATCTAATCCTGTTTGCTCCCCACGCTTTCGCGCCTCAG | 5023 | 1.1636095757446592 | No Hit |
| GACTACTAGGGTATCTAATCCTGTTCGCTCCCCACGCTTTCGCTCCTCAG | 4535 | 1.0505613032056598 | No Hit |
| GACTACAGGGGTATCTAATCCTGTTCGCTCCCCACGCTTTCGCTCCTCAG | 4528 | 1.0489397091323545 | No Hit |
| GACTACAGGGGTATCTAATCCTGTTTGCTCCCCACGCTTTCGCGCCTCAG | 4367 | 1.011643045446332 | No Hit |
| GACTACTCGGGTATCTAATCCTGTTTGCTCCCCACGCTTTCGCGCCTCAG | 4274 | 0.9900990099009901 | No Hit |
| GACTACCGGGGTATCTAATCCTGTTTGCTCCCCACGCTTTCGCGCCTCAG | 4247 | 0.9838442899039552 | No Hit |
| GACTACTAGGGTATCTAATCCTGTTTGCTCCCCACGCTTTCGCGCCTCAG | 4182 | 0.9687866306518346 | No Hit |
| GACTACTCGGGTATCTAATCCTGTTCGCTCCCCACGCTTTCGCTCCTCAG | 4164 | 0.9646168173204779 | No Hit |
| GACTACCGGGGTATCTAATCCTGTTCGCTCCCCACGCTTTCGCTCCTCAG | 4112 | 0.9525706899187812 | No Hit |
| GACTACAAGGGTATCTAATCCTGTTCGCTCCCCACGCTTTCGCTCCTCAG | 4052 | 0.9386713121475928 | No Hit |
| GACTACTGGGGTATCTAATCCTGTTTGATCCCCACGCTTTCGCACATCAG | 3951 | 0.915274026232759 | No Hit |
| GACTACCAGGGTATCTAATCCTGTTTGCTCCCCACGCTTTCGCGCCTCAG | 3899 | 0.9032278988310624 | No Hit |
| GACTACCAGGGTATCTAATCCTGTTCGCTCCCCACGCTTTCGCTCCTCAG | 3853 | 0.8925717092064845 | No Hit |
| GACTACAAGGGTATCTAATCCTGTTTGCTCCCCACGCTTTCGCGCCTCAG | 3851 | 0.8921083966141117 | No Hit |
| GACTACACGGGTATCTAATCCTGTTCGCTCCCCACGCTTTCGCTCCTCAG | 3532 | 0.8182100381306264 | No Hit |
| GACTACCCGGGTATCTAATCCTGTTCGCTCCCCACGCTTTCGCTCCTCAG | 3512 | 0.8135769122068969 | No Hit |
| GACTACCCGGGTATCTAATCCTGTTTGCTCCCCACGCTTTCGCGCCTCAG | 3511 | 0.8133452559107104 | No Hit |
| GACTACAGGGGTATCTAATCCTGTTTGATCCCCACGCTTTCGCACATCAG | 3467 | 0.8031523788785055 | No Hit |
| GACTACACGGGTATCTAATCCTGTTTGCTCCCCACGCTTTCGCGCCTCAG | 3401 | 0.7878630633301983 | No Hit |
| GACTACCGGGGTATCTAATCCTGTTTGATCCCCACGCTTTCGCACATCAG | 3384 | 0.7839249062950282 | No Hit |
| GACTACTAGGGTATCTAATCCTGTTTGATCCCCACGCTTTCGCACATCAG | 3290 | 0.7621492144534997 | No Hit |
| GACTACTCGGGTATCTAATCCTGTTTGATCCCCACGCTTTCGCACATCAG | 3171 | 0.7345821152073093 | No Hit |
| GACTACAAGGGTATCTAATCCTGTTTGATCCCCACGCTTTCGCACATCAG | 3006 | 0.696358826336541 | No Hit |
| GACTACCAGGGTATCTAATCCTGTTTGATCCCCACGCTTTCGCACATCAG | 2964 | 0.686629261896709 | No Hit |
| GACTACCCGGGTATCTAATCCTGTTTGATCCCCACGCTTTCGCACATCAG | 2646 | 0.6129625597094104 | No Hit |
| GACTACACGGGTATCTAATCCTGTTTGATCCCCACGCTTTCGCACATCAG | 2644 | 0.6124992471170374 | No Hit |
| GACTACTGGGGTATCTAATCCTGTTTGCTCCCCACGCTTTCGTGCATGAG | 2138 | 0.4952811612466816 | No Hit |
| GACTACTGGGGTATCTAATCCTGTTTGCTCCCCATGCTTTCGTACCTCAG | 2041 | 0.4728105005165935 | No Hit |
| GACTACAGGGGTATCTAATCCTGTTTGCTCCCCACGCTTTCGTGCATGAG | 1898 | 0.4396836501619278 | No Hit |
| GACTACTCGGGTATCTAATCCTGTTTGCTCCCCACGCTTTCGTGCATGAG | 1776 | 0.41142158202717793 | No Hit |
| GACTACTAGGGTATCTAATCCTGTTTGCTCCCCACGCTTTCGTGCATGAG | 1731 | 0.40099704869878655 | No Hit |
| GACTACAGGGGTATCTAATCCTGTTTGCTCCCCATGCTTTCGTACCTCAG | 1723 | 0.39914379832929475 | No Hit |
| GACTACCGGGGTATCTAATCCTGTTTGCTCCCCACGCTTTCGTGCATGAG | 1719 | 0.3982171731445489 | No Hit |
| GACTACTAGGGTATCTAATCCTGTTTGCTCCCCATGCTTTCGTACCTCAG | 1650 | 0.3822328887076822 | No Hit |
| GACTACTCGGGTATCTAATCCTGTTTGCTCCCCATGCTTTCGTACCTCAG | 1646 | 0.3813062635229363 | No Hit |
| GACTACCGGGGTATCTAATCCTGTTTGCTCCCCATGCTTTCGTACCTCAG | 1642 | 0.3803796383381904 | No Hit |
| GACTACCAGGGTATCTAATCCTGTTTGCTCCCCATGCTTTCGTACCTCAG | 1565 | 0.36254210353183186 | No Hit |
| GACTACAAGGGTATCTAATCCTGTTTGCTCCCCACGCTTTCGTGCATGAG | 1558 | 0.36092050945852655 | No Hit |
| GACTACCAGGGTATCTAATCCTGTTTGCTCCCCACGCTTTCGTGCATGAG | 1555 | 0.3602255405699672 | No Hit |
| GACTACAAGGGTATCTAATCCTGTTTGCTCCCCATGCTTTCGTACCTCAG | 1475 | 0.3416930368750492 | No Hit |
| GACTACACGGGTATCTAATCCTGTTTGCTCCCCACGCTTTCGTGCATGAG | 1463 | 0.33891316132081156 | No Hit |
| GACTACCCGGGTATCTAATCCTGTTTGCTCCCCACGCTTTCGTGCATGAG | 1384 | 0.3206123139220801 | No Hit |
| GACTACTGGGGTATCTAATCCTGTTCGCTCCCCATGCTTTCGCTTCTCAG | 1364 | 0.3159791879983506 | No Hit |
| GACTACCCGGGTATCTAATCCTGTTTGCTCCCCATGCTTTCGTACCTCAG | 1359 | 0.31482090651741823 | No Hit |
| GACTACACGGGTATCTAATCCTGTTTGCTCCCCATGCTTTCGTACCTCAG | 1358 | 0.31458925022123174 | No Hit |
| GACTACTGGGGTATCTAATCCTGTTTGCTCCCCACGCTTTCGAGCCTCAG | 1343 | 0.31111440577843463 | No Hit |
| GACTACTGGGGTATCTAATCCTGTTCGCTCCCCACACTTTCGCTCCTCAG | 1205 | 0.27914583690470124 | No Hit |
| GACTACAGGGGTATCTAATCCTGTTTGCTCCCCACGCTTTCGAGCCTCAG | 1174 | 0.27196449172292053 | No Hit |
| GACTACTGGGGTATCTAATCCTGTTTGCTCCCCACGCTGTCGCGCCTCAG | 1100 | 0.2548219258051215 | No Hit |
| GACTACTAGGGTATCTAATCCTGTTTGCTCCCCACGCTTTCGAGCCTCAG | 1099 | 0.254590269508935 | No Hit |
| GACTACTCGGGTATCTAATCCTGTTTGCTCCCCACGCTTTCGAGCCTCAG | 1095 | 0.2536636443241891 | No Hit |
| GACTACCGGGGTATCTAATCCTGTTCGCTCCCCATGCTTTCGCTTCTCAG | 1087 | 0.2518103939546973 | No Hit |
| GACTACTAGGGTATCTAATCCTGTTCGCTCCCCATGCTTTCGCTTCTCAG | 1086 | 0.25157873765851085 | No Hit |
| GACTACAGGGGTATCTAATCCTGTTCGCTCCCCATGCTTTCGCTTCTCAG | 1051 | 0.24347076729198422 | No Hit |
| GACTACTGGGGTATCTAATCCTGTTTGCTCCCCACGCTTTCGTGCCTCAG | 1027 | 0.23791101618350885 | No Hit |
| GACTACCGGGGTATCTAATCCTGTTTGCTCCCCACGCTTTCGAGCCTCAG | 1021 | 0.23652107840639003 | No Hit |
| GACTACCAGGGTATCTAATCCTGTTTGCTCCCCACGCTTTCGAGCCTCAG | 1009 | 0.23374120285215233 | No Hit |
| GACTACAGGGGTATCTAATCCTGTTCGCTCCCCACACTTTCGCTCCTCAG | 1008 | 0.2335095465559658 | No Hit |
| GACTACAGGGGTATCTAATCCTGTTTGCTCCCCACGCTGTCGCGCCTCAG | 1007 | 0.23327789025977935 | No Hit |
| GACTACTCGGGTATCTAATCCTGTTCGCTCCCCATGCTTTCGCTTCTCAG | 993 | 0.23003470211316873 | No Hit |
| GACTACCGGGGTATCTAATCCTGTTTGCTCCCCACGCTGTCGCGCCTCAG | 975 | 0.2258648887818122 | No Hit |
| GACTACCAGGGTATCTAATCCTGTTCGCTCCCCATGCTTTCGCTTCTCAG | 966 | 0.22377998211613395 | No Hit |
| GACTACTAGGGTATCTAATCCTGTTTGCTCCCCACGCTGTCGCGCCTCAG | 956 | 0.2214634191542692 | No Hit |
| GACTACCGGGGTATCTAATCCTGTTCGCTCCCCACACTTTCGCTCCTCAG | 948 | 0.2196101687847774 | No Hit |
| GACTACTAGGGTATCTAATCCTGTTCGCTCCCCACACTTTCGCTCCTCAG | 941 | 0.21798857471147207 | No Hit |
| GACTACAAGGGTATCTAATCCTGTTCGCTCCCCATGCTTTCGCTTCTCAG | 936 | 0.2168302932305397 | No Hit |
| GACTACAAGGGTATCTAATCCTGTTTGCTCCCCACGCTTTCGAGCCTCAG | 935 | 0.21659863693435322 | No Hit |
| GACTACCCGGGTATCTAATCCTGTTCGCTCCCCATGCTTTCGCTTCTCAG | 922 | 0.21358710508392909 | No Hit |
| GACTACTCGGGTATCTAATCCTGTTTGCTCCCCACGCTGTCGCGCCTCAG | 917 | 0.2124288236029967 | No Hit |
| GACTACCAGGGTATCTAATCCTGTTCGCTCCCCACACTTTCGCTCCTCAG | 906 | 0.2098806043449455 | No Hit |
| GACTACTGGGGTATCTAATCCTGTTCGCTACCCATGCTTTCGCTCCTCAG | 898 | 0.20802735397545372 | No Hit |
| GACTACTCGGGTATCTAATCCTGTTCGCTCCCCACACTTTCGCTCCTCAG | 891 | 0.20640575990214838 | No Hit |
| GACTACAAGGGTATCTAATCCTGTTTGCTCCCCACGCTGTCGCGCCTCAG | 884 | 0.20478416582884307 | No Hit |
| GACTACACGGGTATCTAATCCTGTTTGCTCCCCACGCTTTCGAGCCTCAG | 877 | 0.20316257175553776 | No Hit |
| GACTACCGGGGTATCTAATCCTGTTTGCTCCCCACGCTTTCGTGCCTCAG | 852 | 0.19737116435087587 | No Hit |
| GACTACTGGGGTATCTAATCCTGTTTGCTACCCACGCTTTCGAATCTCAG | 836 | 0.1936646636118923 | No Hit |
| GACTACAGGGGTATCTAATCCTGTTTGCTCCCCACGCTTTCGTGCCTCAG | 834 | 0.19320135101951935 | No Hit |
| GACTACTAGGGTATCTAATCCTGTTTGCTCCCCACGCTTTCGTGCCTCAG | 826 | 0.19134810065002755 | No Hit |
| GACTACAGGGGTATCTAATCCTGTTCGCTACCCATGCTTTCGCTCCTCAG | 824 | 0.1908847880576546 | No Hit |
| GACTACAAGGGTATCTAATCCTGTTCGCTCCCCACACTTTCGCTCCTCAG | 819 | 0.18972650657672227 | No Hit |
| GACTACCAGGGTATCTAATCCTGTTTGCTCCCCACGCTGTCGCGCCTCAG | 817 | 0.1892631939843493 | No Hit |
| GACTACCCGGGTATCTAATCCTGTTTGCTCCCCACGCTTTCGAGCCTCAG | 810 | 0.18764159991104398 | No Hit |
| GACTACACGGGTATCTAATCCTGTTTGCTCCCCACGCTGTCGCGCCTCAG | 810 | 0.18764159991104398 | No Hit |
| GACTACACGGGTATCTAATCCTGTTCGCTCCCCATGCTTTCGCTTCTCAG | 797 | 0.18463006806061982 | No Hit |
| GACTACCCGGGTATCTAATCCTGTTTGCTCCCCACGCTGTCGCGCCTCAG | 795 | 0.18416675546824687 | No Hit |
| GACTACTCGGGTATCTAATCCTGTTTGCTCCCCACGCTTTCGTGCCTCAG | 791 | 0.18324013028350097 | No Hit |
| GACTACCCGGGTATCTAATCCTGTTCGCTCCCCACACTTTCGCTCCTCAG | 788 | 0.18254516139494154 | No Hit |
| GACTACTCGGGTATCTAATCCTGTTCGCTACCCATGCTTTCGCTCCTCAG | 787 | 0.18231350509875507 | No Hit |
| GACTACTGGGGTATCTAATCCCATTTGCTCCCCTAGCTTTCGTCTCTCAG | 782 | 0.1811552236178227 | No Hit |
| GACTACCAGGGTATCTAATCCTGTTTGCTCCCCACGCTTTCGTGCCTCAG | 777 | 0.17999694213689033 | No Hit |
| GACTACCGGGGTATCTAATCCTGTTCGCTACCCATGCTTTCGCTCCTCAG | 769 | 0.17814369176739853 | No Hit |
| GACTACACGGGTATCTAATCCTGTTCGCTCCCCACACTTTCGCTCCTCAG | 768 | 0.17791203547121207 | No Hit |
| GACTACTAGGGTATCTAATCCTGTTCGCTACCCATGCTTTCGCTCCTCAG | 766 | 0.17744872287883912 | No Hit |
| GACTACTAGGGTATCTAATCCTGTTTGCTACCCACGCTTTCGAATCTCAG | 761 | 0.17629044139790676 | No Hit |
| GACTACTCGGGTATCTAATCCTGTTTGCTACCCACGCTTTCGAATCTCAG | 756 | 0.1751321599169744 | No Hit |
| GACTACAAGGGTATCTAATCCTGTTTGCTCCCCACGCTTTCGTGCCTCAG | 738 | 0.17096234658561785 | No Hit |
| GACTACAGGGGTATCTAATCCTGTTTGCTACCCACGCTTTCGAATCTCAG | 735 | 0.17026737769705844 | No Hit |
| GACTACAGGGGTATCTAATCCCATTTGCTCCCCTAGCTTTCGTCTCTCAG | 712 | 0.16493928288476953 | No Hit |
| GACTACCGGGGTATCTAATCCTGTTTGCTACCCACGCTTTCGAATCTCAG | 702 | 0.16262271992290478 | No Hit |
| GACTACTAGGGTATCTAATCCCATTTGCTCCCCTAGCTTTCGTCTCTCAG | 682 | 0.1579895939991753 | No Hit |
| GACTACACGGGTATCTAATCCTGTTCGCTACCCATGCTTTCGCTCCTCAG | 664 | 0.15381978066781876 | No Hit |
| GACTACAAGGGTATCTAATCCTGTTTGCTACCCACGCTTTCGAATCTCAG | 656 | 0.151966530298327 | No Hit |
| GACTACCAGGGTATCTAATCCTGTTTGCTACCCACGCTTTCGAATCTCAG | 656 | 0.151966530298327 | No Hit |
| GACTACCCGGGTATCTAATCCTGTTTGCTCCCCACGCTTTCGTGCCTCAG | 651 | 0.1508082488173946 | No Hit |
| GACTACCAGGGTATCTAATCCTGTTCGCTACCCATGCTTTCGCTCCTCAG | 651 | 0.1508082488173946 | No Hit |
| GACTACAAGGGTATCTAATCCTGTTCGCTACCCATGCTTTCGCTCCTCAG | 648 | 0.1501132799288352 | No Hit |
| GACTACTGGGGTATCTAATCCTGTTCGCTCCCCACGCTTTCGTGCCTCAG | 648 | 0.1501132799288352 | No Hit |
| GACTACCGGGGTATCTAATCCCATTTGCTCCCCTAGCTTTCGTCTCTCAG | 641 | 0.14849168585552985 | No Hit |
| GACTACACGGGTATCTAATCCTGTTTGCTCCCCACGCTTTCGTGCCTCAG | 632 | 0.1464067791898516 | No Hit |
| GACTACCCGGGTATCTAATCCTGTTCGCTACCCATGCTTTCGCTCCTCAG | 624 | 0.14455352882035982 | No Hit |
| GACTACCAGGGTATCTAATCCCATTTGCTCCCCTAGCTTTCGTCTCTCAG | 615 | 0.14246862215468156 | No Hit |
| GACTACTCGGGTATCTAATCCCATTTGCTCCCCTAGCTTTCGTCTCTCAG | 608 | 0.14084702808137622 | No Hit |
| GACTACACGGGTATCTAATCCTGTTTGCTACCCACGCTTTCGAATCTCAG | 578 | 0.133897339195782 | No Hit |
| GACTACCCGGGTATCTAATCCTGTTTGCTACCCACGCTTTCGAATCTCAG | 574 | 0.1329707140110361 | No Hit |
| GACTACTGGGGTATCTAATCCTGTTTGCTCCCCATGCTTTCGCACCTCAG | 568 | 0.13158077623391726 | No Hit |
| GACTACAGGGGTATCTAATCCTGTTCGCTCCCCACGCTTTCGTGCCTCAG | 567 | 0.13134911993773077 | No Hit |
| GACTACAAGGGTATCTAATCCCATTTGCTCCCCTAGCTTTCGTCTCTCAG | 555 | 0.1285692443834931 | No Hit |
| GACTACCGGGGTATCTAATCCTGTTCGCTCCCCACGCTTTCGTGCCTCAG | 552 | 0.1278742754949337 | No Hit |
| GACTACACGGGTATCTAATCCCATTTGCTCCCCTAGCTTTCGTCTCTCAG | 544 | 0.1260210251254419 | No Hit |
| GACTACTCGGGTATCTAATCCTGTTCGCTCCCCACGCTTTCGTGCCTCAG | 535 | 0.12393611845976363 | No Hit |
| GACTACTAGGGTATCTAATCCTGTTTGCTCCCCATGCTTTCGCACCTCAG | 519 | 0.12022961772078003 | No Hit |
| GACTACTAGGGTATCTAATCCTGTTCGCTCCCCACGCTTTCGTGCCTCAG | 519 | 0.12022961772078003 | No Hit |
| GACTACTGGGGTATCTAATCCTGTTTGCTCCCCACGCTTTCGCACCTGAG | 516 | 0.11953464883222062 | No Hit |
| GACTACCCGGGTATCTAATCCCATTTGCTCCCCTAGCTTTCGTCTCTCAG | 514 | 0.11907133623984766 | No Hit |
| GACTACAGGGGTATCTAATCCTGTTTGCTCCCCATGCTTTCGCACCTCAG | 513 | 0.1188396799436612 | No Hit |
| GACTACCAGGGTATCTAATCCTGTTCGCTCCCCACGCTTTCGTGCCTCAG | 494 | 0.11443821031611819 | No Hit |
| GACTACTGGGGTATCTAATCCTGTTTGCTCCCCACGCTTTCGCGTCTCAG | 483 | 0.11188999105806698 | No Hit |
| GACTACTCGGGTATCTAATCCTGTTTGCTCCCCATGCTTTCGCACCTCAG | 475 | 0.11003674068857518 | No Hit |
| GACTACAAGGGTATCTAATCCTGTTCGCTCCCCACGCTTTCGTGCCTCAG | 458 | 0.10609858365340512 | No Hit |
| GACTACCGGGGTATCTAATCCTGTTTGCTCCCCATGCTTTCGCACCTCAG | 455 | 0.1054036147648457 | No Hit |
| GACTACAGGGGTATCTAATCCTGTTTGCTCCCCACGCTTTCGCGTCTCAG | 448 | 0.10378202069154037 | No Hit |
| GACTACTAGGGTATCTAATCCTGTTTGCTCCCCACGCTTTCGCGTCTCAG | 446 | 0.10331870809916742 | No Hit |
| GACTACCCGGGTATCTAATCCTGTTCGCTCCCCACGCTTTCGTGCCTCAG | 446 | 0.10331870809916742 | No Hit |

## Adapter Content

## Kmer Content

| Sequence | Count | PValue | Obs/Exp Max | Max Obs/Exp Position |
| --- | --- | --- | --- | --- |
| GCAAGGG | 5 | 2.866E-4 | 5340.381 | 295 |
| TTATGCG | 5 | 2.866E-4 | 5340.381 | 295 |
| TGTGGCG | 5 | 2.866E-4 | 5340.381 | 295 |
| GTTAGAA | 5 | 2.866E-4 | 5340.381 | 295 |
| GTAGGCG | 10 | 1.424869E-7 | 5340.381 | 295 |
| AGTGAGG | 5 | 2.866E-4 | 5340.381 | 295 |
| TTTTGCG | 5 | 2.866E-4 | 5340.381 | 295 |
| GATAGCA | 5 | 2.866E-4 | 5340.381 | 295 |
| GTTAGCG | 370 | 0.0 | 5196.0464 | 295 |
| TTAGCCG | 1165 | 0.0 | 4561.0977 | 295 |
| TTAGGCG | 55 | 0.0 | 4369.403 | 295 |
| TTAGACG | 45 | 0.0 | 4153.63 | 295 |
| GTTAGCA | 45 | 0.0 | 4153.63 | 295 |
| GTTAGGG | 20 | 2.1282176E-10 | 4005.2856 | 295 |
| GTTGGCG | 80 | 0.0 | 4005.2856 | 295 |
| TTTGCCG | 50 | 0.0 | 3738.267 | 295 |
| GTTTGCG | 25 | 5.18412E-10 | 3204.2288 | 295 |
| GTTTGGG | 10 | 0.0011462575 | 2670.1904 | 295 |
| GTTTGAG | 10 | 0.0011462575 | 2670.1904 | 295 |
| AGGTAGG | 10 | 0.0011462575 | 2670.1904 | 295 |

Produced by FastQC (version 0.11.7)
