# Supplementary material for: Assessment of airborne bacteria from a public health institution in Mexico City
Source: PLOS Glob Public Health. 2024 Nov 7;4(11):e0003672. doi: 10.1371/journal.pgph.0003672 (PMC11542838; doi:10.1371/journal.pgph.0003672)
Supplement: S1 Text — (ZIP) [file pgph.0003672.s001.zip › Hospital_16S_QC/21022023_CUD1_16S_S43_L001_R1_001_fastqc.html]

21022023\_CUD1\_16S\_S43\_L001\_R1\_001.fastq.gz FastQC Report 

FastQC Report

Wed 15 Mar 2023  
21022023\_CUD1\_16S\_S43\_L001\_R1\_001.fastq.gz

## Summary

- Basic Statistics
- Per base sequence quality
- Per tile sequence quality
- Per sequence quality scores
- Per base sequence content
- Per sequence GC content
- Per base N content
- Sequence Length Distribution
- Sequence Duplication Levels
- Overrepresented sequences
- Adapter Content
- Kmer Content

## Basic Statistics

| Measure | Value |
| --- | --- |
| Filename | 21022023\_CUD1\_16S\_S43\_L001\_R1\_001.fastq.gz |
| File type | Conventional base calls |
| Encoding | Sanger / Illumina 1.9 |
| Total Sequences | 25146 |
| Sequences flagged as poor quality | 0 |
| Sequence length | 80-301 |
| %GC | 54 |

## Per base sequence quality

## Per tile sequence quality

## Per sequence quality scores

## Per base sequence content

## Per sequence GC content

## Per base N content

## Sequence Length Distribution

## Sequence Duplication Levels

## Overrepresented sequences

| Sequence | Count | Percentage | Possible Source |
| --- | --- | --- | --- |
| CCTACGGGTGGCTGCAGTGGGGAATATTGGACAATGGGCGCAAGCCTGAT | 766 | 3.046210132824306 | No Hit |
| CCTACGGGAGGCTGCAGTGGGGAATATTGGACAATGGGCGCAAGCCTGAT | 710 | 2.8235106975264457 | No Hit |
| CCTACGGGTGGCTGCAGTGGGGAATATTGGACAATGGGCGAAAGCCTGAT | 689 | 2.739998409289748 | No Hit |
| CCTACGGGCGGCTGCAGTGGGGAATATTGGACAATGGGCGCAAGCCTGAT | 627 | 2.493438320209974 | No Hit |
| CCTACGGGTGGCTGCAGTGGGGAATATTGCACAATGGGCGCAAGCCTGAT | 604 | 2.4019724807126384 | No Hit |
| CCTACGGGAGGCTGCAGTGGGGAATATTGCACAATGGGCGCAAGCCTGAT | 562 | 2.2349479042392426 | No Hit |
| CCTACGGGGGGCTGCAGTGGGGAATATTGGACAATGGGCGCAAGCCTGAT | 556 | 2.211087250457329 | No Hit |
| CCTACGGGCGGCTGCAGTGGGGAATATTGGACAATGGGCGAAAGCCTGAT | 533 | 2.1196214109599936 | No Hit |
| CCTACGGGAGGCTGCAGTGGGGAATATTGGACAATGGGCGAAAGCCTGAT | 497 | 1.976457488268512 | No Hit |
| CCTACGGGCGGCTGCAGTGGGGAATATTGCACAATGGGCGCAAGCCTGAT | 477 | 1.8969219756621332 | No Hit |
| CCTACGGGGGGCTGCAGTGGGGAATATTGCACAATGGGCGCAAGCCTGAT | 457 | 1.8173864630557546 | No Hit |
| CCTACGGGAGGCAGCAGTGGGGAATATTGGACAATGGGCGCAAGCCTGAT | 440 | 1.7497812773403325 | No Hit |
| CCTACGGGGGGCTGCAGTGGGGAATATTGGACAATGGGCGAAAGCCTGAT | 418 | 1.6622922134733158 | No Hit |
| CCTACGGGGGGCAGCAGTGGGGAATATTGGACAATGGGCGCAAGCCTGAT | 398 | 1.5827567008669372 | No Hit |
| CCTACGGGAGGCAGCAGTGGGGAATATTGCACAATGGGCGCAAGCCTGAT | 389 | 1.5469657201940668 | No Hit |
| CCTACGGGTGGCAGCAGTGGGGAATATTGGACAATGGGCGCAAGCCTGAT | 364 | 1.4475463294360933 | No Hit |
| CCTACGGGGGGCAGCAGTGGGGAATATTGGACAATGGGCGAAAGCCTGAT | 363 | 1.4435695538057742 | No Hit |
| CCTACGGGCGGCAGCAGTGGGGAATATTGGACAATGGGCGCAAGCCTGAT | 350 | 1.391871470611628 | No Hit |
| CCTACGGGAGGCAGCAGTGGGGAATATTGGACAATGGGCGAAAGCCTGAT | 348 | 1.3839179193509903 | No Hit |
| CCTACGGGTGGCTGCAGTGGGGAATATTGCACAATGGGCGAAAGCCTGAT | 339 | 1.3481269386781198 | No Hit |
| CCTACGGGAGGCTGCAGTGGGGAATATTGCACAATGGGCGAAAGCCTGAT | 336 | 1.336196611787163 | No Hit |
| CCTACGGGTGGCAGCAGTGGGGAATATTGCACAATGGGCGCAAGCCTGAT | 319 | 1.268591426071741 | No Hit |
| CCTACGGGTGGCAGCAGTGGGGAATATTGGACAATGGGCGAAAGCCTGAT | 315 | 1.2526843235504654 | No Hit |
| CCTACGGGCGGCAGCAGTGGGGAATATTGGACAATGGGCGAAAGCCTGAT | 305 | 1.212916567247276 | No Hit |
| CCTACGGGCGGCAGCAGTGGGGAATATTGCACAATGGGCGCAAGCCTGAT | 284 | 1.1294042790105783 | No Hit |
| CCTACGGGGGGCAGCAGTGGGGAATATTGCACAATGGGCGCAAGCCTGAT | 264 | 1.0498687664041995 | No Hit |
| CCTACGGGGGGCTGCAGTGGGGAATATTGCACAATGGGCGAAAGCCTGAT | 262 | 1.0419152151435618 | No Hit |
| CCTACGGGCGGCTGCAGTGGGGAATATTGCACAATGGGCGAAAGCCTGAT | 243 | 0.9663564781675018 | No Hit |
| CCTACGGGTGGCTGCAGTAGGGAATCTTCCGCAATGGGCGAAAGCCTGAC | 231 | 0.9186351706036745 | No Hit |
| CCTACGGGAGGCAGCAGTGGGGAATATTGCACAATGGGCGAAAGCCTGAT | 224 | 0.8907977411914421 | No Hit |
| CCTACGGGGGGCAGCAGTGGGGAATATTGCACAATGGGCGAAAGCCTGAT | 206 | 0.819215779845701 | No Hit |
| CCTACGGGAGGCTGCAGTAGGGAATCTTCCGCAATGGGCGAAAGCCTGAC | 201 | 0.7993319016941064 | No Hit |
| CCTACGGGTGGCAGCAGTGGGGAATATTGCACAATGGGCGAAAGCCTGAT | 196 | 0.7794480235425117 | No Hit |
| CCTACGGGAGGCAGCAGTAGGGAATCTTCCGCAATGGGCGAAAGCCTGAC | 176 | 0.699912510936133 | No Hit |
| CCTACGGGCGGCTGCAGTAGGGAATCTTCCGCAATGGGCGAAAGCCTGAC | 171 | 0.6800286327845383 | No Hit |
| CCTACGGGCGGCAGCAGTGGGGAATATTGCACAATGGGCGAAAGCCTGAT | 171 | 0.6800286327845383 | No Hit |
| CCTACGGGTGGCTGCAGTGGGGAATATTGCACAATGGGCGGAAGCCTGAT | 170 | 0.6760518571542193 | No Hit |
| CCTACGGGGGGCTGCAGTAGGGAATCTTCCGCAATGGGCGAAAGCCTGAC | 168 | 0.6680983058935815 | No Hit |
| CCTACGGGGGGCAGCAGTAGGGAATCTTCCGCAATGGGCGAAAGCCTGAC | 143 | 0.5686789151356081 | No Hit |
| CCTACGGGCGGCTGCAGTGGGGAATATTGCACAATGGGCGGAAGCCTGAT | 142 | 0.5647021395052891 | No Hit |
| CCTACGGGTGGCAGCAGTAGGGAATCTTCCGCAATGGGCGAAAGCCTGAC | 142 | 0.5647021395052891 | No Hit |
| CCTACGGGGGGCTGCAGTGGGGAATATTGCACAATGGGCGGAAGCCTGAT | 140 | 0.5567485882446512 | No Hit |
| CCTACGGGAGGCAGCAGTAGGGAATCTTCCGCAATGGACGAAAGTCTGAC | 131 | 0.5209576075717809 | No Hit |
| CCTACGGGAGGCTGCAGTGGGGAATATTGCACAATGGGCGGAAGCCTGAT | 123 | 0.4891434025292293 | No Hit |
| CCTACGGGCGGCAGCAGTAGGGAATCTTCCGCAATGGGCGAAAGCCTGAC | 119 | 0.4732363000079535 | No Hit |
| CCTACGGGTGGCAGCAGTAGGGAATCTTCCGCAATGGACGAAAGTCTGAC | 115 | 0.4573291974866778 | No Hit |
| CTTGGTCATTTAGAGGAAGTAAAAGTCGTAACAAGGTTTCCGTAGGTGAA | 113 | 0.4493756462260399 | No Hit |
| CCTACGGGGGGCAGCAGTAGGGAATCTTCCGCAATGGACGAAAGTCTGAC | 112 | 0.44539887059572103 | No Hit |
| CCTACGGGTGGCTGCAGTGGGGAATATTGCACAATGGGGGAAACCCTGAT | 111 | 0.441422094965402 | No Hit |
| CCTACGGGAGGCTGCAGTGGGGAATATTGCACAATGGGGGAAACCCTGAT | 107 | 0.42551499244412627 | No Hit |
| CCTACGGGAGGCAGCAGTGGGGAATATTGCACAATGGGCGGAAGCCTGAT | 95 | 0.3777936848802991 | No Hit |
| CCTACGGGGGGCTGCAGTGGGGAATATTGCACAATGGGGGAAACCCTGAT | 94 | 0.3738169092499801 | No Hit |
| CCTACGGGAGGCTGCAGTAGGGAATCTTCCGCAATGGACGAAAGTCTGAC | 93 | 0.36984013361966117 | No Hit |
| CCTACGGGCGGCTGCAGTGGGGAATATTGCACAATGGGGGAAACCCTGAT | 90 | 0.35790980672870437 | No Hit |
| CCTACGGGGGGCAGCAGTGGGGAATATTGCACAATGGGCGGAAGCCTGAT | 88 | 0.3499562554680665 | No Hit |
| CCTACGGGTGGCAGCAGTGGGGAATATTGCACAATGGGCGGAAGCCTGAT | 85 | 0.33802592857710967 | No Hit |
| CCTACGGGTGGCTGCAGTGGGGAATTTTGGACAATGGGCGCAAGCCTGAT | 83 | 0.3300723773164718 | No Hit |
| CCTACGGGTGGCTGCAGTGGGGAATATTGGACAATGGGGGCAACCCTGAT | 78 | 0.3101884991648771 | No Hit |
| CCTACGGGGGGCTGCAGTAGGGAATCTTCCGCAATGGACGAAAGTCTGAC | 77 | 0.30621172353455817 | No Hit |
| CCTACGGGCGGCAGCAGTAGGGAATCTTCCGCAATGGACGAAAGTCTGAC | 76 | 0.3022349479042392 | No Hit |
| CCTACGGGTGGCTGCAGTAGGGAATCTTCCGCAATGGACGAAAGTCTGAC | 75 | 0.29825817227392026 | No Hit |
| CCTACGGGCGGCAGCAGTGGGGAATATTGCACAATGGGCGGAAGCCTGAT | 73 | 0.2903046210132824 | No Hit |
| CCTACGGGAGGCAGCAGTGGGGAATATTGGACAATGGGGGGAACCCTGAT | 72 | 0.28632784538296346 | No Hit |
| CCTACGGGTGGCTGCAGTGGGGAATATTGGACAATGGGGGGAACCCTGAT | 70 | 0.2783742941223256 | No Hit |
| CCTACGGGTGGCTGCAGTGGGGAATCTTAGACAATGGGGGCAACCCTGAT | 68 | 0.2704207428616877 | No Hit |
| CCTACGGGTGGCAGCAGTGGGGAATATTGGACAATGGGGGCAACCCTGAT | 64 | 0.254513640340412 | No Hit |
| CCTACGGGAGGCTGCAGTGGGGAATATTGGACAATGGGGGGAACCCTGAT | 64 | 0.254513640340412 | No Hit |
| CCTACGGGAGGCAGCAGTGGGGAATATTGCACAATGGGGGAAACCCTGAT | 64 | 0.254513640340412 | No Hit |
| CCTACGGGTGGCAGCAGTGGGGAATATTGGACAATGGGGGGAACCCTGAT | 62 | 0.24656008907977411 | No Hit |
| CCTACGGGAGGCTGCAGTGGGGAATTTTGGACAATGGGCGCAAGCCTGAT | 59 | 0.23462976218881731 | No Hit |
| CCTACGGGGGGCAGCAGTAGGGAATCTTCCGCAATGGACGCAAGTCTGAC | 59 | 0.23462976218881731 | No Hit |
| CCTACGGGAGGCTGCAGTGGGGAATATTGGACAATGGGGGCAACCCTGAT | 59 | 0.23462976218881731 | No Hit |
| CCTACGGGGGGCTGCAGTGGGGAATTTTGGACAATGGGCGCAAGCCTGAT | 56 | 0.22269943529786052 | No Hit |
| CCTACGGGTGGCAGCAGTAGGGAATCTTCCGCAATGGACGCAAGTCTGAC | 56 | 0.22269943529786052 | No Hit |
| CCTACGGGCGGCAGCAGTGGGGAATATTGCACAATGGGGGAAACCCTGAT | 56 | 0.22269943529786052 | No Hit |
| CCTACGGGCGGCTGCAGTGGGGAATATTGGACAATGGGGGCAACCCTGAT | 55 | 0.21872265966754156 | No Hit |
| CCTACGGGCGGCTGCAGTGGGGAATTTTGGACAATGGGCGCAAGCCTGAT | 54 | 0.21474588403722264 | No Hit |
| CCTACGGGTGGCAGCAGTAGGGAATCTTCCACAATGGACGAAAGTCTGAT | 54 | 0.21474588403722264 | No Hit |
| CCTACGGGGGGCAGCAGTAGGGAATCTTCCACAATGGACGAAAGTCTGAT | 53 | 0.2107691084069037 | No Hit |
| CCTACGGGCGGCTGCAGTAGGGAATCTTCCGCAATGGACGAAAGTCTGAC | 52 | 0.20679233277658476 | No Hit |
| CCTACGGGGGGCTGCAGTGGGGAATATTGGACAATGGGGGGAACCCTGAT | 51 | 0.2028155571462658 | No Hit |
| CCTACGGGCGGCTGCAGTGGGGAATCTTAGACAATGGGGGCAACCCTGAT | 51 | 0.2028155571462658 | No Hit |
| CCTACGGGGGGCTGCAGTAGGGAATCTTCGGCAATGGACGGAAGTCTGAC | 51 | 0.2028155571462658 | No Hit |
| CCTACGGGCGGCAGCAGTGGGGAATATTGGACAATGGGGGGAACCCTGAT | 50 | 0.1988387815159469 | No Hit |
| CCTACGGGTGGCAGCAGTGGGGAATATTGCACAATGGGGGAAACCCTGAT | 50 | 0.1988387815159469 | No Hit |
| CCTACGGGCGGCTGCAGTGGGGAATATTGGACAATGGGGGGAACCCTGAT | 50 | 0.1988387815159469 | No Hit |
| CCTACGGGTGGCAGCAGTGGGGAATTTTGGACAATGGGCGCAAGCCTGAT | 49 | 0.19486200588562794 | No Hit |
| CCTACGGGAGGCAGCAGTAGGGAATCTTCCGCAATGGACGCAAGTCTGAC | 49 | 0.19486200588562794 | No Hit |
| CCTACGGGGGGCAGCAGTAGGGAATCTTCGGCAATGGACGGAAGTCTGAC | 48 | 0.19088523025530899 | No Hit |
| CCTACGGGGGGCTGCAGTGGGGAATCTTAGACAATGGGGGCAACCCTGAT | 48 | 0.19088523025530899 | No Hit |
| CCTACGGGAGGCTGCAGTGGGGAATCTTAGACAATGGGGGCAACCCTGAT | 46 | 0.1829316789946711 | No Hit |
| CCTACGGGGGGCAGCAGTGGGGAATATTGCACAATGGGGGAAACCCTGAT | 45 | 0.17895490336435219 | No Hit |
| CCTACGGGAGGCAGCAGTGGGGAATATTGGACAATGGGGGCAACCCTGAT | 44 | 0.17497812773403326 | No Hit |
| CTACGTCATTTAGAGGAAGTAAAAGTCGTAACAAGGTTTCCGTAGGTGAA | 43 | 0.1710013521037143 | No Hit |
| CCTACGGGGGGCAGCAGTGGGGAATATTGGACAATGGGGGGAACCCTGAT | 43 | 0.1710013521037143 | No Hit |
| CCTACGGGGGGCTGCAGTGGGGAATATTGGACAATGGGGGCAACCCTGAT | 43 | 0.1710013521037143 | No Hit |
| CCTACGGGCGGCAGCAGTGGGGAATATTGGACAATGGGGGCAACCCTGAT | 42 | 0.1670245764733954 | No Hit |
| CCTACGGGCGGCTGCAGTGGGGAATATTGCGCAATGGGCGAAAGCCTGAC | 41 | 0.16304780084307643 | No Hit |
| CCTACGGGAGGCTGCAGTAGGGAATCTTCGGCAATGGACGGAAGTCTGAC | 41 | 0.16304780084307643 | No Hit |
| CCTACGGGCGGCAGCAGTGGGGAATTTTGGACAATGGGCGCAAGCCTGAT | 41 | 0.16304780084307643 | No Hit |
| CCTACGGGGGGCAGCAGTGGGGAATATTGGACAATGGGGGCAACCCTGAT | 41 | 0.16304780084307643 | No Hit |
| CCTACGGGAGGCAGCAGTAGGGAATCTTCCACAATGGACGAAAGTCTGAT | 41 | 0.16304780084307643 | No Hit |
| CCTACGGGAGGCTGCAGTAGGGAATCTTCCGCAATGGACGCAAGTCTGAC | 40 | 0.1590710252127575 | No Hit |
| CCTACGGGTGGCTGCAGTAGGGAATCTTCGGCAATGGACGGAAGTCTGAC | 39 | 0.15509424958243856 | No Hit |
| CCTACGGGAGGCAGCAGTGGGGAATTTTGGACAATGGGCGCAAGCCTGAT | 38 | 0.1511174739521196 | No Hit |
| CCTACGGGTGGCTGCAGTGAGGAATATTGGTCAATGGGCGAGAGCCTGAA | 38 | 0.1511174739521196 | No Hit |
| CCTACGGGTGGCTGCAGTAGGGAATCTTCCGCAATGGACGCAAGTCTGAC | 37 | 0.14714069832180068 | No Hit |
| CCTACGGGCGGCAGCAGTAGGGAATCTTCCGCAATGGACGCAAGTCTGAC | 36 | 0.14316392269148173 | No Hit |
| CCTACGGGTGGCTGCAGTGGGGAATATTGCGCAATGGGCGAAAGCCTGAC | 36 | 0.14316392269148173 | No Hit |
| CCTACGGGGGGCTGCAGTGGGGAATTTTGGACAATGGGCGAAAGCCTGAT | 34 | 0.13521037143084386 | No Hit |
| CCTACGGGAGGCTGCAGTGGGGAATTTTCCGCAATGGGCGAAAGCCTGAC | 33 | 0.13123359580052493 | No Hit |
| CCTACGGGAGGCAGCAGTGGGGAATCTTAGACAATGGGGGCAACCCTGAT | 33 | 0.13123359580052493 | No Hit |
| CTCGGTCATTTAGAGGAAGTAAAAGTCGTAACAAGGTTTCCGTAGGTGAA | 33 | 0.13123359580052493 | No Hit |
| CTTAGTTATTTAGAGGAAGTAAAAGTCGTAACAAGGTTTCCGTAGGTGAA | 33 | 0.13123359580052493 | No Hit |
| CCTACGGGTGGCTGCAGTGGGGAATCTTGCGCAATGGGCGAAAGCCTGAC | 33 | 0.13123359580052493 | No Hit |
| CCTACGGGTGGCTGCAGTGGGGAATTTTCCGCAATGGGCGAAAGCCTGAC | 33 | 0.13123359580052493 | No Hit |
| CCTACGGGGGGCTGCAGTAGGGAATCTTCCGCAATGGACGCAAGTCTGAC | 32 | 0.127256820170206 | No Hit |
| CCTACGGGAGGCAGCAGTAGGGAATCTTCGGCAATGGACGGAAGTCTGAC | 32 | 0.127256820170206 | No Hit |
| CCTACGGGCGGCTGCAGTAGGGAATCTTCGGCAATGGGGGCAACCCTGAC | 31 | 0.12328004453988706 | No Hit |
| CCTACGGGAGGCTGCAGTGAGGAATATTGGTCAATGGGCGAGAGCCTGAA | 31 | 0.12328004453988706 | No Hit |
| CTTGGTCATTTAGAGGAAGTAAAAGTCGTAACAAGGTCTCCGTAGGTGAA | 31 | 0.12328004453988706 | No Hit |
| CCTACGGGCGGCTGCAGTAGGGAATCTTCGGCAATGGACGGAAGTCTGAC | 31 | 0.12328004453988706 | No Hit |
| CCTACGGGTGGCAGCAGTAGGGAATCTTCGGCAATGGACGGAAGTCTGAC | 30 | 0.11930326890956812 | No Hit |
| CCTACGGGCGGCAGCAGTAGGGAATCTTCCACAATGGACGAAAGTCTGAT | 30 | 0.11930326890956812 | No Hit |
| CCTACGGGAGGCTGCAGTGGGGAATTTTGGACAATGGGCGAAAGCCTGAT | 30 | 0.11930326890956812 | No Hit |
| CCTACGGGGGGCTGCAGTAGGGAATCTTCGGCAATGGGGGCAACCCTGAC | 30 | 0.11930326890956812 | No Hit |
| CCTACGGGAGGCTGCAGTGGGGAATCTTGCGCAATGGGCGAAAGCCTGAC | 30 | 0.11930326890956812 | No Hit |
| CCTACGGGAGGCTGCAGTGGGGAATATTGCGCAATGGGCGAAAGCCTGAC | 30 | 0.11930326890956812 | No Hit |
| CCTACGGGAGGCAGCAGTGGGGAATCTTCCGCAATGGGCGAAAGCCTGAC | 29 | 0.11532649327924918 | No Hit |
| CCTACGGGGGGCTGCAGTGGGGAATATTGCGCAATGGGCGAAAGCCTGAC | 29 | 0.11532649327924918 | No Hit |
| CCTACGGGAGGCTGCAGTAGGGAATCTTCCACAATGGACGAAAGTCTGAT | 29 | 0.11532649327924918 | No Hit |
| CCTACGGGTGGCTGCAGTGGGGAATCTTCCGCAATGGGCGAAAGCCTGAC | 28 | 0.11134971764893026 | No Hit |
| CCTACGGGTGGCAGCAGTGGGGAATCTTAGACAATGGGGGCAACCCTGAT | 28 | 0.11134971764893026 | No Hit |
| CCTACGGGAGGCTGCAGTGGGGAATATTGGACAATGGGCGGAAGCCTGAT | 28 | 0.11134971764893026 | No Hit |
| CCTACGGGTGGCTGCAGTAGGGAATCTTCCACAATGGACGAAAGTCTGAT | 28 | 0.11134971764893026 | No Hit |
| CCTACGGGTGGCAGCAGTGGGGAATCTTCCGCAATGGGCGAAAGCCTGAC | 28 | 0.11134971764893026 | No Hit |
| CCTACGGGCGGCTGCAGTGGGGAATTTTGGACAATGGGCGAAAGCCTGAT | 28 | 0.11134971764893026 | No Hit |
| CCCGGTCATTTAGAGGAAGTAAAAGTCGTAACAAGGTTTCCGTAGGTGAA | 28 | 0.11134971764893026 | No Hit |
| CCTACGGGTGGCTGCAGTGGGGAATTTTGGACAATGGGCGAAAGCCTGAT | 27 | 0.10737294201861132 | No Hit |
| CCTACGGGGGGCTGCAGTGGGGAATCTTGCGCAATGGGCGAAAGCCTGAC | 27 | 0.10737294201861132 | No Hit |
| CCTACGGGGGGCAGCAGTGGGGAATCTTAGACAATGGGGGCAACCCTGAT | 27 | 0.10737294201861132 | No Hit |
| CCTACGGGCGGCTGCAGTGAGGAATATTGGTCAATGGGCGAGAGCCTGAA | 26 | 0.10339616638829238 | No Hit |
| CCTACGGGGGGCAGCAGTGGGGAATTTTGGACAATGGGCGCAAGCCTGAT | 26 | 0.10339616638829238 | No Hit |
| CCTACGGGCGGCAGCAGTAGGGAATCTTCGGCAATGGACGGAAGTCTGAC | 26 | 0.10339616638829238 | No Hit |
| CCTACGGGTGGCTGCAGTAGGGAATCTTCGGCAATGGGGGCAACCCTGAC | 26 | 0.10339616638829238 | No Hit |
| CCTACGGGGGGCTGCAGTGAGGAATATTGGTCAATGGGCGAGAGCCTGAA | 26 | 0.10339616638829238 | No Hit |

## Adapter Content

## Kmer Content

| Sequence | Count | PValue | Obs/Exp Max | Max Obs/Exp Position |
| --- | --- | --- | --- | --- |
| GCGATAG | 10 | 6.8894E-4 | 315.33975 | 295 |
| GGGAGAG | 15 | 5.4792417E-6 | 315.33975 | 295 |
| AGTGTTG | 25 | 3.45608E-10 | 315.33975 | 295 |
| ATTCGAA | 135 | 0.0 | 315.33975 | 295 |
| GCTTGTG | 10 | 6.8894E-4 | 315.33975 | 295 |
| GAGAGAG | 40 | 0.0 | 315.33975 | 295 |
| GGAAGAG | 245 | 0.0 | 302.46875 | 295 |
| GAGGCAG | 265 | 0.0 | 293.9821 | 8 |
| GGGCAGC | 260 | 0.0 | 293.9821 | 9 |
| GCGGCAG | 250 | 0.0 | 293.9821 | 8 |
| AGGCAGC | 265 | 0.0 | 293.9821 | 9 |
| CGGCTGC | 340 | 0.0 | 293.9821 | 9 |
| CGGCAGC | 250 | 0.0 | 293.9821 | 9 |
| CATTTAG | 20 | 6.171467E-8 | 293.98206 | 7 |
| TATTTAG | 10 | 8.50343E-4 | 293.98206 | 7 |
| TTTAGAG | 30 | 3.6379788E-12 | 293.98206 | 9 |
| GGGAAGC | 10 | 8.50343E-4 | 293.98206 | 6 |
| GGGCGGC | 585 | 0.0 | 293.98206 | 6 |
| TCATTTA | 20 | 6.171467E-8 | 293.98206 | 6 |
| CCGGTCA | 10 | 8.50343E-4 | 293.98206 | 2 |

Produced by FastQC (version 0.11.7)
