# Supplementary material for: Assessment of airborne bacteria from a public health institution in Mexico City
Source: PLOS Glob Public Health. 2024 Nov 7;4(11):e0003672. doi: 10.1371/journal.pgph.0003672 (PMC11542838; doi:10.1371/journal.pgph.0003672)
Supplement: S1 Text — (ZIP) [file pgph.0003672.s001.zip › Hospital_16S_QC/21022023_CUD1_16S_S43_L001_R2_001_fastqc.html]

21022023\_CUD1\_16S\_S43\_L001\_R2\_001.fastq.gz FastQC Report 

FastQC Report

Wed 15 Mar 2023  
21022023\_CUD1\_16S\_S43\_L001\_R2\_001.fastq.gz

## Summary

- Basic Statistics
- Per base sequence quality
- Per tile sequence quality
- Per sequence quality scores
- Per base sequence content
- Per sequence GC content
- Per base N content
- Sequence Length Distribution
- Sequence Duplication Levels
- Overrepresented sequences
- Adapter Content
- Kmer Content

## Basic Statistics

| Measure | Value |
| --- | --- |
| Filename | 21022023\_CUD1\_16S\_S43\_L001\_R2\_001.fastq.gz |
| File type | Conventional base calls |
| Encoding | Sanger / Illumina 1.9 |
| Total Sequences | 25146 |
| Sequences flagged as poor quality | 0 |
| Sequence length | 80-301 |
| %GC | 54 |

## Per base sequence quality

## Per tile sequence quality

## Per sequence quality scores

## Per base sequence content

## Per sequence GC content

## Per base N content

## Sequence Length Distribution

## Sequence Duplication Levels

## Overrepresented sequences

| Sequence | Count | Percentage | Possible Source |
| --- | --- | --- | --- |
| GACTACTGGGGTATCTAATCCTGTTTGCTCCCCACGCTTTCGCACCTCAG | 644 | 2.5610435059253955 | No Hit |
| GACTACAGGGGTATCTAATCCTGTTTGCTCCCCACGCTTTCGCACCTCAG | 552 | 2.1951801479360533 | No Hit |
| GACTACTAGGGTATCTAATCCTGTTTGCTCCCCACGCTTTCGCACCTCAG | 531 | 2.111667859699356 | No Hit |
| GACTACCGGGGTATCTAATCCTGTTTGCTCCCCACGCTTTCGCACCTCAG | 522 | 2.0758768790264854 | No Hit |
| GACTACTCGGGTATCTAATCCTGTTTGCTCCCCACGCTTTCGCACCTCAG | 518 | 2.0599697765052096 | No Hit |
| GACTACCAGGGTATCTAATCCTGTTTGCTCCCCACGCTTTCGCACCTCAG | 516 | 2.052016225244572 | No Hit |
| GACTACAAGGGTATCTAATCCTGTTTGCTCCCCACGCTTTCGCACCTCAG | 512 | 2.036109122723296 | No Hit |
| GACTACCCGGGTATCTAATCCTGTTTGCTCCCCACGCTTTCGCACCTCAG | 430 | 1.7100135210371432 | No Hit |
| GACTACACGGGTATCTAATCCTGTTTGCTCCCCACGCTTTCGCACCTCAG | 416 | 1.6543386622126781 | No Hit |
| GACTACTGGGGTATCTAATCCTGTTCGCTCCCCATGCTTTCGCTCCTCAG | 413 | 1.6424083353217211 | No Hit |
| GACTACTAGGGTATCTAATCCTGTTCGCTCCCCATGCTTTCGCTCCTCAG | 399 | 1.586733476497256 | No Hit |
| GACTACTGGGGTATCTAATCCTGTTTGCTCCCCACGCTTTCGCGCCTCAG | 379 | 1.5071979638908772 | No Hit |
| GACTACAGGGGTATCTAATCCTGTTCGCTCCCCATGCTTTCGCTCCTCAG | 360 | 1.4316392269148175 | No Hit |
| GACTACACGGGTATCTAATCCTGTTCGCTCCCCATGCTTTCGCTCCTCAG | 330 | 1.3123359580052494 | No Hit |
| GACTACCAGGGTATCTAATCCTGTTCGCTCCCCATGCTTTCGCTCCTCAG | 320 | 1.27256820170206 | No Hit |
| GACTACTCGGGTATCTAATCCTGTTCGCTCCCCATGCTTTCGCTCCTCAG | 319 | 1.268591426071741 | No Hit |
| GACTACTCGGGTATCTAATCCTGTTTGCTCCCCACGCTTTCGCGCCTCAG | 300 | 1.193032689095681 | No Hit |
| GACTACAAGGGTATCTAATCCTGTTCGCTCCCCATGCTTTCGCTCCTCAG | 297 | 1.1811023622047243 | No Hit |
| GACTACCCGGGTATCTAATCCTGTTCGCTCCCCATGCTTTCGCTCCTCAG | 297 | 1.1811023622047243 | No Hit |
| GACTACCGGGGTATCTAATCCTGTTCGCTCCCCATGCTTTCGCTCCTCAG | 297 | 1.1811023622047243 | No Hit |
| GACTACTAGGGTATCTAATCCTGTTTGCTCCCCACGCTTTCGCGCCTCAG | 276 | 1.0975900739680267 | No Hit |
| GACTACAGGGGTATCTAATCCTGTTTGCTCCCCACGCTTTCGCGCCTCAG | 276 | 1.0975900739680267 | No Hit |
| GACTACCGGGGTATCTAATCCTGTTTGCTCCCCACGCTTTCGCGCCTCAG | 271 | 1.077706195816432 | No Hit |
| GACTACCAGGGTATCTAATCCTGTTTGCTCCCCACGCTTTCGCGCCTCAG | 253 | 1.006124234470691 | No Hit |
| GACTACAAGGGTATCTAATCCTGTTTGCTCCCCACGCTTTCGCGCCTCAG | 241 | 0.958402926906864 | No Hit |
| GACTACCCGGGTATCTAATCCTGTTTGCTCCCCACGCTTTCGCGCCTCAG | 240 | 0.954426151276545 | No Hit |
| GACTACACGGGTATCTAATCCTGTTTGCTCCCCACGCTTTCGCGCCTCAG | 231 | 0.9186351706036745 | No Hit |
| GACTACTAGGGTATCTAATCCTGTTTGCTCCCCATGCTTTCGTACCTCAG | 226 | 0.8987512924520799 | No Hit |
| GACTACAGGGGTATCTAATCCTGTTTGCTCCCCATGCTTTCGTACCTCAG | 220 | 0.8748906386701663 | No Hit |
| GACTACTGGGGTATCTAATCCTGTTTGCTCCCCACGCTTTCGCACCTGAG | 216 | 0.8589835361488906 | No Hit |
| GACTACTGGGGTATCTAATCCTGTTTGCTCCCCATGCTTTCGTACCTCAG | 211 | 0.8390996579972958 | No Hit |
| GACTACCGGGGTATCTAATCCTGTTTGCTCCCCATGCTTTCGTACCTCAG | 210 | 0.8351228823669768 | No Hit |
| GACTACTGGGGTATCTAATCCTGTTTGATCCCCACGCTTTCGCACATCAG | 199 | 0.7913783504334686 | No Hit |
| GACTACAGGGGTATCTAATCCTGTTTGATCCCCACGCTTTCGCACATCAG | 194 | 0.7714944722818738 | No Hit |
| GACTACTAGGGTATCTAATCCTGTTTGATCCCCACGCTTTCGCACATCAG | 193 | 0.767517696651555 | No Hit |
| GACTACCAGGGTATCTAATCCTGTTTGCTCCCCATGCTTTCGTACCTCAG | 190 | 0.7555873697605981 | No Hit |
| GACTACTCGGGTATCTAATCCTGTTTGCTCCCCATGCTTTCGTACCTCAG | 185 | 0.7357034916090034 | No Hit |
| GACTACAAGGGTATCTAATCCTGTTTGCTCCCCACGCTTTCGCACCTGAG | 185 | 0.7357034916090034 | No Hit |
| GACTACTCGGGTATCTAATCCTGTTTGCTCCCCACGCTTTCGCACCTGAG | 184 | 0.7317267159786844 | No Hit |
| GACTACCGGGGTATCTAATCCTGTTTGCTCCCCACGCTTTCGCACCTGAG | 182 | 0.7237731647180466 | No Hit |
| GACTACAGGGGTATCTAATCCTGTTTGCTCCCCACGCTTTCGCACCTGAG | 180 | 0.7158196134574087 | No Hit |
| GACTACTAGGGTATCTAATCCTGTTTGCTCCCCACGCTTTCGCACCTGAG | 172 | 0.6840054084148572 | No Hit |
| GACTACCAGGGTATCTAATCCTGTTTGCTCCCCACGCTTTCGCACCTGAG | 164 | 0.6521912033723057 | No Hit |
| GACTACACGGGTATCTAATCCTGTTTGCTCCCCATGCTTTCGTACCTCAG | 163 | 0.6482144277419868 | No Hit |
| GACTACCCGGGTATCTAATCCTGTTTGCTCCCCACGCTTTCGCACCTGAG | 159 | 0.6323073252207111 | No Hit |
| GACTACTCGGGTATCTAATCCTGTTTGATCCCCACGCTTTCGCACATCAG | 153 | 0.6084466714387974 | No Hit |
| GACTACACGGGTATCTAATCCTGTTTGCTCCCCACGCTTTCGCACCTGAG | 145 | 0.576632466396246 | No Hit |
| GACTACCCGGGTATCTAATCCTGTTTGATCCCCACGCTTTCGCACATCAG | 142 | 0.5647021395052891 | No Hit |
| GACTACCGGGGTATCTAATCCTGTTTGATCCCCACGCTTTCGCACATCAG | 142 | 0.5647021395052891 | No Hit |
| GACTACAAGGGTATCTAATCCTGTTTGATCCCCACGCTTTCGCACATCAG | 141 | 0.5607253638749702 | No Hit |
| GACTACCCGGGTATCTAATCCTGTTTGCTCCCCATGCTTTCGTACCTCAG | 140 | 0.5567485882446512 | No Hit |
| GACTACCAGGGTATCTAATCCTGTTTGATCCCCACGCTTTCGCACATCAG | 138 | 0.5487950369840133 | No Hit |
| GACTACAAGGGTATCTAATCCTGTTTGCTCCCCATGCTTTCGTACCTCAG | 136 | 0.5408414857233754 | No Hit |
| GACTACACGGGTATCTAATCCTGTTTGATCCCCACGCTTTCGCACATCAG | 116 | 0.4613059731169967 | No Hit |
| GACTACAGGGGTATCTAATCCTGTTCGCTACCCATGCTTTCGCTCCTCAG | 114 | 0.4533524218563589 | No Hit |
| GACTACCGGGGTATCTAATCCTGTTCGCTACCCATGCTTTCGCTCCTCAG | 114 | 0.4533524218563589 | No Hit |
| GACTACCAGGGTATCTAATCCTGTTCGCTACCCATGCTTTCGCTCCTCAG | 112 | 0.44539887059572103 | No Hit |
| GACTACTGGGGTATCTAATCCTGTTCGCTACCCATGCTTTCGCTCCTCAG | 105 | 0.4175614411834884 | No Hit |
| GACTACTCGGGTATCTAATCCTGTTCGCTACCCATGCTTTCGCTCCTCAG | 103 | 0.4096078899228505 | No Hit |
| GACTACAAGGGTATCTAATCCTGTTCGCTACCCATGCTTTCGCTCCTCAG | 101 | 0.4016543386622127 | No Hit |
| GACTACTCGGGTATCTAATCCTGTTCGCTCCCCACGCTTTCGCTCCTCAG | 96 | 0.38177046051061797 | No Hit |
| GACTACAGGGGTATCTAATCCTGTTCGCTCCCCACGCTTTCGCTCCTCAG | 92 | 0.3658633579893422 | No Hit |
| GACTACCAGGGTATCTAATCCTGTTCGCTCCCCACGCTTTCGCTCCTCAG | 90 | 0.35790980672870437 | No Hit |
| GACTACACGGGTATCTAATCCTGTTCGCTACCCATGCTTTCGCTCCTCAG | 89 | 0.3539330310983854 | No Hit |
| GACTACTGGGGTATCTAATCCTGTTCGCTCCCCACGCTTTCGCTCCTCAG | 88 | 0.3499562554680665 | No Hit |
| GACTACTAGGGTATCTAATCCTGTTCGCTACCCATGCTTTCGCTCCTCAG | 87 | 0.34597947983774757 | No Hit |
| GACTACTGGGGTATCTAATCCTGTTTGCTCCCCACGCTTTCGTGCATGAG | 86 | 0.3420027042074286 | No Hit |
| GACTACAAGGGTATCTAATCCTGTTCGCTCCCCACGCTTTCGCTCCTCAG | 84 | 0.3340491529467908 | No Hit |
| GACTACTAGGGTATCTAATCCTGTTTGCTCCCCACGCTTTCGTGCATGAG | 83 | 0.3300723773164718 | No Hit |
| GACTACAGGGGTATCTAATCCTGTTTGCTCCCCACGCTTTCGTGCATGAG | 83 | 0.3300723773164718 | No Hit |
| GACTACTGGGGTATCTAATCCTGTTTGCTCCCCACGCTTTCGTGCCTCAG | 83 | 0.3300723773164718 | No Hit |
| GACTACTGGGGTATCTAATCCTGTTTGCTCCCCACGCTTTCGAGCCTCAG | 82 | 0.32609560168615287 | No Hit |
| GACTACTGGGGTATCTAATCCTGTTTGCTCCCCATGCTTTCGCACCTCAG | 81 | 0.322118826055834 | No Hit |
| GACTACCCGGGTATCTAATCCTGTTCGCTACCCATGCTTTCGCTCCTCAG | 80 | 0.318142050425515 | No Hit |
| GACTACTAGGGTATCTAATCCTGTTTGCTCCCCACGCTTTCGTGCCTCAG | 79 | 0.314165274795196 | No Hit |
| GACTACCCGGGTATCTAATCCTGTTCGCTCCCCACGCTTTCGCTCCTCAG | 78 | 0.3101884991648771 | No Hit |
| GACTACTCGGGTATCTAATCCTGTTTGCTCCCCACGCTTTCGTGCATGAG | 74 | 0.29428139664360137 | No Hit |
| GACTACTAGGGTATCTAATCCTGTTCGCTCCCCACGCTTTCGCTCCTCAG | 73 | 0.2903046210132824 | No Hit |
| GACTACCGGGGTATCTAATCCTGTTCGCTCCCCACGCTTTCGCTCCTCAG | 73 | 0.2903046210132824 | No Hit |
| GACTACACGGGTATCTAATCCTGTTCGCTCCCCACGCTTTCGCTCCTCAG | 72 | 0.28632784538296346 | No Hit |
| GACTACTCGGGTATCTAATCCTGTTTGCTCCCCACGCTTTCGAGCCTCAG | 72 | 0.28632784538296346 | No Hit |
| GACTACCGGGGTATCTAATCCTGTTTGCTCCCCACGCTTTCGAGCCTCAG | 71 | 0.28235106975264457 | No Hit |
| GACTACCGGGGTATCTAATCCTGTTTGCTCCCCATGCTTTCGCACCTCAG | 71 | 0.28235106975264457 | No Hit |
| GACTACAGGGGTATCTAATCCTGTTTGCTCCCCACGCTTTCGAGCCTCAG | 71 | 0.28235106975264457 | No Hit |
| GACTACACGGGTATCTAATCCTGTTTGCTCCCCACGCTTTCGTGCATGAG | 70 | 0.2783742941223256 | No Hit |
| GACTACCGGGGTATCTAATCCTGTTTGCTCCCCACGCTTTCGTGCCTCAG | 69 | 0.27439751849200666 | No Hit |
| GACTACAAGGGTATCTAATCCTGTTTGCTCCCCACGCTTTCGTGCCTCAG | 69 | 0.27439751849200666 | No Hit |
| GACTACCAGGGTATCTAATCCTGTTTGCTCCCCACGCTTTCGTGCCTCAG | 66 | 0.26246719160104987 | No Hit |
| GACTACTAGGGTATCTAATCCTGTTTGCTCCCCACGCTTTCGAGCCTCAG | 66 | 0.26246719160104987 | No Hit |
| GACTACTCGGGTATCTAATCCTGTTTGCTCCCCACGCTTTCGTGCCTCAG | 66 | 0.26246719160104987 | No Hit |
| GACTACCAGGGTATCTAATCCTGTTTGCTCCCCACGCTTTCGTGCATGAG | 63 | 0.25053686471009307 | No Hit |
| GACTACAAGGGTATCTAATCCTGTTTGCTCCCCACGCTTTCGTGCATGAG | 63 | 0.25053686471009307 | No Hit |
| GACTACTAGGGTATCTAATCCTGTTTGCTCCCCATGCTTTCGCACCTCAG | 61 | 0.2425833134494552 | No Hit |
| GACTACTCGGGTATCTAATCCTGTTTGCTCCCCATGCTTTCGCACCTCAG | 60 | 0.23860653781913624 | No Hit |
| GACTACAGGGGTATCTAATCCTGTTTGCTCCCCATGCTTTCGCACCTCAG | 60 | 0.23860653781913624 | No Hit |
| GCTGCGTTCTTCATCGATGCCGGAACCAAGAGATCCATTGTTGAAAGTTT | 60 | 0.23860653781913624 | No Hit |
| GACTACCCGGGTATCTAATCCTGTTTGCTCCCCACGCTTTCGTGCATGAG | 58 | 0.23065298655849836 | No Hit |
| GACTACACGGGTATCTAATCCTGTTTGCTCCCCATGCTTTCGCACCTCAG | 56 | 0.22269943529786052 | No Hit |
| GACTACCGGGGTATCTAATCCTGTTTGCTCCCCACGCTTTCGTGCATGAG | 56 | 0.22269943529786052 | No Hit |
| GACTACACGGGTATCTAATCCTGTTTGCTCCCCACGCTTTCGAGCCTCAG | 55 | 0.21872265966754156 | No Hit |
| GACTACAAGGGTATCTAATCCTGTTTGCTCCCCATGCTTTCGCACCTCAG | 54 | 0.21474588403722264 | No Hit |
| GACTACACGGGTATCTAATCCTGTTTGCTCCCCACGCTTTCGTGCCTCAG | 54 | 0.21474588403722264 | No Hit |
| GACTACCAGGGTATCTAATCCTGTTTGCTCCCCACGCTTTCGAGCCTCAG | 54 | 0.21474588403722264 | No Hit |
| GACTACCCGGGTATCTAATCCTGTTTGCTCCCCACGCTTTCGAGCCTCAG | 53 | 0.2107691084069037 | No Hit |
| GACTACAGGGGTATCTAATCCTGTTTGCTACCCACGCTTTCGAACCTCAG | 53 | 0.2107691084069037 | No Hit |
| GACTACAAGGGTATCTAATCCTGTTTGCTCCCCACGCTTTCGAGCCTCAG | 53 | 0.2107691084069037 | No Hit |
| GACTACCCGGGTATCTAATCCTGTTTGCTCCCCATGCTTTCGCACCTCAG | 49 | 0.19486200588562794 | No Hit |
| GACTACCAGGGTATCTAATCCTGTTTGCTCCCCATGCTTTCGCACCTCAG | 49 | 0.19486200588562794 | No Hit |
| GACTACAGGGGTATCTAATCCTGTTTGCTCCCCACGCTTTCGTGCCTCAG | 48 | 0.19088523025530899 | No Hit |
| GCTGCGTTCTTCATCGATGCCGGAACCAAGAGATCCGTTGTTGAAAGTTT | 46 | 0.1829316789946711 | No Hit |
| GACTACCCGGGTATCTAATCCTGTTTGCTCCCCACGCTTTCGTGCCTCAG | 46 | 0.1829316789946711 | No Hit |
| GACTACTGGGGTATCTAATCCTGTTTGCTACCCACGCTTTCGAACCTCAG | 45 | 0.17895490336435219 | No Hit |
| GACTACTAGGGTATCTAATCCTGTTTGCTACCCACGCTTTCGAACCTCAG | 40 | 0.1590710252127575 | No Hit |
| GACTACAAGGGTATCTAATCCTGTTTGCTACCCACGCTTTCGAACCTCAG | 40 | 0.1590710252127575 | No Hit |
| GACTACCCGGGTATCTAATCCTGTTTGCTACCCACGCTTTCGAACCTCAG | 38 | 0.1511174739521196 | No Hit |
| GACTACCGGGGTATCTAATCCTGTTTGCTACCCACGCTTTCGAACCTCAG | 36 | 0.14316392269148173 | No Hit |
| GACTACTGGGGTATCTAATCCTGTTCGCTCCCCATGCTTTCGCTTCTCAG | 36 | 0.14316392269148173 | No Hit |
| GACTACAGGGGTATCTAAGCCTGTTCGCTCCCCACGCTTTCGCTCCTCAG | 35 | 0.1391871470611628 | No Hit |
| GACTACCAGGGTATCTAATCCTGTTTGCTACCCACGCTTTCGAACCTCAG | 34 | 0.13521037143084386 | No Hit |
| GACTACAGGGGTATCTAATCCTGTTCGCTCCCCATGCTTTCGCTTCTCAG | 32 | 0.127256820170206 | No Hit |
| GACTACTAGGGTATCTAATCCTGTTCGCTCCCCATGCTTTCGCTTCTCAG | 31 | 0.12328004453988706 | No Hit |
| GCTGCGTTCTTCATCGATGCCAGAACCAAGAGATCCGTTGTTAAAAGTTT | 30 | 0.11930326890956812 | No Hit |
| GACTACCGGGGTATCTAATCCTGTTCGCTCCCCATGCTTTCGCTTCTCAG | 29 | 0.11532649327924918 | No Hit |
| GACTACCAGGGTATCTAATCCTGTTCGCTCCCCATGCTTTCGCTTCTCAG | 29 | 0.11532649327924918 | No Hit |
| GACTACTGGGGTATCTAATCCTGTTCGCTCCCCACGCTTTCGAGCCTCAG | 29 | 0.11532649327924918 | No Hit |
| GACTACTCGGGTATCTAATCCTGTTTGCTACCCACGCTTTCGAACCTCAG | 28 | 0.11134971764893026 | No Hit |
| GACTACACGGGTATCTAATCCTGTTTGCTACCCACGCTTTCGAACCTCAG | 28 | 0.11134971764893026 | No Hit |
| GACTACTCGGGTATCTAATCCTGTTCGCTCCCCACGCTTTCGTGCCTCAG | 27 | 0.10737294201861132 | No Hit |
| GACTACCCGGGTATCTAATCCTGTTCGCTCCCCATGCTTTCGCTTCTCAG | 27 | 0.10737294201861132 | No Hit |
| GACTACAGGGGTATCTAATCCGGTTCGCTCCCCACACTTTCGCGCCTCAG | 26 | 0.10339616638829238 | No Hit |

## Adapter Content

## Kmer Content

| Sequence | Count | PValue | Obs/Exp Max | Max Obs/Exp Position |
| --- | --- | --- | --- | --- |
| TTAGACG | 15 | 5.820766E-11 | 5266.607 | 295 |
| GTTTGGG | 5 | 2.847625E-4 | 5266.607 | 295 |
| GTGAGCG | 5 | 2.847625E-4 | 5266.607 | 295 |
| ATAGCCG | 5 | 2.847625E-4 | 5266.607 | 295 |
| GTTAGCG | 10 | 1.3386853E-7 | 5266.607 | 295 |
| TTAGGCG | 5 | 2.847625E-4 | 5266.607 | 295 |
| ACTGCTT | 10 | 0.0011389161 | 2633.3035 | 295 |
| GTTTGCA | 10 | 0.0011389161 | 2633.3035 | 295 |
| ATTACCA | 10 | 0.0011389161 | 2633.3035 | 295 |
| GTTTAGT | 15 | 0.00256226 | 1755.5358 | 295 |
| TTAGCCG | 200 | 0.0 | 1579.982 | 295 |
| AGTTAGG | 75 | 0.0 | 702.2143 | 295 |
| CTACAGG | 365 | 0.0 | 293.755 | 3 |
| ACTCGGG | 310 | 0.0 | 293.755 | 5 |
| TACAGGG | 365 | 0.0 | 293.755 | 4 |
| GGGGTAT | 910 | 0.0 | 293.755 | 8 |
| TACTCGG | 310 | 0.0 | 293.755 | 4 |
| TACCAGG | 250 | 0.0 | 293.755 | 4 |
| CTACTCG | 310 | 0.0 | 293.755 | 3 |
| GACTACT | 915 | 0.0 | 293.755 | 1 |

Produced by FastQC (version 0.11.7)
