# Supplementary material for: Assessment of airborne bacteria from a public health institution in Mexico City
Source: PLOS Glob Public Health. 2024 Nov 7;4(11):e0003672. doi: 10.1371/journal.pgph.0003672 (PMC11542838; doi:10.1371/journal.pgph.0003672)
Supplement: S1 Text — (ZIP) [file pgph.0003672.s001.zip › Hospital_16S_QC/21022023_CUD2_16S_S44_L001_R1_001_fastqc.html]

21022023\_CUD2\_16S\_S44\_L001\_R1\_001.fastq.gz FastQC Report 

FastQC Report

Wed 15 Mar 2023  
21022023\_CUD2\_16S\_S44\_L001\_R1\_001.fastq.gz

## Summary

- Basic Statistics
- Per base sequence quality
- Per tile sequence quality
- Per sequence quality scores
- Per base sequence content
- Per sequence GC content
- Per base N content
- Sequence Length Distribution
- Sequence Duplication Levels
- Overrepresented sequences
- Adapter Content
- Kmer Content

## Basic Statistics

| Measure | Value |
| --- | --- |
| Filename | 21022023\_CUD2\_16S\_S44\_L001\_R1\_001.fastq.gz |
| File type | Conventional base calls |
| Encoding | Sanger / Illumina 1.9 |
| Total Sequences | 38708 |
| Sequences flagged as poor quality | 0 |
| Sequence length | 35-301 |
| %GC | 55 |

## Per base sequence quality

## Per tile sequence quality

## Per sequence quality scores

## Per base sequence content

## Per sequence GC content

## Per base N content

## Sequence Length Distribution

## Sequence Duplication Levels

## Overrepresented sequences

| Sequence | Count | Percentage | Possible Source |
| --- | --- | --- | --- |
| CCTACGGGAGGCTGCAGTGGGGAATATTGCACAATGGGCGCAAGCCTGAT | 632 | 1.6327374186214736 | No Hit |
| CCTACGGGGGGCTGCAGTGGGGAATATTGCACAATGGGCGCAAGCCTGAT | 621 | 1.6043195205125556 | No Hit |
| CCTACGGGTGGCTGCAGTGGGGAATATTGCACAATGGGCGCAAGCCTGAT | 620 | 1.6017360752299266 | No Hit |
| CCTACGGGAGGCTGCAGTGGGGAATATTGCACAATGGGCGAAAGCCTGAT | 607 | 1.5681512865557505 | No Hit |
| CCTACGGGTGGCTGCAGTGGGGAATATTGCACAATGGGCGAAAGCCTGAT | 596 | 1.5397333884468327 | No Hit |
| CCTACGGGTGGCTGCAGTGGGGAATATTGGACAATGGGCGCAAGCCTGAT | 590 | 1.5242327167510592 | No Hit |
| CCTACGGGAGGCTGCAGTGGGGAATATTGGACAATGGGCGCAAGCCTGAT | 576 | 1.4880644827942544 | No Hit |
| CCTACGGGGGGCAGCAGTGGGGAATATTGCACAATGGGCGCAAGCCTGAT | 561 | 1.4493128035548208 | No Hit |
| CCTACGGGAGGCAGCAGTGGGGAATATTGCACAATGGGCGCAAGCCTGAT | 561 | 1.4493128035548208 | No Hit |
| CCTACGGGGGGCTGCAGTGGGGAATATTGGACAATGGGCGCAAGCCTGAT | 551 | 1.4234783507285316 | No Hit |
| CCTACGGGTGGCAGCAGTGGGGAATATTGCACAATGGGCGCAAGCCTGAT | 515 | 1.3304743205538907 | No Hit |
| CCTACGGGGGGCTGCAGTGGGGAATATTGCACAATGGGCGAAAGCCTGAT | 514 | 1.3278908752712617 | No Hit |
| CCTACGGGGGGCAGCAGTGGGGAATATTGCACAATGGGCGAAAGCCTGAT | 502 | 1.2968895318797147 | No Hit |
| CCTACGGGGGGCAGCAGTGGGGAATATTGGACAATGGGCGCAAGCCTGAT | 487 | 1.258137852640281 | No Hit |
| CCTACGGGAGGCAGCAGTGGGGAATATTGCACAATGGGCGAAAGCCTGAT | 483 | 1.2478040715097654 | No Hit |
| CCTACGGGCGGCTGCAGTGGGGAATATTGGACAATGGGCGCAAGCCTGAT | 475 | 1.227136509248734 | No Hit |
| CCTACGGGCGGCTGCAGTGGGGAATATTGCACAATGGGCGCAAGCCTGAT | 451 | 1.1651338224656402 | No Hit |
| CCTACGGGTGGCTGCAGTGGGGAATATTGGACAATGGGCGAAAGCCTGAT | 427 | 1.1031311356825462 | No Hit |
| CCTACGGGAGGCAGCAGTGGGGAATATTGGACAATGGGCGCAAGCCTGAT | 427 | 1.1031311356825462 | No Hit |
| CCTACGGGTGGCAGCAGTGGGGAATATTGCACAATGGGCGAAAGCCTGAT | 425 | 1.0979642451172884 | No Hit |
| CCTACGGGAGGCTGCAGTGGGGAATATTGGACAATGGGCGAAAGCCTGAT | 419 | 1.082463573421515 | No Hit |
| CCTACGGGGGGCTGCAGTGGGGAATATTGGACAATGGGCGAAAGCCTGAT | 413 | 1.0669629017257414 | No Hit |
| CCTACGGGGGGCAGCAGTGGGGAATATTGGACAATGGGCGAAAGCCTGAT | 408 | 1.0540456753125969 | No Hit |
| CCTACGGGTGGCAGCAGTGGGGAATATTGGACAATGGGCGCAAGCCTGAT | 402 | 1.0385450036168233 | No Hit |
| CCTACGGGCGGCTGCAGTGGGGAATATTGCACAATGGGCGAAAGCCTGAT | 397 | 1.0256277772036788 | No Hit |
| CCTACGGGGGGCTGCAGTGGGGAATCTTAGACAATGGGGGCAACCCTGAT | 386 | 0.9972098790947608 | No Hit |
| CCTACGGGTGGCTGCAGTGGGGAATCTTAGACAATGGGGGCAACCCTGAT | 382 | 0.9868760979642451 | No Hit |
| CCTACGGGAGGCAGCAGTGGGGAATATTGGACAATGGGCGAAAGCCTGAT | 369 | 0.9532913092900693 | No Hit |
| CCTACGGGTGGCAGCAGTGGGGAATATTGGACAATGGGCGAAAGCCTGAT | 365 | 0.9429575281595536 | No Hit |
| CCTACGGGAGGCTGCAGTGGGGAATCTTAGACAATGGGGGCAACCCTGAT | 351 | 0.9067892942027488 | No Hit |
| CCTACGGGCGGCAGCAGTGGGGAATATTGCACAATGGGCGCAAGCCTGAT | 350 | 0.9042058489201199 | No Hit |
| CCTACGGGGGGCAGCAGTAGGGAATCTTCCGCAATGGGCGAAAGCCTGAC | 343 | 0.8861217319417174 | No Hit |
| CCTACGGGCGGCTGCAGTGGGGAATATTGGACAATGGGCGAAAGCCTGAT | 339 | 0.8757879508112018 | No Hit |
| CCTACGGGAGGCTGCAGTGGGGAATATTGCACAATGGGCGGAAGCCTGAT | 336 | 0.8680376149633151 | No Hit |
| CCTACGGGCGGCAGCAGTGGGGAATATTGCACAATGGGCGAAAGCCTGAT | 336 | 0.8680376149633151 | No Hit |
| CCTACGGGAGGCAGCAGTGGGGAATCTTAGACAATGGGGGCAACCCTGAT | 311 | 0.8034514828975923 | No Hit |
| CCTACGGGCGGCAGCAGTGGGGAATATTGGACAATGGGCGCAAGCCTGAT | 303 | 0.782783920636561 | No Hit |
| CCTACGGGCGGCAGCAGTGGGGAATATTGGACAATGGGCGAAAGCCTGAT | 302 | 0.780200475353932 | No Hit |
| CCTACGGGGGGCAGCAGTGGGGAATCTTAGACAATGGGGGCAACCCTGAT | 298 | 0.7698666942234164 | No Hit |
| CCTACGGGCGGCTGCAGTGGGGAATCTTAGACAATGGGGGCAACCCTGAT | 297 | 0.7672832489407875 | No Hit |
| CCTACGGGAGGCAGCAGTAGGGAATCTTCCGCAATGGGCGAAAGCCTGAC | 292 | 0.7543660225276428 | No Hit |
| CCTACGGGTGGCTGCAGTGGGGAATATTGCACAATGGGCGGAAGCCTGAT | 287 | 0.7414487961144983 | No Hit |
| CCTACGGGAGGCTGCAGTAGGGAATCTTCCGCAATGGGCGAAAGCCTGAC | 279 | 0.720781233853467 | No Hit |
| CCTACGGGGGGCTGCAGTGGGGAATATTGCACAATGGGCGGAAGCCTGAT | 269 | 0.6949467810271779 | No Hit |
| CCTACGGGTGGCAGCAGTAGGGAATCTTCCGCAATGGGCGAAAGCCTGAC | 267 | 0.68977989046192 | No Hit |
| CCTACGGGGGGCTGCAGTAGGGAATCTTCCGCAATGGGCGAAAGCCTGAC | 246 | 0.6355275395267128 | No Hit |
| CCTACGGGTGGCAGCAGTGGGGAATCTTAGACAATGGGGGCAACCCTGAT | 244 | 0.630360648961455 | No Hit |
| CCTACGGGCGGCTGCAGTGGGGAATATTGCACAATGGGCGGAAGCCTGAT | 240 | 0.6200268678309393 | No Hit |
| CCTACGGGTGGCTGCAGTAGGGAATCTTCCGCAATGGGCGAAAGCCTGAC | 237 | 0.6122765319830527 | No Hit |
| CCTACGGGGGGCAGCAGTAGGGAATCTTCCGCAATGGACGAAAGTCTGAC | 226 | 0.5838586338741345 | No Hit |
| CCTACGGGAGGCAGCAGTAGGGAATCTTCCGCAATGGACGAAAGTCTGAC | 223 | 0.5761082980262477 | No Hit |
| CCTACGGGAGGCAGCAGTGGGGAATATTGCACAATGGGCGGAAGCCTGAT | 223 | 0.5761082980262477 | No Hit |
| CCTACGGGTGGCAGCAGTAGGGAATCTTCCGCAATGGACGAAAGTCTGAC | 218 | 0.5631910716131032 | No Hit |
| CCTACGGGGGGCAGCAGTGGGGAATATTGCACAATGGGCGGAAGCCTGAT | 216 | 0.5580241810478453 | No Hit |
| CCTACGGGCGGCAGCAGTAGGGAATCTTCCGCAATGGGCGAAAGCCTGAC | 214 | 0.5528572904825876 | No Hit |
| CCTACGGGAGGCTGCAGTGGGGAATTTTGGACAATGGGCGCAAGCCTGAT | 209 | 0.5399400640694431 | No Hit |
| CCTACGGGGGGCTGCAGTGGGGAATTTTGGACAATGGGCGCAAGCCTGAT | 200 | 0.5166890565257828 | No Hit |
| CCTACGGGAGGCTGCAGTAGGGAATCTTCCGCAATGGACGAAAGTCTGAC | 194 | 0.5011883848300094 | No Hit |
| CCTACGGGGGGCAGCAGTGGGGAATTTTGGACAATGGGCGCAAGCCTGAT | 187 | 0.48310426785160687 | No Hit |
| CCTACGGGCGGCAGCAGTGGGGAATATTGCACAATGGGCGGAAGCCTGAT | 179 | 0.46243670559057554 | No Hit |
| CCTACGGGTGGCTGCAGTGGGGAATTTTGGACAATGGGCGCAAGCCTGAT | 178 | 0.45985326030794665 | No Hit |
| CCTACGGGTGGCTGCAGTGGGGAATATTGGACAATGGGCGGAAGCCTGAT | 177 | 0.4572698150253178 | No Hit |
| CCTACGGGTGGCAGCAGTGGGGAATATTGCACAATGGGCGGAAGCCTGAT | 175 | 0.45210292446005995 | No Hit |
| CCTACGGGCGGCAGCAGTGGGGAATCTTAGACAATGGGGGCAACCCTGAT | 168 | 0.43401880748165755 | No Hit |
| CCTACGGGCGGCAGCAGTAGGGAATCTTCCGCAATGGACGAAAGTCTGAC | 159 | 0.41076779993799734 | No Hit |
| CCTACGGGCGGCTGCAGTAGGGAATCTTCCGCAATGGGCGAAAGCCTGAC | 156 | 0.4030174640901106 | No Hit |
| CCTACGGGAGGCTGCAGTGGGGAATATTGGACAATGGGCGGAAGCCTGAT | 155 | 0.40043401880748164 | No Hit |
| CCTACGGGTGGCAGCAGTGGGGAATTTTGGACAATGGGCGCAAGCCTGAT | 150 | 0.38751679239433706 | No Hit |
| CCTACGGGAGGCAGCAGTGGGGAATTTTGGACAATGGGCGCAAGCCTGAT | 149 | 0.3849333471117082 | No Hit |
| CCTACGGGAGGCTGCAGTGGGGAATATTGGACAATGGGGGGAACCCTGAT | 146 | 0.3771830112638214 | No Hit |
| CCTACGGGTGGCTGCAGTAGGGAATCTTCCGCAATGGACGAAAGTCTGAC | 144 | 0.3720161206985636 | No Hit |
| CCTACGGGGGGCAGCAGTGGGGAATATTGGACAATGGGCGGAAGCCTGAT | 140 | 0.36168233956804796 | No Hit |
| CCTACGGGTGGCTGCAGTGGGGAATATTGGACAATGGGGGGAACCCTGAT | 140 | 0.36168233956804796 | No Hit |
| CCTACGGGGGGCTGCAGTGGGGAATATTGGACAATGGGGGGAACCCTGAT | 139 | 0.359098894285419 | No Hit |
| CCTACGGGGGGCTGCAGTGGGGAATATTGGACAATGGGCGGAAGCCTGAT | 135 | 0.3487651131549034 | No Hit |
| CCTACGGGCGGCAGCAGTGGGGAATTTTGGACAATGGGCGCAAGCCTGAT | 134 | 0.34618166787227445 | No Hit |
| CCTACGGGAGGCAGCAGTGGGGAATATTGGACAATGGGGGGAACCCTGAT | 133 | 0.34359822258964556 | No Hit |
| CCTACGGGGGGCTGCAGTAGGGAATCTTCCGCAATGGACGAAAGTCTGAC | 131 | 0.33843133202438774 | No Hit |
| CCTACGGGCGGCTGCAGTGGGGAATTTTGGACAATGGGCGCAAGCCTGAT | 128 | 0.330680996176501 | No Hit |
| CCTACGGGGGGCAGCAGTGGGGAATATTGGACAATGGGGGGAACCCTGAT | 127 | 0.3280975508938721 | No Hit |
| CCTACGGGAGGCAGCAGTGGGGAATATTGGACAATGGGGGCAACCCTGAT | 124 | 0.3203472150459853 | No Hit |
| CCTACGGGTGGCTGCAGTGGGGAATATTGGACAATGGGGGCAACCCTGAT | 123 | 0.3177637697633564 | No Hit |
| CCTACGGGTGGCAGCAGTGGGGAATATTGGACAATGGGCGGAAGCCTGAT | 122 | 0.3151803244807275 | No Hit |
| CCTACGGGAGGCTGCAGTGGGGAATATTGGACAATGGGGGCAACCCTGAT | 122 | 0.3151803244807275 | No Hit |
| CCTACGGGAGGCAGCAGTGGGGAATATTGGACAATGGGCGGAAGCCTGAT | 119 | 0.30742998863284077 | No Hit |
| CCTACGGGTGGCAGCAGTGGGGAATATTGGACAATGGGGGGAACCCTGAT | 119 | 0.30742998863284077 | No Hit |
| CCTACGGGTGGCTGCAGTGGGGAATATTGCGCAATGGGCGAAAGCCTGAC | 114 | 0.2945127622196962 | No Hit |
| CCTACGGGCGGCTGCAGTGGGGAATATTGGACAATGGGCGGAAGCCTGAT | 113 | 0.29192931693706725 | No Hit |
| CCTACGGGGGGCTGCAGTGGGGAATATTGGACAATGGGGGCAACCCTGAT | 112 | 0.28934587165443837 | No Hit |
| CCTACGGGAGGCTGCAGTGGGGAATATTGCGCAATGGGCGAAAGCCTGAC | 111 | 0.28676242637180943 | No Hit |
| CCTACGGGCGGCTGCAGTAGGGAATCTTCCGCAATGGACGAAAGTCTGAC | 107 | 0.2764286452412938 | No Hit |
| CCTACGGGCGGCTGCAGTGGGGAATATTGGACAATGGGGGCAACCCTGAT | 103 | 0.2660948641107781 | No Hit |
| CCTACGGGGGGCAGCAGTGGGGAATATTGGACAATGGGGGCAACCCTGAT | 101 | 0.26092797354552033 | No Hit |
| CCTACGGGCGGCTGCAGTGGGGAATATTGGACAATGGGGGGAACCCTGAT | 99 | 0.25576108298026246 | No Hit |
| CCTACGGGCGGCAGCAGTGGGGAATATTGGACAATGGGGGCAACCCTGAT | 93 | 0.24026041128448902 | No Hit |
| CTTGGTCATTTAGAGGAAGTAAAAGTCGTAACAAGGTTTCCGTAGGTGAA | 93 | 0.24026041128448902 | No Hit |
| CCTACGGGCGGCAGCAGTGGGGAATATTGGACAATGGGGGGAACCCTGAT | 91 | 0.23509352071923117 | No Hit |
| CCTACGGGTGGCAGCAGTGGGGAATATTGGACAATGGGGGCAACCCTGAT | 90 | 0.23251007543660224 | No Hit |
| CCTACGGGGGGCTGCAGTGGGGAATATTGCGCAATGGGCGAAAGCCTGAC | 85 | 0.21959284902345766 | No Hit |
| CCTACGGGTGGCAGCAGTAGGGAATCTTCCGCAATGGACGCAAGTCTGAC | 83 | 0.21442595845819987 | No Hit |
| CCTACGGGGGGCAGCAGTAGGGAATCTTCCGCAATGGACGCAAGTCTGAC | 83 | 0.21442595845819987 | No Hit |
| CCTACGGGCGGCTGCAGTGGGGAATATTGCGCAATGGGCGAAAGCCTGAC | 80 | 0.20667562261031314 | No Hit |
| CCTACGGGGGGCAGCAGTGGGGAATATTGCGCAATGGGCGAAAGCCTGAC | 79 | 0.2040921773276842 | No Hit |
| CCTACGGGAGGCAGCAGTGGGGAATATTGCGCAATGGGCGAAAGCCTGAC | 78 | 0.2015087320450553 | No Hit |
| CCTACGGGCGGCAGCAGTGGGGAATATTGGACAATGGGCGGAAGCCTGAT | 78 | 0.2015087320450553 | No Hit |
| CCTACGGGAGGCAGCAGTAGGGAATCTTCCGCAATGGACGCAAGTCTGAC | 78 | 0.2015087320450553 | No Hit |
| CCTACGGGTGGCAGCAGTGGGGAATATTGCGCAATGGGCGAAAGCCTGAC | 69 | 0.17825772450139504 | No Hit |
| CCTACGGGAGGCTGCAGTAGGGAATCTTCCGCAATGGACGCAAGTCTGAC | 66 | 0.17050738865350834 | No Hit |
| CCTACGGGTGGCAGCAGTAGGGAATCTTCCACAATGGACGAAAGTCTGAT | 65 | 0.1679239433708794 | No Hit |
| CCTACGGGTGGCTGCAGTGGGGAATTTTGGACAATGGGCGAAAGCCTGAT | 62 | 0.16017360752299264 | No Hit |
| CCTACGGGGGGCAGCAGTGGGGAATATTGCGCAATGGGCGGAAGCCTGAC | 62 | 0.16017360752299264 | No Hit |
| CCTACGGGCGGCAGCAGTGGGGAATATTGCGCAATGGGCGAAAGCCTGAC | 61 | 0.15759016224036376 | No Hit |
| CCTACGGGAGGCTGCAGTGGGGAATTTTGGACAATGGGCGAAAGCCTGAT | 59 | 0.15242327167510591 | No Hit |
| CCTACGGGGGGCAGCAGTAGGGAATCTTCCACAATGGACGAAAGTCTGAT | 59 | 0.15242327167510591 | No Hit |
| CCTACGGGTGGCTGCAGTGGGGAATATTGCACAATGGGGGAAACCCTGAT | 58 | 0.149839826392477 | No Hit |
| CCTACGGGAGGCAGCAGTAGGGAATCTTCCACAATGGACGAAAGTCTGAT | 57 | 0.1472563811098481 | No Hit |
| CCTACGGGGGGCTGCAGTGGGGAATATTGCACAATGGGGGAAACCCTGAT | 55 | 0.14208949054459027 | No Hit |
| CCTACGGGCGGCTGCAGTAGGGAATCTTCCGCAATGGACGCAAGTCTGAC | 55 | 0.14208949054459027 | No Hit |
| CCTACGGGTGGCTGCAGTGGGGAATCTTAGACAATGGGCGCAAGCCTGAT | 53 | 0.13692259997933245 | No Hit |
| CCTACGGGTGGCTGCAGTAGGGAATCTTCCGCAATGGACGCAAGTCTGAC | 52 | 0.13433915469670352 | No Hit |
| CCTACGGGCGGCAGCAGTAGGGAATCTTCCGCAATGGACGCAAGTCTGAC | 52 | 0.13433915469670352 | No Hit |
| CCTACGGGTGGCAGCAGTGGGGAATCTTGGACAATGGGGGCAACCCTGAT | 52 | 0.13433915469670352 | No Hit |
| CCTACGGGGGGCTGCAGTAGGGAATCTTCCGCAATGGACGCAAGTCTGAC | 51 | 0.1317557094140746 | No Hit |
| CCTACGGGAGGCTGCAGTGGGGAATATTGCACAATGGGGGAAACCCTGAT | 51 | 0.1317557094140746 | No Hit |
| CCTACGGGGGGCAGCAGTAGGGAATATTGGGCAATGGGCGAGAGCCTGAC | 50 | 0.1291722641314457 | No Hit |
| CCTACGGGTGGCTGCAGTGGGGAATCTTGGACAATGGGGGCAACCCTGAT | 49 | 0.1265888188488168 | No Hit |
| CCTACGGGTGGCAGCAGTGGGGAATTTTGGACAATGGGCGAAAGCCTGAT | 49 | 0.1265888188488168 | No Hit |
| CCTACGGGAGGCTGCAGTAGGGAATCTTCCACAATGGACGAAAGTCTGAT | 48 | 0.12400537356618788 | No Hit |
| CCTACGGGAGGCTGCAGTGGGGAATATTGCGCAATGGGCGGAAGCCTGAC | 48 | 0.12400537356618788 | No Hit |
| CCTACGGGTGGCTGCAGTGGGGAATCTTAGACAATGGGGGAAACCCTGAT | 47 | 0.12142192828355895 | No Hit |
| CCTACGGGGGGCTGCAGTGGGGAATTTTGGACAATGGGCGAAAGCCTGAT | 47 | 0.12142192828355895 | No Hit |
| CCTACGGGTGGCTGCAGTGGGGAATATTGCGCAATGGGCGGAAGCCTGAC | 47 | 0.12142192828355895 | No Hit |
| CCTACGGGGGGCAGCAGTGGGGAATTTTGGACAATGGGGGCAACCCTGAT | 46 | 0.11883848300093004 | No Hit |
| CCTACGGGTGGCAGCAGTGGGGAATATTGCGCAATGGGCGGAAGCCTGAC | 45 | 0.11625503771830112 | No Hit |
| CCTACGGGAGGCTGCAGTGGGGAATCTTAGACAATGGGCGCAAGCCTGAT | 45 | 0.11625503771830112 | No Hit |
| CCTACGGGAGGCTGCAGTGGGGAATCTTAGACAATGGGGGAAACCCTGAT | 45 | 0.11625503771830112 | No Hit |
| CCTACGGGTGGCAGCAGTGGGGAATTTTGGACAATGGGGGCAACCCTGAT | 44 | 0.11367159243567222 | No Hit |
| CCTACGGGTGGCTGCAGTGGGGAATTTTGGACAATGGGGGCAACCCTGAT | 44 | 0.11367159243567222 | No Hit |
| CCTACGGGGGGCTGCAGTGGGGAATTTTCCGCAATGGGCGAAAGCCTGAC | 43 | 0.1110881471530433 | No Hit |
| CCTACGGGAGGCAGCAGTGGGGAATATTGCGCAATGGGCGGAAGCCTGAC | 43 | 0.1110881471530433 | No Hit |
| CCTACGGGAGGCTGCAGTGGGGAATTTTGGACAATGGGGGCAACCCTGAT | 42 | 0.10850470187041439 | No Hit |
| CCTACGGGGGGCTGCAGTGGGGAATCTTAGACAATGGGGGAAACCCTGAT | 42 | 0.10850470187041439 | No Hit |
| CCTACGGGGGGCTGCAGTGGGGAATCTTGGACAATGGGGGCAACCCTGAT | 42 | 0.10850470187041439 | No Hit |
| CCTACGGGGGGCTGCAGTGGGGAATCTTAGACAATGGGCGCAAGCCTGAT | 41 | 0.10592125658778546 | No Hit |
| CCTACGGGCGGCTGCAGTGGGGAATCTTAGACAATGGGCGCAAGCCTGAT | 40 | 0.10333781130515657 | No Hit |
| CCTACGGGAGGCTGCAGTGGGGAATCTTGGACAATGGGGGCAACCCTGAT | 40 | 0.10333781130515657 | No Hit |
| CCTACGGGTGGCTGCAGTAGGGAATATTGGGCAATGGGCGAGAGCCTGAC | 40 | 0.10333781130515657 | No Hit |
| CCTACGGGAGGCAGCAGTGGGGAATTTTGGACAATGGGCGAAAGCCTGAT | 40 | 0.10333781130515657 | No Hit |
| CCTACGGGAGGCTGCAGTGGGGAATCTTGCGCAATGGGCGAAAGCCTGAC | 40 | 0.10333781130515657 | No Hit |
| CCTACGGGTGGCAGCAGTAGGGAATATTGGGCAATGGGCGAGAGCCTGAC | 40 | 0.10333781130515657 | No Hit |
| CCTACGGGTGGCTGCAGTGGGGAATTTTCCGCAATGGGCGAAAGCCTGAC | 39 | 0.10075436602252764 | No Hit |

## Adapter Content

## Kmer Content

| Sequence | Count | PValue | Obs/Exp Max | Max Obs/Exp Position |
| --- | --- | --- | --- | --- |
| AGTGTTG | 55 | 0.0 | 313.5248 | 295 |
| GCTGGTG | 15 | 5.6325553E-6 | 313.52478 | 295 |
| TCGGCAG | 10 | 7.0255034E-4 | 313.52478 | 295 |
| ATTGGAT | 10 | 7.0255034E-4 | 313.52478 | 295 |
| GTATCAG | 10 | 7.0255034E-4 | 313.52478 | 295 |
| CATTCAA | 15 | 5.6325553E-6 | 313.52478 | 295 |
| ATGTGAT | 40 | 0.0 | 313.52478 | 295 |
| GAACTGG | 20 | 4.5039997E-8 | 313.52478 | 295 |
| TACTGAT | 20 | 4.5039997E-8 | 313.52478 | 295 |
| ATTTGAA | 45 | 0.0 | 313.52478 | 295 |
| GAGAGAG | 235 | 0.0 | 313.52478 | 295 |
| CTTTGTG | 80 | 0.0 | 313.52478 | 295 |
| TCCGAAA | 10 | 7.0255034E-4 | 313.52478 | 295 |
| GGGAGAG | 250 | 0.0 | 300.9838 | 295 |
| ATCCAAA | 110 | 0.0 | 299.27368 | 295 |
| AGGTATG | 90 | 0.0 | 296.10672 | 295 |
| ATCTGAT | 90 | 0.0 | 296.10672 | 295 |
| CTACGGA | 15 | 7.276565E-6 | 294.08142 | 2 |
| GCGGCAG | 345 | 0.0 | 294.0814 | 8 |
| CTTAGTT | 10 | 8.5126486E-4 | 294.0814 | 1 |

Produced by FastQC (version 0.11.7)
