# Supplementary material for: Assessment of airborne bacteria from a public health institution in Mexico City
Source: PLOS Glob Public Health. 2024 Nov 7;4(11):e0003672. doi: 10.1371/journal.pgph.0003672 (PMC11542838; doi:10.1371/journal.pgph.0003672)
Supplement: S1 Text — (ZIP) [file pgph.0003672.s001.zip › Hospital_16S_QC/21022023_CUD2_16S_S44_L001_R2_001_fastqc.html]

21022023\_CUD2\_16S\_S44\_L001\_R2\_001.fastq.gz FastQC Report 

FastQC Report

Wed 15 Mar 2023  
21022023\_CUD2\_16S\_S44\_L001\_R2\_001.fastq.gz

## Summary

- Basic Statistics
- Per base sequence quality
- Per tile sequence quality
- Per sequence quality scores
- Per base sequence content
- Per sequence GC content
- Per base N content
- Sequence Length Distribution
- Sequence Duplication Levels
- Overrepresented sequences
- Adapter Content
- Kmer Content

## Basic Statistics

| Measure | Value |
| --- | --- |
| Filename | 21022023\_CUD2\_16S\_S44\_L001\_R2\_001.fastq.gz |
| File type | Conventional base calls |
| Encoding | Sanger / Illumina 1.9 |
| Total Sequences | 38708 |
| Sequences flagged as poor quality | 0 |
| Sequence length | 35-301 |
| %GC | 54 |

## Per base sequence quality

## Per tile sequence quality

## Per sequence quality scores

## Per base sequence content

## Per sequence GC content

## Per base N content

## Sequence Length Distribution

## Sequence Duplication Levels

## Overrepresented sequences

| Sequence | Count | Percentage | Possible Source |
| --- | --- | --- | --- |
| GACTACTGGGGTATCTAATCCTGTTCGCTCCCCATGCTTTCGCTCCTCAG | 925 | 2.389686886431745 | No Hit |
| GACTACTGGGGTATCTAATCCTGTTTGCTCCCCACGCTTTCGCACCTCAG | 789 | 2.038338327994213 | No Hit |
| GACTACAGGGGTATCTAATCCTGTTCGCTCCCCATGCTTTCGCTCCTCAG | 786 | 2.030587992146326 | No Hit |
| GACTACCGGGGTATCTAATCCTGTTCGCTCCCCATGCTTTCGCTCCTCAG | 710 | 1.834246150666529 | No Hit |
| GACTACAGGGGTATCTAATCCTGTTTGCTCCCCACGCTTTCGCACCTCAG | 709 | 1.8316627053839 | No Hit |
| GACTACTCGGGTATCTAATCCTGTTCGCTCCCCATGCTTTCGCTCCTCAG | 689 | 1.7799937997313215 | No Hit |
| GACTACCGGGGTATCTAATCCTGTTTGCTCCCCACGCTTTCGCACCTCAG | 689 | 1.7799937997313215 | No Hit |
| GACTACTAGGGTATCTAATCCTGTTCGCTCCCCATGCTTTCGCTCCTCAG | 684 | 1.767076573318177 | No Hit |
| GACTACTAGGGTATCTAATCCTGTTTGCTCCCCACGCTTTCGCACCTCAG | 680 | 1.7567427921876613 | No Hit |
| GACTACCAGGGTATCTAATCCTGTTCGCTCCCCATGCTTTCGCTCCTCAG | 675 | 1.743825565774517 | No Hit |
| GACTACAAGGGTATCTAATCCTGTTCGCTCCCCATGCTTTCGCTCCTCAG | 671 | 1.7334917846440014 | No Hit |
| GACTACTCGGGTATCTAATCCTGTTTGCTCCCCACGCTTTCGCACCTCAG | 663 | 1.71282422238297 | No Hit |
| GACTACCCGGGTATCTAATCCTGTTCGCTCCCCATGCTTTCGCTCCTCAG | 650 | 1.679239433708794 | No Hit |
| GACTACACGGGTATCTAATCCTGTTCGCTCCCCATGCTTTCGCTCCTCAG | 617 | 1.5939857393820398 | No Hit |
| GACTACAAGGGTATCTAATCCTGTTTGCTCCCCACGCTTTCGCACCTCAG | 601 | 1.5526506148599772 | No Hit |
| GACTACCAGGGTATCTAATCCTGTTTGCTCCCCACGCTTTCGCACCTCAG | 599 | 1.5474837242947193 | No Hit |
| GACTACCCGGGTATCTAATCCTGTTTGCTCCCCACGCTTTCGCACCTCAG | 592 | 1.529399607316317 | No Hit |
| GACTACACGGGTATCTAATCCTGTTTGCTCCCCACGCTTTCGCACCTCAG | 525 | 1.3563087733801797 | No Hit |
| GACTACTGGGGTATCTAATCCTGTTTGCTCCCCACGCTTTCGCGCCTCAG | 396 | 1.0230443319210498 | No Hit |
| GACTACTAGGGTATCTAATCCTGTTTGCTCCCCACGCTTTCGCGCCTCAG | 380 | 0.9817092073989873 | No Hit |
| GACTACCGGGGTATCTAATCCTGTTTGCTCCCCACGCTTTCGCGCCTCAG | 376 | 0.9713754262684716 | No Hit |
| GACTACAGGGGTATCTAATCCTGTTTGCTCCCCACGCTTTCGCGCCTCAG | 351 | 0.9067892942027488 | No Hit |
| GACTACTCGGGTATCTAATCCTGTTTGCTCCCCACGCTTTCGCGCCTCAG | 344 | 0.8887051772243464 | No Hit |
| GACTACAAGGGTATCTAATCCTGTTTGCTCCCCACGCTTTCGCGCCTCAG | 307 | 0.7931177017670765 | No Hit |
| GACTACACGGGTATCTAATCCTGTTTGCTCCCCACGCTTTCGCGCCTCAG | 301 | 0.7776170300713031 | No Hit |
| GACTACCAGGGTATCTAATCCTGTTTGCTCCCCACGCTTTCGCGCCTCAG | 293 | 0.7569494678102717 | No Hit |
| GACTACCCGGGTATCTAATCCTGTTTGCTCCCCACGCTTTCGCGCCTCAG | 290 | 0.7491991319623851 | No Hit |
| GACTACTGGGGTATCTAATCCTGTTTGATCCCCACGCTTTCGCACATCAG | 282 | 0.7285315697013537 | No Hit |
| GACTACAGGGGTATCTAATCCTGTTTGATCCCCACGCTTTCGCACATCAG | 259 | 0.6691123282008887 | No Hit |
| GACTACTCGGGTATCTAATCCTGTTTGCTCCCCACGCTTTCGTGCATGAG | 252 | 0.6510282112224863 | No Hit |
| GACTACTCGGGTATCTAATCCTGTTTGCTCCCCATGCTTTCGTACCTCAG | 251 | 0.6484447659398573 | No Hit |
| GACTACAGGGGTATCTAATCCTGTTTGCTCCCCATGCTTTCGTACCTCAG | 250 | 0.6458613206572285 | No Hit |
| GACTACTGGGGTATCTAATCCTGTTTGCTCCCCACGCTTTCGTGCATGAG | 240 | 0.6200268678309393 | No Hit |
| GACTACTAGGGTATCTAATCCTGTTTGCTCCCCACGCTTTCGTGCATGAG | 238 | 0.6148599772656815 | No Hit |
| GACTACTAGGGTATCTAATCCTGTTTGATCCCCACGCTTTCGCACATCAG | 228 | 0.5890255244393924 | No Hit |
| GACTACCAGGGTATCTAATCCTGTTTGCTCCCCACGCTTTCGTGCATGAG | 227 | 0.5864420791567635 | No Hit |
| GACTACAAGGGTATCTAATCCTGTTTGATCCCCACGCTTTCGCACATCAG | 227 | 0.5864420791567635 | No Hit |
| GACTACAGGGGTATCTAATCCTGTTTGCTCCCCACGCTTTCGTGCATGAG | 227 | 0.5864420791567635 | No Hit |
| GACTACCAGGGTATCTAATCCTGTTTGATCCCCACGCTTTCGCACATCAG | 226 | 0.5838586338741345 | No Hit |
| GACTACTAGGGTATCTAATCCTGTTTGCTCCCCATGCTTTCGTACCTCAG | 223 | 0.5761082980262477 | No Hit |
| GACTACTGGGGTATCTAATCCTGTTTGCTCCCCATGCTTTCGTACCTCAG | 222 | 0.5735248527436189 | No Hit |
| GACTACTCGGGTATCTAATCCTGTTTGATCCCCACGCTTTCGCACATCAG | 219 | 0.5657745168957322 | No Hit |
| GACTACCGGGGTATCTAATCCTGTTTGATCCCCACGCTTTCGCACATCAG | 216 | 0.5580241810478453 | No Hit |
| GACTACCGGGGTATCTAATCCTGTTTGCTCCCCACGCTTTCGTGCATGAG | 215 | 0.5554407357652165 | No Hit |
| GACTACCAGGGTATCTAATCCTGTTTGCTCCCCATGCTTTCGTACCTCAG | 208 | 0.5373566187868141 | No Hit |
| GACTACCCGGGTATCTAATCCTGTTTGATCCCCACGCTTTCGCACATCAG | 206 | 0.5321897282215562 | No Hit |
| GACTACCCGGGTATCTAATCCTGTTTGCTCCCCACGCTTTCGTGCATGAG | 200 | 0.5166890565257828 | No Hit |
| GACTACAAGGGTATCTAATCCTGTTTGCTCCCCATGCTTTCGTACCTCAG | 194 | 0.5011883848300094 | No Hit |
| GACTACCGGGGTATCTAATCCTGTTTGCTCCCCATGCTTTCGTACCTCAG | 190 | 0.49085460369949363 | No Hit |
| GACTACAAGGGTATCTAATCCTGTTTGCTCCCCACGCTTTCGTGCATGAG | 190 | 0.49085460369949363 | No Hit |
| GACTACTGGGGTATCTAATCCTGTTCGCTCCCCACGCTTTCGCTCCTCAG | 186 | 0.48052082256897805 | No Hit |
| GACTACACGGGTATCTAATCCTGTTTGATCCCCACGCTTTCGCACATCAG | 184 | 0.47535393200372017 | No Hit |
| GACTACACGGGTATCTAATCCTGTTTGCTCCCCACGCTTTCGTGCATGAG | 182 | 0.47018704143846235 | No Hit |
| GACTACCCGGGTATCTAATCCTGTTTGCTCCCCATGCTTTCGTACCTCAG | 174 | 0.449519479177431 | No Hit |
| GACTACACGGGTATCTAATCCTGTTTGCTCCCCATGCTTTCGTACCTCAG | 171 | 0.44176914332954426 | No Hit |
| GACTACTGGGGTATCTAATCCTGTTCGCTACCCATGCTTTCGCTCCTCAG | 158 | 0.4081843546553684 | No Hit |
| GACTACTCGGGTATCTAATCCTGTTCGCTCCCCACGCTTTCGCTCCTCAG | 158 | 0.4081843546553684 | No Hit |
| GACTACTAGGGTATCTAATCCTGTTCGCTCCCCACGCTTTCGCTCCTCAG | 156 | 0.4030174640901106 | No Hit |
| GACTACCGGGGTATCTAATCCTGTTCGCTCCCCACGCTTTCGCTCCTCAG | 156 | 0.4030174640901106 | No Hit |
| GACTACAGGGGTATCTAATCCTGTTCGCTCCCCACGCTTTCGCTCCTCAG | 148 | 0.38234990182907924 | No Hit |
| GACTACCAGGGTATCTAATCCTGTTCGCTCCCCACGCTTTCGCTCCTCAG | 147 | 0.37976645654645036 | No Hit |
| GACTACTGGGGTATCTAATCCTGTTTGCTCCCCACGCTTTCGTGCCTCAG | 146 | 0.3771830112638214 | No Hit |
| GACTACCAGGGTATCTAATCCTGTTTGCTCCCCACGCTTTCGTGCCTCAG | 145 | 0.37459956598119254 | No Hit |
| GACTACAGGGGTATCTAATCCTGTTTGCTCCCCACGCTTTCGTGCCTCAG | 145 | 0.37459956598119254 | No Hit |
| GACTACACGGGTATCTAATCCTGTTCGCTCCCCACGCTTTCGCTCCTCAG | 138 | 0.3565154490027901 | No Hit |
| GACTACTGGGGTATCTAATCCTGTTTGCTCCCCATGCTTTCGCACCTCAG | 136 | 0.3513485584375323 | No Hit |
| GACTACTGGGGTATCTAATCCTGTTCGCTCCCCATGCTTTCGCTTCTCAG | 136 | 0.3513485584375323 | No Hit |
| GACTACCCGGGTATCTAATCCTGTTCGCTCCCCACGCTTTCGCTCCTCAG | 135 | 0.3487651131549034 | No Hit |
| GACTACCGGGGTATCTAATCCTGTTCGCTACCCATGCTTTCGCTCCTCAG | 133 | 0.34359822258964556 | No Hit |
| GACTACTAGGGTATCTAATCCTGTTCGCTACCCATGCTTTCGCTCCTCAG | 131 | 0.33843133202438774 | No Hit |
| GACTACAAGGGTATCTAATCCTGTTCGCTCCCCACGCTTTCGCTCCTCAG | 127 | 0.3280975508938721 | No Hit |
| GACTACTAGGGTATCTAATCCTGTTTGCTCCCCACGCTTTCGTGCCTCAG | 127 | 0.3280975508938721 | No Hit |
| GACTACAGGGGTATCTAATCCTGTTCGCTACCCATGCTTTCGCTCCTCAG | 127 | 0.3280975508938721 | No Hit |
| GACTACAGGGGTATCTAATCCTGTTTGCTCCCCACGCTTTCGCACCTGAG | 126 | 0.32551410561124317 | No Hit |
| GACTACCGGGGTATCTAATCCTGTTTGCTCCCCACGCTTTCGTGCCTCAG | 121 | 0.3125968791980986 | No Hit |
| GACTACTAGGGTATCTAATCCTGTTTGCTCCCCATGCTTTCGCACCTCAG | 119 | 0.30742998863284077 | No Hit |
| GACTACAAGGGTATCTAATCCTGTTTGCTCCCCACGCTTTCGTGCCTCAG | 118 | 0.30484654335021183 | No Hit |
| GACTACAGGGGTATCTAATCCTGTTTGCTCCCCATGCTTTCGCACCTCAG | 114 | 0.2945127622196962 | No Hit |
| GACTACTCGGGTATCTAATCCTGTTTGCTCCCCACGCTTTCGTGCCTCAG | 114 | 0.2945127622196962 | No Hit |
| GACTACCCGGGTATCTAATCCTGTTTGCTCCCCACGCTTTCGCACCTGAG | 112 | 0.28934587165443837 | No Hit |
| GACTACAAGGGTATCTAATCCTGTTTGCTCCCCACGCTTTCGCACCTGAG | 110 | 0.28417898108918055 | No Hit |
| GACTACTCGGGTATCTAATCCTGTTCGCTACCCATGCTTTCGCTCCTCAG | 110 | 0.28417898108918055 | No Hit |
| GACTACAAGGGTATCTAATCCTGTTCGCTACCCATGCTTTCGCTCCTCAG | 107 | 0.2764286452412938 | No Hit |
| GACTACCCGGGTATCTAATCCTGTTTGCTCCCCACGCTTTCGTGCCTCAG | 107 | 0.2764286452412938 | No Hit |
| GACTACCGGGGTATCTAATCCTGTTTGCTCCCCATGCTTTCGCACCTCAG | 106 | 0.2738451999586649 | No Hit |
| GACTACTAGGGTATCTAATCCTGTTTGCTCCCCACGCTTTCGCACCTGAG | 105 | 0.27126175467603597 | No Hit |
| GACTACAGGGGTATCTAATCCTGTTCGCTCCCCATGCTTTCGCTTCTCAG | 103 | 0.2660948641107781 | No Hit |
| GACTACTGGGGTATCTAATCCTGTTCGCTCCCCACACTTTCGCTCCTCAG | 103 | 0.2660948641107781 | No Hit |
| GACTACTCGGGTATCTAATCCTGTTCGCTCCCCACACTTTCGCTCCTCAG | 103 | 0.2660948641107781 | No Hit |
| GACTACCAGGGTATCTAATCCTGTTCGCTCCCCATGCTTTCGCTTCTCAG | 102 | 0.2635114188281492 | No Hit |
| GACTACTAGGGTATCTAATCCTGTTCGCTCCCCACACTTTCGCTCCTCAG | 102 | 0.2635114188281492 | No Hit |
| GACTACAAGGGTATCTAATCCTGTTCGCTCCCCATGCTTTCGCTTCTCAG | 101 | 0.26092797354552033 | No Hit |
| GACTACCAGGGTATCTAATCCTGTTTGCTCCCCATGCTTTCGCACCTCAG | 101 | 0.26092797354552033 | No Hit |
| GACTACCAGGGTATCTAATCCTGTTCGCTCCCCACACTTTCGCTCCTCAG | 100 | 0.2583445282628914 | No Hit |
| GACTACCAGGGTATCTAATCCTGTTCGCTACCCATGCTTTCGCTCCTCAG | 98 | 0.2531776376976336 | No Hit |
| GACTACACGGGTATCTAATCCTGTTTGCTCCCCACGCTTTCGTGCCTCAG | 97 | 0.2505941924150047 | No Hit |
| GACTACCGGGGTATCTAATCCTGTTTGCTCCCCACGCTTTCGCACCTGAG | 96 | 0.24801074713237575 | No Hit |
| GACTACTAGGGTATCTAATCCTGTTCGCTCCCCATGCTTTCGCTTCTCAG | 95 | 0.24542730184974682 | No Hit |
| GACTACTCGGGTATCTAATCCTGTTTGCTCCCCATGCTTTCGCACCTCAG | 95 | 0.24542730184974682 | No Hit |
| GACTACAAGGGTATCTAATCCTGTTTGCTCCCCATGCTTTCGCACCTCAG | 89 | 0.22992663015397333 | No Hit |
| GACTACTCGGGTATCTAATCCTGTTTGCTCCCCACGCTTTCGCACCTGAG | 87 | 0.2247597395887155 | No Hit |
| GACTACTCGGGTATCTAATCCTGTTCGCTCCCCATGCTTTCGCTTCTCAG | 86 | 0.2221762943060866 | No Hit |
| GACTACACGGGTATCTAATCCTGTTCGCTACCCATGCTTTCGCTCCTCAG | 86 | 0.2221762943060866 | No Hit |
| GACTACTGGGGTATCTAATCCTGTTTGCTCCCCACGCTTTCGCACCTGAG | 85 | 0.21959284902345766 | No Hit |
| GACTACCCGGGTATCTAATCCTGTTTGCTCCCCATGCTTTCGCACCTCAG | 85 | 0.21959284902345766 | No Hit |
| GACTACTGGGGTATCTAATCCTGTTTGCTCCCCACGCTTTCGAGCCTCAG | 85 | 0.21959284902345766 | No Hit |
| GACTACCCGGGTATCTAATCCTGTTCGCTACCCATGCTTTCGCTCCTCAG | 84 | 0.21700940374082878 | No Hit |
| GACTACAAGGGTATCTAATCCTGTTCGCTCCCCACACTTTCGCTCCTCAG | 83 | 0.21442595845819987 | No Hit |
| GACTACCAGGGTATCTAATCCTGTTTGCTCCCCACGCTTTCGCACCTGAG | 83 | 0.21442595845819987 | No Hit |
| GACTACCGGGGTATCTAATCCTGTTCGCTCCCCACACTTTCGCTCCTCAG | 81 | 0.20925906789294202 | No Hit |
| GACTACAGGGGTATCTAATCCTGTTTGCTCCCCACGCTTTCGAGCCTCAG | 80 | 0.20667562261031314 | No Hit |
| GACTACACGGGTATCTAATCCTGTTTGCTCCCCATGCTTTCGCACCTCAG | 78 | 0.2015087320450553 | No Hit |
| GACTACACGGGTATCTAATCCTGTTTGCTCCCCACGCTTTCGCACCTGAG | 77 | 0.19892528676242635 | No Hit |
| GACTACCGGGGTATCTAATCCTGTTCGCTCCCCATGCTTTCGCTTCTCAG | 77 | 0.19892528676242635 | No Hit |
| GACTACAAGGGTATCTAATCCTGTTTGCTCCCCACGCTTTCGAGCCTCAG | 75 | 0.19375839619716853 | No Hit |
| GACTACACGGGTATCTAATCCTGTTCGCTCCCCATGCTTTCGCTTCTCAG | 75 | 0.19375839619716853 | No Hit |
| GACTACAGGGGTATCTAATCCTGTTCGCTCCCCACACTTTCGCTCCTCAG | 74 | 0.19117495091453962 | No Hit |
| GACTACTCGGGTATCTAATCCTGTTTGCTCCCCACGCTTTCGAGCCTCAG | 73 | 0.1885915056319107 | No Hit |
| GACTACACGGGTATCTAATCCTGTTCGCTCCCCACACTTTCGCTCCTCAG | 72 | 0.1860080603492818 | No Hit |
| GACTACCGGGGTATCTAATCCTGTTTGCTCCCCACGCTTTCGAGCCTCAG | 70 | 0.18084116978402398 | No Hit |
| GACTACCAGGGTATCTAATCCTGTTTGCTCCCCACGCTTTCGAGCCTCAG | 69 | 0.17825772450139504 | No Hit |
| GACTACTAGGGTATCTAATCCTGTTTGCTCCCCACGCTTTCGAGCCTCAG | 69 | 0.17825772450139504 | No Hit |
| GACTACCCGGGTATCTAATCCTGTTCGCTCCCCACACTTTCGCTCCTCAG | 68 | 0.17567427921876616 | No Hit |
| GACTACCCGGGTATCTAATCCTGTTCGCTCCCCATGCTTTCGCTTCTCAG | 67 | 0.17309083393613722 | No Hit |
| GACTACACGGGTATCTAATCCTGTTTGCTCCCCACGCTTTCGAGCCTCAG | 64 | 0.1653404980882505 | No Hit |
| GACTACTGGGGTATCTAATCCTGTTTGCTACCCACGCTTTCGAATCTCAG | 63 | 0.16275705280562158 | No Hit |
| GACTACTCGGGTATCTAATCCTGTTTGCTCCCCACGCTGTCGCGCCTCAG | 61 | 0.15759016224036376 | No Hit |
| GACTACTGGGGTATCTAATCCTGTTTGCTACCCACGCTTTCGAACCTCAG | 59 | 0.15242327167510591 | No Hit |
| GACTACTGGGGTATCTAATCCTGTTCGCTCCCCACGCTTTCGTGCCTCAG | 58 | 0.149839826392477 | No Hit |
| GACTACCAGGGTATCTAATCCTGTTTGCTCCCCACGCTGTCGCGCCTCAG | 57 | 0.1472563811098481 | No Hit |
| GACTACTAGGGTATCTAATCCTGTTTGCTACCCACGCTTTCGAACCTCAG | 56 | 0.14467293582721918 | No Hit |
| GACTACCAGGGTATCTAATCCTGTTTGCTACCCACGCTTTCGAACCTCAG | 55 | 0.14208949054459027 | No Hit |
| GACTACAGGGGTATCTAATCCTGTTTGCTACCCACGCTTTCGAACCTCAG | 55 | 0.14208949054459027 | No Hit |
| GACTACTCGGGTATCTAATCCTGTTTGCTACCCACGCTTTCGAACCTCAG | 54 | 0.13950604526196134 | No Hit |
| GACTACCCGGGTATCTAATCCTGTTTGCTCCCCACGCTTTCGAGCCTCAG | 51 | 0.1317557094140746 | No Hit |
| GACTACCGGGGTATCTAATCCTGTTTGCTACCCACGCTTTCGAACCTCAG | 51 | 0.1317557094140746 | No Hit |
| GCTGCGTTCTTCATCGATGCCGGAACCAAGAGATCCATTGTTGAAAGTTT | 50 | 0.1291722641314457 | No Hit |
| GACTACTAGGGTATCTAATCCTGTTTGCTCCCCACGCTGTCGCGCCTCAG | 49 | 0.1265888188488168 | No Hit |
| GACTACCGGGGTATCTAATCCTGTTTGCTCCCCACGCTGTCGCGCCTCAG | 48 | 0.12400537356618788 | No Hit |
| GACTACCGGGGTATCTAATCCTGTTTGCTACCCACGCTTTCGAATCTCAG | 48 | 0.12400537356618788 | No Hit |
| GACTACAAGGGTATCTAATCCTGTTTGCTACCCACGCTTTCGAACCTCAG | 47 | 0.12142192828355895 | No Hit |
| GACTACTGGGGTATCTAAGCCTGTTCGCTCCCCACGCTTTCGCTCCTCAG | 46 | 0.11883848300093004 | No Hit |
| GACTACAGGGGTATCTAATCCTGTTCGCTCCCCACGCTTTCGCTTCTCAG | 46 | 0.11883848300093004 | No Hit |
| GACTACCCGGGTATCTAATCCTGTTCGCTCCCCACGCTTTCGTGCCTCAG | 46 | 0.11883848300093004 | No Hit |
| GACTACTAGGGTATCTAATCCTGTTCGCTCCCCACGCTTTCGTGCCTCAG | 46 | 0.11883848300093004 | No Hit |
| GACTACAAGGGTATCTAATCCTGTTTGCTCCCCACGCTGTCGCGCCTCAG | 45 | 0.11625503771830112 | No Hit |
| GACTACCAGGGTATCTAATCCTGTTTGCTACCCACGCTTTCGAATCTCAG | 45 | 0.11625503771830112 | No Hit |
| GACTACAGGGGTATCTAATCCTGTTTGCTCCCCACGCTGTCGCGCCTCAG | 45 | 0.11625503771830112 | No Hit |
| GACTACTGGGGTATCTAATCCGGTTCGCTCCCCACACTTTCGCGCCTCAG | 45 | 0.11625503771830112 | No Hit |
| GACTACCAGGGTATCTAATCCTGTTCGCTCCCCACGCTTTCGTGCCTCAG | 44 | 0.11367159243567222 | No Hit |
| GACTACCCGGGTATCTAATCCTGTTTGCTCCCCACGCTGTCGCGCCTCAG | 44 | 0.11367159243567222 | No Hit |
| GACTACTCGGGTATCTAATCCTGTTTGCTACCCACGCTTTCGAATCTCAG | 44 | 0.11367159243567222 | No Hit |
| GACTACACGGGTATCTAATCCTGTTTGCTACCCACGCTTTCGAACCTCAG | 43 | 0.1110881471530433 | No Hit |
| GACTACCCGGGTATCTAATCCTGTTTGCTACCCACGCTTTCGAACCTCAG | 43 | 0.1110881471530433 | No Hit |
| GACTACTCGGGTATCTAATCCTGTTCGCTCCCCACGCTTTCGTGCCTCAG | 42 | 0.10850470187041439 | No Hit |
| GACTACTGGGGTATCTAATCCTGTTTGCTCCCCACGCTGTCGCGCCTCAG | 42 | 0.10850470187041439 | No Hit |
| GACTACTAGGGTATCTAATCCTGTTCGCTCCCCACGCTTTCGCTTCTCAG | 40 | 0.10333781130515657 | No Hit |
| GGGGGGGGGGGGGGGGGGGGGGGGGGGGGGGGGGGGGGGGGGGGGGGGGG | 40 | 0.10333781130515657 | No Hit |
| GACTACACGGGTATCTAATCCTGTTTGCTCCCCACGCTGTCGCGCCTCAG | 40 | 0.10333781130515657 | No Hit |
| GACTACTGGGGTATCTAATCCTGTTCGCTCCCCACGCTTTCGCTTCTCAG | 39 | 0.10075436602252764 | No Hit |
| GACTACAAGGGTATCTAATCCTGTTCGCTCCCCACGCTTTCGCTTCTCAG | 39 | 0.10075436602252764 | No Hit |

## Adapter Content

## Kmer Content

| Sequence | Count | PValue | Obs/Exp Max | Max Obs/Exp Position |
| --- | --- | --- | --- | --- |
| TTAGACG | 5 | 1.7766611E-4 | 6689.441 | 295 |
| GTTAGGG | 5 | 1.7766611E-4 | 6689.441 | 295 |
| GTTAGCG | 20 | 0.0 | 6689.441 | 295 |
| GTTAGCA | 10 | 6.665141E-8 | 6689.441 | 295 |
| TTAGCCG | 110 | 0.0 | 5777.2446 | 295 |
| CTTAGCG | 10 | 7.105978E-4 | 3344.7205 | 295 |
| ATAGACG | 10 | 7.105978E-4 | 3344.7205 | 295 |
| TTTAGCG | 15 | 0.001598695 | 2229.8137 | 295 |
| TTTGACG | 20 | 0.002841858 | 1672.3602 | 295 |
| AGTTAGG | 65 | 0.0 | 1029.1448 | 295 |
| GACTACT | 1410 | 0.0 | 293.85144 | 1 |
| GACTACC | 1210 | 0.0 | 293.85144 | 1 |
| AGGGGTA | 505 | 0.0 | 293.8514 | 7 |
| CTACAGG | 505 | 0.0 | 293.8514 | 3 |
| TGCGTTC | 15 | 7.299326E-6 | 293.8514 | 3 |
| CTGGGGT | 540 | 0.0 | 293.8514 | 6 |
| AAGGGTA | 380 | 0.0 | 293.8514 | 7 |
| TACACGG | 280 | 0.0 | 293.8514 | 4 |
| CTAGGGT | 460 | 0.0 | 293.8514 | 6 |
| CTGCGTT | 15 | 7.299326E-6 | 293.8514 | 2 |

Produced by FastQC (version 0.11.7)
