# Supplementary material for: Assessment of airborne bacteria from a public health institution in Mexico City
Source: PLOS Glob Public Health. 2024 Nov 7;4(11):e0003672. doi: 10.1371/journal.pgph.0003672 (PMC11542838; doi:10.1371/journal.pgph.0003672)
Supplement: S1 Fig — (ZIP) [file pgph.0003672.s002.zip › 16S.krona english.html]

Javascript must be enabled to view this page.

magnitude
magnitudeUnassigned

AVPS\_OH\_day\_1
AVPS\_OH\_day\_2
AVPS\_OH\_day\_3
AVPS\_F1\_day\_1
AVPS\_F1\_day\_2
AVPS\_F1\_day\_3
AVPS\_F2\_day\_1
AVPS\_F2\_day\_2
AVPS\_F2\_day\_3
AVPS\_EU\_day\_1
AVPS\_EU\_day\_2
CuS\_OH\_day\_1
CuS\_OH\_day\_2
CuS\_OH\_day\_3
CuS\_F1\_day\_1
CuS\_F1\_day\_2
CuS\_F1\_day\_3
CuS\_F2\_day\_1
CuS\_F2\_day\_2
CuS\_F2\_day\_3
CuS\_EU\_day\_1
CuS\_EU\_day\_2

105229311843431055532897384875491908381877236882140111836411224851103203172403435940706323033084628297799991371716236504131512436537610

651533111447116415519291071094917113216

111111

111111

1

1

1

11111

11111

11111

641533010447116415519281061094817113216

6112112113412

6112112113412

6112112113412

6112112113412

491502994361142150151743634816103212

41711125122533

13

1

3

11

11

21711125122493

14111111

1148

13115212

361152162647131106634840128259

361152162647131106634840128259

361152162647131106634840128259

9341216122439799281273

9341216122439799281273

9341216122439799281273

41114160112

41114160112

41114160112

160

4112

1411

239

239

239

5

1

1

34

32112

32112

2112

2112

111

111

105222811841901055501897373875447908374877225882140111836411224211103048172403435940704423032794627227798901371226236334131402433337594

66121133221123634291

66121133221123634291

14105

13105

13105

1

1

6681132221113210191

6681132221113210191

421111828

2481321211221911

2

2

2

11

11

11

1136

1136

1136

111296105

111296105

111296105

21102

21102

996

996

113

113

84212213195643285091337568042171354301973151418

84212213195643285091337568042171354301973151418

54212213195643285091337568042171353541973149416

1858267410571105019173691699

1858267410571105019173691699

54212213177135024350331856992171244371604133317

54212213177135024350331856992171244371604133317

37622

37622

37622

3368121101530425440210172856137119112833641

171632

171632

171632

10

71632

3368121101530425440210002840137115912833641

41211

41211

1

4121

2958101101530425440210002839137115912833640

2958101101530425440210002839137115912833640

322492111

3221162081554881230

7121

116133820721

631766213413823456524523

6312111133723321026824831

3417169702294331351285237181

311718017420412

116511217212338631

4579

4579

4579

4579

4579

501275123391565139713319611976603544731941539364596421200239

452121396151516207326453489639673872161151

32449463366130727125192218584360

32449463366130727125192218584360

32449463366130727125192218584360

1321294710521314119572636912045201411891

1321294710521314119572636912045201411891

1321294710521314119572636912045201411891

211335218932518355

211335218932518355

211335218932518355

211335218932518355

311194800208

1

1

1

31194800208

3735

3735

31157800173

31157800173

38362

38362

38362

38362

11135831111031145350425214462156179

1113572051103114329475014361855179

11304126196101128565014321854179

95

294442208481811251

111012211969264821414729178

121

2

11

153796132912

153796132912

1106255720210301

1106255720210301

1106255720210301

510422128182116934574

510422128182116934574

510422128182116934574

132111

410422125162116834563

381164218371463118611517197394363106391861349

381164218371463118611517197394363106391861349

381164218371463118611517197394363106391861349

14143214231121162

11171424331691479757249255331

1024151025131321532219951071165

11213121111

157012625344318891529821531233

28117122120471398152268137810734938

28117122120471398152268137810734938

28117122120471398152268137810734938

1326025267361010681820

275

132602526735834881815

58

1511512260471146849712722552938

1272102

3

51512260471146577711672552938

1

31142413373423404273253530517929582647431126

511411223128140910896559435631120

511411223128140910896559435631120

12

12

112231281421086965943565118

112231281421086965943565118

413111267269242

413111267269242

17117624322473116955614

17106514322473116955614

17106514322473116955614

17106514322473116955614

1111

1111

11

11

9216333011771451126124711227572

9216333011771451126124711227572

9216333011771451126124711227572

9113323011701428562

5713093161

11164

18914563771

3311114434

61223107225399115735411

61223107225399115735411

61223107225399115735411

112151

112151

512131071489911573541

514861176251

1310738811031

111

617711921431

617711921431

617711921431

617711921431

617711921431

745331481153

745331481153

745331481153

335231471153

313

2147

14111153

1

1

11

11

4

3

1

533111451234116255770816115566851

533111451234116255770816115566851

533111451234116255770816115566851

533111451234116255770816115566851

533111451234116255770816115566851

210525123

210525123

210525123

11

11

19525123

12

1

1942323

2111111

2111111

2111111

2111111

2111111

113526027142613281472177109917971946147848161450166

113526027142613281472177109917971946147848161450166

492453121026112141216169771265114

492453121026112141216169771265114

11

11

31121

3121151

217

2121

4457

1

11

11

11111

56448

382051782620312124514264110

2411655

2411655

2411655

262232323431293106144257

262232323431293106144257

291

37

332111

1

1

617122122511

1

1

13122

4111

7

101116155

1

37641214685068442423794372

36341114455024423137918

133213459215611111

1

23

11143265

1

4124411

1

34997

5

1312366114264

11111

1313654264

1169152191072587840658871423

6963837267401

6963837267401

111521272688611657871423

961862335276874723

1156186455845891244

111211231

111211231

111211231

111211231

1121

11123

31075950746228724317048224835012475625767356216609835095960280881584960471849587842180257397299572242578861384581227416580

247731378149441362339830734114867391590071376126563351944746059585619676166608402277

2225615172325357531914048931119832321

2225615172325357531914048931119832321

1

11211533

7421321244221131

157225107024938910

11421167557529641734918684399

683837466212445125722244244489518948739021136454404791540143256

683837466212445125722244244489518948739021136454404791540143256

204121236533341109334532044944792

2813733941851630715410428037610614

71844323110711728411479160179232

612253121

3626210113183217914629912105468195232115

4838131139311410384086181810319010811210912

2141122133292343465314043

268231226202

12145233311

15131211178117511

9821342141128174348218412

14568919261121939191156183132681211

11711181871631661176411

321

4410221111151

2201575257111068799331792

305771532571283010214091035479213413775

305771532571283010214091035479213413775

1

13030879114516

5211422521127152984671071712

114171732

15215

3

1

1181

11

26

111741

22716435

15355

18681234473554457

1

16212182341331822942294603691

16212182341331822942294603691

399

11

11117134118991

511

11132239022136125

1158165190

53

53

53

2210117834675752112225340159191150141

1270932835563181222434015217115014

5261113112274113

7683227255321712222661521614714

1031856321231721

821524121131411

212

28332211

24035355100418512126223011014336415581051358315251101411436878459617404140455141853

235865663286610113339195766951442280889312692023891198694211251075

911111

431381286180445

2319742521913511282517538090783226748089227522314115537206109885

1111

2

2441221212269291513273533862248164010185

472111554227221

2110651651131

11

211

111531

45525311311141121211442

374112111

11

11

435278918151111253209107831911915973804104198340367464179240073205299700

51

151221310131229117

51

5331616771206180

7258311242191516213426935664154212718802166692655081

9453216227111177419731453

9610122126832575052358446392547264454

746910120253212015712211619592049115

4128117532078825053291661484261701644

3111

285175841199

141111

1223521131

15141353261622147151

215721322121

3311705223512651

11111103233149753211156

1111201062

23611123021118113012927266253

111111

131121211271012212

816427126214433

1843466631114245471598724292165620142255

12113552319020913124

11146462533211582168158

15

4311612437132411545

1113115485

41

105867228282134284269299472427823

14171288127252114364

263122742555111532167874082302987356

31112214131931

331220131286426166710520137825627118

122711123126322311

531654441411861

66111212254129169108871

7672120201137328413331382

2111211111

1111141251185

2212212621

116873

1112

5154211318111111418

1

121

1331712216313

111115521102264733154455536130421646310031872

23

1

11221121

49721215235388461268544472521371

3121113772494708346717365

21412132241

31219637108

11716612561

3108122113911122646111190199947415726

232112811829610253868189471

33492

1

26

2111214526525762102

15

11

3211011118

12111391

171

1061

4962285231668217921221159723654078502071632554074871230268261231368197858040917255

87849334232750523461068195632719921451330136345014485109492271008

341132230818663881490248461911929684087

1

111111110787291410184642205543013

828541146

23121111510793157214651559722681074

31411672

14

3167

2

1

1

1

11471552172847211

11471552172847211

1114593083939715237391

1114593083939715237391

7933902872446522468110742724171112328267922720130679545187826

2219732421561532637

136218266115648560992312047956151401930107505299105659

285449110222115946587190169911961681079137413109

2910198148232304353131423993965446106228121855

4111104112261704191

2124324141731127257417341011

31

416276341

1101109

1101109

1133

1133

54641511137314623

22661

1

1111

54241213612613

1

11

4335111216211614273513324317

1

1

24411028

24411028

111801

178

111

1

1

111

111

311511521891

311511521891

313138221

313138221

1

1

613194

613194

1121

1121

532114041553

31131352

2311401201

11

11

11

11

11

11

226352276112788294487313413815153464014154270484231159181124731661282

277224

119

158224

12315424226041128296107837321092156112745116

1511

224516975317377117473

1771213822

7

1112225

21317397

31632834510448

111

7114141332666399926511081452953263

1141138

21871304181

31113147111110824681

1130

3131471691413201

1118511

40917

314132511732422637

11

1222662

221211844

12125422763

1

4372122421874749562614055402125171677682848931083164

21116601118468910832411516107719374016

81

435212141170873838227164238101061161575129561043148

1115158111004382231241192

10211

111

3512552395

114211199182227241141

8633199527387797991155

6991205

442799426182534991155

1100263

42

13018722819291

1321093

3082

7122587

1103111

3621683621612

3621683621612

1722147485151311324074256282105813064781606113024375

7173

17014146504631121314104601545952

114212846211264704145671

211

11115

1113053786276688907407113299923522

121354241

1

1

123191248112140151149882629356411981456238468672

21124583291

19

211505691002501355001139

2112111511963151039671936195811471317178857629

32264121222

11

111

31413163121

917151510922454229293610892312290236815352871657161

112

613442111521562273241651728

31612156422352228178610681746220534213281131657153

14423242

22212002413911220099

1197

22220024139112399

47113235533642208468738946115191926

111240130

16913261

251144433212072823389223517621

1

175

11117

1111061

1

1

111

11108311158321401723

11112092751142853671

11260531

147

1

6059661

1105

1

1127514

53911324545152472112762

53911322545152472112762

2

11

11

11

564432241514122381720645241905112245280901346531350770330398585063401

564432241514122381720645241905112245280901346531350770330398585063401

97

3122231787870186835759998102354778143

111096714051829348551159

311

108

1

1

11311

2

43

1

1

31

8341135113071976226603225162471381

112111118255236041216565251516169562

32171134201621

1

31

1

21113

3392417110635101668319684429628208341135111024454619285533462651

1

1

1312252

11114414011116

23

1

81411111235483282610743572142079915195532012248

222146812201411831092195566312349475683219281390108169

221846812191411831092195466312349475583219271389108169

13217449727817462413182213574508891202275

415191786220665622024823013236103711379162

5110312111613651164538114617422

10518714

311111

2

1111

1

1

61401202820395245289182476348245916156223293941023415514812811377

3111111131

3111111131

1182

157

125

22224980111983169314114

22224980111983169314114

111358349976191111855743593110383

111358349976191111855743593110383

3233521217211412539114414101014074750981499219222488835787

14331711491857441455253447431

11573

1

11

11921811232912271611355

31271521

108

121111

3

1112410651

12711

111101

526122127011

2

41822311832854812413962752297817814358427718

13073834634918491115565566

111

1111

1233110535

111

206181235253415164864298123453858520953252010386828242505

144812222448373823538779252995761103415142812697

111062

11395107112029111821261056318

11

111294017813539350104237035

11391356732611011

1

16832502151127121

311159030760

1100364434635585385408260620153277

222923131581

19711

211211177287421891191713227411322788534

21

8221226366

18113029275

11124

111

14

6147111121257242182422151

111117121

11

80

13588113912113

4121241161251212

16950619057133471947622228010161664825562116432340133582148119385

1514721172336104139151820737019059475834

41646122672

41646122672

221

221

11147010721359279141819233218957199832

45127

1114701072135927914181473321885719985

11254111319

1

15

6309

541

2

1

21186354

21186354

21186354

627512111837584638080922561416747883200

2102356322256

2102356322256

121211248126455302265

111122293114

1212121643111

46119329640

221132119216614756445731346

221132119216614756445731346

1

1

11354231011918695

334231011908695

1121

3147413133

3147413133

223382294551193883167758

221192163

12311

1200353235957055

171741316072

1

46141

154

11111071936239113741

1071

1119362131174

1126012

173

173

125200

125200

197111455335212

197111455335212

197111455335212

1132312120645193159438543146656

9122118927018438306145

9122118927018438306145

2211313759315102371656

1

121313759315102361656

11

503671517973127551352225414235236861134733221862

46354141595112714834211441219241721104131221622

142164116

11111

45353131594112714833212121976711094131161522

11

11112166

11112166

1156

1156

1

1

41212223311102427935036032240

1

2176234133210

1

4121222113412753493572930

11052

11052

38917452018917115023241421563418604614

21329714810261

241

114

917

11114832

311

36917451918917114723224582416544514

2853535611454100191932613123210

1

1491

83820137511647431321145133

481814164320567932422315345256105553533619

4715342116635421

1

222111

11

112

1

1111

2113

111

1

11133112

1342

4211141533205176274023142552045071063415

1111121922

1212111211

22402211

2216121777341461

1551252325743413244433

242161131471472117145001910

2112212

21189230132433

2113

8923013243

285281505168284766169835247466124033252223153165929349963601372174363030913395554098364411539524756975704070872246589

2811165243077095471

2811165243077095471

11

11955

12111

1

1103

71

1163227709046

11

11

11

1

1

1

5373807386182518619182438262125638134834340120234734826771125751416821941425472

5373807386182518619182438262125638134834340120234734826771125751416821941425472

45

2151895

2

752414510211021229701425415663

222111

2

1

1

6599211212321322034111

2316

207492173133213351121137161119182172451162614

111211

18141746212111

11159

21531001

41

353335447523652887944272

11

11

143355391995053644334771881141501850

43

39174025141024421186256365200610345814561412882727928102079

3

19115332611501732441

1173541131021908612213

131

11

20211132311321

110113852

21

54931125111441446153209041432367956682434656

1

3241211214021224121

13

161311

3424481

342212951122812609209120816221010353414194

2

74

153121112241

4273623715264134333045222275092571124671838420725159308120134527669

1279131

1

28197511704428215216934924666612099225064301016513234862530531111068229241150526115722597648417697727353278049565021

24819486247111316914024495252947147628373604108037256945717518418691150655564424339012828413292665827853727

37712226128911321

310402637112172361033198415380107013042361515183222004461718754308469

163143111220310035609217232247193705114172

163614154338171453104894449956819542274536224717653250

446013158332816994256628766781746259251349077296018243656476507144351

21682185372101016751724157351954135226460410783023272544941565043814484944679327059308316661761822832855

33781307972810392091714680452358817316152824058848366389345064593754601581733094486931406612221711294

25710907244106211

211415018445311602125

427103186

33750307902799222091714680382358617316142124058448354389245064553754541557932910441631404596221681169

14326181211874238851525010811372

112161811121653652502104

112161811121653652502104

3111185413231501061368

41168143106168

1111262

11

2851531

3169615713916114445074732102192441791

11

11

1111

1

11

1

295962513911114445064692092192441756

21

11

17

1241

36

21

15101

27389121884412

1

11094

12

111

41221501445209812291746

21224335

1

1

2121

1

1

112134

1811157367356762311926571531513843

1811157367356762311926571531513843

61

73342

501521193164

18111543648346211815611531352543

3129

137966129253413816411191

137966129253413816411191

1125512925113816411191

3671133

16286028136752339825187958367822891321335711072466336223933988254

125

125

20601243281061312357367252888413314524762149808227738

20601243281061312357367252888413314524762149808227738

3161951124

3161951124

160314131811528256531049148185831603186137710915246

15775625117748174233

2211538159218112804313240

3

1

9

1

4511121511

2911

1151

1214351823597661

5

62

11

1361

1

1

1

1611244102841427

8

122101543

12254

1013

111121

111121

1514951613335120530214111

8322320429741

2

3

11

611293

111

1

11120441

11

11

11141

11141

613916111321111

1

61391611132111

677616257133163598016115463149564523983611452296115418451209244131

3256224972055141212564515483411411964114918021046239122

325622197205414120056451548341139174511391591921239122

107

31112112101125

1

1107102

352554233124156398011110321937852433254316359

352554233124156398011110321937652451759

33242156

2

122047618

122047618

122047618

62111180250278649321

62111178205278648221

1

112124448111

11

121

6961

15

111845

41781931

451

451

21

21

161898940792469152331382921119931732362833123619453109955993455427468

14975693875246772231362921119921546362832104819036102854343155422458

34474391421363251928314109279271763186254441753078137287

21322912521951

1111

1912213812168155111645251017629229012

11

1111

11117

1

134437612152910265101434626194191060639156

533

61111213911171

1214711773

6913112047299271792548219188462543581417

37429221113815463961119813040245813034

11213955111140512516511681706045514396732332

1574

11111111602196

12119253

1214202428121186118841771165300510

1

1434161937313

119318841364158156510

1

41421131

73211142

235229752733378185218128393166711141074124645340279733

1

1

6131

6131

14222710510652102666857501726291954731

1

1

311

3379

1

11

11

14117310510441102646857122725291954630

211

143521924132811111

11122133

142528411158111

1

1

1

1

312711418

312711418

11961

11961

1232

12

32

77195161482267252016212299987105557240522718971

11111

1

331881878750214322271

42411398610545704032251866

1

61160139153181138601111

1

321131111

41

12

26461

112

1

1

1

1

4018227011469974547106217912548846336273

3718026710449871347106117812538746236255

37672964610417510863939035741

121111

5662964610414983838913416

2262473822325

3417320010446911312131674872514

215743121157161

211

2

145237142

34264110723111110511514

323121321111118

323121321111118

1

21121

211

1112211118

11111981

111981

111981

111981

111981

11

11

11

11

1

1

1

1

1

631454261218516943231378617302614115101417317548461875430711436116812149157191220

27331949121683138198322452562234417145240411096994837403097143272

27331949121683138198322452562234417145240411096994837403097143272

1829194611168293818835352562234411144240351096494736632980139269

110533843527412056610438142182

1

1

1

1

12

3

1

82

7515721

991

211211865

1212

1

1

2211111

122191951681226982113152513292494133535546301057187628184

69

1351336

67

8677227132324824757426845

11212215912944340171

1

1

1

1

631

5224820114

111

11

1

1

52593

1021522116832924273966379811810825673515

421226142242112

86

9431211710610657743

742217106106443

111177

1

1

1117

1117

63442412516671733216358724171810072664686967744093627112150

63442412516671733216358724171810072664686967744093627112150

1194681

111

19368

392520719115679223241518262688197053113562709283163104

12512911866121592743

721483125115527937113293223

113112132233339197291

61551504

111251

111110122420970

152201518

9431165521915756

2111214135643

116326101112

127643812281283495142181916

28241691710475211116755491037

11114391314611852832239118011784292

164211

164211

22192865792113117239716319694149032116997944946

7373286111769390213218779731510156501931

131013155

31132125110053433

11

1014112141142

242161

1921324025141364855834931372713

11716119

11716119

11716119

11716119

13924444173325155142873653121951089377109747625689141488

261

6

6

21

21

12986761188

12986761188

2787652

127815

131

10924444173325155113864453121950329376109747625501141487

881842417232435001850153121849609285103447275471137484

44212111

1111

12221127825

11154154

225521512402345

117227

21001

1114

412195110893421587181701

117

12

1312222330012520630876793051210

4724

1115605011206221658268903920271

111162224114424787622331467

1

211113892736449654353970056143163362793464

1119914312623313

11

17

31

1

161

1

1

1111

8214312

111

111

16111111372891294

5110414

11137229

2

64

111217

52736839814492121134726754821967686116269403496163938822502323310

52736839814492121134726754821967686116269403496163938822502323310

2519518366285411472574961312337942814671199469126936716570

106751528226114015241271339924

101451311269512343038244376523314

11

12115111110

180447246141763723342530937394291630193893313641

21511224114511

46812122421020335681182621213

99

366312011115131

1

1110

18

18

2121491155629282094510057

465351101414154257816

166966446151455202241

1210191332681362338351138

761222381235241123

11111

15136

4211131131115

1

1

1

114021956186276810106

715175148627674103

1

4253442162

11

3327128101140864284261913887091851195298118

45122351

1

282211681140864284261913857041851195298117

8474841619342307136125406814015541825757

8474841619342307136125406814015541825757

13266710112321129434430153393136

8235610121151443

2

2

2

8235610121131443

8235610121131443

2412131

62316102111412

5311131128934430013353133

311121881161852

311121881161852

311121881161852

22111213441843352281

22111213441843352281

23431110

11

1

11021

1180

11112118

231

12

942161152564439191772742715215929916953206666526

942161152564439191772742715215929916953206666526

942161152564439191772742715215929916953206666526

2

2

51312

112

21

212

31

31

892141152564438191732742715015929916553205646526

231115113

47101381027102410210962913424215421176602614

42111741437336147016020121245711322643912

12

12

1111316410521

1111316410521

1111316410521

1111316410521

190

7

111

181

1012641

63515365724250819366642054156375795276115085223384753376139538422011635364142883164818611294894127237801261548302647676335

5289728311653352771058916724833303431653319717419821450163191737013306884976

118513191911

118513191911

118513191911

5199378061623192711058616706779294731573229708415871426561451724113095875948

74213

73213

1

681261592139571013303533958413141313874101043820211257105279

1721

81411118331

1941874

3543269748113024613

11111413210123948107

306970102715721624175614533031811242096643479

1012227813303949501065175221

113

12483065144675296285293944725

4212342133121367935

37512211311497

111122174626

11112618111

22462221162421441

132423164124611

14311111211

25622739

1216151591847769801716212716563108204587169

3442

3

1211151191847769801486212712083098190861169

24813715

241961581011

5753101147102491510813595448660643481261145215

179179641541

161623111

1120

11213

3111325702194432559521714

122112111

921

110140935921753743221158726946200

11221627721619288157442076

191135213

21170151

11034718717516

34723

11016417516

51011131418122

51011131418122

1968

1968

161603641

161603641

12197

12197

74142121153382622721

1

132411

1

4412112123313111

31131

1126

341412102163155234626916612371222

112321111861

2111713

11106213112123452673107

221134183125361

11

214221132118225918974281

118

123

1

12111172158186127

111111311111621

1

1

311623126676231012823782387432112870910278040724054

181

1421311135111457142

1

11

1111

1

11

192116223612711552822272528602495908234338973546

16111471111715

12

3292227696179372137221

21741

122

21112

1

1111221

1111221

873492032611574727265996226232372733902951057941619026

15181311721371276505344171451

51815354674651101319143610161

42214574532613111121123651

1

217101321210172133334323

143933217161449123718939192109608171214516

11199113

1

315521117231

1

4520810312633440132210291160134633943658

111

132111

11111

105

113982162631103614863314712526935

2415153621133863887411127857786283629026

4

11

1583212168201192

1

11

1

2187

1211148202161751

1822111211415

4143583322633275094542871014216

111211

193

113050784

1246

320

42

26821742204

1

1

231988151043111020334284357106513

2214871594311102033428227102513

151121504

253406336771331027111427265963864168182020149637163038200162

2111111

3211110780311414

431

188255282667480541067144114446265143240692170833108

66423211062431

2211106

11169

1216261

7106326226241051

342121

10852232148361025328183

41

1111331841

21165223

6

1

1111757231007011722338

4221221634828358322848615119826228

5101158531110102

1116112

11161211051427921191639824195

108

703123213138127026282051555432412

52111111711334741538314311101

116751

2463121293187515212124482

236321112412

1111117

1

11918531522447

635203154318492879242165153126211728

1104

1104

634183154318482779138162151125208728

1

13111121

21

1113198

21

5291331343164317581381501502520671

2111227

1213213

1

11

1

1

11

1212

1111

1111

1111

1

1

1

1511315051221

1511315051221

1511315051221

634609656268507352666252541226757671761042852144847517760670383385856232854254816381756866578099170321078226785838645231

62834740324156843611278217596543955554071593586944558215846154485526145606421881752

3434291402526395692166927252624403573636216621369278399060473238

116

17134

323428536122633569016692625221739311352461317629144372145821204

111129511054361582834

111311311581022351091311567121

624715195310813

5349686332192183714334813457081285117100020492855872228698763155

156247

31231173330013012265190155638

50321112103112111453993931924343

519197630646331552168640413482418194898915963146

110447128414

114944140

91711191166162111

815

674122530812416343631828151725429194425101

9522132911110970

47111177848100867729025821451722198537813902474311018144633105

4710852828092967678825631221502197537811872464291018144428105

1

3175111723142021223

12424311

1

1812

418210268972325452037382310217053488538239438082459597251

13418338525270101309241

4182926897232545173734057213248238268229338082150595210

131801716218376313904820228413483033637107

3261551871

131771716218350313904665228413473033550106

24214747228157715420342428872

1911174719534413111029268

50301186210239313222072

123

11

11

8571112522351151111111

8571112522351151111111

63398162152850702866609645686574488976086784249084712276061537797869692698525041632593528196265111506932626143729834479

1805273778772342421990414576432938896008942711944182183178135891756312576628401542187283

1

1805273778772342421990414576432838896008942711944182183178135891756312576628401542187283

5581771684463251251362981562593865811717311961851351145240996223

434327335543811361601

111161175

1133

11711316728

31421253

64412611339

1

110119349

1231451551182012213628915512326951616168835179635086933646123

1

1

221149418026115911270721

12

12811802141382177709

12112134720819112

211433484985092115

211433484985092115

108169204402837053320874202066886032463976032568835674871126080241432051653159051969391184224419203146168271261

27421006679882361204163420187246534513310115162230188

19752

1121112

144

923224152141098542199287148

1111

8371891885501824131221121

112

11321641

1

7023712225711

351651

114121

65

11130

104272190688830503277243748766555632455776023768328074798925751235326531635159027564161080920948012138547711196

377172325361189311

2172988102731112814715011096051

1

112121147

2

272

5356911146410133014315322323591215292352292

67314498261

202

6533311129471168

112191

47511015375311841111

3291139313372841561192124629243383171703072911122

3112215723956

11612

2226441711399112111

522

114

1742031591157197350202925440950525970902882431837940843710953763841775747726980333632

148212322741115

287517207340058149616313418124271082252533402115320318914

8441061311

32355167986637542411416014271985251417542810861315

15275254649425651391858812856641739532125022874881768452920822602373194404

35172114513423121228119422511

111

154734181548517515661161413815121014

775523381657137135324314521831842395313424072

61212971221571943949257694043187650222

26181311100281124351299713

38135311122145

107281328221096165392143311371895312492327

31134981713112101965111712325071723837007375710011847

64248503163823204783412353

158912831262222211117142065615511856193828512764071252857254290

327301746131709279039399889172654356698181214766796979782279941232132713438594341846692738743204815472279

3141128788237545111066218166

5324515391811775112413814421852062964321756113263

326543741330165278856399652171994356468179814744795879760222129572022562335206312586064643312829514321911

22034223103934216211304611084150268813802660

6155111744939864888174910354324989159845139

124111511569155222111112694971426111

1187219

9653192521872738

21155514205111

42390551

1911362181241218

111540211621745030181

112112

212201172193761413117914441

1917641311791444

1121117219311

1311141118

11111

11118

14

1621032431239395429423011527429962186219128

1621032431239395429423011527429962186219128

1621032431239395429423011527429962186219128

1167555742060

16

11112635913932342371262

2119111

19

125111211

13621239715512314994

30

232111324216794984341892410951

681132719157567124

681132719157567124

681132719157567124

681132719157567124

15661

58113271915251123

10530118545257139601686844525317898052625610472094441149406201628159619525869613737423078448263205146161886597412390

10526318521257132601576844125314898032625410471993831149355922327837719125732213004121802446928198268153199581712003

12

12

12

311111859639368267931286622332150

311111859639368267931286622332150

21111859015546658118865249141

132

312

1

2382169712421417018149

123014549723322629284115841391062323572320416623117785231280

71211412099211618189950208516157562482896258434

134139316508323141195

128074565682141573284527

81

411384080011170539094561100332921066238318

814095840

1343

1

122822913519752685564350439

180961823487218385335821

255131143033

176

112265232

54523202872361

111549710311541112762131316522322

52912448313322419362954022405603150561629103758882265846

23231946469623234334951353861851

2311185813007354126220315922921301487351

10083

32904132

36734523332868429931156339061152610236884572493494

11083308160852876141168641079818728714348385562081978825296470923275872011081102

10419318421138252662344511397138551275374541104

2332235030113546236054260635

7113318136208931234093773924914594003368

221185106426631157617311

111211321111121

175

1

221

221

21225111

21225111

41333311056781236428821535032730751324

41333311056781236428821535032730751324

108432931490520736311606310487187033441347444463915265189643489172746806888934

108432931490520736311606310487187033441347444463915265189643489172746806888934

8994223630660965957522836279466651741814760

8994223630660965957522836279466651741814760

113121114687781702241545104129331811061

113121114687781702241545104129331811061

3109

430175

25

1

1

118619667582140334

16111

1

2

67

21

9

60

4573

31223213

11201

1

47

1

34155211654372295721001061

7728

62

35093648121351774258180880945382570338201574108199902181860458052423595952137130

35093648121351774258180880945382570338201574108199902181860458052423595952137130

6117131811371236122

4513513110611411098105

34993642121331764257580480945382270236961574108197892181682434852422905845137128

1501151267704410120416423021012746872

1501151267704410120416423021012746872

1491141266441010091642230968706872

170156714340

1

11

391

1691212126293113123443835134815299214788

451229427595623144236146108416

45122541678281313371534546213

208

1319819113

211

1771183865092

111221235116572118558561

1

674163310

11

1157

1185

110

53211

38

1152822926163

59511746

1

31

14181185154412282661416847811

38

14181182144091481821416729811

2317

26296384106

3

1171

11371

1

1

1137

1137

1251035131255616711148983281113533402536381357115594293875

4183131214954960890263695963042031771137113843236741

115821382841711455776429116222731510

3142122133534212721823660517910220478218023142550

31111019401410396184667345442388354779181

321537948712381678165510613735052866

13162541223113728510913

321413104271143307989671051180155

2168190123824935523721661

21111

21111

3292111182

3292185

1197

111556119733125010512

111556119733125010512

23646085261980173435457711402866

23646085261980173435457711402866

1452141741844772602618413746471045233326232103125

1452141741844772602618413746471045233326232103125

1211635154371522177931903052142160149106

1173333984192

2118278143119111261322181654443750457

1

111513195861

1061781

532

1931191611646261269

1121388011053

9812155

111114

86

121458414623081274488181

11501

11

841

1

119361

13

11

100598114642432605781565056244508881126143997898574102281346845619215754002578778118730329201336749425134978129

361914131797535136573903411275623413392237662457738114392

6657469881152281

1171324215141010091830117153514501144221084247

1111791129242

1119121055523

73112131732631491166212250113691488745

12322121606873171074466726

31211113685521292347126113641329981

11212042411

3211371092894

2113171054315443311158632256

29295138

11391644043302375491413

111245615741544114764723437825207719174

1816270

311263

2468922711503993162291676810645241392384318971666106238335786135119641852387159293

1

28117111111611212

14111275

37012215023329794035921

101

223949613

33113111

2442292356467104921093403916866403441273326611209346118748940103

27272153721377397156641

363212213553051111

2389223

11116811125223810399222827412

360

4015121266983111

311

6132192231402163

1796153611121651153132

5347836

561221012621430358367453913

45852547384331131135142310311211134

43

146185076913122112734333514051414959

337151133616331211413111

5111153330

81

59111713122281121812311901902623

27258721271606512850148167

771123429102219941281232791765222

1

4

55486203122334892504363333711157391338

4277183126137211411432355

1113128

321194111125151112124741

12611232417694352269319103282143943

866792224228717711

2045013301270682203120145667318425324145116456355023639211848115

1

2131

43151470671916961142657336143221661890735103187155855491478234208

3413511866014

40151470671912951142457336143221661890735102835155852391418220208

111124

1121

121

1

75107913122801247718640482387788456258889481082547776220477428061311321655857983374249491019477222027486550

1512233859

141618

22131210111113

57832529121112121404123

154421540277453208848781719475117058320917269641746

127530563543685897795446943979798795065480352

31021637169702434786517847144915174159148931214512173196875424782458151181845001406056792528

90314174115224

6986287112164589561267143241068219748654671113669832676510662603848918199252794811377310421639

477132403546004583438677694642421543596175606241171109330197672297516736914843134156578901867

25107232318421117843976861242258125050635111

7478127342592353848431549171

1242218102463286

1

331275813711231

1

2

646124168511169401

11

417111113652463424697231547803071042

41111

37412541964130825

211582024148640

1111124624221452172263185542

1092135168736238792106244431634221815

1092135168736238792106144431634211815

11

5169032452311816718521249528681787424111384326332864224

221161213342522

1221141122942104721171534668122

1

102932131241

272762823231101171807114241860178341229803859249059224

13837116513711004149156771521219640631192072557

13837116513711004149156771521219640631192072557

12311213351754394613321608299147918485159

125612

36561633

1193169814560117512168598789

12114

111240398837936741426177758861170

81111

81111

24

24

133319115816487336542756713023601876188269200

313257185215555414423618

1111731221

14114487794242566121020582531826182

328211536

121190171

2

11711633229301011791655564136690541338

11711633229301011791655564136690541338

211512454771229391118967481964437519141

211512454771229391118967481964437519141

11521841252185519195222

1118149119102

231152111418233419921631128

11134842245451282256849

80256192248333226253381862

2819425823314

9433111233108718491407213145270384

9433111233108718491407213145270384

9433111233108718491407213145270384

981

601

11

64121111723874166113744292

1108334511811792018

15525

1

3

61

311

58231111210318648387169437464602418196834130

58231111210318648387169437464602418196834130

1471402109496673285024811653

1471402109496673285024811653

5813158626432518099303591455674676

21202481629324312476

1111115184171

273211761

1137026216962973920512083766

931825612

11617216421910441

11517216421110441

18

1244325327931901512126038

1211118127311556511654197

33207262345471831

1

11148135124781240106

11148135124781240106

2211281371235108820539270468447393240523767167

113108129109165

113108129109165

113108129109165

221128137106977316501132953296392353414649133

21281111031961

21281111031961

9234209194128134067199602171

9234209194128134067199602171

1291434003482380120734035301792274126130

1291434003482380120734035301792274126130

1486901501178214

1486901501178214

11117514522210713673628449361

11117514522210713673628449361

16631433512671503917789261834

16631433512671503917789261834

16631433512671503917789261834

114191196437815081957136107512121885

114191196437815081957136107512121885

114191196437815081957136107512121885

114191196437815081957136107512121885

11121201

121201

121201

121201

11

11

11

111111316551

8

8

8

8

111111316471

111111316471

111111316471

111111316471
